# Mining *Magnaporthe oryzae* sRNAs With Potential Transboundary Regulation of Rice Genes Associated With Growth and Defense Through Expression Profile Analysis of the Pathogen-Infected Rice

**DOI:** 10.3389/fgene.2019.00296

**Published:** 2019-03-29

**Authors:** Hao Zhang, Sifei Liu, Haowu Chang, Mengping Zhan, Qing-Ming Qin, Borui Zhang, Zhi Li, Yuanning Liu

**Affiliations:** ^1^Key Laboratory of Symbolic Computation and Knowledge Engineering, College of Computer Science and Technology, Ministry of Education, Jilin University, Changchun, China; ^2^College of Plant Sciences, Key Laboratory of Zoonosis Research, Ministry of Education, Jilin University, Changchun, China; ^3^Columbia Independent School, Columbia, MO, United States; ^4^School of Computer Science and Technology, Changchun University of Science and Technology, Changchun, China

**Keywords:** *Magnaporthe oryzae*, sRNA, transboundary regulation, rice, SVM

## Abstract

In recent years, studies have shown that phytopathogenic fungi possess the ability of cross-kingdom regulation of host plants through small RNAs (sRNAs). *Magnaporthe oryzae*, a causative agent of rice blast, introduces disease by penetrating the rice tissues through appressoria. However, little is known about the transboundary regulation of *M. oryzae* sRNAs during the interaction of the pathogen with its host rice. Therefore, investigation of the regulation of *M. oryzae* through sRNAs in the infected rice plants has important theoretical and practical significance for disease control and production improvement. Based on the high-throughput data of *M. oryzae* sRNAs and the mixed sRNAs during infection, the differential expressions of sRNAs in *M. oryzae* before and during infection were compared, it was found that expression levels of 366 *M. oryzae* sRNAs were upregulated significantly during infection. We trained a SVM model which can be used to predict differentially expressed sRNAs, which has reference significance for the prediction of differentially expressed sRNAs of *M. oryzae* homologous species, and can facilitate the research of *M. oryzae* in the future. Furthermore, fifty core targets were selected from the predicted target genes on rice for functional enrichment analysis, the analysis reveals that there are nine biological processes and one KEGG pathway associated with rice growth and disease defense. These functions correspond to thirteen rice genes. A total of fourteen *M. oryzae* sRNAs targeting the rice genes were identified by data analysis, and their authenticity was verified in the database of *M. oryzae* sRNAs. The 14 *M. oryzae* sRNAs may participate in the transboundary regulation process and act as sRNA effectors to manipulate the rice blast process.

## Introduction

Rice is one of the most important crops in Asia, its production not only directly affects food security but also has a huge impact on the local economy. Rice blast is a disease caused by *Magnaporthe oryzae* attack, resulting in reduced yield. Because of the importance of this crop, studies on how to control rice blast are very popular.

*M. oryzae* is a heterotrophic fungal pathogen. It can reproduce in the form of spores and spread between rice plants through conidia. After germination, germ tubes form special infection structures called appressoria which will penetrate host’s tissues. Rice has two layers of innate immune mechanisms against *M. oryzae*. The first layer of defense is activated when pathogen-associated molecular patterns (PAMPs) are recognized on the cell surface; thus, this PAMP-triggered immunity (PTI) is activated (Hanae et al., 2006; Shimizu et al., 2010; Su et al., 2012). While *M. oryzae* effectors that inhibit PTI can be recognized by rice R proteins, which is the second layer of defense and called effector-triggered immunity (ETI) (Liu et al., 2013). However, the mechanism by which *M. oryzae* infects rice may not be limited to the molecular aspect, but can also to genetic aspects, such as RNA silencing.

RNA silencing or RNA interference (RNAi) is a regulatory mechanism that specifically inhibits the expression of target genes. In this process, double-stranded RNA (dsRNA) is processed into sRNA under the action of the enzyme called RNase III. One of the small RNA (sRNA) strands joins into an effector complex RISC (RNA-induced silencing complexes) capable of degrading the target RNA, therefore inhibiting the mRNA level of the target gene and the subsequent protein biosynthesis (Brodersen and Voinnet, 2006). sRNA is a short, non-coding RNA that specifically expresses in certain physiological stages of an organism and plays an important role in regulation based on its target-mRNA cleavage. For example, miR393b is specifically expressed in the reproductive stage, it cleaves target genes to inhibit flower development; miR172c is specifically expressed in the vegetative stage to inhibit the expression of LOC_Os07g13170.1 (AP2 domain-containing protein) (Yijun et al., 2012). RNAi plays a key role in gene regulation in a variety of eukaryotes, and studies have shown that hairpin RNAs (hpRNAs) can effectively silence the expression of target genes (Chen et al., 2015).

In recent years, studies have found that RNA silencing exists not only in the interior of organisms but also in the interaction between organisms. Some sRNAs can be transferred between interacting organisms and induce gene silencing in the counter party; this mechanism is known as cross-kingdom RNAi (Cai et al., 2018a). Arne et al. (2013) showed that in addition to proteins, sRNA molecules can also act as effectors to inhibit host immunity. sRNAs bind to AGO proteins and direct RISCs to complementary genes to induce gene silencing. sRNAs of *Botrytis cinerea* can inhibit the host plant’s immune response to the pathogen at an early stage of infection by this mechanism, demonstrating that sRNAs can act as effectors by silencing host defense-associated genes, thereby disarming plant immunity and achieving infection (Arne et al., 2013). Later, sRNA Bc-siR37 of the pathogen was found to be delivered to plant cells to silence host immune genes (Wang et al., 2017). This cross-kingdom RNAi mechanism has proven in the process of fungal infection of plants. Based on the results aforementioned, it is safe to infer that the transboundary sRNA regulation of *M. oryzae* of rice may exists.

Current researches on rice blast prevention are mostly focused on the internal immune regulation of rice or *M. oryzae*. For example, rice endogenous miRNAs, such as miR169, play regulatory roles in rice immunity against *M. oryzae* (Li et al., 2017). Endogenous sRNAs of *M. oryzae* may also play a role in the transcriptional regulation of some genes, since these sRNAs are involved in regulation of *M. oryzae* stress responses when plant conditions change (Raman et al., 2013). However, little is known about transboundary regulation of rice by *M. oryzae* sRNAs during rice interaction with the rice blast fungus. Here, the study analyzes the transboundary regulation of *M. oryzae* sRNA on rice based on high-throughput data. By screening the upregulated *M. oryzae* sRNAs during infection and their target rice genes, as well as analyzing the functional enrichment of the target rice genes, the study identifies *M. oryzae* sRNAs that may directly participate in the regulation process of rice infection, which inhibit the growth and the survival of the rice. The study provides a theoretical basis for disease control and yield increase in rice, as well as new ideas for innovative study on the processes of plant infection by other phytopathogenic fungi.

## Data and Methods

**FIGURE 1:**
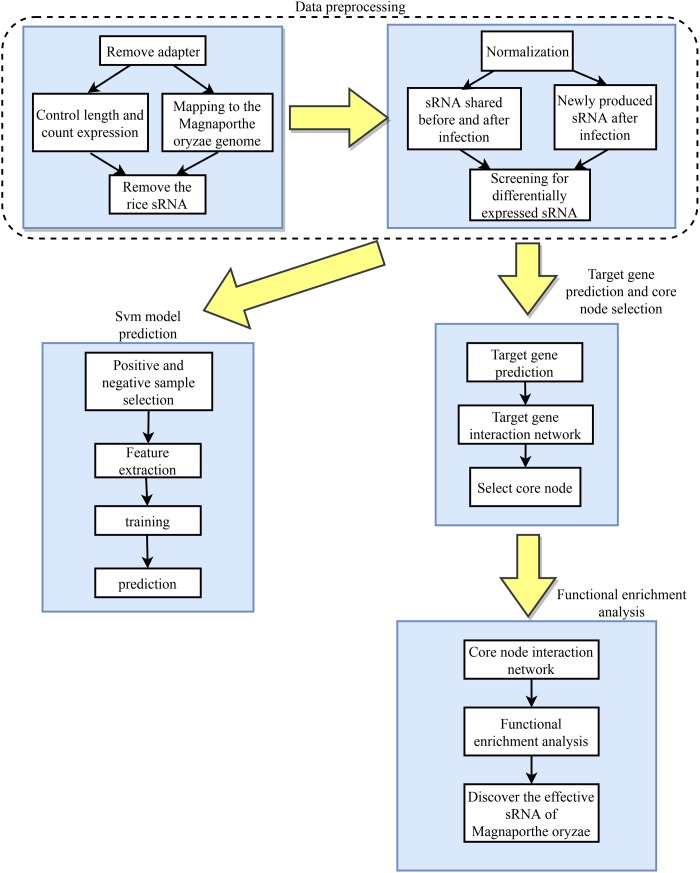
Overall design roadmap for this work.

The differentially expressed rice blast sRNAs during infection were first analyzed through a big data-based method and then the related software was used to predict their target genes in rice. Functional enrichment analysis on the targets were performed to predict gene functions closely related to rice growth and defense, which then allows the experimenters to identify the *M. oryzae* sRNAs that enforce a transboundary regulation on rice during the infection process. The overview of the design roadmap for this work is illustrated in Figure 1.

### Data Source

The sRNA raw data of *M. oryzae* cultured on a complete medium for 16 h, the mixed sRNA raw data of the rice samples infected by *M. oryzae* for 72 h (Raman et al., 2013), the data of wild-type rice leaves 48 h after water treatment, the data of wild-type rice leaves 48 h after *M. Oryzae* infection (Chujo et al., 2013), as well as the *M. oryzae* and rice genomic data and rice mRNA data were used in the analyses. All of these were obtained from NCBI. For the mixed sRNA raw data, we were able to find the mixed sRNA raw data of rice samples infected by *M. oryzae* for 0, 72, and 96 h. *M. oryzae* invades the host through the infection pegs from appressoria. For the molecules that act as effectors during infection, the expression of these molecules takes a certain time. For the rice sample infected by *M. oryzae* for 0 h, because the infection time is too short, many molecules have no time to express, so this sample is not suitable for use. In addition, because LMg96 infects for too long, some molecules have been degraded, so it is not suitable for use, too. In contrast, for the rice sample infected by *M. oryzae* for 72 h, the expression of the molecule is the most active, so this sample is most suitable for subsequent analysis. The data downloaded from NCBI is in SRA format, they had to be converted to FASTQ format before the data could be processed.

### Data Preprocessing

Data preprocessing is a key step in data analysis and has a significant impact on the effectiveness of subsequent analysis. At present, the preprocessing of sRNA high-throughput data is mainly divided into the following steps: filtering, alignment, and normalization. First, high-quality data is obtained by removing adapters and low-quality reads. Second, mapping the data to the genome, the types and corresponding counts of sRNAs that can be mapped to the genome are obtained. Finally, the counts of sRNAs are normalized, and the standardized counts are used to find differentially expressed sRNAs, or to analyze the distribution, variance, and bias of the data (Tam et al., 2015).

#### Adapter and Quality Information

**FIGURE 2:**
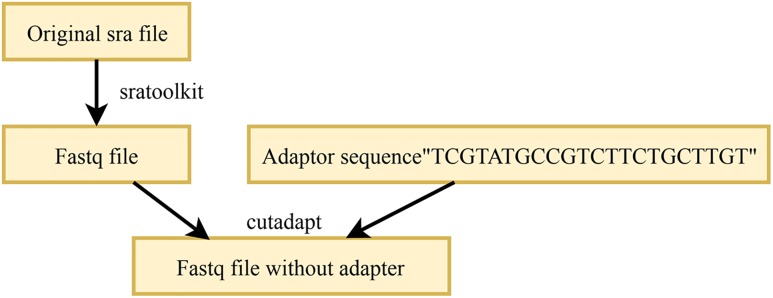
Strategy for removing the adapters in raw RNAs.

In the acquired high-throughput sequencing data, each sRNA sequence is of the same length; this is because Illumina performing adaptor ligation in the process of library preparation (Tam et al., 2015). Therefore, almost every sequence obtained has an adapter sequence of varying lengths. To obtain the correct sRNA sequence, these adapters should be removed. The existing adapter removal tools are mainly FASTX-toolkit, Cutadapt, and Trimmomatic. In this work, Cutadapt^1^ was used to remove the adapters, which requires the knowledge of the adapter sequence used for the high-throughput data. The *M. oryzae* sRNA and the mixed sRNA data used in this work were high-throughput sequencing data based on the Illumina platform and incorporated the international standard adapter “TCGTATGCCGTCTTCTGCTTGT”. In the resultant FASTQ file after removal of the adapter by Cutadapt, the sRNA sequences no longer contain the adapter. The process of the removal of the adapters in sRNAs is illustrated in Figure 2.

**FIGURE 3:**
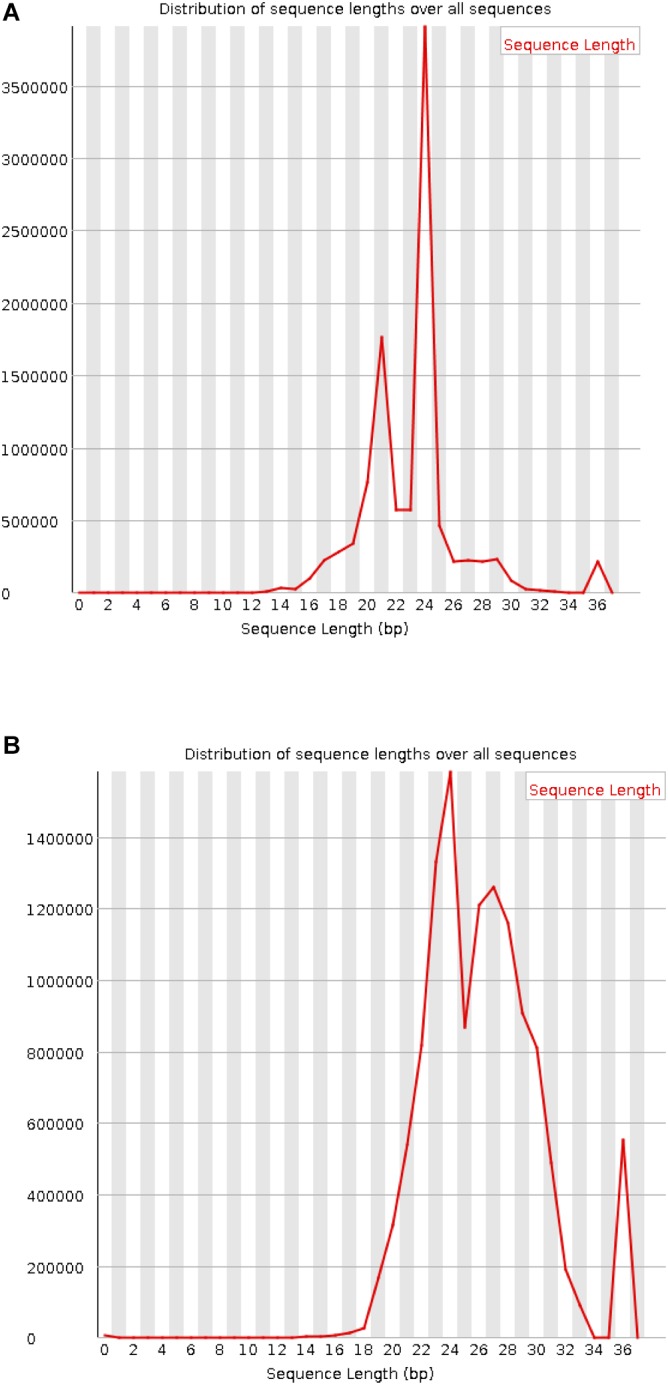
Length distribution of the processed sRNAs. **(A)** Mixed sRNAs after infection. **(B)** *M. oryzae* sRNAs.

In the data source article, although the wrong sequencing data were filtered out by the script, there was no quality control operation on the data (Raman et al., 2013). The length distribution of the preprocessed data is shown in Figure 3. The length distribution of the sRNA in the mixed or infection data after removing the adapter with two peaks between 21 and 27 displays a high quality (Figure 3A). However, an error is shown in the processed *M. oryzae* sRNA data (Figure 3B) due to the *M. oryzae* data and the infection data originated from the same place and using the same adapter. The experimental error may be due to the strict setting of the parameters in the process of removing the adapter. Adapters with more than three mismatches were not removed. For the sake of experimental rigor, the false positive result is minimized in the adapter identification, and the setting of the mismatch parameter is rigorous here. When the length control was performed later, the sequence corresponding to this part of the error was discarded.

#### Data Mapping to the *M. oryzae* Genome

**FIGURE 4:**
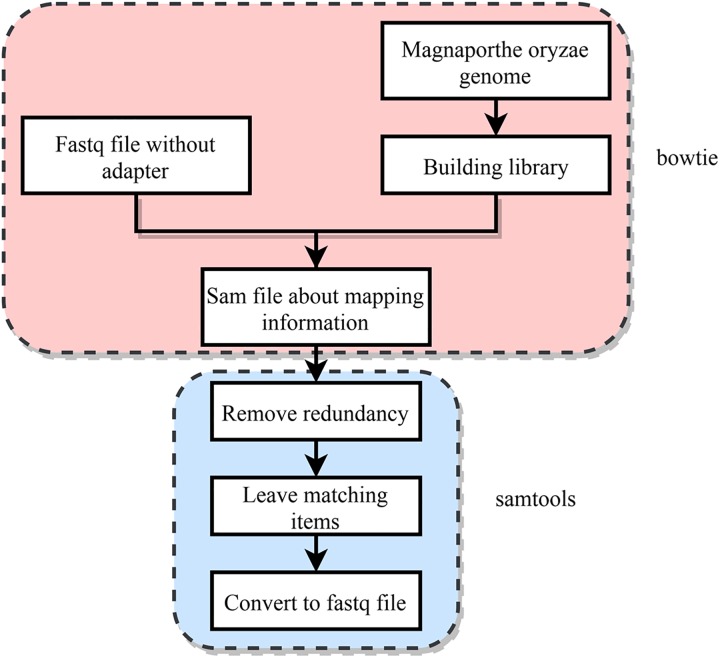
Strategy for mapping data to the *M. oryzae* genome.

The main research object of this work is to identify *M. oryzae* sRNAs with potential cross-kingdom regulation, it is thus necessary to find the sRNA sequences of *M. oryzae* that are differentially expressed before and during host infection. However, some contamination is mixed in the *M. oryzae* sRNA sequences’ data, and in the mixed data during infection. In addition to the *M. oryzae* sRNA sequences and contamination, many sRNA sequences of rice plants also appeared in the data. The *M. oryzae* sRNA and mixed sRNA data of infection were mapped to the genome of *M. oryzae* by using the RNA data without adapter sequences, and the portion of the sRNAs belonging to *M. oryzae* was obtained. In this section, two tools, Bowtie and Samtools, were used. First, the index library of the *M. oryzae* genome obtained from NCBI was constructed, and the index package was obtained. Both of these steps used bowtie (Jun et al., 2012). Then the FASTQ file of the *M. oryzae* sRNAs and the FASTQ file of the mixed sRNAs of infection were mapped to the *M. oryzae* genome. The process was strictly matched, the mismatch parameter was set to 0, and all matching information was output as a SAM file. Then SAMtools software was used to process the generated SAM file, filter out the redundancies, and yield the FASTQ file that only retains the matching sequence (Li et al., 2009). The process is shown in Figure 4.

#### Length Control and Sequence Expression Statistics

In order to find the differentially expressed *M. oryzae* sRNAs, we need to know the expression level of each sRNA sequence before and during infection, that is, the number of each sequence in the data file. However, since each sequence may match multiple locations of the genome during the mapping process, and all matches will eventually be output to the result file, resulting in an increase in the number of sequences in the resultant file, it is inaccurate to count the expression level in the mapped result file. To solve this problem, the following measures were taken:

First, a script was used to extract the sequence in the FASTQ file from the *M. oryzae* sRNAs that had been removed from the adapter but had not been mapped to the genome, to obtain a text file only containing the sequence. Then, a script was used to control the length and count the number of occurrences (expression amount) of each sequence. Since the length of miRNA (a sRNA that inhibits gene expression) is between 18 and 25 nt, it is believed that the length of the *M. oryzae* sRNA targeting rice genes and producing transboundary regulation in rice should also be in this range. Therefore, only the *M. oryzae* sRNA sequences ranging from 18 nt to 25 nt in length were retained. Finally, a table file (herein referred to as file A) containing the sRNA sequences, the lengths of the sequences, and the expression levels of the sequences was obtained.

The FASTQ file of *M. oryzae* sRNA data mapped to the *M. oryzae* genome was handled by a script to obtain a text file containing only the sequences; then, a script was used to control the length and to remove duplicates to obtain a file, which only contained sequences with a length between 18 and 25 nt (herein referred to as file B, which does not contain length and expression information, i.e., only sequences, and each sequence appears only once).

Finally, the following processing was performed on file A and file B through a script: if a line in file A appears in file B, then the line is reserved; if a line is in file A, but its sequence does not appear in file B, then that line is discarded.

**FIGURE 5:**
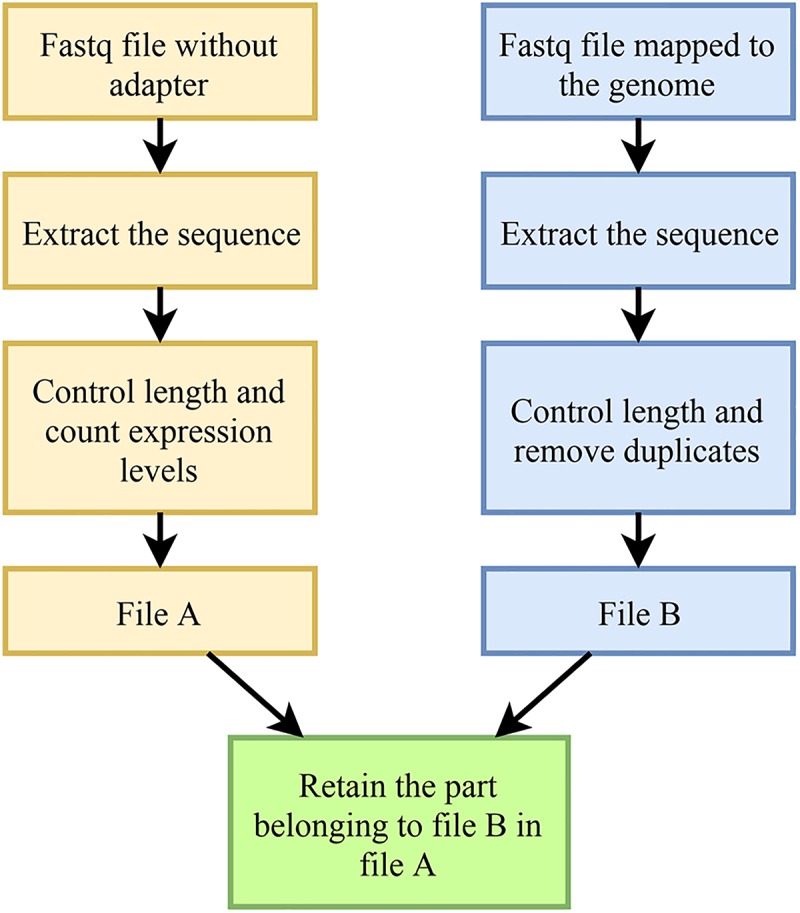
Flowchart for length control and expression count statistics.

The above process is illustrated as Figure 5. In the final file, each sequence can be mapped to the genome of *M. oryzae*, and the expression amount is accurate. The mixed sRNA data file of infection was also processed by the above method. Finally, the obtained *M. oryzae* sRNAs during infection could be mapped to the genome of *M. oryzae*, and the expression amount was accurate.

#### Elimination of Rice sRNA

*M. oryzae* has the same sequence as some sRNAs in rice; thus, only mapping the data to the *M. oryzae* genome cannot guarantee that all the sRNAs obtained belong to *M. oryzae*. Some rice sRNAs may be mistaken as belonging to *M. oryzae* because their sequences are identical to some *M. oryzae* sRNAs. This mistaken identity will bring errors to future experiments. To address this issue, the sRNAs that could be mapped to the *M. oryzae* genome were then mapped to the rice genome and those sRNAs that could be mapped to the rice genome were removed; thus, the final sRNAs were solely from *M. oryzae* genome.

### A Normalization Method Based on 3/4 Quantile Data

To find the *M. oryzae* sRNAs differentially expressed during infection, the *M. oryzae* expression data must be normalized before and during infection to make it comparable. Because the number of species of *M. oryzae* sRNAs before and during infection is quite different, and the number of *M. oryzae* sRNA species during infection is much less than before infection, if the per million counts normalization method is used, then after normalization, the magnitude of the change in data expression during infection will be much larger than that before infection, which makes it impossible to accurately find the sRNAs with a substantial increase in the expression level during infection. To solve this problem, we adopted a normalization method based on a 3/4 quantile. First, the sample data were sorted according to the expression levels from high to low; then, the sRNA ranked at 3/4 was obtained. This sRNA’s expression amount can represent the lower level of expression in this sample; then, the expression amounts of other sRNAs were converted into multiples of the expression amount of this sRNA. Because the expression levels of the data in the sample were all converted to the multiple of the sample’s lower expression level, this method not only avoids the influence of different cardinalities between different samples, but also evades the influence of the differences in the number of species between different samples, thereby making different samples comparable. This normalization method was used to process the data of *M. oryzae* sRNAs before and during infection. Then, the 6,100 sRNAs that appeared before and during infection were extracted to compare their changes in the expression level.

### The Selection of Differentially Expressed sRNAs

**FIGURE 6:**
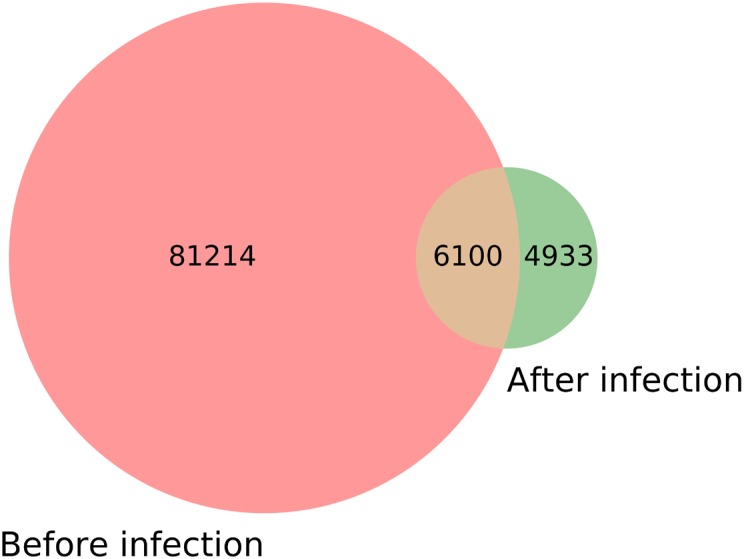
Comparison of the *M. oryzae* sRNA species before and during infection.

Through statistics, it was found that the species of *M. oryzae* sRNAs before and during infection did not completely coincide (Table 1). According to the statistics, there were 87,314 species of *M. oryzae* sRNAs before infection, and 11,033 species during infection. There were 6,100 species of *M. oryzae* sRNAs presenting in the two stages; moreover, 4,933 species of *M. oryzae* sRNAs were newly produced during infection (Figure 6). From the above statistics, most of the *M. oryzae* sRNAs disappeared during rice infection. In order to find *M. oryzae* sRNAs with a significant increase in expression in infection, the 11,033 *M. oryzae* sRNA species were divided into two parts for analysis.

**Table 1:** Statistical information on sRNA species before and during infection.

Raw data	sRNA species before mapping	sRNA species mapped to the *M. oryzae* genome	Species after removal of rice sRNA
*M. oryzae*	350,402	87,453	87,314
Infected 72 h mixed data	120,8231	11,194	11,033


The first part is the 6,100 species of *M. oryzae* sRNAs presenting before and during infection. To clearly observe the changes of sRNA expression levels, the normalization method based on the 3/4 quantile was used to extract the data of *M. oryzae* sRNAs before and after infection; then, a total of 6,100 sRNAs that presented before and during infection were extracted to compare their changes in the expression levels. Since sRNAs regulate the target genes by inhibiting their expression, we thus only screened for the sRNAs which are significantly higher expressed than before infection and their expressions are more than the others after infection. The increase in the expression level was measured by the growth rate using the following Equation (1):

(1) $$Growth\_Rate=\frac{count_{after}-count_{before}}{count_{before}}$$

**FIGURE 7:**
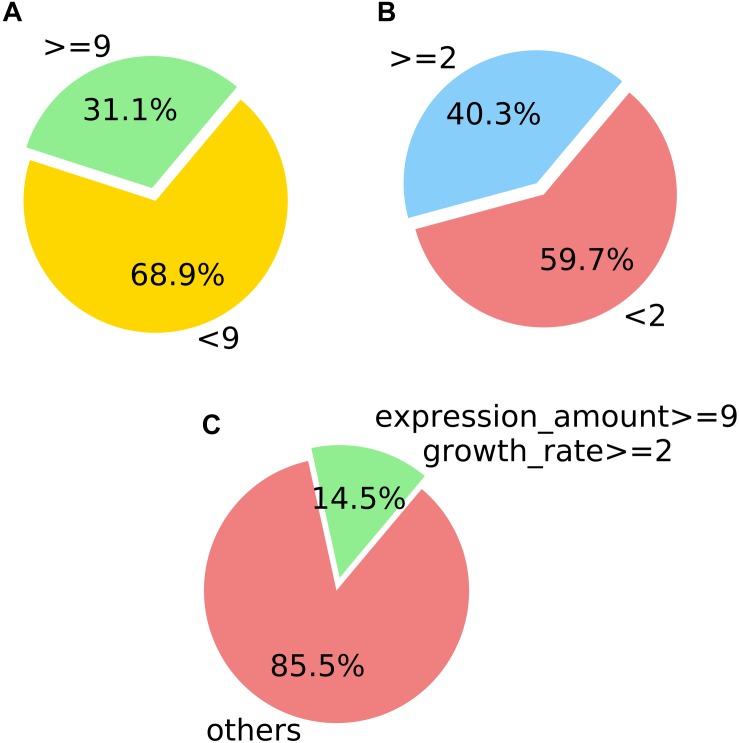
Counting statistics screened by the expression level and expression growth rate of *M. oryzae* sRNA during rice infection. **(A)** Screening by the expression level. **(B)** Screening by the expression growth rate. **(C)** Screening by the two indicated conditions.

All the values in Equation (1) were standardized. For the sRNA which showed growth during infection, the results of screening based on the expression level after infection and the growth rate of expression, are, respectively, shown in Figure 7A,B. Both sRNAs serve to illustrate screening conditions, and the distribution of screening results is shown in Figure 7C.

**FIGURE 8:**
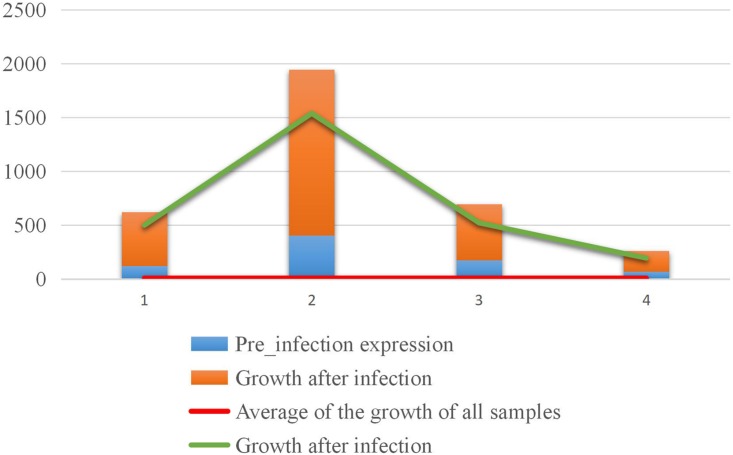
Comparison of the expression levels of the four sRNAs with higher expression levels before and in infection.

**FIGURE 9:**
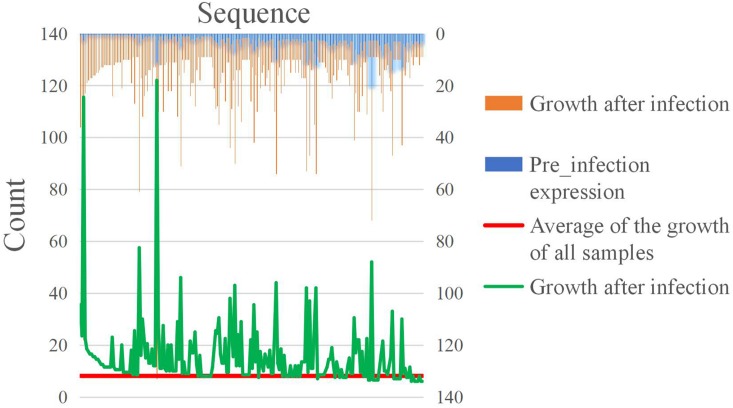
Comparison of the expression of the other 216 sRNAs before and in infection.

In the positively growing sRNAs, the percentage of sRNA with an expression level greater than or equal to 9 during infection was less than 50% (Figure 7A). The percentage of sRNA with a growth rate greater than or equal to 2 was also less than 50% (Figure 7B). Only a small number (220) of the sRNAs met both the criteria. These 220 sRNAs can be considered as the most obvious part of the difference in expression. For four sRNAs of the 220 sRNAs were much high expressed than the others after our sRNA expression level standardization, the comparisons of the four sRNA expressions and the other 216 sRNAs’ expressions are shown in Figure 8, 9, respectively. By comparison of the expression level during infection to that before infection, the growth ratio of these 220 sRNAs in infection is extremely high (Figure 8, 9). From the apex of the blue columnar column and the apex of the whole column, it is obvious that the expression levels are dramatically higher than those before infection.

The second part is the 4,933 *M. oryzae* sRNAs newly produced in infection. This part of sRNAs were extracted from the standardized data and sorted from high to low in expression level. One-hundred-forty-six sRNAs were selected according to their expression levels.

A total of 366 *M. oryzae* sRNAs screened in the above two parts were used as differentially expressed sRNA for the subsequent analysis.

### SVM Model for Predicting Differential Expression of *M. oryzae* sRNAs

#### Selection of Positive and Negative Samples

The SVM is a classic supervised machine learning model. SVM model was used to predict differentially expressed and non-differentially expressed sRNAs. A total of 366 differentially expressed *M. oryzae* sRNAs were used as positive samples and were removed from all *M. oryzae* sRNAs. The remaining *M. oryzae* sRNAs were then used to randomly select the negative samples, and the number of sRNAs in the negative sample was twice as that of the positive sample.

#### Feature Extraction and Normalization

The negative and positive sample labels were set to 0 and 1, respectively. The positive and negative samples were extracted into a file. The RNAfold tool^2^ was used to predict the secondary structure to obtain free energy information. The sequences and their free energy for feature extraction were then extracted. In the process of feature extraction, 1–25 bits (the sequences less than 25 bits in length need to be complemented with N), along with the length, GC percentage, free energy, 5′ mo_base, 5′ di_base, 3′ mo_base, 3′ di_base, and motif of each sequence, were extracted as features, and the features were represented by the letters encoded in binary. In addition, the features were normalized by the normalization method of min-max as shown in Equation (2):

(2) $$y=\frac{x-\min}{\max-\min}$$

Where *x* is the original value, *y* is the normalized result, and min and max represent the minimum and maximum values of this feature. Also, the feature value is scaled between 0 and 1.

#### Model Training

In this step, a total numbers of 3/4 of the positive and negative samples were extracted as the training set. The grid search and fivefold cross-validation method were used to train the parameters (Zhang et al., 2009). The Radial Basis Function (RBF) kernel function expressed as the following Equation (3) was used to train the model. The RBF kernel function is a kind of kernel function, which is used to map the linear indivisible problem in the low dimension to a high dimension, thus making the problem linearly separable. Let <*w*′, *x*′> be the inner product of the high-dimensional space, *x*′ is the high-dimensional vector transformed by *x, w*′ is the constant obtained by transforming the constant w in the low-dimensional space, and there is *K*(*w, x*) lets *g*(*x*) = *K*(*w, x*) + *b* be the same as *f*(*x*′) = <*w*′, *x*′> + *b*, and *K*(*w, x*) is the kernel function. The RBF kernel function is a kernel function that satisfies this condition.

(3) $$K(x,\;x^{\prime })=\exp\left(\frac{-||x-x^{\prime }|{|}^{2}}{2\sigma^{2}}\right)$$

### Target Gene Prediction

To identify *M. oryzae* sRNAs that may play a role in regulation of rice growth and defense, rice mRNAs were used as the targets to predict target genes for the 366 differentially expressed *M. oryzae* sRNAs. In this step, the TAPIR, a target gene prediction tool (Xie et al., 2012), was used. In the process of target gene prediction, the input of the sRNA file must be a FASTA file and the bases in the sRNA sequence should be A, U, G, and C; thus, the sequences of these 366 sRNAs were extracted and the base T was converted to U by a script. The sequence files were then converted to FASTA files before target gene prediction by the TAPIR tool.

### Selection of the Core Node

For each target gene predicted, its corresponding GeneID was found in the mRNA file of rice. After de-duplicating the found GeneIDs, a total number of 1,121 GeneIDs were obtained. For the target rice genes, in order to know whether they are differentially expressed, we added experiments. We obtained the data of wild-type rice leaves 48 h after water treatment and the data of wild-type rice leaves 48 h after *M. Oryzae* infection from NCBI (Chujo et al., 2013), compared the two sets of data and screened the target genes. Considering that the infection time of the comparison data (48 h) is shorter than the infection time of our sRNA data (72 h), and the plant may produce some stress response due to self-protection, we retained target genes with reduced expression levels after infection and target genes with a slight increase in expression levels (less than 0.2) after infection. A total of 685 target genes were retained, and the proportion of target genes with decreased expression was 69.3%. All the 685 GeneIDs were imported into the STRING database^3^ where 586 GeneIDs could be identified and the corresponding interaction network was given. The obtained tabular data of the interaction network from the STRING database (without retaining node annotations) were shown in Supplementary Table S1, which provides the two nodes corresponding to each edge of the network, as well as the proteins corresponding to the nodes, and the score of the relationship’s credibility between the nodes (Supplementary Table S1).

Because of the huge number of nodes, it is difficult to locate the obvious enrichment. Therefore, it is necessary to select the core nodes of the network and to find the obvious enrichment and pathways through the interaction network of the core nodes. To reach this goal, the core nodes of the network were selected through the following two key steps:

Step 1: To obtain the subgraph by the score of the credibility through the relationship between the nodes in the network. In the interaction network, the smaller the score of the credibility of the relationship between nodes, the less possibility of the interaction between the two nodes. Therefore, the threshold of the score was set as 0.6, which means that if the score no less than 0.6, the interaction between the corresponding nodes is authentic. By following this criterium, only the edges with a score of no less than 0.6 were selected. The graph composed of these edges is a sub-graph with higher credibility in the entire interaction network.

Step 2: To select the core nodes based on the degree of the nodes. In an interaction network, the higher a node degree, the more nodes it interacts with. Based on the subgraph obtained in the first step, the degree of each node in the subgraph was counted and sorted the nodes according to the degree from large to small. Finally, a total of 50 nodes were selected as core nodes.

## Results

### The Core Node’s Regulation Network

In this research, the authors re-imported the GeneIDs of the 50 core nodes into the STRING database and the resultant interaction network of these 50 core nodes was listed in Supplementary Table S2. Because the *p*-value of the network is 1.59e-8, the network was provided with high accuracy. In the functional enrichment results of the network, there were 15 Biological Processes (GO), 5 Molecular Functions (GO), 5 Cellular Component (GO), 2 KEGG Pathways, 5 PFAM Protein Domains, and 5 INTERPRO Protein Domains and Features. We mainly analyzed the 15 Biological Processes (GO) and 2 KEGG Pathways. Among all the results given, the false discovery rate was less than 0.05. The BP and KEGG enrichment results were shown in Supplementary Table S3 and Table 2. In the 15 Biological Processes (GO), the order of error detection was ranked from low to high. These biological processes are the cellular protein modification process, chromatin organization, organelle organization, chromosome organization, cellular process, chromatin remodeling, chromatin modification, phosphate-containing compound metabolic process, primary metabolic process, cellular metabolic process, defense response, organic substance metabolic process, response to stimulus, the mitogen-activated protein kinase (MAPK) cascade, and protein phosphorylation. Two KEGG pathways are inositol phosphate metabolism and phosphatidylinositol signaling system.

**Table 2:** The enriched KEGG Pathways.

Pathway ID No.	Pathway description	Observed gene count	False discovery rate	Matching proteins in your network (IDs)
562	Inositol phosphate metabolism	2	0.0418	LOC_Os05g03610.1, LOC_Os08g33200.1
4070	Phosphatidylinositol signaling system	2	0.0418	LOC_Os05g03610.1, LOC_Os08g33200.1


### Regulatory Pathways Associated With Rice Growth and Defense

In the 15 biological processes, the defense response, and the stimulating response directly affect the ability of plants (here, rice) to cope with external unfavorable factors, thereby affecting rice survival. When plants are stimulated by pathogenic bacterial infection, injury, temperature, drought, salinity, permeability, ultraviolet radiation, ozone, and reactive oxygen species, MAPK is activated. After translation, it is regulated by phosphorylation (Zhang and Klessig, 2001). Therefore, the MAPK cascade and protein phosphorylation are also closely related to rice’s ability to cope with factors of life-threatening in its growth environment.

For the top five GO biological processes, the gene sets enriched in the pathways were imported into the DAVID database and found the lower functional pathways corresponding to the five pathways. The verification results show that there are the biological processes for defense response and the biological processes that positively regulate growth rate in the lower regulatory pathways of these five pathways. In the lower regulation of chromatin organization, organelle organization, chromosome organization, and cellular processes, there are three biological processes: response to temperature stimulation, cell proliferation, and multicellular biological development. In other words, the top five functional enrichments are closely related to rice defense and growth. For the two KEGG Pathways, inositol phosphate metabolism is closely related to biosensory extracellular stimulation.

**FIGURE 10:**
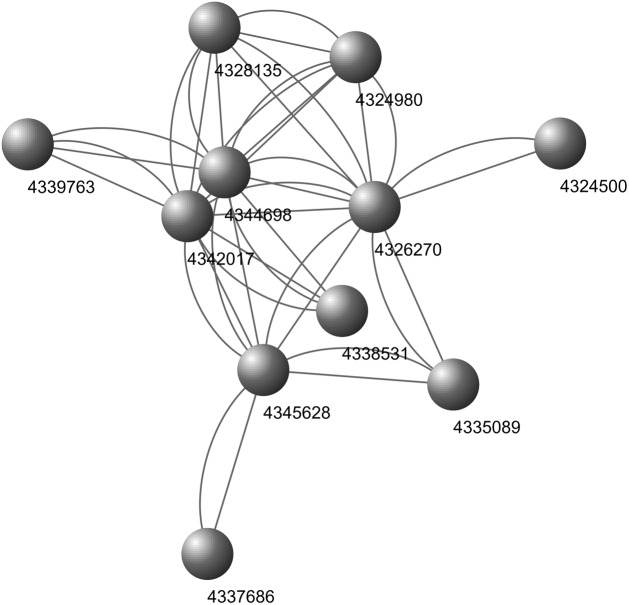
Interaction network of the enriched genes in the biological processes. Figures: GeneIDs.

Nine biological processes related to rice defense response and growth process and one KEGG pathway were found in our work. Interestingly, the 9 biological process pathways already contain all the genes that can be enriched into the 15 biological processes in core nodes. Further analysis demonstrates that the enriched genes display close interactions (Figure 10). These pathways may be used to identify sRNA effectors that facilitate rice infection by the pathogen.

### Discovery of *M. oryzae* sRNAs as Potential Effectors in the Infection Process

**FIGURE 11:**
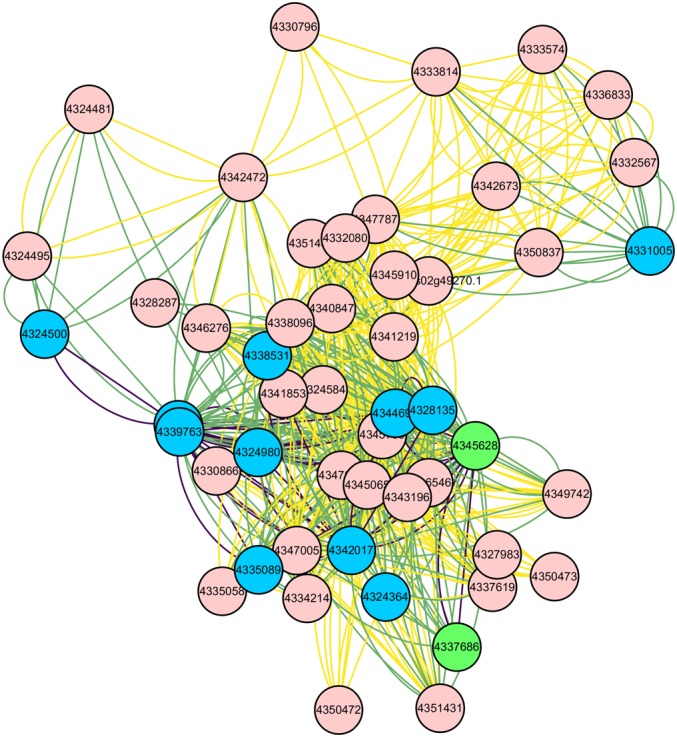
Interaction network of the 50 core nodes. The blue nodes represent 11 genes enriched into 9 biological processes, and the green nodes represent 2 genes enriched into one KEGG Pathway. The relationships among these 13 nodes are represented by purple lines. The relationships among these 13 nodes and other nodes are represented by the green lines.

Through the above 9 biological processes and one KEGG Pathway related to rice growth and defense, the IDs of the proteins enriched in these pathways can be found (Supplementary Table S3 and Table 2). These proteins correspond to 13 genes in the core nodes (Supplementary Table S2), and these genes also distribute in the 50 core nodes’ interaction networks (Figure 11). From the results of target gene prediction, 14 *M. oryzae* sRNAs targeting these 13 rice genes were found. These 14 sRNA sequences can be obtained in the *Magnaporthe* Next-Gen Sequence sRNA database^4^ (Table 3 and Supplementary Table S4). The comparison of the two columns of LMg0 and LMg72 shows that these 14 sRNAs are actively expressed at 72 h post infection, which further confirms our viewpoint about these sRNAs may serve as effectors that facilitate rice infection by the pathogen.

**Table 3:** The resultant 14 sRNA sequences in the *Magnaporthe* Next-Gen Sequence sRNA database (Clip version).

Sequence	Len	Sum	Average	Max	Min (>0)	LMg0	LMg72
TAGACTTTGATCTGAGCAA	19	1029	257	754	2	0	754
TGGCAAGTATAGGCCTGTA	19	1145	95	467	7	0	467
AGCCTGACGATGTCGTTGATGCT	23	627	314	610	17	0	610
TGGAAGCGTTAGGGGCTTTG	20	811	116	395	2	0	395
ACGATCTGCAGCGCTTTTCGT	21	3446	246	1293	2	0	1293
ACGATCTGCAGCGCTTTTCG	20	2254	225	1109	2	0	682
CAGGCGAGGGCGCTCTGCT	19	2108	192	627	2	214	574
GCACTTGGAAGCATGGGGCT	20	845	282	682	26	0	682
TAGCGGGGAACTGTGCATG	19	700	140	467	30	0	467
GGACATGGTTTTGGACGAA	19	1236	88	467	9	0	467
TACAAGGGACGAAGTGTCT	19	1349	104	646	2	0	646
AACCCGGAGGTCTCTGGA	18	2291	153	722	6	0	431
AGTGGTCGTAGACCGCCTGA	20	1933	161	1126	5	0	359
CAGGCAGTTGGACTTGACCT	20	889	296	539	28	322	539


*Len, length (nt); Sum, the sum of abundance; Average, the average of abundance; Max, maximum abundance; Min (>0), minimum abundance; LMg0, the name of the sample; LMg72, the name of the sample*.

### The SVM Model Prediction Results

**FIGURE 12:**
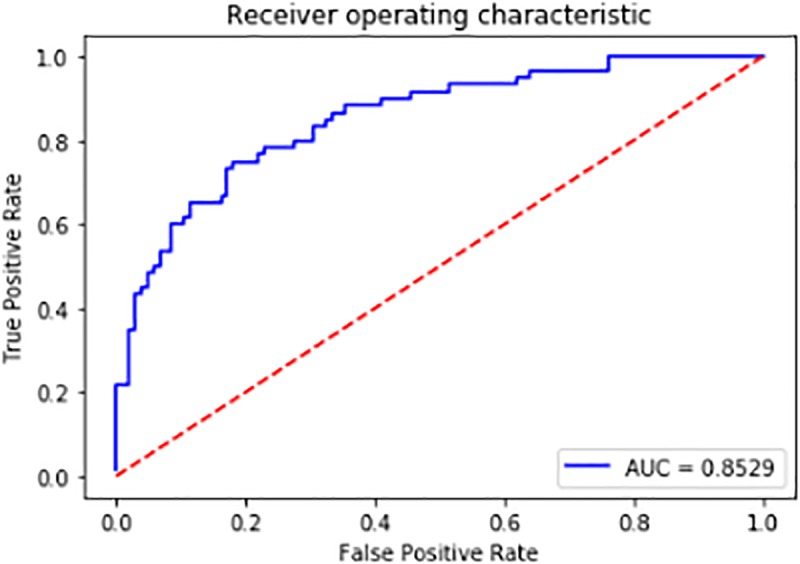
ROC Curve.

We used the remaining 1/4 of the positive and negative samples as the test set. The accuracy of the final model prediction reached as high as 83%. The Receiver Operating Characteristic (ROC) curve is shown in Figure 12. The further the curve is from the diagonal line, the better the model performs. The value of the Area Under the Curve (AUC) can evaluate the model intuitively, the larger the value of AUC is, the better the model is at discriminating between positives and negatives. It can be seen from the figure that the curve is far from the diagonal line and the AUC value is 0.85. Thus the model can be used to select the differentially expressed sRNAs after obtaining the sRNAs mapped to the pathogen genome. The selection of the differential-expressed sRNAs in other species of fungal plant pathogens can also refer to this model.

## Discussion

### The sRNAs Involved in the Transboundary Regulation

Cumulated evidence indicates that transboundary regulations of pathogens on the host plants exist (Shimizu et al., 2010; Su et al., 2012). Based on the findings, the study assumes that the pathogenic mechanism also exists during rice infection by *M. oryzae*. However, little is known about whether sRNA affects rice growth or defense. This study found that during rice infection, although most of the *M. oryzae* sRNA disappeared, some sRNA remained in the infected rice, and some were upregulated. All the *M. oryzae* sRNAs existing in the rice tissue have the potential to interact with the host rice. Based on the characteristics of sRNA inhibition of gene expression, only the upregulated *M. oryzae* sRNAs in infection were considered in this process. The sRNA transport and regulation between plants and pathogens is bidirectional. After the pathogen invades plant, the expression levels of some sRNAs in host plants increase, conversely inhibiting the pathogen gene-expression to resist the invasion by the pathogens (Cai et al., 2018b). In this work, the study omitted the analysis on the roles of rice sRNA due to the demand of more focused research on the pathogenic side. The roles of *M. oryzae* sRNAs with decreased expression levels during infection also need to be further investigated.

### The Limitations of Target Gene Prediction Software

For the *M. oryzae* sRNAs with increased expression level in infection, the study predicted their target genes in rice and directly analyzed the functional enrichments of these target genes. The results in this study are based on the target gene prediction software, which may be affected by the algorithms in the software. Therefore, the results obtained may be incomplete because the data collected are based on only one target gene prediction software. If multiple software packages for target gene prediction are used for a comprehensive analysis, there may be more results of target gene predictions. As the actual targeting relationship in the organism is complicated, the prediction results given by the software may not be accurate. The screening for the actual target genes should give more credible results if the prediction genes can be further validated via experimental data.

### The Method of Selecting Core Nodes

After obtaining the interaction network of all the target genes, the subgraphs were screened out based on the credibility score of the relationship between the nodes and selected the core nodes according to the degree of the nodes in the subgraph. This method of screening the core nodes is simple, which points to the need for more efficient or universal algorithms to select the core nodes.

### The Application of Machine Learning Models

The authors trained the SVM model to predict differentially expressed sRNAs. However, the predicted results of this model are only sRNAs with significantly increased expression levels in the infection. Although sRNAs with decreased expression levels may also play an important role in the infection process, this model does not apply to the down-regulated sRNAs. In addition, there are multiple machine learning models; many of them are suitable for classifying samples. It is not known which model can achieve the best results. Different models can be used to predict differentially expressed sRNAs, and their results can be compared to determine which machine learning model can achieve the best prediction.

### The Significance of Finding Effector sRNA

Using the method of high-throughput data presented in this study, 14 *M. oryzae* sRNAs were identified, which may act as effectors to silence rice genes and cause disease. The data used in this work were experimentally validated and the authenticity of these 14 sRNAs was confirmed in the *Magnaporthe* Next-Gen Sequence sRNA database. However, since this study is based on the hypothesis that a cross-kingdom RNAi mechanism exists between *M. oryzae* and rice. This hypothesis requires further biological experiments to verify. Because this mechanism may exist during the infestation of other fungi on plants, this study lays a foundation for the discovery of sRNA effectors in other fungi. In addition, when various phytopathogenic fungi infect plants, there is little known on whether a certain similarity exists among their sRNA effectors. To clarify the similarities and functions of the sRNA effectors from diverse fungal pathogens may constitute an intriguing research direction. If the relationships among these fungal sRNAs are determined, the discovery will provide an important theoretical basis for new ideas on the prevention and control of plant diseases.

## Data Availability

Publicly available datasets were analyzed in this study. This data can be found here:

https://www.ncbi.nlm.nih.gov/Traces/study/?acc=SRX214117;

https://www.ncbi.nlm.nih.gov/Traces/study/?acc=SRX214123;

https://www.ncbi.nlm.nih.gov/Traces/wgs/AACU03?val=AACU03.1;

https://www.ncbi.nlm.nih.gov/Traces/wgs/AACU03?val=LVCG01.1;

https://www.ncbi.nlm.nih.gov/nuccore/?term=Oryza+sativa;

https://www.ncbi.nlm.nih.gov/geo/query/acc.cgi?acc=GSM973470;

https://www.ncbi.nlm.nih.gov/geo/query/acc.cgi?acc=GSM973471.

## Author Contributions

YL and HZ conceived and directed the project. SL, HC, and BZ obtained the raw data and interpreted the data. HZ, SL, MZ, HC, and ZL conducted the data analysis and interpreted the results. HZ, SL, HC, and ZL helped to design the study and reviewed the data. HZ, SL, BZ, and Q-MQ wrote and/or edited the manuscript. All authors drafted and reviewed the manuscript and approved it for publication.

## Conflict of Interest Statement

The authors declare that the research was conducted in the absence of any commercial or financial relationships that could be construed as a potential conflict of interest.

## Footnotes

1 https://cutadapt.readthedocs.io/en/stable/

2 http://rna.tbi.univie.ac.at/cgi-bin/RNAWebSuite/RNAfold.cgi

3 https://version-10-5.string-db.Org/

4 https://mpss.danforthcenter.org/dbs/index.php?SITE=mg_sRNA
