# Supplementary material for: Mining Magnaporthe oryzae sRNAs With Potential Transboundary Regulation of Rice Genes Associated With Growth and Defense Through Expression Profile Analysis of the Pathogen-Infected Rice
Source: Front Genet. 2019 Mar 29;10:296. doi: 10.3389/fgene.2019.00296 (PMC6449695; doi:10.3389/fgene.2019.00296)
Supplement: Supplementary file 1 [file Table_1.DOCX]

Supplementary Material

# Supplementary Tables

Table S1. Interaction network table of target genes

| node1 | node2 | node1 accession | node2 accession | score |
| --- | --- | --- | --- | --- |
| 4323903 | 4339379 | LOC_Os01g19820.1 | LOC_Os05g45020.1 | 0.458 |
| 4324082 | 4332080 | LOC_Os01g06320.1 | LOC_Os03g11910.1 | 0.683 |
| 4324082 | 4333079 | LOC_Os01g06320.1 | LOC_Os03g29260.1 | 0.618 |
| 4324082 | 4338096 | LOC_Os01g06320.1 | LOC_Os05g11990.1 | 0.42 |
| 4324082 | 4341853 | LOC_Os01g06320.1 | LOC_Os06g46600.1 | 0.572 |
| 4324082 | 4343196 | LOC_Os01g06320.1 | LOC_Os07g28800.1 | 0.476 |
| 4324364 | 4326546 | LOC_Os01g69030.2 | LOC_Os01g53920.1 | 0.634 |
| 4324364 | 4330971 | LOC_Os01g69030.2 | LOC_Os02g54820.1 | 0.965 |
| 4324364 | 4332258 | LOC_Os01g69030.2 | LOC_Os03g14540.1 | 0.573 |
| 4324364 | 4338096 | LOC_Os01g69030.2 | LOC_Os05g11990.1 | 0.671 |
| 4324364 | 4342410 | LOC_Os01g69030.2 | LOC_Os07g05620.1 | 0.421 |
| 4324364 | 4344584 | LOC_Os01g69030.2 | LOC_Os08g03570.1 | 0.573 |
| 4324364 | 4345065 | LOC_Os01g69030.2 | LOC_Os08g14990.1 | 0.634 |
| 4324364 | 4345708 | LOC_Os01g69030.2 | LOC_Os08g34650.1 | 0.634 |
| 4324364 | 4347095 | LOC_Os01g69030.2 | LOC_Os09g25540.1 | 0.634 |
| 4324364 | 4348853 | LOC_Os01g69030.2 | LOC_Os10g32980.1 | 0.519 |
| 4324428 | 4331991 | LOC_Os01g53070.1 | LOC_Os03g10780.1 | 0.55 |
| 4324428 | 4337228 | LOC_Os01g53070.1 | LOC_Os04g55700.1 | 0.785 |
| 4324481 | 4324495 | LOC_Os03g17100.1 | LOC_Os01g05900.1 | 0.986 |
| 4324481 | 4324500 | LOC_Os03g17100.1 | LOC_Os01g05630.1 | 0.986 |
| 4324481 | 4324980 | LOC_Os03g17100.1 | LOC_Os01g67970.1 | 0.645 |
| 4324481 | 4326151 | LOC_Os03g17100.1 | LOC_Os01g01689.1 | 0.47 |
| 4324481 | 4327384 | LOC_Os03g17100.1 | LOC_Os01g62230.1 | 0.986 |
| 4324481 | 4342472 | LOC_Os03g17100.1 | LOC_Os07g06980.1 | 0.657 |
| 4324481 | 4344819 | LOC_Os03g17100.1 | LOC_Os08g08210.1 | 0.483 |
| 4324481 | H2B.9 | LOC_Os03g17100.1 | LOC_Os05g49860.1 | 0.986 |
| 4324495 | 4324481 | LOC_Os01g05900.1 | LOC_Os03g17100.1 | 0.986 |
| 4324495 | 4324500 | LOC_Os01g05900.1 | LOC_Os01g05630.1 | 0.645 |
| 4324495 | 4326270 | LOC_Os01g05900.1 | LOC_Os01g16414.1 | 0.473 |
| 4324495 | 4327384 | LOC_Os01g05900.1 | LOC_Os01g62230.1 | 0.54 |
| 4324495 | 4327790 | LOC_Os01g05900.1 | LOC_Os01g13570.1 | 0.678 |
| 4324495 | 4341655 | LOC_Os01g05900.1 | LOC_Os06g43790.1 | 0.683 |
| 4324495 | 4341683 | LOC_Os01g05900.1 | LOC_Os06g44060.1 | 0.769 |
| 4324495 | 4342472 | LOC_Os01g05900.1 | LOC_Os07g06980.1 | 0.774 |
| 4324495 | 4343299 | LOC_Os01g05900.1 | LOC_Os07g31450.1 | 0.463 |
| 4324495 | 4344819 | LOC_Os01g05900.1 | LOC_Os08g08210.1 | 0.492 |
| 4324495 | 4346109 | LOC_Os01g05900.1 | LOC_Os08g41630.1 | 0.53 |
| 4324495 | H2B.9 | LOC_Os01g05900.1 | LOC_Os05g49860.1 | 0.54 |
| 4324500 | 4324481 | LOC_Os01g05630.1 | LOC_Os03g17100.1 | 0.986 |
| 4324500 | 4324495 | LOC_Os01g05630.1 | LOC_Os01g05900.1 | 0.645 |
| 4324500 | 4326270 | LOC_Os01g05630.1 | LOC_Os01g16414.1 | 0.473 |
| 4324500 | 4327384 | LOC_Os01g05630.1 | LOC_Os01g62230.1 | 0.617 |
| 4324500 | 4341655 | LOC_Os01g05630.1 | LOC_Os06g43790.1 | 0.683 |
| 4324500 | 4342472 | LOC_Os01g05630.1 | LOC_Os07g06980.1 | 0.774 |
| 4324500 | 4343299 | LOC_Os01g05630.1 | LOC_Os07g31450.1 | 0.463 |
| 4324500 | 4344819 | LOC_Os01g05630.1 | LOC_Os08g08210.1 | 0.492 |
| 4324500 | 4346109 | LOC_Os01g05630.1 | LOC_Os08g41630.1 | 0.53 |
| 4324500 | H2B.9 | LOC_Os01g05630.1 | LOC_Os05g49860.1 | 0.894 |
| 4324511 | 4326546 | LOC_Os01g05620.1 | LOC_Os01g53920.1 | 0.404 |
| 4324511 | 4327667 | LOC_Os01g05620.1 | LOC_Os01g40590.1 | 0.404 |
| 4324511 | 4328725 | LOC_Os01g05620.1 | LOC_Os02g12440.1 | 0.404 |
| 4324511 | 4330701 | LOC_Os01g05620.1 | LOC_Os02g50970.1 | 0.404 |
| 4324511 | 4331134 | LOC_Os01g05620.1 | LOC_Os02g57080.1 | 0.404 |
| 4324511 | 4333060 | LOC_Os01g05620.1 | LOC_Os03g28300.1 | 0.404 |
| 4324511 | 4334921 | LOC_Os01g05620.1 | LOC_Os04g01874.1 | 0.404 |
| 4324511 | 4335824 | LOC_Os01g05620.1 | LOC_Os04g34250.1 | 0.404 |
| 4324511 | 4337251 | LOC_Os01g05620.1 | LOC_Os04g56090.1 | 0.404 |
| 4324511 | 4338080 | LOC_Os01g05620.1 | LOC_Os05g11750.1 | 0.404 |
| 4324511 | 4338096 | LOC_Os01g05620.1 | LOC_Os05g11990.1 | 0.628 |
| 4324511 | 4339142 | LOC_Os01g05620.1 | LOC_Os05g40770.1 | 0.404 |
| 4324511 | 4341066 | LOC_Os01g05620.1 | LOC_Os06g29080.1 | 0.404 |
| 4324511 | 4341568 | LOC_Os01g05620.1 | LOC_Os06g41980.1 | 0.404 |
| 4324511 | 4342396 | LOC_Os01g05620.1 | LOC_Os07g05370.1 | 0.404 |
| 4324511 | 4342410 | LOC_Os01g05620.1 | LOC_Os07g05620.1 | 0.404 |
| 4324511 | 4343699 | LOC_Os01g05620.1 | LOC_Os07g38810.1 | 0.404 |
| 4324511 | 4343832 | LOC_Os01g05620.1 | LOC_Os07g41140.1 | 0.404 |
| 4324511 | 4345065 | LOC_Os01g05620.1 | LOC_Os08g14990.1 | 0.404 |
| 4324511 | 4345628 | LOC_Os01g05620.1 | LOC_Os08g33200.1 | 0.806 |
| 4324511 | 4345708 | LOC_Os01g05620.1 | LOC_Os08g34650.1 | 0.404 |
| 4324511 | 4347005 | LOC_Os01g05620.1 | LOC_Os09g23740.1 | 0.806 |
| 4324511 | 4347095 | LOC_Os01g05620.1 | LOC_Os09g25540.1 | 0.404 |
| 4324511 | 4352691 | LOC_Os01g05620.1 | LOC_Os12g40419.1 | 0.404 |
| 4324511 | 4352904 | LOC_Os01g05620.1 | LOC_Os12g44090.1 | 0.404 |
| 4324584 | 4324980 | LOC_Os01g11920.1 | LOC_Os01g67970.1 | 0.652 |
| 4324584 | 4326270 | LOC_Os01g11920.1 | LOC_Os01g16414.1 | 0.646 |
| 4324584 | 4326546 | LOC_Os01g11920.1 | LOC_Os01g53920.1 | 0.691 |
| 4324584 | 4328135 | LOC_Os01g11920.1 | LOC_Os02g03060.1 | 0.553 |
| 4324584 | 4329448 | LOC_Os01g11920.1 | LOC_Os02g30620.1 | 0.513 |
| 4324584 | 4330744 | LOC_Os01g11920.1 | LOC_Os02g51540.1 | 0.531 |
| 4324584 | 4332080 | LOC_Os01g11920.1 | LOC_Os03g11910.1 | 0.807 |
| 4324584 | 4338531 | LOC_Os01g11920.1 | LOC_Os05g29030.1 | 0.516 |
| 4324584 | 4339763 | LOC_Os01g11920.1 | LOC_Os05g50970.1 | 0.516 |
| 4324584 | 4339944 | LOC_Os01g11920.1 | LOC_Os06g02900.1 | 0.531 |
| 4324584 | 4341853 | LOC_Os01g11920.1 | LOC_Os06g46600.1 | 0.656 |
| 4324584 | 4342017 | LOC_Os01g11920.1 | LOC_Os06g49430.1 | 0.516 |
| 4324584 | 4342410 | LOC_Os01g11920.1 | LOC_Os07g05620.1 | 0.403 |
| 4324584 | 4342472 | LOC_Os01g11920.1 | LOC_Os07g06980.1 | 0.475 |
| 4324584 | 4343196 | LOC_Os01g11920.1 | LOC_Os07g28800.1 | 0.669 |
| 4324584 | 4344698 | LOC_Os01g11920.1 | LOC_Os08g06060.1 | 0.516 |
| 4324584 | 4345065 | LOC_Os01g11920.1 | LOC_Os08g14990.1 | 0.691 |
| 4324584 | 4345708 | LOC_Os01g11920.1 | LOC_Os08g34650.1 | 0.691 |
| 4324584 | 4346276 | LOC_Os01g11920.1 | LOC_Os08g44050.1 | 0.478 |
| 4324584 | 4347095 | LOC_Os01g11920.1 | LOC_Os09g25540.1 | 0.691 |
| 4324584 | 4348561 | LOC_Os01g11920.1 | LOC_Os10g26010.1 | 0.433 |
| 4324584 | 4349742 | LOC_Os01g11920.1 | LOC_Os11g04600.1 | 0.595 |
| 4324584 | 4350837 | LOC_Os01g11920.1 | LOC_Os11g38170.1 | 0.418 |
| 4324584 | 4351008 | LOC_Os01g11920.1 | LOC_Os11g42350.1 | 0.455 |
| 4324584 | 4351457 | LOC_Os01g11920.1 | LOC_Os12g04410.1 | 0.595 |
| 4324587 | 4345365 | LOC_Os01g11946.1 | LOC_Os08g27010.1 | 0.403 |
| 4324660 | 4326546 | LOC_Os01g52380.1 | LOC_Os01g53920.1 | 0.404 |
| 4324660 | 4327667 | LOC_Os01g52380.1 | LOC_Os01g40590.1 | 0.404 |
| 4324660 | 4328725 | LOC_Os01g52380.1 | LOC_Os02g12440.1 | 0.404 |
| 4324660 | 4330701 | LOC_Os01g52380.1 | LOC_Os02g50970.1 | 0.404 |
| 4324660 | 4331134 | LOC_Os01g52380.1 | LOC_Os02g57080.1 | 0.404 |
| 4324660 | 4333060 | LOC_Os01g52380.1 | LOC_Os03g28300.1 | 0.404 |
| 4324660 | 4334921 | LOC_Os01g52380.1 | LOC_Os04g01874.1 | 0.404 |
| 4324660 | 4335824 | LOC_Os01g52380.1 | LOC_Os04g34250.1 | 0.404 |
| 4324660 | 4337251 | LOC_Os01g52380.1 | LOC_Os04g56090.1 | 0.404 |
| 4324660 | 4338080 | LOC_Os01g52380.1 | LOC_Os05g11750.1 | 0.404 |
| 4324660 | 4338096 | LOC_Os01g52380.1 | LOC_Os05g11990.1 | 0.628 |
| 4324660 | 4339142 | LOC_Os01g52380.1 | LOC_Os05g40770.1 | 0.404 |
| 4324660 | 4341066 | LOC_Os01g52380.1 | LOC_Os06g29080.1 | 0.404 |
| 4324660 | 4341568 | LOC_Os01g52380.1 | LOC_Os06g41980.1 | 0.404 |
| 4324660 | 4342396 | LOC_Os01g52380.1 | LOC_Os07g05370.1 | 0.404 |
| 4324660 | 4342410 | LOC_Os01g52380.1 | LOC_Os07g05620.1 | 0.404 |
| 4324660 | 4343699 | LOC_Os01g52380.1 | LOC_Os07g38810.1 | 0.404 |
| 4324660 | 4343832 | LOC_Os01g52380.1 | LOC_Os07g41140.1 | 0.404 |
| 4324660 | 4345065 | LOC_Os01g52380.1 | LOC_Os08g14990.1 | 0.404 |
| 4324660 | 4345628 | LOC_Os01g52380.1 | LOC_Os08g33200.1 | 0.806 |
| 4324660 | 4345708 | LOC_Os01g52380.1 | LOC_Os08g34650.1 | 0.404 |
| 4324660 | 4347005 | LOC_Os01g52380.1 | LOC_Os09g23740.1 | 0.806 |
| 4324660 | 4347095 | LOC_Os01g52380.1 | LOC_Os09g25540.1 | 0.404 |
| 4324660 | 4352691 | LOC_Os01g52380.1 | LOC_Os12g40419.1 | 0.404 |
| 4324660 | 4352904 | LOC_Os01g52380.1 | LOC_Os12g44090.1 | 0.404 |
| 4324709 | 4332380 | LOC_Os01g56570.1 | LOC_Os03g16290.1 | 0.405 |
| 4324709 | 4342645 | LOC_Os01g56570.1 | LOC_Os07g09690.1 | 0.439 |
| 4324709 | STLP1 | LOC_Os01g56570.1 | LOC_Os01g63970.1 | 0.436 |
| 4324980 | 4324481 | LOC_Os01g67970.1 | LOC_Os03g17100.1 | 0.645 |
| 4324980 | 4324584 | LOC_Os01g67970.1 | LOC_Os01g11920.1 | 0.652 |
| 4324980 | 4326239 | LOC_Os01g67970.1 | LOC_Os01g15480.1 | 0.478 |
| 4324980 | 4326270 | LOC_Os01g67970.1 | LOC_Os01g16414.1 | 0.611 |
| 4324980 | 4326546 | LOC_Os01g67970.1 | LOC_Os01g53920.1 | 0.703 |
| 4324980 | 4328135 | LOC_Os01g67970.1 | LOC_Os02g03060.1 | 0.659 |
| 4324980 | 4330484 | LOC_Os01g67970.1 | LOC_Os02g47970.1 | 0.471 |
| 4324980 | 4337063 | LOC_Os01g67970.1 | LOC_Os04g53410.1 | 0.465 |
| 4324980 | 4337360 | LOC_Os01g67970.1 | LOC_Os04g57560.1 | 0.565 |
| 4324980 | 4338096 | LOC_Os01g67970.1 | LOC_Os05g11990.1 | 0.666 |
| 4324980 | 4341655 | LOC_Os01g67970.1 | LOC_Os06g43790.1 | 0.414 |
| 4324980 | 4342017 | LOC_Os01g67970.1 | LOC_Os06g49430.1 | 0.718 |
| 4324980 | 4342410 | LOC_Os01g67970.1 | LOC_Os07g05620.1 | 0.41 |
| 4324980 | 4342472 | LOC_Os01g67970.1 | LOC_Os07g06980.1 | 0.81 |
| 4324980 | 4343299 | LOC_Os01g67970.1 | LOC_Os07g31450.1 | 0.44 |
| 4324980 | 4344698 | LOC_Os01g67970.1 | LOC_Os08g06060.1 | 0.718 |
| 4324980 | 4344819 | LOC_Os01g67970.1 | LOC_Os08g08210.1 | 0.815 |
| 4324980 | 4345065 | LOC_Os01g67970.1 | LOC_Os08g14990.1 | 0.703 |
| 4324980 | 4345708 | LOC_Os01g67970.1 | LOC_Os08g34650.1 | 0.703 |
| 4324980 | 4347095 | LOC_Os01g67970.1 | LOC_Os09g25540.1 | 0.703 |
| 4324980 | 4351431 | LOC_Os01g67970.1 | LOC_Os12g03990.1 | 0.459 |
| 4324980 | H2B.9 | LOC_Os01g67970.1 | LOC_Os05g49860.1 | 0.426 |
| 4324980 | LOC_Os03g64080.1 | LOC_Os01g67970.1 | LOC_Os03g64080.1 | 0.788 |
| 4325022 | 4326546 | LOC_Os01g67770.1 | LOC_Os01g53920.1 | 0.463 |
| 4325022 | 4332241 | LOC_Os01g67770.1 | LOC_Os03g14260.1 | 0.426 |
| 4325022 | 4335058 | LOC_Os01g67770.1 | LOC_Os04g08740.1 | 0.861 |
| 4325022 | 4338096 | LOC_Os01g67770.1 | LOC_Os05g11990.1 | 0.478 |
| 4325022 | 4341965 | LOC_Os01g67770.1 | LOC_Os06g48720.1 | 0.61 |
| 4325022 | 4345065 | LOC_Os01g67770.1 | LOC_Os08g14990.1 | 0.463 |
| 4325022 | 4345708 | LOC_Os01g67770.1 | LOC_Os08g34650.1 | 0.463 |
| 4325022 | 4347095 | LOC_Os01g67770.1 | LOC_Os09g25540.1 | 0.463 |
| 4325329 | 4328300 | LOC_Os01g08700.1 | LOC_Os02g05510.1 | 0.421 |
| 4325329 | 4329677 | LOC_Os01g08700.1 | LOC_Os02g35180.1 | 0.421 |
| 4325329 | 4330868 | LOC_Os01g08700.1 | LOC_Os02g53140.1 | 0.964 |
| 4325329 | 4333882 | LOC_Os01g08700.1 | LOC_Os03g50310.1 | 0.421 |
| 4325329 | 4346315 | LOC_Os01g08700.1 | LOC_Os08g44510.1 | 0.644 |
| 4326027 | 4328305 | LOC_Os01g25820.1 | LOC_Os02g05620.1 | 0.416 |
| 4326027 | 4341965 | LOC_Os01g25820.1 | LOC_Os06g48720.1 | 0.461 |
| 4326027 | 4342017 | LOC_Os01g25820.1 | LOC_Os06g49430.1 | 0.431 |
| 4326027 | 4344698 | LOC_Os01g25820.1 | LOC_Os08g06060.1 | 0.431 |
| 4326027 | 4351005 | LOC_Os01g25820.1 | LOC_Os11g42200.1 | 0.603 |
| 4326151 | 4324481 | LOC_Os01g01689.1 | LOC_Os03g17100.1 | 0.47 |
| 4326151 | 4328135 | LOC_Os01g01689.1 | LOC_Os02g03060.1 | 0.471 |
| 4326151 | 4330902 | LOC_Os01g01689.1 | LOC_Os02g53680.1 | 0.764 |
| 4326151 | 4331991 | LOC_Os01g01689.1 | LOC_Os03g10780.1 | 0.712 |
| 4326151 | 4344306 | LOC_Os01g01689.1 | LOC_Os07g48360.1 | 0.663 |
| 4326151 | 4344819 | LOC_Os01g01689.1 | LOC_Os08g08210.1 | 0.584 |
| 4326239 | 4324980 | LOC_Os01g15480.1 | LOC_Os01g67970.1 | 0.478 |
| 4326239 | 4346276 | LOC_Os01g15480.1 | LOC_Os08g44050.1 | 0.458 |
| 4326239 | 4347823 | LOC_Os01g15480.1 | LOC_Os09g38450.1 | 0.425 |
| 4326270 | 4324495 | LOC_Os01g16414.1 | LOC_Os01g05900.1 | 0.473 |
| 4326270 | 4324500 | LOC_Os01g16414.1 | LOC_Os01g05630.1 | 0.473 |
| 4326270 | 4324584 | LOC_Os01g16414.1 | LOC_Os01g11920.1 | 0.646 |
| 4326270 | 4324980 | LOC_Os01g16414.1 | LOC_Os01g67970.1 | 0.611 |
| 4326270 | 4326546 | LOC_Os01g16414.1 | LOC_Os01g53920.1 | 0.606 |
| 4326270 | 4327384 | LOC_Os01g16414.1 | LOC_Os01g62230.1 | 0.473 |
| 4326270 | 4328135 | LOC_Os01g16414.1 | LOC_Os02g03060.1 | 0.466 |
| 4326270 | 4330744 | LOC_Os01g16414.1 | LOC_Os02g51540.1 | 0.4 |
| 4326270 | 4331144 | LOC_Os01g16414.1 | LOC_Os02g57190.1 | 0.803 |
| 4326270 | 4335089 | LOC_Os01g16414.1 | LOC_Os04g09860.1 | 0.697 |
| 4326270 | 4337317 | LOC_Os01g16414.1 | LOC_Os04g56980.1 | 0.603 |
| 4326270 | 4338096 | LOC_Os01g16414.1 | LOC_Os05g11990.1 | 0.582 |
| 4326270 | 4339944 | LOC_Os01g16414.1 | LOC_Os06g02900.1 | 0.4 |
| 4326270 | 4342017 | LOC_Os01g16414.1 | LOC_Os06g49430.1 | 0.519 |
| 4326270 | 4342472 | LOC_Os01g16414.1 | LOC_Os07g06980.1 | 0.433 |
| 4326270 | 4343299 | LOC_Os01g16414.1 | LOC_Os07g31450.1 | 0.435 |
| 4326270 | 4344698 | LOC_Os01g16414.1 | LOC_Os08g06060.1 | 0.519 |
| 4326270 | 4345065 | LOC_Os01g16414.1 | LOC_Os08g14990.1 | 0.606 |
| 4326270 | 4345628 | LOC_Os01g16414.1 | LOC_Os08g33200.1 | 0.514 |
| 4326270 | 4345708 | LOC_Os01g16414.1 | LOC_Os08g34650.1 | 0.606 |
| 4326270 | 4346276 | LOC_Os01g16414.1 | LOC_Os08g44050.1 | 0.549 |
| 4326270 | 4347005 | LOC_Os01g16414.1 | LOC_Os09g23740.1 | 0.514 |
| 4326270 | 4347095 | LOC_Os01g16414.1 | LOC_Os09g25540.1 | 0.606 |
| 4326270 | 4348805 | LOC_Os01g16414.1 | LOC_Os10g31970.1 | 0.435 |
| 4326270 | 4352699 | LOC_Os01g16414.1 | LOC_Os12g40490.1 | 0.428 |
| 4326270 | H2B.9 | LOC_Os01g16414.1 | LOC_Os05g49860.1 | 0.473 |
| 4326270 | IRL7 | LOC_Os01g16414.1 | LOC_Os03g11360.2 | 0.45 |
| 4326378 | 4331991 | LOC_Os01g40980.1 | LOC_Os03g10780.1 | 0.558 |
| 4326378 | 4337228 | LOC_Os01g40980.1 | LOC_Os04g55700.1 | 0.401 |
| 4326378 | 4338689 | LOC_Os01g40980.1 | LOC_Os05g32600.1 | 0.899 |
| 4326378 | 4342544 | LOC_Os01g40980.1 | LOC_Os07g08050.1 | 0.722 |
| 4326378 | 4344306 | LOC_Os01g40980.1 | LOC_Os07g48360.1 | 0.476 |
| 4326439 | 4327790 | LOC_Os01g01302.1 | LOC_Os01g13570.1 | 0.404 |
| 4326439 | 4329037 | LOC_Os01g01302.1 | LOC_Os02g18450.1 | 0.722 |
| 4326439 | 4345365 | LOC_Os01g01302.1 | LOC_Os08g27010.1 | 0.847 |
| 4326439 | 4346508 | LOC_Os01g01302.1 | LOC_Os09g07570.1 | 0.477 |
| 4326546 | 4324364 | LOC_Os01g53920.1 | LOC_Os01g69030.2 | 0.634 |
| 4326546 | 4324511 | LOC_Os01g53920.1 | LOC_Os01g05620.1 | 0.404 |
| 4326546 | 4324584 | LOC_Os01g53920.1 | LOC_Os01g11920.1 | 0.691 |
| 4326546 | 4324660 | LOC_Os01g53920.1 | LOC_Os01g52380.1 | 0.404 |
| 4326546 | 4324980 | LOC_Os01g53920.1 | LOC_Os01g67970.1 | 0.703 |
| 4326546 | 4325022 | LOC_Os01g53920.1 | LOC_Os01g67770.1 | 0.463 |
| 4326546 | 4326270 | LOC_Os01g53920.1 | LOC_Os01g16414.1 | 0.606 |
| 4326546 | 4327983 | LOC_Os01g53920.1 | LOC_Os02g01170.1 | 0.655 |
| 4326546 | 4328135 | LOC_Os01g53920.1 | LOC_Os02g03060.1 | 0.677 |
| 4326546 | 4328633 | LOC_Os01g53920.1 | LOC_Os02g10520.1 | 0.605 |
| 4326546 | 4329422 | LOC_Os01g53920.1 | LOC_Os02g29960.1 | 0.495 |
| 4326546 | 4330003 | LOC_Os01g53920.1 | LOC_Os02g40664.1 | 0.421 |
| 4326546 | 4331144 | LOC_Os01g53920.1 | LOC_Os02g57190.1 | 0.457 |
| 4326546 | 4331168 | LOC_Os01g53920.1 | LOC_Os02g57470.1 | 0.691 |
| 4326546 | 4332080 | LOC_Os01g53920.1 | LOC_Os03g11910.1 | 0.776 |
| 4326546 | 4333771 | LOC_Os01g53920.1 | LOC_Os03g48320.1 | 0.404 |
| 4326546 | 4334214 | LOC_Os01g53920.1 | LOC_Os03g55560.1 | 0.927 |
| 4326546 | 4334935 | LOC_Os01g53920.1 | LOC_Os04g02110.1 | 0.404 |
| 4326546 | 4335058 | LOC_Os01g53920.1 | LOC_Os04g08740.1 | 0.659 |
| 4326546 | 4335089 | LOC_Os01g53920.1 | LOC_Os04g09860.1 | 0.606 |
| 4326546 | 4336120 | LOC_Os01g53920.1 | LOC_Os04g39460.1 | 0.404 |
| 4326546 | 4337257 | LOC_Os01g53920.1 | LOC_Os04g56160.1 | 0.588 |
| 4326546 | 4337619 | LOC_Os01g53920.1 | LOC_Os05g02500.1 | 0.817 |
| 4326546 | 4337658 | LOC_Os01g53920.1 | LOC_Os05g03100.1 | 0.655 |
| 4326546 | 4337686 | LOC_Os01g53920.1 | LOC_Os05g03610.1 | 0.726 |
| 4326546 | 4338096 | LOC_Os01g53920.1 | LOC_Os05g11990.1 | 0.968 |
| 4326546 | 4338481 | LOC_Os01g53920.1 | LOC_Os05g27880.1 | 0.661 |
| 4326546 | 4338531 | LOC_Os01g53920.1 | LOC_Os05g29030.1 | 0.963 |
| 4326546 | 4339763 | LOC_Os01g53920.1 | LOC_Os05g50970.1 | 0.963 |
| 4326546 | 4340110 | LOC_Os01g53920.1 | LOC_Os06g05359.1 | 0.404 |
| 4326546 | 4340753 | LOC_Os01g53920.1 | LOC_Os06g16450.1 | 0.404 |
| 4326546 | 4340847 | LOC_Os01g53920.1 | LOC_Os06g20340.1 | 0.817 |
| 4326546 | 4340915 | LOC_Os01g53920.1 | LOC_Os06g22340.1 | 0.495 |
| 4326546 | 4340964 | LOC_Os01g53920.1 | LOC_Os06g23530.1 | 0.446 |
| 4326546 | 4341219 | LOC_Os01g53920.1 | LOC_Os06g34690.1 | 0.641 |
| 4326546 | 4341646 | LOC_Os01g53920.1 | LOC_Os06g43670.1 | 0.404 |
| 4326546 | 4341853 | LOC_Os01g53920.1 | LOC_Os06g46600.1 | 0.776 |
| 4326546 | 4341965 | LOC_Os01g53920.1 | LOC_Os06g48720.1 | 0.551 |
| 4326546 | 4342017 | LOC_Os01g53920.1 | LOC_Os06g49430.1 | 0.765 |
| 4326546 | 4343299 | LOC_Os01g53920.1 | LOC_Os07g31450.1 | 0.762 |
| 4326546 | 4343339 | LOC_Os01g53920.1 | LOC_Os07g32430.1 | 0.446 |
| 4326546 | 4343395 | LOC_Os01g53920.1 | LOC_Os07g33480.1 | 0.495 |
| 4326546 | 4343409 | LOC_Os01g53920.1 | LOC_Os07g33730.1 | 0.404 |
| 4326546 | 4344698 | LOC_Os01g53920.1 | LOC_Os08g06060.1 | 0.846 |
| 4326546 | 4345065 | LOC_Os01g53920.1 | LOC_Os08g14990.1 | 0.956 |
| 4326546 | 4345202 | LOC_Os01g53920.1 | LOC_Os08g20000.1 | 0.404 |
| 4326546 | 4345297 | LOC_Os01g53920.1 | LOC_Os08g24380.1 | 0.404 |
| 4326546 | 4345304 | LOC_Os01g53920.1 | LOC_Os08g24760.1 | 0.446 |
| 4326546 | 4345628 | LOC_Os01g53920.1 | LOC_Os08g33200.1 | 0.985 |
| 4326546 | 4345708 | LOC_Os01g53920.1 | LOC_Os08g34650.1 | 0.953 |
| 4326546 | 4345910 | LOC_Os01g53920.1 | LOC_Os08g38410.1 | 0.628 |
| 4326546 | 4346276 | LOC_Os01g53920.1 | LOC_Os08g44050.1 | 0.703 |
| 4326546 | 4346315 | LOC_Os01g53920.1 | LOC_Os08g44510.1 | 0.709 |
| 4326546 | 4347005 | LOC_Os01g53920.1 | LOC_Os09g23740.1 | 0.985 |
| 4326546 | 4347095 | LOC_Os01g53920.1 | LOC_Os09g25540.1 | 0.956 |
| 4326546 | 4347787 | LOC_Os01g53920.1 | LOC_Os09g37860.1 | 0.537 |
| 4326546 | 4348731 | LOC_Os01g53920.1 | LOC_Os10g30580.1 | 0.584 |
| 4326546 | 4348805 | LOC_Os01g53920.1 | LOC_Os10g31970.1 | 0.762 |
| 4326546 | 4348853 | LOC_Os01g53920.1 | LOC_Os10g32980.1 | 0.434 |
| 4326546 | 4349742 | LOC_Os01g53920.1 | LOC_Os11g04600.1 | 0.838 |
| 4326546 | 4350109 | LOC_Os01g53920.1 | LOC_Os11g11770.1 | 0.404 |
| 4326546 | 4350130 | LOC_Os01g53920.1 | LOC_Os11g12300.1 | 0.404 |
| 4326546 | 4350472 | LOC_Os01g53920.1 | LOC_Os11g27264.1 | 0.614 |
| 4326546 | 4350473 | LOC_Os01g53920.1 | LOC_Os11g27329.1 | 0.614 |
| 4326546 | 4350814 | LOC_Os01g53920.1 | LOC_Os11g37860.1 | 0.404 |
| 4326546 | 4350954 | LOC_Os01g53920.1 | LOC_Os11g40780.1 | 0.404 |
| 4326546 | 4350996 | LOC_Os01g53920.1 | LOC_Os11g42040.1 | 0.404 |
| 4326546 | 4351038 | LOC_Os01g53920.1 | LOC_Os11g43250.1 | 0.404 |
| 4326546 | 4351041 | LOC_Os01g53920.1 | LOC_Os11g43390.1 | 0.404 |
| 4326546 | 4351431 | LOC_Os01g53920.1 | LOC_Os12g03990.1 | 0.817 |
| 4326546 | 4351457 | LOC_Os01g53920.1 | LOC_Os12g04410.1 | 0.838 |
| 4326546 | 4351749 | LOC_Os01g53920.1 | LOC_Os12g10410.1 | 0.404 |
| 4326546 | 4352130 | LOC_Os01g53920.1 | LOC_Os12g25170.1 | 0.404 |
| 4326546 | 4352270 | LOC_Os01g53920.1 | LOC_Os12g31620.1 | 0.404 |
| 4326546 | LOC_Os02g49270.1 | LOC_Os01g53920.1 | LOC_Os02g49270.1 | 0.537 |
| 4326830 | 4349306 | LOC_Os01g70790.1 | LOC_Os10g40140.1 | 0.445 |
| 4327022 | 4337658 | LOC_Os01g55610.1 | LOC_Os05g03100.1 | 0.495 |
| 4327051 | 4337435 | LOC_Os01g57720.1 | LOC_Os04g58560.1 | 0.691 |
| 4327384 | 4324481 | LOC_Os01g62230.1 | LOC_Os03g17100.1 | 0.986 |
| 4327384 | 4324495 | LOC_Os01g62230.1 | LOC_Os01g05900.1 | 0.54 |
| 4327384 | 4324500 | LOC_Os01g62230.1 | LOC_Os01g05630.1 | 0.617 |
| 4327384 | 4326270 | LOC_Os01g62230.1 | LOC_Os01g16414.1 | 0.473 |
| 4327384 | 4341655 | LOC_Os01g62230.1 | LOC_Os06g43790.1 | 0.683 |
| 4327384 | 4342472 | LOC_Os01g62230.1 | LOC_Os07g06980.1 | 0.774 |
| 4327384 | 4343299 | LOC_Os01g62230.1 | LOC_Os07g31450.1 | 0.463 |
| 4327384 | 4344819 | LOC_Os01g62230.1 | LOC_Os08g08210.1 | 0.492 |
| 4327384 | 4346109 | LOC_Os01g62230.1 | LOC_Os08g41630.1 | 0.53 |
| 4327384 | H2B.9 | LOC_Os01g62230.1 | LOC_Os05g49860.1 | 0.54 |
| 4327389 | ARF15 | LOC_Os01g62300.1 | LOC_Os05g48870.1 | 0.445 |
| 4327667 | 4324511 | LOC_Os01g40590.1 | LOC_Os01g05620.1 | 0.404 |
| 4327667 | 4324660 | LOC_Os01g40590.1 | LOC_Os01g52380.1 | 0.404 |
| 4327667 | 4333771 | LOC_Os01g40590.1 | LOC_Os03g48320.1 | 0.404 |
| 4327667 | 4334935 | LOC_Os01g40590.1 | LOC_Os04g02110.1 | 0.404 |
| 4327667 | 4335058 | LOC_Os01g40590.1 | LOC_Os04g08740.1 | 0.415 |
| 4327667 | 4336120 | LOC_Os01g40590.1 | LOC_Os04g39460.1 | 0.404 |
| 4327667 | 4338096 | LOC_Os01g40590.1 | LOC_Os05g11990.1 | 0.703 |
| 4327667 | 4338531 | LOC_Os01g40590.1 | LOC_Os05g29030.1 | 0.724 |
| 4327667 | 4339763 | LOC_Os01g40590.1 | LOC_Os05g50970.1 | 0.724 |
| 4327667 | 4340110 | LOC_Os01g40590.1 | LOC_Os06g05359.1 | 0.404 |
| 4327667 | 4340753 | LOC_Os01g40590.1 | LOC_Os06g16450.1 | 0.404 |
| 4327667 | 4341646 | LOC_Os01g40590.1 | LOC_Os06g43670.1 | 0.404 |
| 4327667 | 4342017 | LOC_Os01g40590.1 | LOC_Os06g49430.1 | 0.468 |
| 4327667 | 4343409 | LOC_Os01g40590.1 | LOC_Os07g33730.1 | 0.404 |
| 4327667 | 4344698 | LOC_Os01g40590.1 | LOC_Os08g06060.1 | 0.468 |
| 4327667 | 4345202 | LOC_Os01g40590.1 | LOC_Os08g20000.1 | 0.404 |
| 4327667 | 4345297 | LOC_Os01g40590.1 | LOC_Os08g24380.1 | 0.404 |
| 4327667 | 4345628 | LOC_Os01g40590.1 | LOC_Os08g33200.1 | 0.562 |
| 4327667 | 4347005 | LOC_Os01g40590.1 | LOC_Os09g23740.1 | 0.562 |
| 4327667 | 4347787 | LOC_Os01g40590.1 | LOC_Os09g37860.1 | 0.408 |
| 4327667 | 4349742 | LOC_Os01g40590.1 | LOC_Os11g04600.1 | 0.423 |
| 4327667 | 4350109 | LOC_Os01g40590.1 | LOC_Os11g11770.1 | 0.404 |
| 4327667 | 4350130 | LOC_Os01g40590.1 | LOC_Os11g12300.1 | 0.404 |
| 4327667 | 4350814 | LOC_Os01g40590.1 | LOC_Os11g37860.1 | 0.404 |
| 4327667 | 4350954 | LOC_Os01g40590.1 | LOC_Os11g40780.1 | 0.404 |
| 4327667 | 4350996 | LOC_Os01g40590.1 | LOC_Os11g42040.1 | 0.404 |
| 4327667 | 4351038 | LOC_Os01g40590.1 | LOC_Os11g43250.1 | 0.404 |
| 4327667 | 4351041 | LOC_Os01g40590.1 | LOC_Os11g43390.1 | 0.404 |
| 4327667 | 4351457 | LOC_Os01g40590.1 | LOC_Os12g04410.1 | 0.423 |
| 4327667 | 4351749 | LOC_Os01g40590.1 | LOC_Os12g10410.1 | 0.404 |
| 4327667 | 4352130 | LOC_Os01g40590.1 | LOC_Os12g25170.1 | 0.404 |
| 4327667 | 4352270 | LOC_Os01g40590.1 | LOC_Os12g31620.1 | 0.404 |
| 4327667 | IRL7 | LOC_Os01g40590.1 | LOC_Os03g11360.2 | 0.404 |
| 4327667 | LOC_Os02g49270.1 | LOC_Os01g40590.1 | LOC_Os02g49270.1 | 0.408 |
| 4327790 | 4324495 | LOC_Os01g13570.1 | LOC_Os01g05900.1 | 0.678 |
| 4327790 | 4326439 | LOC_Os01g13570.1 | LOC_Os01g01302.1 | 0.404 |
| 4327790 | 4334290 | LOC_Os01g13570.1 | LOC_Os03g56460.1 | 0.616 |
| 4327790 | 4338096 | LOC_Os01g13570.1 | LOC_Os05g11990.1 | 0.509 |
| 4327790 | 4341683 | LOC_Os01g13570.1 | LOC_Os06g44060.1 | 0.654 |
| 4327790 | 4344267 | LOC_Os01g13570.1 | LOC_Os07g47820.1 | 0.42 |
| 4327790 | 4346699 | LOC_Os01g13570.1 | LOC_Os09g14670.1 | 0.445 |
| 4327790 | 4347311 | LOC_Os01g13570.1 | LOC_Os09g29070.1 | 0.616 |
| 4327967 | 4327983 | LOC_Os01g58780.1 | LOC_Os02g01170.1 | 0.908 |
| 4327967 | 4337586 | LOC_Os01g58780.1 | LOC_Os05g01940.1 | 0.9 |
| 4327967 | 4344172 | LOC_Os01g58780.1 | LOC_Os07g46560.1 | 0.902 |
| 4327983 | 4326546 | LOC_Os02g01170.1 | LOC_Os01g53920.1 | 0.655 |
| 4327983 | 4327967 | LOC_Os02g01170.1 | LOC_Os01g58780.1 | 0.908 |
| 4327983 | 4337586 | LOC_Os02g01170.1 | LOC_Os05g01940.1 | 0.908 |
| 4327983 | 4337658 | LOC_Os02g01170.1 | LOC_Os05g03100.1 | 0.422 |
| 4327983 | 4340849 | LOC_Os02g01170.1 | LOC_Os06g20370.1 | 0.657 |
| 4327983 | 4344172 | LOC_Os02g01170.1 | LOC_Os07g46560.1 | 0.9 |
| 4327983 | 4345065 | LOC_Os02g01170.1 | LOC_Os08g14990.1 | 0.655 |
| 4327983 | 4345708 | LOC_Os02g01170.1 | LOC_Os08g34650.1 | 0.655 |
| 4327983 | 4347095 | LOC_Os02g01170.1 | LOC_Os09g25540.1 | 0.655 |
| 4327983 | 4351436 | LOC_Os02g01170.1 | LOC_Os12g04030.1 | 0.403 |
| 4328135 | 4324584 | LOC_Os02g03060.1 | LOC_Os01g11920.1 | 0.553 |
| 4328135 | 4324980 | LOC_Os02g03060.1 | LOC_Os01g67970.1 | 0.659 |
| 4328135 | 4326151 | LOC_Os02g03060.1 | LOC_Os01g01689.1 | 0.471 |
| 4328135 | 4326270 | LOC_Os02g03060.1 | LOC_Os01g16414.1 | 0.466 |
| 4328135 | 4326546 | LOC_Os02g03060.1 | LOC_Os01g53920.1 | 0.677 |
| 4328135 | 4328287 | LOC_Os02g03060.1 | LOC_Os02g05340.1 | 0.991 |
| 4328135 | 4330866 | LOC_Os02g03060.1 | LOC_Os02g53120.1 | 0.528 |
| 4328135 | 4330902 | LOC_Os02g03060.1 | LOC_Os02g53680.1 | 0.444 |
| 4328135 | 4331168 | LOC_Os02g03060.1 | LOC_Os02g57470.1 | 0.405 |
| 4328135 | 4331991 | LOC_Os02g03060.1 | LOC_Os03g10780.1 | 0.472 |
| 4328135 | 4332190 | LOC_Os02g03060.1 | LOC_Os03g13460.1 | 0.717 |
| 4328135 | 4337619 | LOC_Os02g03060.1 | LOC_Os05g02500.1 | 0.496 |
| 4328135 | 4338096 | LOC_Os02g03060.1 | LOC_Os05g11990.1 | 0.598 |
| 4328135 | 4338689 | LOC_Os02g03060.1 | LOC_Os05g32600.1 | 0.934 |
| 4328135 | 4339099 | LOC_Os02g03060.1 | LOC_Os05g39850.1 | 0.964 |
| 4328135 | 4340847 | LOC_Os02g03060.1 | LOC_Os06g20340.1 | 0.496 |
| 4328135 | 4340849 | LOC_Os02g03060.1 | LOC_Os06g20370.1 | 0.744 |
| 4328135 | 4341510 | LOC_Os02g03060.1 | LOC_Os06g40840.1 | 0.717 |
| 4328135 | 4342017 | LOC_Os02g03060.1 | LOC_Os06g49430.1 | 0.594 |
| 4328135 | 4342410 | LOC_Os02g03060.1 | LOC_Os07g05620.1 | 0.442 |
| 4328135 | 4344698 | LOC_Os02g03060.1 | LOC_Os08g06060.1 | 0.619 |
| 4328135 | 4345065 | LOC_Os02g03060.1 | LOC_Os08g14990.1 | 0.675 |
| 4328135 | 4345502 | LOC_Os02g03060.1 | LOC_Os08g30820.1 | 0.449 |
| 4328135 | 4345708 | LOC_Os02g03060.1 | LOC_Os08g34650.1 | 0.671 |
| 4328135 | 4346208 | LOC_Os02g03060.1 | LOC_Os08g43090.1 | 0.434 |
| 4328135 | 4346276 | LOC_Os02g03060.1 | LOC_Os08g44050.1 | 0.612 |
| 4328135 | 4347095 | LOC_Os02g03060.1 | LOC_Os09g25540.1 | 0.673 |
| 4328135 | 4347221 | LOC_Os02g03060.1 | LOC_Os09g27700.1 | 0.717 |
| 4328135 | 4349742 | LOC_Os02g03060.1 | LOC_Os11g04600.1 | 0.678 |
| 4328135 | 4351431 | LOC_Os02g03060.1 | LOC_Os12g03990.1 | 0.496 |
| 4328135 | 4351457 | LOC_Os02g03060.1 | LOC_Os12g04410.1 | 0.678 |
| 4328287 | 4328135 | LOC_Os02g05340.1 | LOC_Os02g03060.1 | 0.991 |
| 4328287 | 4328632 | LOC_Os02g05340.1 | LOC_Os02g10510.1 | 0.726 |
| 4328287 | 4330868 | LOC_Os02g05340.1 | LOC_Os02g53140.1 | 0.9 |
| 4328287 | 4337063 | LOC_Os02g05340.1 | LOC_Os04g53410.1 | 0.903 |
| 4328287 | 4348731 | LOC_Os02g05340.1 | LOC_Os10g30580.1 | 0.748 |
| 4328287 | 4349719 | LOC_Os02g05340.1 | LOC_Os11g04220.1 | 0.792 |
| 4328287 | 4351436 | LOC_Os02g05340.1 | LOC_Os12g04030.1 | 0.792 |
| 4328300 | 4325329 | LOC_Os02g05510.1 | LOC_Os01g08700.1 | 0.421 |
| 4328300 | 4342017 | LOC_Os02g05510.1 | LOC_Os06g49430.1 | 0.414 |
| 4328300 | 4344698 | LOC_Os02g05510.1 | LOC_Os08g06060.1 | 0.414 |
| 4328305 | 4326027 | LOC_Os02g05620.1 | LOC_Os01g25820.1 | 0.416 |
| 4328305 | 4333493 | LOC_Os02g05620.1 | LOC_Os03g42750.1 | 0.857 |
| 4328632 | 4328287 | LOC_Os02g10510.1 | LOC_Os02g05340.1 | 0.726 |
| 4328632 | 4337658 | LOC_Os02g10510.1 | LOC_Os05g03100.1 | 0.431 |
| 4328633 | 4326546 | LOC_Os02g10520.1 | LOC_Os01g53920.1 | 0.605 |
| 4328633 | 4338096 | LOC_Os02g10520.1 | LOC_Os05g11990.1 | 0.447 |
| 4328633 | 4345065 | LOC_Os02g10520.1 | LOC_Os08g14990.1 | 0.605 |
| 4328633 | 4345708 | LOC_Os02g10520.1 | LOC_Os08g34650.1 | 0.605 |
| 4328633 | 4347095 | LOC_Os02g10520.1 | LOC_Os09g25540.1 | 0.605 |
| 4328702 | 4338096 | LOC_Os02g11960.1 | LOC_Os05g11990.1 | 0.477 |
| 4328702 | 4341281 | LOC_Os02g11960.1 | LOC_Os06g36090.1 | 0.643 |
| 4328725 | 4324511 | LOC_Os02g12440.1 | LOC_Os01g05620.1 | 0.404 |
| 4328725 | 4324660 | LOC_Os02g12440.1 | LOC_Os01g52380.1 | 0.404 |
| 4328725 | 4333771 | LOC_Os02g12440.1 | LOC_Os03g48320.1 | 0.404 |
| 4328725 | 4334935 | LOC_Os02g12440.1 | LOC_Os04g02110.1 | 0.404 |
| 4328725 | 4335058 | LOC_Os02g12440.1 | LOC_Os04g08740.1 | 0.415 |
| 4328725 | 4336120 | LOC_Os02g12440.1 | LOC_Os04g39460.1 | 0.404 |
| 4328725 | 4338096 | LOC_Os02g12440.1 | LOC_Os05g11990.1 | 0.703 |
| 4328725 | 4338531 | LOC_Os02g12440.1 | LOC_Os05g29030.1 | 0.703 |
| 4328725 | 4339763 | LOC_Os02g12440.1 | LOC_Os05g50970.1 | 0.703 |
| 4328725 | 4340110 | LOC_Os02g12440.1 | LOC_Os06g05359.1 | 0.404 |
| 4328725 | 4340753 | LOC_Os02g12440.1 | LOC_Os06g16450.1 | 0.404 |
| 4328725 | 4341646 | LOC_Os02g12440.1 | LOC_Os06g43670.1 | 0.404 |
| 4328725 | 4342017 | LOC_Os02g12440.1 | LOC_Os06g49430.1 | 0.469 |
| 4328725 | 4343409 | LOC_Os02g12440.1 | LOC_Os07g33730.1 | 0.404 |
| 4328725 | 4344698 | LOC_Os02g12440.1 | LOC_Os08g06060.1 | 0.492 |
| 4328725 | 4345202 | LOC_Os02g12440.1 | LOC_Os08g20000.1 | 0.404 |
| 4328725 | 4345297 | LOC_Os02g12440.1 | LOC_Os08g24380.1 | 0.404 |
| 4328725 | 4345628 | LOC_Os02g12440.1 | LOC_Os08g33200.1 | 0.562 |
| 4328725 | 4347005 | LOC_Os02g12440.1 | LOC_Os09g23740.1 | 0.562 |
| 4328725 | 4347787 | LOC_Os02g12440.1 | LOC_Os09g37860.1 | 0.408 |
| 4328725 | 4350109 | LOC_Os02g12440.1 | LOC_Os11g11770.1 | 0.404 |
| 4328725 | 4350130 | LOC_Os02g12440.1 | LOC_Os11g12300.1 | 0.404 |
| 4328725 | 4350814 | LOC_Os02g12440.1 | LOC_Os11g37860.1 | 0.404 |
| 4328725 | 4350954 | LOC_Os02g12440.1 | LOC_Os11g40780.1 | 0.404 |
| 4328725 | 4350996 | LOC_Os02g12440.1 | LOC_Os11g42040.1 | 0.404 |
| 4328725 | 4351038 | LOC_Os02g12440.1 | LOC_Os11g43250.1 | 0.404 |
| 4328725 | 4351041 | LOC_Os02g12440.1 | LOC_Os11g43390.1 | 0.404 |
| 4328725 | 4351749 | LOC_Os02g12440.1 | LOC_Os12g10410.1 | 0.404 |
| 4328725 | 4352130 | LOC_Os02g12440.1 | LOC_Os12g25170.1 | 0.404 |
| 4328725 | 4352270 | LOC_Os02g12440.1 | LOC_Os12g31620.1 | 0.404 |
| 4328725 | LOC_Os02g49270.1 | LOC_Os02g12440.1 | LOC_Os02g49270.1 | 0.408 |
| 4328745 | BGLU2 | LOC_Os02g12730.1 | LOC_Os01g59819.1 | 0.468 |
| 4328828 | 4334290 | LOC_Os02g14110.1 | LOC_Os03g56460.1 | 0.566 |
| 4328828 | 4335673 | LOC_Os02g14110.1 | LOC_Os04g32020.1 | 0.924 |
| 4328828 | 4346699 | LOC_Os02g14110.1 | LOC_Os09g14670.1 | 0.441 |
| 4328828 | 4347172 | LOC_Os02g14110.1 | LOC_Os09g26880.1 | 0.438 |
| 4328828 | 4347311 | LOC_Os02g14110.1 | LOC_Os09g29070.1 | 0.566 |
| 4328828 | 4348561 | LOC_Os02g14110.1 | LOC_Os10g26010.1 | 0.428 |
| 4328828 | 4351017 | LOC_Os02g14110.1 | LOC_Os11g42510.1 | 0.689 |
| 4328828 | 4351847 | LOC_Os02g14110.1 | LOC_Os12g13320.1 | 0.936 |
| 4329037 | 4326439 | LOC_Os02g18450.1 | LOC_Os01g01302.1 | 0.722 |
| 4329037 | 4345365 | LOC_Os02g18450.1 | LOC_Os08g27010.1 | 0.723 |
| 4329037 | 4346508 | LOC_Os02g18450.1 | LOC_Os09g07570.1 | 0.592 |
| 4329037 | 4350398 | LOC_Os02g18450.1 | LOC_Os11g24450.1 | 0.741 |
| 4329416 | 4332258 | LOC_Os02g29530.1 | LOC_Os03g14540.1 | 0.724 |
| 4329416 | 4344268 | LOC_Os02g29530.1 | LOC_Os07g47830.1 | 0.922 |
| 4329422 | 4326546 | LOC_Os02g29960.1 | LOC_Os01g53920.1 | 0.495 |
| 4329422 | 4340373 | LOC_Os02g29960.1 | LOC_Os06g09240.1 | 0.436 |
| 4329422 | 4343183 | LOC_Os02g29960.1 | LOC_Os07g28480.1 | 0.412 |
| 4329422 | 4343352 | LOC_Os02g29960.1 | LOC_Os07g32620.1 | 0.436 |
| 4329422 | 4343395 | LOC_Os02g29960.1 | LOC_Os07g33480.1 | 0.494 |
| 4329422 | 4345065 | LOC_Os02g29960.1 | LOC_Os08g14990.1 | 0.495 |
| 4329422 | 4345708 | LOC_Os02g29960.1 | LOC_Os08g34650.1 | 0.495 |
| 4329422 | 4347095 | LOC_Os02g29960.1 | LOC_Os09g25540.1 | 0.495 |
| 4329422 | 4347809 | LOC_Os02g29960.1 | LOC_Os09g38239.1 | 0.403 |
| 4329438 | 4337164 | LOC_Os02g30310.1 | LOC_Os04g54790.1 | 0.401 |
| 4329438 | 4338096 | LOC_Os02g30310.1 | LOC_Os05g11990.1 | 0.466 |
| 4329448 | 4324584 | LOC_Os02g30620.1 | LOC_Os01g11920.1 | 0.513 |
| 4329448 | 4331168 | LOC_Os02g30620.1 | LOC_Os02g57470.1 | 0.513 |
| 4329448 | 4332080 | LOC_Os02g30620.1 | LOC_Os03g11910.1 | 0.879 |
| 4329448 | 4338096 | LOC_Os02g30620.1 | LOC_Os05g11990.1 | 0.972 |
| 4329448 | 4341219 | LOC_Os02g30620.1 | LOC_Os06g34690.1 | 0.575 |
| 4329448 | 4341853 | LOC_Os02g30620.1 | LOC_Os06g46600.1 | 0.879 |
| 4329448 | 4345628 | LOC_Os02g30620.1 | LOC_Os08g33200.1 | 0.636 |
| 4329448 | 4347005 | LOC_Os02g30620.1 | LOC_Os09g23740.1 | 0.636 |
| 4329677 | 4325329 | LOC_Os02g35180.1 | LOC_Os01g08700.1 | 0.421 |
| 4329677 | 4342017 | LOC_Os02g35180.1 | LOC_Os06g49430.1 | 0.414 |
| 4329677 | 4344698 | LOC_Os02g35180.1 | LOC_Os08g06060.1 | 0.414 |
| 4330003 | 4326546 | LOC_Os02g40664.1 | LOC_Os01g53920.1 | 0.421 |
| 4330003 | 4345065 | LOC_Os02g40664.1 | LOC_Os08g14990.1 | 0.421 |
| 4330003 | 4345708 | LOC_Os02g40664.1 | LOC_Os08g34650.1 | 0.421 |
| 4330003 | 4347095 | LOC_Os02g40664.1 | LOC_Os09g25540.1 | 0.421 |
| 4330159 | 4332080 | LOC_Os02g43350.1 | LOC_Os03g11910.1 | 0.401 |
| 4330159 | 4337164 | LOC_Os02g43350.1 | LOC_Os04g54790.1 | 0.567 |
| 4330159 | 4345628 | LOC_Os02g43350.1 | LOC_Os08g33200.1 | 0.408 |
| 4330297 | 4330484 | LOC_Os02g45250.1 | LOC_Os02g47970.1 | 0.716 |
| 4330484 | 4324980 | LOC_Os02g47970.1 | LOC_Os01g67970.1 | 0.471 |
| 4330484 | 4330297 | LOC_Os02g47970.1 | LOC_Os02g45250.1 | 0.716 |
| 4330484 | 4346276 | LOC_Os02g47970.1 | LOC_Os08g44050.1 | 0.459 |
| 4330701 | 4324511 | LOC_Os02g50970.1 | LOC_Os01g05620.1 | 0.404 |
| 4330701 | 4324660 | LOC_Os02g50970.1 | LOC_Os01g52380.1 | 0.404 |
| 4330701 | 4333771 | LOC_Os02g50970.1 | LOC_Os03g48320.1 | 0.404 |
| 4330701 | 4334214 | LOC_Os02g50970.1 | LOC_Os03g55560.1 | 0.465 |
| 4330701 | 4334935 | LOC_Os02g50970.1 | LOC_Os04g02110.1 | 0.404 |
| 4330701 | 4335058 | LOC_Os02g50970.1 | LOC_Os04g08740.1 | 0.864 |
| 4330701 | 4336120 | LOC_Os02g50970.1 | LOC_Os04g39460.1 | 0.404 |
| 4330701 | 4338096 | LOC_Os02g50970.1 | LOC_Os05g11990.1 | 0.703 |
| 4330701 | 4338531 | LOC_Os02g50970.1 | LOC_Os05g29030.1 | 0.78 |
| 4330701 | 4339763 | LOC_Os02g50970.1 | LOC_Os05g50970.1 | 0.78 |
| 4330701 | 4340110 | LOC_Os02g50970.1 | LOC_Os06g05359.1 | 0.404 |
| 4330701 | 4340753 | LOC_Os02g50970.1 | LOC_Os06g16450.1 | 0.404 |
| 4330701 | 4341646 | LOC_Os02g50970.1 | LOC_Os06g43670.1 | 0.404 |
| 4330701 | 4342017 | LOC_Os02g50970.1 | LOC_Os06g49430.1 | 0.538 |
| 4330701 | 4343409 | LOC_Os02g50970.1 | LOC_Os07g33730.1 | 0.404 |
| 4330701 | 4344698 | LOC_Os02g50970.1 | LOC_Os08g06060.1 | 0.514 |
| 4330701 | 4345202 | LOC_Os02g50970.1 | LOC_Os08g20000.1 | 0.404 |
| 4330701 | 4345297 | LOC_Os02g50970.1 | LOC_Os08g24380.1 | 0.404 |
| 4330701 | 4345628 | LOC_Os02g50970.1 | LOC_Os08g33200.1 | 0.562 |
| 4330701 | 4346315 | LOC_Os02g50970.1 | LOC_Os08g44510.1 | 0.406 |
| 4330701 | 4347005 | LOC_Os02g50970.1 | LOC_Os09g23740.1 | 0.562 |
| 4330701 | 4347787 | LOC_Os02g50970.1 | LOC_Os09g37860.1 | 0.408 |
| 4330701 | 4350109 | LOC_Os02g50970.1 | LOC_Os11g11770.1 | 0.404 |
| 4330701 | 4350130 | LOC_Os02g50970.1 | LOC_Os11g12300.1 | 0.404 |
| 4330701 | 4350814 | LOC_Os02g50970.1 | LOC_Os11g37860.1 | 0.404 |
| 4330701 | 4350954 | LOC_Os02g50970.1 | LOC_Os11g40780.1 | 0.404 |
| 4330701 | 4350996 | LOC_Os02g50970.1 | LOC_Os11g42040.1 | 0.404 |
| 4330701 | 4351038 | LOC_Os02g50970.1 | LOC_Os11g43250.1 | 0.404 |
| 4330701 | 4351041 | LOC_Os02g50970.1 | LOC_Os11g43390.1 | 0.404 |
| 4330701 | 4351749 | LOC_Os02g50970.1 | LOC_Os12g10410.1 | 0.404 |
| 4330701 | 4352130 | LOC_Os02g50970.1 | LOC_Os12g25170.1 | 0.404 |
| 4330701 | 4352270 | LOC_Os02g50970.1 | LOC_Os12g31620.1 | 0.404 |
| 4330701 | IRL7 | LOC_Os02g50970.1 | LOC_Os03g11360.2 | 0.404 |
| 4330701 | LOC_Os02g49270.1 | LOC_Os02g50970.1 | LOC_Os02g49270.1 | 0.408 |
| 4330709 | 4334290 | LOC_Os02g51070.1 | LOC_Os03g56460.1 | 0.549 |
| 4330709 | 4347311 | LOC_Os02g51070.1 | LOC_Os09g29070.1 | 0.549 |
| 4330744 | 4324584 | LOC_Os02g51540.1 | LOC_Os01g11920.1 | 0.531 |
| 4330744 | 4326270 | LOC_Os02g51540.1 | LOC_Os01g16414.1 | 0.4 |
| 4330744 | 4350472 | LOC_Os02g51540.1 | LOC_Os11g27264.1 | 0.68 |
| 4330744 | 4350473 | LOC_Os02g51540.1 | LOC_Os11g27329.1 | 0.68 |
| 4330796 | 4333814 | LOC_Os02g52250.1 | LOC_Os03g49210.1 | 0.635 |
| 4330796 | 4340964 | LOC_Os02g52250.1 | LOC_Os06g23530.1 | 0.73 |
| 4330796 | 4342472 | LOC_Os02g52250.1 | LOC_Os07g06980.1 | 0.604 |
| 4330796 | 4343339 | LOC_Os02g52250.1 | LOC_Os07g32430.1 | 0.97 |
| 4330796 | 4345304 | LOC_Os02g52250.1 | LOC_Os08g24760.1 | 0.895 |
| 4330796 | 4345502 | LOC_Os02g52250.1 | LOC_Os08g30820.1 | 0.9 |
| 4330796 | 4345910 | LOC_Os02g52250.1 | LOC_Os08g38410.1 | 0.664 |
| 4330866 | 4328135 | LOC_Os02g53120.1 | LOC_Os02g03060.1 | 0.528 |
| 4330866 | 4332190 | LOC_Os02g53120.1 | LOC_Os03g13460.1 | 0.687 |
| 4330866 | 4334214 | LOC_Os02g53120.1 | LOC_Os03g55560.1 | 0.43 |
| 4330866 | 4337619 | LOC_Os02g53120.1 | LOC_Os05g02500.1 | 0.422 |
| 4330866 | 4337886 | LOC_Os02g53120.1 | LOC_Os05g06840.1 | 0.405 |
| 4330866 | 4339099 | LOC_Os02g53120.1 | LOC_Os05g39850.1 | 0.72 |
| 4330866 | 4340847 | LOC_Os02g53120.1 | LOC_Os06g20340.1 | 0.422 |
| 4330866 | 4340849 | LOC_Os02g53120.1 | LOC_Os06g20370.1 | 0.705 |
| 4330866 | 4341510 | LOC_Os02g53120.1 | LOC_Os06g40840.1 | 0.687 |
| 4330866 | 4343388 | LOC_Os02g53120.1 | LOC_Os07g33370.1 | 0.444 |
| 4330866 | 4347221 | LOC_Os02g53120.1 | LOC_Os09g27700.1 | 0.687 |
| 4330866 | 4348731 | LOC_Os02g53120.1 | LOC_Os10g30580.1 | 0.629 |
| 4330866 | 4351431 | LOC_Os02g53120.1 | LOC_Os12g03990.1 | 0.422 |
| 4330868 | 4325329 | LOC_Os02g53140.1 | LOC_Os01g08700.1 | 0.964 |
| 4330868 | 4328287 | LOC_Os02g53140.1 | LOC_Os02g05340.1 | 0.9 |
| 4330868 | 4334135 | LOC_Os02g53140.1 | LOC_Os03g54084.1 | 0.501 |
| 4330868 | 4347677 | LOC_Os02g53140.1 | LOC_Os09g36240.1 | 0.582 |
| 4330868 | 4349531 | LOC_Os02g53140.1 | LOC_Os11g01140.1 | 0.514 |
| 4330868 | 4349939 | LOC_Os02g53140.1 | LOC_Os11g07910.1 | 0.595 |
| 4330902 | 4326151 | LOC_Os02g53680.1 | LOC_Os01g01689.1 | 0.764 |
| 4330902 | 4328135 | LOC_Os02g53680.1 | LOC_Os02g03060.1 | 0.444 |
| 4330902 | 4331991 | LOC_Os02g53680.1 | LOC_Os03g10780.1 | 0.571 |
| 4330902 | 4337886 | LOC_Os02g53680.1 | LOC_Os05g06840.1 | 0.51 |
| 4330902 | 4339099 | LOC_Os02g53680.1 | LOC_Os05g39850.1 | 0.748 |
| 4330902 | 4343276 | LOC_Os02g53680.1 | LOC_Os07g30980.1 | 0.473 |
| 4330902 | 4344306 | LOC_Os02g53680.1 | LOC_Os07g48360.1 | 0.866 |
| 4330902 | 4351770 | LOC_Os02g53680.1 | LOC_Os12g10670.1 | 0.793 |
| 4330971 | 4324364 | LOC_Os02g54820.1 | LOC_Os01g69030.2 | 0.965 |
| 4330971 | 4332080 | LOC_Os02g54820.1 | LOC_Os03g11910.1 | 0.545 |
| 4330971 | 4334290 | LOC_Os02g54820.1 | LOC_Os03g56460.1 | 0.53 |
| 4330971 | 4335058 | LOC_Os02g54820.1 | LOC_Os04g08740.1 | 0.401 |
| 4330971 | 4336977 | LOC_Os02g54820.1 | LOC_Os04g52370.1 | 0.936 |
| 4330971 | 4341219 | LOC_Os02g54820.1 | LOC_Os06g34690.1 | 0.423 |
| 4330971 | 4341853 | LOC_Os02g54820.1 | LOC_Os06g46600.1 | 0.544 |
| 4330971 | 4342770 | LOC_Os02g54820.1 | LOC_Os07g12640.1 | 0.478 |
| 4330971 | 4345628 | LOC_Os02g54820.1 | LOC_Os08g33200.1 | 0.511 |
| 4330971 | 4347005 | LOC_Os02g54820.1 | LOC_Os09g23740.1 | 0.511 |
| 4330971 | 4347172 | LOC_Os02g54820.1 | LOC_Os09g26880.1 | 0.457 |
| 4330971 | 4347311 | LOC_Os02g54820.1 | LOC_Os09g29070.1 | 0.53 |
| 4331005 | 4332567 | LOC_Os02g55260.1 | LOC_Os03g18840.1 | 0.901 |
| 4331005 | 4333574 | LOC_Os02g55260.1 | LOC_Os03g44530.1 | 0.499 |
| 4331005 | 4333814 | LOC_Os02g55260.1 | LOC_Os03g49210.1 | 0.835 |
| 4331005 | 4336833 | LOC_Os02g55260.1 | LOC_Os04g50660.1 | 0.903 |
| 4331005 | 4340964 | LOC_Os02g55260.1 | LOC_Os06g23530.1 | 0.506 |
| 4331005 | 4342673 | LOC_Os02g55260.1 | LOC_Os07g10350.1 | 0.769 |
| 4331005 | 4347787 | LOC_Os02g55260.1 | LOC_Os09g37860.1 | 0.716 |
| 4331005 | 4350837 | LOC_Os02g55260.1 | LOC_Os11g38170.1 | 0.492 |
| 4331005 | LOC_Os02g49270.1 | LOC_Os02g55260.1 | LOC_Os02g49270.1 | 0.679 |
| 4331019 | 4339983 | LOC_Os02g55440.1 | LOC_Os06g03760.1 | 0.496 |
| 4331134 | 4324511 | LOC_Os02g57080.1 | LOC_Os01g05620.1 | 0.404 |
| 4331134 | 4324660 | LOC_Os02g57080.1 | LOC_Os01g52380.1 | 0.404 |
| 4331134 | 4333771 | LOC_Os02g57080.1 | LOC_Os03g48320.1 | 0.404 |
| 4331134 | 4334935 | LOC_Os02g57080.1 | LOC_Os04g02110.1 | 0.404 |
| 4331134 | 4335058 | LOC_Os02g57080.1 | LOC_Os04g08740.1 | 0.415 |
| 4331134 | 4336120 | LOC_Os02g57080.1 | LOC_Os04g39460.1 | 0.404 |
| 4331134 | 4338096 | LOC_Os02g57080.1 | LOC_Os05g11990.1 | 0.703 |
| 4331134 | 4338531 | LOC_Os02g57080.1 | LOC_Os05g29030.1 | 0.724 |
| 4331134 | 4339763 | LOC_Os02g57080.1 | LOC_Os05g50970.1 | 0.724 |
| 4331134 | 4340110 | LOC_Os02g57080.1 | LOC_Os06g05359.1 | 0.404 |
| 4331134 | 4340753 | LOC_Os02g57080.1 | LOC_Os06g16450.1 | 0.404 |
| 4331134 | 4341646 | LOC_Os02g57080.1 | LOC_Os06g43670.1 | 0.404 |
| 4331134 | 4342017 | LOC_Os02g57080.1 | LOC_Os06g49430.1 | 0.468 |
| 4331134 | 4343409 | LOC_Os02g57080.1 | LOC_Os07g33730.1 | 0.404 |
| 4331134 | 4344698 | LOC_Os02g57080.1 | LOC_Os08g06060.1 | 0.468 |
| 4331134 | 4345202 | LOC_Os02g57080.1 | LOC_Os08g20000.1 | 0.404 |
| 4331134 | 4345297 | LOC_Os02g57080.1 | LOC_Os08g24380.1 | 0.404 |
| 4331134 | 4345628 | LOC_Os02g57080.1 | LOC_Os08g33200.1 | 0.562 |
| 4331134 | 4347005 | LOC_Os02g57080.1 | LOC_Os09g23740.1 | 0.562 |
| 4331134 | 4347787 | LOC_Os02g57080.1 | LOC_Os09g37860.1 | 0.408 |
| 4331134 | 4349742 | LOC_Os02g57080.1 | LOC_Os11g04600.1 | 0.423 |
| 4331134 | 4350109 | LOC_Os02g57080.1 | LOC_Os11g11770.1 | 0.404 |
| 4331134 | 4350130 | LOC_Os02g57080.1 | LOC_Os11g12300.1 | 0.404 |
| 4331134 | 4350814 | LOC_Os02g57080.1 | LOC_Os11g37860.1 | 0.404 |
| 4331134 | 4350954 | LOC_Os02g57080.1 | LOC_Os11g40780.1 | 0.404 |
| 4331134 | 4350996 | LOC_Os02g57080.1 | LOC_Os11g42040.1 | 0.404 |
| 4331134 | 4351038 | LOC_Os02g57080.1 | LOC_Os11g43250.1 | 0.404 |
| 4331134 | 4351041 | LOC_Os02g57080.1 | LOC_Os11g43390.1 | 0.404 |
| 4331134 | 4351457 | LOC_Os02g57080.1 | LOC_Os12g04410.1 | 0.423 |
| 4331134 | 4351749 | LOC_Os02g57080.1 | LOC_Os12g10410.1 | 0.404 |
| 4331134 | 4352130 | LOC_Os02g57080.1 | LOC_Os12g25170.1 | 0.404 |
| 4331134 | 4352270 | LOC_Os02g57080.1 | LOC_Os12g31620.1 | 0.404 |
| 4331134 | IRL7 | LOC_Os02g57080.1 | LOC_Os03g11360.2 | 0.404 |
| 4331134 | LOC_Os02g49270.1 | LOC_Os02g57080.1 | LOC_Os02g49270.1 | 0.408 |
| 4331144 | 4326270 | LOC_Os02g57190.1 | LOC_Os01g16414.1 | 0.803 |
| 4331144 | 4326546 | LOC_Os02g57190.1 | LOC_Os01g53920.1 | 0.457 |
| 4331144 | 4335089 | LOC_Os02g57190.1 | LOC_Os04g09860.1 | 0.727 |
| 4331144 | 4345065 | LOC_Os02g57190.1 | LOC_Os08g14990.1 | 0.457 |
| 4331144 | 4345708 | LOC_Os02g57190.1 | LOC_Os08g34650.1 | 0.457 |
| 4331144 | 4347095 | LOC_Os02g57190.1 | LOC_Os09g25540.1 | 0.457 |
| 4331168 | 4326546 | LOC_Os02g57470.1 | LOC_Os01g53920.1 | 0.691 |
| 4331168 | 4328135 | LOC_Os02g57470.1 | LOC_Os02g03060.1 | 0.405 |
| 4331168 | 4329448 | LOC_Os02g57470.1 | LOC_Os02g30620.1 | 0.513 |
| 4331168 | 4332080 | LOC_Os02g57470.1 | LOC_Os03g11910.1 | 0.404 |
| 4331168 | 4341853 | LOC_Os02g57470.1 | LOC_Os06g46600.1 | 0.404 |
| 4331168 | 4343196 | LOC_Os02g57470.1 | LOC_Os07g28800.1 | 0.513 |
| 4331168 | 4345065 | LOC_Os02g57470.1 | LOC_Os08g14990.1 | 0.691 |
| 4331168 | 4345708 | LOC_Os02g57470.1 | LOC_Os08g34650.1 | 0.691 |
| 4331168 | 4347095 | LOC_Os02g57470.1 | LOC_Os09g25540.1 | 0.691 |
| 4331168 | 4347787 | LOC_Os02g57470.1 | LOC_Os09g37860.1 | 0.402 |
| 4331168 | 4350837 | LOC_Os02g57470.1 | LOC_Os11g38170.1 | 0.467 |
| 4331253 | 4331345 | LOC_Os02g58490.1 | LOC_Os03g01890.1 | 0.401 |
| 4331253 | 4336992 | LOC_Os02g58490.1 | LOC_Os04g52550.1 | 0.917 |
| 4331253 | 4342472 | LOC_Os02g58490.1 | LOC_Os07g06980.1 | 0.449 |
| 4331253 | 4348919 | LOC_Os02g58490.1 | LOC_Os10g33960.1 | 0.401 |
| 4331253 | ARF15 | LOC_Os02g58490.1 | LOC_Os05g48870.1 | 0.423 |
| 4331345 | 4331253 | LOC_Os03g01890.1 | LOC_Os02g58490.1 | 0.401 |
| 4331367 | 4336833 | LOC_Os03g02110.1 | LOC_Os04g50660.1 | 0.535 |
| 4331367 | 4347787 | LOC_Os03g02110.1 | LOC_Os09g37860.1 | 0.491 |
| 4331991 | 4324428 | LOC_Os03g10780.1 | LOC_Os01g53070.1 | 0.55 |
| 4331991 | 4326151 | LOC_Os03g10780.1 | LOC_Os01g01689.1 | 0.712 |
| 4331991 | 4326378 | LOC_Os03g10780.1 | LOC_Os01g40980.1 | 0.558 |
| 4331991 | 4328135 | LOC_Os03g10780.1 | LOC_Os02g03060.1 | 0.472 |
| 4331991 | 4330902 | LOC_Os03g10780.1 | LOC_Os02g53680.1 | 0.571 |
| 4331991 | 4337228 | LOC_Os03g10780.1 | LOC_Os04g55700.1 | 0.427 |
| 4331991 | 4338096 | LOC_Os03g10780.1 | LOC_Os05g11990.1 | 0.451 |
| 4331991 | 4338689 | LOC_Os03g10780.1 | LOC_Os05g32600.1 | 0.617 |
| 4331991 | 4342673 | LOC_Os03g10780.1 | LOC_Os07g10350.1 | 0.439 |
| 4331991 | 4343276 | LOC_Os03g10780.1 | LOC_Os07g30980.1 | 0.691 |
| 4331991 | 4344306 | LOC_Os03g10780.1 | LOC_Os07g48360.1 | 0.792 |
| 4331991 | 4345628 | LOC_Os03g10780.1 | LOC_Os08g33200.1 | 0.4 |
| 4331991 | 4347005 | LOC_Os03g10780.1 | LOC_Os09g23740.1 | 0.4 |
| 4331991 | 4351770 | LOC_Os03g10780.1 | LOC_Os12g10670.1 | 0.551 |
| 4332040 | 4347935 | LOC_Os03g11410.1 | LOC_Os10g01044.1 | 0.565 |
| 4332040 | 4352803 | LOC_Os03g11410.1 | LOC_Os12g42230.1 | 0.718 |
| 4332049 | 4332567 | LOC_Os03g11510.1 | LOC_Os03g18840.1 | 0.825 |
| 4332049 | 4347787 | LOC_Os03g11510.1 | LOC_Os09g37860.1 | 0.483 |
| 4332049 | LOC_Os06g04340.1 | LOC_Os03g11510.1 | LOC_Os06g04340.1 | 0.425 |
| 4332080 | 4324082 | LOC_Os03g11910.1 | LOC_Os01g06320.1 | 0.683 |
| 4332080 | 4324584 | LOC_Os03g11910.1 | LOC_Os01g11920.1 | 0.807 |
| 4332080 | 4326546 | LOC_Os03g11910.1 | LOC_Os01g53920.1 | 0.776 |
| 4332080 | 4329448 | LOC_Os03g11910.1 | LOC_Os02g30620.1 | 0.879 |
| 4332080 | 4330159 | LOC_Os03g11910.1 | LOC_Os02g43350.1 | 0.401 |
| 4332080 | 4330971 | LOC_Os03g11910.1 | LOC_Os02g54820.1 | 0.545 |
| 4332080 | 4331168 | LOC_Os03g11910.1 | LOC_Os02g57470.1 | 0.404 |
| 4332080 | 4333079 | LOC_Os03g11910.1 | LOC_Os03g29260.1 | 0.413 |
| 4332080 | 4338096 | LOC_Os03g11910.1 | LOC_Os05g11990.1 | 0.928 |
| 4332080 | 4340563 | LOC_Os03g11910.1 | LOC_Os06g12370.1 | 0.438 |
| 4332080 | 4341219 | LOC_Os03g11910.1 | LOC_Os06g34690.1 | 0.843 |
| 4332080 | 4341853 | LOC_Os03g11910.1 | LOC_Os06g46600.1 | 0.653 |
| 4332080 | 4342673 | LOC_Os03g11910.1 | LOC_Os07g10350.1 | 0.437 |
| 4332080 | 4343196 | LOC_Os03g11910.1 | LOC_Os07g28800.1 | 0.91 |
| 4332080 | 4344935 | LOC_Os03g11910.1 | LOC_Os08g10608.1 | 0.406 |
| 4332080 | 4345065 | LOC_Os03g11910.1 | LOC_Os08g14990.1 | 0.776 |
| 4332080 | 4345628 | LOC_Os03g11910.1 | LOC_Os08g33200.1 | 0.88 |
| 4332080 | 4345708 | LOC_Os03g11910.1 | LOC_Os08g34650.1 | 0.776 |
| 4332080 | 4347005 | LOC_Os03g11910.1 | LOC_Os09g23740.1 | 0.88 |
| 4332080 | 4347095 | LOC_Os03g11910.1 | LOC_Os09g25540.1 | 0.776 |
| 4332080 | 4348731 | LOC_Os03g11910.1 | LOC_Os10g30580.1 | 0.551 |
| 4332129 | 4332241 | LOC_Os03g12590.1 | LOC_Os03g14260.1 | 0.431 |
| 4332129 | 4345819 | LOC_Os03g12590.1 | LOC_Os08g36994.1 | 0.844 |
| 4332129 | 4349719 | LOC_Os03g12590.1 | LOC_Os11g04220.1 | 0.595 |
| 4332129 | 4349939 | LOC_Os03g12590.1 | LOC_Os11g07910.1 | 0.492 |
| 4332129 | 4351436 | LOC_Os03g12590.1 | LOC_Os12g04030.1 | 0.595 |
| 4332129 | LOC_Os03g64080.1 | LOC_Os03g12590.1 | LOC_Os03g64080.1 | 0.41 |
| 4332190 | 4328135 | LOC_Os03g13460.1 | LOC_Os02g03060.1 | 0.717 |
| 4332190 | 4330866 | LOC_Os03g13460.1 | LOC_Os02g53120.1 | 0.687 |
| 4332190 | 4337886 | LOC_Os03g13460.1 | LOC_Os05g06840.1 | 0.585 |
| 4332190 | 4343388 | LOC_Os03g13460.1 | LOC_Os07g33370.1 | 0.732 |
| 4332241 | 4325022 | LOC_Os03g14260.1 | LOC_Os01g67770.1 | 0.426 |
| 4332241 | 4332129 | LOC_Os03g14260.1 | LOC_Os03g12590.1 | 0.431 |
| 4332258 | 4324364 | LOC_Os03g14540.1 | LOC_Os01g69030.2 | 0.573 |
| 4332258 | 4329416 | LOC_Os03g14540.1 | LOC_Os02g29530.1 | 0.724 |
| 4332258 | 4334290 | LOC_Os03g14540.1 | LOC_Os03g56460.1 | 0.46 |
| 4332258 | 4336977 | LOC_Os03g14540.1 | LOC_Os04g52370.1 | 0.484 |
| 4332258 | 4338096 | LOC_Os03g14540.1 | LOC_Os05g11990.1 | 0.491 |
| 4332258 | 4347311 | LOC_Os03g14540.1 | LOC_Os09g29070.1 | 0.46 |
| 4332258 | 4348853 | LOC_Os03g14540.1 | LOC_Os10g32980.1 | 0.485 |
| 4332380 | 4324709 | LOC_Os03g16290.1 | LOC_Os01g56570.1 | 0.405 |
| 4332380 | 4342645 | LOC_Os03g16290.1 | LOC_Os07g09690.1 | 0.543 |
| 4332380 | STLP1 | LOC_Os03g16290.1 | LOC_Os01g63970.1 | 0.408 |
| 4332492 | 4339523 | LOC_Os03g17940.1 | LOC_Os05g47660.1 | 0.408 |
| 4332567 | 4331005 | LOC_Os03g18840.1 | LOC_Os02g55260.1 | 0.901 |
| 4332567 | 4332049 | LOC_Os03g18840.1 | LOC_Os03g11510.1 | 0.825 |
| 4332567 | 4333574 | LOC_Os03g18840.1 | LOC_Os03g44530.1 | 0.912 |
| 4332567 | 4333814 | LOC_Os03g18840.1 | LOC_Os03g49210.1 | 0.973 |
| 4332567 | 4336833 | LOC_Os03g18840.1 | LOC_Os04g50660.1 | 0.997 |
| 4332567 | 4337228 | LOC_Os03g18840.1 | LOC_Os04g55700.1 | 0.602 |
| 4332567 | 4342673 | LOC_Os03g18840.1 | LOC_Os07g10350.1 | 0.983 |
| 4332567 | 4347787 | LOC_Os03g18840.1 | LOC_Os09g37860.1 | 0.909 |
| 4332567 | 4350837 | LOC_Os03g18840.1 | LOC_Os11g38170.1 | 0.821 |
| 4332567 | LOC_Os02g49270.1 | LOC_Os03g18840.1 | LOC_Os02g49270.1 | 0.784 |
| 4332567 | LOC_Os06g04340.1 | LOC_Os03g18840.1 | LOC_Os06g04340.1 | 0.405 |
| 4332628 | 4335058 | LOC_Os03g19680.1 | LOC_Os04g08740.1 | 0.555 |
| 4332628 | 4344267 | LOC_Os03g19680.1 | LOC_Os07g47820.1 | 0.471 |
| 4332628 | 4347172 | LOC_Os03g19680.1 | LOC_Os09g26880.1 | 0.557 |
| 4332696 | 4335058 | LOC_Os03g20780.1 | LOC_Os04g08740.1 | 0.461 |
| 4332696 | 4350996 | LOC_Os03g20780.1 | LOC_Os11g42040.1 | 0.845 |
| 4332784 | 4347823 | LOC_Os03g22040.1 | LOC_Os09g38450.1 | 0.428 |
| 4333060 | 4324511 | LOC_Os03g28300.1 | LOC_Os01g05620.1 | 0.404 |
| 4333060 | 4324660 | LOC_Os03g28300.1 | LOC_Os01g52380.1 | 0.404 |
| 4333060 | 4333771 | LOC_Os03g28300.1 | LOC_Os03g48320.1 | 0.404 |
| 4333060 | 4334214 | LOC_Os03g28300.1 | LOC_Os03g55560.1 | 0.466 |
| 4333060 | 4334935 | LOC_Os03g28300.1 | LOC_Os04g02110.1 | 0.404 |
| 4333060 | 4335058 | LOC_Os03g28300.1 | LOC_Os04g08740.1 | 0.49 |
| 4333060 | 4336120 | LOC_Os03g28300.1 | LOC_Os04g39460.1 | 0.404 |
| 4333060 | 4338096 | LOC_Os03g28300.1 | LOC_Os05g11990.1 | 0.703 |
| 4333060 | 4338531 | LOC_Os03g28300.1 | LOC_Os05g29030.1 | 0.78 |
| 4333060 | 4339763 | LOC_Os03g28300.1 | LOC_Os05g50970.1 | 0.78 |
| 4333060 | 4340110 | LOC_Os03g28300.1 | LOC_Os06g05359.1 | 0.404 |
| 4333060 | 4340753 | LOC_Os03g28300.1 | LOC_Os06g16450.1 | 0.404 |
| 4333060 | 4341646 | LOC_Os03g28300.1 | LOC_Os06g43670.1 | 0.404 |
| 4333060 | 4342017 | LOC_Os03g28300.1 | LOC_Os06g49430.1 | 0.516 |
| 4333060 | 4343409 | LOC_Os03g28300.1 | LOC_Os07g33730.1 | 0.404 |
| 4333060 | 4344698 | LOC_Os03g28300.1 | LOC_Os08g06060.1 | 0.514 |
| 4333060 | 4345202 | LOC_Os03g28300.1 | LOC_Os08g20000.1 | 0.404 |
| 4333060 | 4345297 | LOC_Os03g28300.1 | LOC_Os08g24380.1 | 0.404 |
| 4333060 | 4345628 | LOC_Os03g28300.1 | LOC_Os08g33200.1 | 0.562 |
| 4333060 | 4347005 | LOC_Os03g28300.1 | LOC_Os09g23740.1 | 0.562 |
| 4333060 | 4347787 | LOC_Os03g28300.1 | LOC_Os09g37860.1 | 0.408 |
| 4333060 | 4350109 | LOC_Os03g28300.1 | LOC_Os11g11770.1 | 0.404 |
| 4333060 | 4350130 | LOC_Os03g28300.1 | LOC_Os11g12300.1 | 0.404 |
| 4333060 | 4350814 | LOC_Os03g28300.1 | LOC_Os11g37860.1 | 0.404 |
| 4333060 | 4350954 | LOC_Os03g28300.1 | LOC_Os11g40780.1 | 0.404 |
| 4333060 | 4350996 | LOC_Os03g28300.1 | LOC_Os11g42040.1 | 0.404 |
| 4333060 | 4351038 | LOC_Os03g28300.1 | LOC_Os11g43250.1 | 0.404 |
| 4333060 | 4351041 | LOC_Os03g28300.1 | LOC_Os11g43390.1 | 0.404 |
| 4333060 | 4351749 | LOC_Os03g28300.1 | LOC_Os12g10410.1 | 0.404 |
| 4333060 | 4352130 | LOC_Os03g28300.1 | LOC_Os12g25170.1 | 0.404 |
| 4333060 | 4352270 | LOC_Os03g28300.1 | LOC_Os12g31620.1 | 0.404 |
| 4333060 | IRL7 | LOC_Os03g28300.1 | LOC_Os03g11360.2 | 0.404 |
| 4333060 | LOC_Os02g49270.1 | LOC_Os03g28300.1 | LOC_Os02g49270.1 | 0.408 |
| 4333079 | 4324082 | LOC_Os03g29260.1 | LOC_Os01g06320.1 | 0.618 |
| 4333079 | 4332080 | LOC_Os03g29260.1 | LOC_Os03g11910.1 | 0.413 |
| 4333079 | 4344935 | LOC_Os03g29260.1 | LOC_Os08g10608.1 | 0.763 |
| 4333493 | 4328305 | LOC_Os03g42750.1 | LOC_Os02g05620.1 | 0.857 |
| 4333493 | 4346808 | LOC_Os03g42750.1 | LOC_Os09g17810.1 | 0.855 |
| 4333493 | 4349875 | LOC_Os03g42750.1 | LOC_Os11g06700.1 | 0.984 |
| 4333574 | 4331005 | LOC_Os03g44530.1 | LOC_Os02g55260.1 | 0.499 |
| 4333574 | 4332567 | LOC_Os03g44530.1 | LOC_Os03g18840.1 | 0.912 |
| 4333574 | 4333814 | LOC_Os03g44530.1 | LOC_Os03g49210.1 | 0.985 |
| 4333574 | 4336833 | LOC_Os03g44530.1 | LOC_Os04g50660.1 | 0.871 |
| 4333574 | 4342673 | LOC_Os03g44530.1 | LOC_Os07g10350.1 | 0.719 |
| 4333574 | 4347787 | LOC_Os03g44530.1 | LOC_Os09g37860.1 | 0.866 |
| 4333574 | 4350837 | LOC_Os03g44530.1 | LOC_Os11g38170.1 | 0.532 |
| 4333574 | LOC_Os02g49270.1 | LOC_Os03g44530.1 | LOC_Os02g49270.1 | 0.819 |
| 4333586 | 4341510 | LOC_Os03g44760.1 | LOC_Os06g40840.1 | 0.776 |
| 4333771 | 4326546 | LOC_Os03g48320.1 | LOC_Os01g53920.1 | 0.404 |
| 4333771 | 4327667 | LOC_Os03g48320.1 | LOC_Os01g40590.1 | 0.404 |
| 4333771 | 4328725 | LOC_Os03g48320.1 | LOC_Os02g12440.1 | 0.404 |
| 4333771 | 4330701 | LOC_Os03g48320.1 | LOC_Os02g50970.1 | 0.404 |
| 4333771 | 4331134 | LOC_Os03g48320.1 | LOC_Os02g57080.1 | 0.404 |
| 4333771 | 4333060 | LOC_Os03g48320.1 | LOC_Os03g28300.1 | 0.404 |
| 4333771 | 4334921 | LOC_Os03g48320.1 | LOC_Os04g01874.1 | 0.404 |
| 4333771 | 4335824 | LOC_Os03g48320.1 | LOC_Os04g34250.1 | 0.404 |
| 4333771 | 4337251 | LOC_Os03g48320.1 | LOC_Os04g56090.1 | 0.404 |
| 4333771 | 4338080 | LOC_Os03g48320.1 | LOC_Os05g11750.1 | 0.404 |
| 4333771 | 4338096 | LOC_Os03g48320.1 | LOC_Os05g11990.1 | 0.628 |
| 4333771 | 4339142 | LOC_Os03g48320.1 | LOC_Os05g40770.1 | 0.404 |
| 4333771 | 4341066 | LOC_Os03g48320.1 | LOC_Os06g29080.1 | 0.404 |
| 4333771 | 4341568 | LOC_Os03g48320.1 | LOC_Os06g41980.1 | 0.404 |
| 4333771 | 4342396 | LOC_Os03g48320.1 | LOC_Os07g05370.1 | 0.404 |
| 4333771 | 4342410 | LOC_Os03g48320.1 | LOC_Os07g05620.1 | 0.404 |
| 4333771 | 4343699 | LOC_Os03g48320.1 | LOC_Os07g38810.1 | 0.404 |
| 4333771 | 4343832 | LOC_Os03g48320.1 | LOC_Os07g41140.1 | 0.404 |
| 4333771 | 4345065 | LOC_Os03g48320.1 | LOC_Os08g14990.1 | 0.404 |
| 4333771 | 4345628 | LOC_Os03g48320.1 | LOC_Os08g33200.1 | 0.806 |
| 4333771 | 4345708 | LOC_Os03g48320.1 | LOC_Os08g34650.1 | 0.404 |
| 4333771 | 4347005 | LOC_Os03g48320.1 | LOC_Os09g23740.1 | 0.806 |
| 4333771 | 4352691 | LOC_Os03g48320.1 | LOC_Os12g40419.1 | 0.404 |
| 4333771 | 4352904 | LOC_Os03g48320.1 | LOC_Os12g44090.1 | 0.404 |
| 4333814 | 4330796 | LOC_Os03g49210.1 | LOC_Os02g52250.1 | 0.635 |
| 4333814 | 4331005 | LOC_Os03g49210.1 | LOC_Os02g55260.1 | 0.835 |
| 4333814 | 4332567 | LOC_Os03g49210.1 | LOC_Os03g18840.1 | 0.973 |
| 4333814 | 4333574 | LOC_Os03g49210.1 | LOC_Os03g44530.1 | 0.985 |
| 4333814 | 4336833 | LOC_Os03g49210.1 | LOC_Os04g50660.1 | 0.966 |
| 4333814 | 4340964 | LOC_Os03g49210.1 | LOC_Os06g23530.1 | 0.413 |
| 4333814 | 4342472 | LOC_Os03g49210.1 | LOC_Os07g06980.1 | 0.628 |
| 4333814 | 4342673 | LOC_Os03g49210.1 | LOC_Os07g10350.1 | 0.978 |
| 4333814 | 4343339 | LOC_Os03g49210.1 | LOC_Os07g32430.1 | 0.451 |
| 4333814 | 4345304 | LOC_Os03g49210.1 | LOC_Os08g24760.1 | 0.413 |
| 4333814 | 4347787 | LOC_Os03g49210.1 | LOC_Os09g37860.1 | 0.998 |
| 4333814 | 4350837 | LOC_Os03g49210.1 | LOC_Os11g38170.1 | 0.924 |
| 4333814 | LOC_Os02g49270.1 | LOC_Os03g49210.1 | LOC_Os02g49270.1 | 0.997 |
| 4333882 | 4325329 | LOC_Os03g50310.1 | LOC_Os01g08700.1 | 0.421 |
| 4333882 | 4340892 | LOC_Os03g50310.1 | LOC_Os06g21590.1 | 0.635 |
| 4333882 | 4342017 | LOC_Os03g50310.1 | LOC_Os06g49430.1 | 0.414 |
| 4333882 | 4344698 | LOC_Os03g50310.1 | LOC_Os08g06060.1 | 0.414 |
| 4334029 | 4334114 | LOC_Os03g52630.1 | LOC_Os03g53800.1 | 0.88 |
| 4334029 | 4348853 | LOC_Os03g52630.1 | LOC_Os10g32980.1 | 0.593 |
| 4334114 | 4334029 | LOC_Os03g53800.1 | LOC_Os03g52630.1 | 0.88 |
| 4334114 | BGLU2 | LOC_Os03g53800.1 | LOC_Os01g59819.1 | 0.412 |
| 4334135 | 4330868 | LOC_Os03g54084.1 | LOC_Os02g53140.1 | 0.501 |
| 4334214 | 4326546 | LOC_Os03g55560.1 | LOC_Os01g53920.1 | 0.927 |
| 4334214 | 4330701 | LOC_Os03g55560.1 | LOC_Os02g50970.1 | 0.465 |
| 4334214 | 4330866 | LOC_Os03g55560.1 | LOC_Os02g53120.1 | 0.43 |
| 4334214 | 4333060 | LOC_Os03g55560.1 | LOC_Os03g28300.1 | 0.466 |
| 4334214 | 4338531 | LOC_Os03g55560.1 | LOC_Os05g29030.1 | 0.593 |
| 4334214 | 4339142 | LOC_Os03g55560.1 | LOC_Os05g40770.1 | 0.438 |
| 4334214 | 4339763 | LOC_Os03g55560.1 | LOC_Os05g50970.1 | 0.593 |
| 4334214 | 4342017 | LOC_Os03g55560.1 | LOC_Os06g49430.1 | 0.714 |
| 4334214 | 4342410 | LOC_Os03g55560.1 | LOC_Os07g05620.1 | 0.45 |
| 4334214 | 4344698 | LOC_Os03g55560.1 | LOC_Os08g06060.1 | 0.687 |
| 4334214 | 4345065 | LOC_Os03g55560.1 | LOC_Os08g14990.1 | 0.927 |
| 4334214 | 4345628 | LOC_Os03g55560.1 | LOC_Os08g33200.1 | 0.408 |
| 4334214 | 4345708 | LOC_Os03g55560.1 | LOC_Os08g34650.1 | 0.927 |
| 4334214 | 4347005 | LOC_Os03g55560.1 | LOC_Os09g23740.1 | 0.408 |
| 4334214 | 4347095 | LOC_Os03g55560.1 | LOC_Os09g25540.1 | 0.927 |
| 4334214 | IRL7 | LOC_Os03g55560.1 | LOC_Os03g11360.2 | 0.604 |
| 4334290 | 4327790 | LOC_Os03g56460.1 | LOC_Os01g13570.1 | 0.616 |
| 4334290 | 4328828 | LOC_Os03g56460.1 | LOC_Os02g14110.1 | 0.566 |
| 4334290 | 4330709 | LOC_Os03g56460.1 | LOC_Os02g51070.1 | 0.549 |
| 4334290 | 4330971 | LOC_Os03g56460.1 | LOC_Os02g54820.1 | 0.53 |
| 4334290 | 4332258 | LOC_Os03g56460.1 | LOC_Os03g14540.1 | 0.46 |
| 4334290 | 4336977 | LOC_Os03g56460.1 | LOC_Os04g52370.1 | 0.529 |
| 4334290 | 4338096 | LOC_Os03g56460.1 | LOC_Os05g11990.1 | 0.432 |
| 4334290 | 4341219 | LOC_Os03g56460.1 | LOC_Os06g34690.1 | 0.427 |
| 4334290 | 4341252 | LOC_Os03g56460.1 | LOC_Os06g35540.1 | 0.566 |
| 4334290 | 4342770 | LOC_Os03g56460.1 | LOC_Os07g12640.1 | 0.497 |
| 4334290 | 4344584 | LOC_Os03g56460.1 | LOC_Os08g03570.1 | 0.46 |
| 4334290 | 4347172 | LOC_Os03g56460.1 | LOC_Os09g26880.1 | 0.566 |
| 4334290 | 4347311 | LOC_Os03g56460.1 | LOC_Os09g29070.1 | 0.938 |
| 4334290 | 4352803 | LOC_Os03g56460.1 | LOC_Os12g42230.1 | 0.948 |
| 4334412 | 4342770 | LOC_Os03g58100.1 | LOC_Os07g12640.1 | 0.431 |
| 4334795 | 4345365 | LOC_Os03g63360.1 | LOC_Os08g27010.1 | 0.6 |
| 4334795 | 4346508 | LOC_Os03g63360.1 | LOC_Os09g07570.1 | 0.426 |
| 4334921 | 4324511 | LOC_Os04g01874.1 | LOC_Os01g05620.1 | 0.404 |
| 4334921 | 4324660 | LOC_Os04g01874.1 | LOC_Os01g52380.1 | 0.404 |
| 4334921 | 4333771 | LOC_Os04g01874.1 | LOC_Os03g48320.1 | 0.404 |
| 4334921 | 4334935 | LOC_Os04g01874.1 | LOC_Os04g02110.1 | 0.404 |
| 4334921 | 4335058 | LOC_Os04g01874.1 | LOC_Os04g08740.1 | 0.415 |
| 4334921 | 4336120 | LOC_Os04g01874.1 | LOC_Os04g39460.1 | 0.404 |
| 4334921 | 4338096 | LOC_Os04g01874.1 | LOC_Os05g11990.1 | 0.703 |
| 4334921 | 4338531 | LOC_Os04g01874.1 | LOC_Os05g29030.1 | 0.703 |
| 4334921 | 4339763 | LOC_Os04g01874.1 | LOC_Os05g50970.1 | 0.703 |
| 4334921 | 4340110 | LOC_Os04g01874.1 | LOC_Os06g05359.1 | 0.404 |
| 4334921 | 4340753 | LOC_Os04g01874.1 | LOC_Os06g16450.1 | 0.404 |
| 4334921 | 4341646 | LOC_Os04g01874.1 | LOC_Os06g43670.1 | 0.404 |
| 4334921 | 4342017 | LOC_Os04g01874.1 | LOC_Os06g49430.1 | 0.492 |
| 4334921 | 4343409 | LOC_Os04g01874.1 | LOC_Os07g33730.1 | 0.404 |
| 4334921 | 4344698 | LOC_Os04g01874.1 | LOC_Os08g06060.1 | 0.492 |
| 4334921 | 4345202 | LOC_Os04g01874.1 | LOC_Os08g20000.1 | 0.404 |
| 4334921 | 4345297 | LOC_Os04g01874.1 | LOC_Os08g24380.1 | 0.404 |
| 4334921 | 4345628 | LOC_Os04g01874.1 | LOC_Os08g33200.1 | 0.562 |
| 4334921 | 4347005 | LOC_Os04g01874.1 | LOC_Os09g23740.1 | 0.562 |
| 4334921 | 4347787 | LOC_Os04g01874.1 | LOC_Os09g37860.1 | 0.408 |
| 4334921 | 4350109 | LOC_Os04g01874.1 | LOC_Os11g11770.1 | 0.404 |
| 4334921 | 4350130 | LOC_Os04g01874.1 | LOC_Os11g12300.1 | 0.404 |
| 4334921 | 4350814 | LOC_Os04g01874.1 | LOC_Os11g37860.1 | 0.404 |
| 4334921 | 4350954 | LOC_Os04g01874.1 | LOC_Os11g40780.1 | 0.404 |
| 4334921 | 4350996 | LOC_Os04g01874.1 | LOC_Os11g42040.1 | 0.404 |
| 4334921 | 4351038 | LOC_Os04g01874.1 | LOC_Os11g43250.1 | 0.404 |
| 4334921 | 4351041 | LOC_Os04g01874.1 | LOC_Os11g43390.1 | 0.404 |
| 4334921 | 4351749 | LOC_Os04g01874.1 | LOC_Os12g10410.1 | 0.404 |
| 4334921 | 4352130 | LOC_Os04g01874.1 | LOC_Os12g25170.1 | 0.404 |
| 4334921 | 4352270 | LOC_Os04g01874.1 | LOC_Os12g31620.1 | 0.404 |
| 4334921 | IRL7 | LOC_Os04g01874.1 | LOC_Os03g11360.2 | 0.404 |
| 4334921 | LOC_Os02g49270.1 | LOC_Os04g01874.1 | LOC_Os02g49270.1 | 0.408 |
| 4334935 | 4326546 | LOC_Os04g02110.1 | LOC_Os01g53920.1 | 0.404 |
| 4334935 | 4327667 | LOC_Os04g02110.1 | LOC_Os01g40590.1 | 0.404 |
| 4334935 | 4328725 | LOC_Os04g02110.1 | LOC_Os02g12440.1 | 0.404 |
| 4334935 | 4330701 | LOC_Os04g02110.1 | LOC_Os02g50970.1 | 0.404 |
| 4334935 | 4331134 | LOC_Os04g02110.1 | LOC_Os02g57080.1 | 0.404 |
| 4334935 | 4333060 | LOC_Os04g02110.1 | LOC_Os03g28300.1 | 0.404 |
| 4334935 | 4334921 | LOC_Os04g02110.1 | LOC_Os04g01874.1 | 0.404 |
| 4334935 | 4335824 | LOC_Os04g02110.1 | LOC_Os04g34250.1 | 0.404 |
| 4334935 | 4337251 | LOC_Os04g02110.1 | LOC_Os04g56090.1 | 0.404 |
| 4334935 | 4338080 | LOC_Os04g02110.1 | LOC_Os05g11750.1 | 0.404 |
| 4334935 | 4338096 | LOC_Os04g02110.1 | LOC_Os05g11990.1 | 0.628 |
| 4334935 | 4339142 | LOC_Os04g02110.1 | LOC_Os05g40770.1 | 0.404 |
| 4334935 | 4341066 | LOC_Os04g02110.1 | LOC_Os06g29080.1 | 0.404 |
| 4334935 | 4341568 | LOC_Os04g02110.1 | LOC_Os06g41980.1 | 0.404 |
| 4334935 | 4342396 | LOC_Os04g02110.1 | LOC_Os07g05370.1 | 0.404 |
| 4334935 | 4342410 | LOC_Os04g02110.1 | LOC_Os07g05620.1 | 0.404 |
| 4334935 | 4343699 | LOC_Os04g02110.1 | LOC_Os07g38810.1 | 0.404 |
| 4334935 | 4343832 | LOC_Os04g02110.1 | LOC_Os07g41140.1 | 0.404 |
| 4334935 | 4345065 | LOC_Os04g02110.1 | LOC_Os08g14990.1 | 0.404 |
| 4334935 | 4345628 | LOC_Os04g02110.1 | LOC_Os08g33200.1 | 0.806 |
| 4334935 | 4345708 | LOC_Os04g02110.1 | LOC_Os08g34650.1 | 0.404 |
| 4334935 | 4347005 | LOC_Os04g02110.1 | LOC_Os09g23740.1 | 0.806 |
| 4334935 | 4347095 | LOC_Os04g02110.1 | LOC_Os09g25540.1 | 0.404 |
| 4334935 | 4352691 | LOC_Os04g02110.1 | LOC_Os12g40419.1 | 0.404 |
| 4334935 | 4352904 | LOC_Os04g02110.1 | LOC_Os12g44090.1 | 0.404 |
| 4335058 | 4325022 | LOC_Os04g08740.1 | LOC_Os01g67770.1 | 0.861 |
| 4335058 | 4326546 | LOC_Os04g08740.1 | LOC_Os01g53920.1 | 0.659 |
| 4335058 | 4327667 | LOC_Os04g08740.1 | LOC_Os01g40590.1 | 0.415 |
| 4335058 | 4328725 | LOC_Os04g08740.1 | LOC_Os02g12440.1 | 0.415 |
| 4335058 | 4330701 | LOC_Os04g08740.1 | LOC_Os02g50970.1 | 0.864 |
| 4335058 | 4330971 | LOC_Os04g08740.1 | LOC_Os02g54820.1 | 0.401 |
| 4335058 | 4331134 | LOC_Os04g08740.1 | LOC_Os02g57080.1 | 0.415 |
| 4335058 | 4332628 | LOC_Os04g08740.1 | LOC_Os03g19680.1 | 0.555 |
| 4335058 | 4332696 | LOC_Os04g08740.1 | LOC_Os03g20780.1 | 0.461 |
| 4335058 | 4333060 | LOC_Os04g08740.1 | LOC_Os03g28300.1 | 0.49 |
| 4335058 | 4334921 | LOC_Os04g08740.1 | LOC_Os04g01874.1 | 0.415 |
| 4335058 | 4335824 | LOC_Os04g08740.1 | LOC_Os04g34250.1 | 0.415 |
| 4335058 | 4337251 | LOC_Os04g08740.1 | LOC_Os04g56090.1 | 0.415 |
| 4335058 | 4338080 | LOC_Os04g08740.1 | LOC_Os05g11750.1 | 0.415 |
| 4335058 | 4338096 | LOC_Os04g08740.1 | LOC_Os05g11990.1 | 0.528 |
| 4335058 | 4339142 | LOC_Os04g08740.1 | LOC_Os05g40770.1 | 0.461 |
| 4335058 | 4341066 | LOC_Os04g08740.1 | LOC_Os06g29080.1 | 0.415 |
| 4335058 | 4341568 | LOC_Os04g08740.1 | LOC_Os06g41980.1 | 0.415 |
| 4335058 | 4342017 | LOC_Os04g08740.1 | LOC_Os06g49430.1 | 0.48 |
| 4335058 | 4342396 | LOC_Os04g08740.1 | LOC_Os07g05370.1 | 0.415 |
| 4335058 | 4342410 | LOC_Os04g08740.1 | LOC_Os07g05620.1 | 0.415 |
| 4335058 | 4343699 | LOC_Os04g08740.1 | LOC_Os07g38810.1 | 0.415 |
| 4335058 | 4343832 | LOC_Os04g08740.1 | LOC_Os07g41140.1 | 0.415 |
| 4335058 | 4344698 | LOC_Os04g08740.1 | LOC_Os08g06060.1 | 0.48 |
| 4335058 | 4345065 | LOC_Os04g08740.1 | LOC_Os08g14990.1 | 0.659 |
| 4335058 | 4345708 | LOC_Os04g08740.1 | LOC_Os08g34650.1 | 0.659 |
| 4335058 | 4347095 | LOC_Os04g08740.1 | LOC_Os09g25540.1 | 0.659 |
| 4335058 | 4349531 | LOC_Os04g08740.1 | LOC_Os11g01140.1 | 0.522 |
| 4335058 | 4352691 | LOC_Os04g08740.1 | LOC_Os12g40419.1 | 0.415 |
| 4335058 | 4352803 | LOC_Os04g08740.1 | LOC_Os12g42230.1 | 0.42 |
| 4335058 | 4352904 | LOC_Os04g08740.1 | LOC_Os12g44090.1 | 0.415 |
| 4335058 | LOC_Os03g20790.1 | LOC_Os04g08740.1 | LOC_Os03g20790.1 | 0.461 |
| 4335089 | 4326270 | LOC_Os04g09860.1 | LOC_Os01g16414.1 | 0.697 |
| 4335089 | 4326546 | LOC_Os04g09860.1 | LOC_Os01g53920.1 | 0.606 |
| 4335089 | 4331144 | LOC_Os04g09860.1 | LOC_Os02g57190.1 | 0.727 |
| 4335089 | 4337317 | LOC_Os04g09860.1 | LOC_Os04g56980.1 | 0.603 |
| 4335089 | 4338096 | LOC_Os04g09860.1 | LOC_Os05g11990.1 | 0.48 |
| 4335089 | 4343299 | LOC_Os04g09860.1 | LOC_Os07g31450.1 | 0.444 |
| 4335089 | 4345065 | LOC_Os04g09860.1 | LOC_Os08g14990.1 | 0.606 |
| 4335089 | 4345628 | LOC_Os04g09860.1 | LOC_Os08g33200.1 | 0.514 |
| 4335089 | 4345708 | LOC_Os04g09860.1 | LOC_Os08g34650.1 | 0.606 |
| 4335089 | 4347005 | LOC_Os04g09860.1 | LOC_Os09g23740.1 | 0.514 |
| 4335089 | 4347095 | LOC_Os04g09860.1 | LOC_Os09g25540.1 | 0.606 |
| 4335089 | 4348805 | LOC_Os04g09860.1 | LOC_Os10g31970.1 | 0.435 |
| 4335673 | 4328828 | LOC_Os04g32020.1 | LOC_Os02g14110.1 | 0.924 |
| 4335673 | 4341252 | LOC_Os04g32020.1 | LOC_Os06g35540.1 | 0.924 |
| 4335673 | 4352803 | LOC_Os04g32020.1 | LOC_Os12g42230.1 | 0.569 |
| 4335824 | 4324511 | LOC_Os04g34250.1 | LOC_Os01g05620.1 | 0.404 |
| 4335824 | 4324660 | LOC_Os04g34250.1 | LOC_Os01g52380.1 | 0.404 |
| 4335824 | 4333771 | LOC_Os04g34250.1 | LOC_Os03g48320.1 | 0.404 |
| 4335824 | 4334935 | LOC_Os04g34250.1 | LOC_Os04g02110.1 | 0.404 |
| 4335824 | 4335058 | LOC_Os04g34250.1 | LOC_Os04g08740.1 | 0.415 |
| 4335824 | 4336120 | LOC_Os04g34250.1 | LOC_Os04g39460.1 | 0.404 |
| 4335824 | 4338096 | LOC_Os04g34250.1 | LOC_Os05g11990.1 | 0.703 |
| 4335824 | 4338531 | LOC_Os04g34250.1 | LOC_Os05g29030.1 | 0.703 |
| 4335824 | 4339763 | LOC_Os04g34250.1 | LOC_Os05g50970.1 | 0.703 |
| 4335824 | 4340110 | LOC_Os04g34250.1 | LOC_Os06g05359.1 | 0.404 |
| 4335824 | 4340753 | LOC_Os04g34250.1 | LOC_Os06g16450.1 | 0.404 |
| 4335824 | 4341646 | LOC_Os04g34250.1 | LOC_Os06g43670.1 | 0.404 |
| 4335824 | 4342017 | LOC_Os04g34250.1 | LOC_Os06g49430.1 | 0.469 |
| 4335824 | 4343409 | LOC_Os04g34250.1 | LOC_Os07g33730.1 | 0.404 |
| 4335824 | 4344698 | LOC_Os04g34250.1 | LOC_Os08g06060.1 | 0.468 |
| 4335824 | 4345202 | LOC_Os04g34250.1 | LOC_Os08g20000.1 | 0.404 |
| 4335824 | 4345297 | LOC_Os04g34250.1 | LOC_Os08g24380.1 | 0.404 |
| 4335824 | 4345628 | LOC_Os04g34250.1 | LOC_Os08g33200.1 | 0.562 |
| 4335824 | 4347005 | LOC_Os04g34250.1 | LOC_Os09g23740.1 | 0.562 |
| 4335824 | 4347787 | LOC_Os04g34250.1 | LOC_Os09g37860.1 | 0.408 |
| 4335824 | 4350109 | LOC_Os04g34250.1 | LOC_Os11g11770.1 | 0.404 |
| 4335824 | 4350130 | LOC_Os04g34250.1 | LOC_Os11g12300.1 | 0.404 |
| 4335824 | 4350814 | LOC_Os04g34250.1 | LOC_Os11g37860.1 | 0.404 |
| 4335824 | 4350954 | LOC_Os04g34250.1 | LOC_Os11g40780.1 | 0.404 |
| 4335824 | 4350996 | LOC_Os04g34250.1 | LOC_Os11g42040.1 | 0.404 |
| 4335824 | 4351038 | LOC_Os04g34250.1 | LOC_Os11g43250.1 | 0.404 |
| 4335824 | 4351041 | LOC_Os04g34250.1 | LOC_Os11g43390.1 | 0.404 |
| 4335824 | 4351749 | LOC_Os04g34250.1 | LOC_Os12g10410.1 | 0.404 |
| 4335824 | 4352130 | LOC_Os04g34250.1 | LOC_Os12g25170.1 | 0.404 |
| 4335824 | 4352270 | LOC_Os04g34250.1 | LOC_Os12g31620.1 | 0.404 |
| 4335824 | IRL7 | LOC_Os04g34250.1 | LOC_Os03g11360.2 | 0.404 |
| 4335824 | LOC_Os02g49270.1 | LOC_Os04g34250.1 | LOC_Os02g49270.1 | 0.408 |
| 4336120 | 4326546 | LOC_Os04g39460.1 | LOC_Os01g53920.1 | 0.404 |
| 4336120 | 4327667 | LOC_Os04g39460.1 | LOC_Os01g40590.1 | 0.404 |
| 4336120 | 4328725 | LOC_Os04g39460.1 | LOC_Os02g12440.1 | 0.404 |
| 4336120 | 4330701 | LOC_Os04g39460.1 | LOC_Os02g50970.1 | 0.404 |
| 4336120 | 4331134 | LOC_Os04g39460.1 | LOC_Os02g57080.1 | 0.404 |
| 4336120 | 4333060 | LOC_Os04g39460.1 | LOC_Os03g28300.1 | 0.404 |
| 4336120 | 4334921 | LOC_Os04g39460.1 | LOC_Os04g01874.1 | 0.404 |
| 4336120 | 4335824 | LOC_Os04g39460.1 | LOC_Os04g34250.1 | 0.404 |
| 4336120 | 4337251 | LOC_Os04g39460.1 | LOC_Os04g56090.1 | 0.404 |
| 4336120 | 4338080 | LOC_Os04g39460.1 | LOC_Os05g11750.1 | 0.404 |
| 4336120 | 4338096 | LOC_Os04g39460.1 | LOC_Os05g11990.1 | 0.628 |
| 4336120 | 4339142 | LOC_Os04g39460.1 | LOC_Os05g40770.1 | 0.404 |
| 4336120 | 4341066 | LOC_Os04g39460.1 | LOC_Os06g29080.1 | 0.404 |
| 4336120 | 4341568 | LOC_Os04g39460.1 | LOC_Os06g41980.1 | 0.404 |
| 4336120 | 4342396 | LOC_Os04g39460.1 | LOC_Os07g05370.1 | 0.404 |
| 4336120 | 4342410 | LOC_Os04g39460.1 | LOC_Os07g05620.1 | 0.404 |
| 4336120 | 4343699 | LOC_Os04g39460.1 | LOC_Os07g38810.1 | 0.404 |
| 4336120 | 4343832 | LOC_Os04g39460.1 | LOC_Os07g41140.1 | 0.404 |
| 4336120 | 4345065 | LOC_Os04g39460.1 | LOC_Os08g14990.1 | 0.404 |
| 4336120 | 4345628 | LOC_Os04g39460.1 | LOC_Os08g33200.1 | 0.806 |
| 4336120 | 4345708 | LOC_Os04g39460.1 | LOC_Os08g34650.1 | 0.404 |
| 4336120 | 4347005 | LOC_Os04g39460.1 | LOC_Os09g23740.1 | 0.806 |
| 4336120 | 4347095 | LOC_Os04g39460.1 | LOC_Os09g25540.1 | 0.404 |
| 4336120 | 4352691 | LOC_Os04g39460.1 | LOC_Os12g40419.1 | 0.404 |
| 4336120 | 4352904 | LOC_Os04g39460.1 | LOC_Os12g44090.1 | 0.404 |
| 4336833 | 4331005 | LOC_Os04g50660.1 | LOC_Os02g55260.1 | 0.903 |
| 4336833 | 4331367 | LOC_Os04g50660.1 | LOC_Os03g02110.1 | 0.535 |
| 4336833 | 4332567 | LOC_Os04g50660.1 | LOC_Os03g18840.1 | 0.997 |
| 4336833 | 4333574 | LOC_Os04g50660.1 | LOC_Os03g44530.1 | 0.871 |
| 4336833 | 4333814 | LOC_Os04g50660.1 | LOC_Os03g49210.1 | 0.966 |
| 4336833 | 4337228 | LOC_Os04g50660.1 | LOC_Os04g55700.1 | 0.412 |
| 4336833 | 4340964 | LOC_Os04g50660.1 | LOC_Os06g23530.1 | 0.565 |
| 4336833 | 4342673 | LOC_Os04g50660.1 | LOC_Os07g10350.1 | 0.996 |
| 4336833 | 4343339 | LOC_Os04g50660.1 | LOC_Os07g32430.1 | 0.562 |
| 4336833 | 4345304 | LOC_Os04g50660.1 | LOC_Os08g24760.1 | 0.562 |
| 4336833 | 4347787 | LOC_Os04g50660.1 | LOC_Os09g37860.1 | 0.966 |
| 4336833 | 4350837 | LOC_Os04g50660.1 | LOC_Os11g38170.1 | 0.966 |
| 4336833 | LOC_Os02g49270.1 | LOC_Os04g50660.1 | LOC_Os02g49270.1 | 0.968 |
| 4336851 | 4337658 | LOC_Os04g50930.1 | LOC_Os05g03100.1 | 0.495 |
| 4336977 | 4330971 | LOC_Os04g52370.1 | LOC_Os02g54820.1 | 0.936 |
| 4336977 | 4332258 | LOC_Os04g52370.1 | LOC_Os03g14540.1 | 0.484 |
| 4336977 | 4334290 | LOC_Os04g52370.1 | LOC_Os03g56460.1 | 0.529 |
| 4336977 | 4338096 | LOC_Os04g52370.1 | LOC_Os05g11990.1 | 0.567 |
| 4336977 | 4343352 | LOC_Os04g52370.1 | LOC_Os07g32620.1 | 0.426 |
| 4336977 | 4344584 | LOC_Os04g52370.1 | LOC_Os08g03570.1 | 0.484 |
| 4336977 | 4347311 | LOC_Os04g52370.1 | LOC_Os09g29070.1 | 0.529 |
| 4336977 | 4348853 | LOC_Os04g52370.1 | LOC_Os10g32980.1 | 0.584 |
| 4336992 | 4331253 | LOC_Os04g52550.1 | LOC_Os02g58490.1 | 0.917 |
| 4337063 | 4324980 | LOC_Os04g53410.1 | LOC_Os01g67970.1 | 0.465 |
| 4337063 | 4328287 | LOC_Os04g53410.1 | LOC_Os02g05340.1 | 0.903 |
| 4337063 | 4346276 | LOC_Os04g53410.1 | LOC_Os08g44050.1 | 0.454 |
| 4337164 | 4329438 | LOC_Os04g54790.1 | LOC_Os02g30310.1 | 0.401 |
| 4337164 | 4330159 | LOC_Os04g54790.1 | LOC_Os02g43350.1 | 0.567 |
| 4337178 | 4344329 | LOC_Os04g55060.1 | LOC_Os07g48610.1 | 0.438 |
| 4337178 | 4347677 | LOC_Os04g55060.1 | LOC_Os09g36240.1 | 0.438 |
| 4337178 | 4348486 | LOC_Os04g55060.1 | LOC_Os10g22960.1 | 0.438 |
| 4337178 | 4352803 | LOC_Os04g55060.1 | LOC_Os12g42230.1 | 0.408 |
| 4337228 | 4324428 | LOC_Os04g55700.1 | LOC_Os01g53070.1 | 0.785 |
| 4337228 | 4326378 | LOC_Os04g55700.1 | LOC_Os01g40980.1 | 0.401 |
| 4337228 | 4331991 | LOC_Os04g55700.1 | LOC_Os03g10780.1 | 0.427 |
| 4337228 | 4332567 | LOC_Os04g55700.1 | LOC_Os03g18840.1 | 0.602 |
| 4337228 | 4336833 | LOC_Os04g55700.1 | LOC_Os04g50660.1 | 0.412 |
| 4337228 | 4351770 | LOC_Os04g55700.1 | LOC_Os12g10670.1 | 0.786 |
| 4337228 | 4352699 | LOC_Os04g55700.1 | LOC_Os12g40490.1 | 0.422 |
| 4337251 | 4324511 | LOC_Os04g56090.1 | LOC_Os01g05620.1 | 0.404 |
| 4337251 | 4324660 | LOC_Os04g56090.1 | LOC_Os01g52380.1 | 0.404 |
| 4337251 | 4333771 | LOC_Os04g56090.1 | LOC_Os03g48320.1 | 0.404 |
| 4337251 | 4334935 | LOC_Os04g56090.1 | LOC_Os04g02110.1 | 0.404 |
| 4337251 | 4335058 | LOC_Os04g56090.1 | LOC_Os04g08740.1 | 0.415 |
| 4337251 | 4336120 | LOC_Os04g56090.1 | LOC_Os04g39460.1 | 0.404 |
| 4337251 | 4338096 | LOC_Os04g56090.1 | LOC_Os05g11990.1 | 0.703 |
| 4337251 | 4338531 | LOC_Os04g56090.1 | LOC_Os05g29030.1 | 0.703 |
| 4337251 | 4339763 | LOC_Os04g56090.1 | LOC_Os05g50970.1 | 0.703 |
| 4337251 | 4340110 | LOC_Os04g56090.1 | LOC_Os06g05359.1 | 0.404 |
| 4337251 | 4340753 | LOC_Os04g56090.1 | LOC_Os06g16450.1 | 0.404 |
| 4337251 | 4341646 | LOC_Os04g56090.1 | LOC_Os06g43670.1 | 0.404 |
| 4337251 | 4342017 | LOC_Os04g56090.1 | LOC_Os06g49430.1 | 0.469 |
| 4337251 | 4343409 | LOC_Os04g56090.1 | LOC_Os07g33730.1 | 0.404 |
| 4337251 | 4344698 | LOC_Os04g56090.1 | LOC_Os08g06060.1 | 0.492 |
| 4337251 | 4345202 | LOC_Os04g56090.1 | LOC_Os08g20000.1 | 0.404 |
| 4337251 | 4345297 | LOC_Os04g56090.1 | LOC_Os08g24380.1 | 0.404 |
| 4337251 | 4345628 | LOC_Os04g56090.1 | LOC_Os08g33200.1 | 0.562 |
| 4337251 | 4347005 | LOC_Os04g56090.1 | LOC_Os09g23740.1 | 0.562 |
| 4337251 | 4347787 | LOC_Os04g56090.1 | LOC_Os09g37860.1 | 0.408 |
| 4337251 | 4350109 | LOC_Os04g56090.1 | LOC_Os11g11770.1 | 0.404 |
| 4337251 | 4350130 | LOC_Os04g56090.1 | LOC_Os11g12300.1 | 0.404 |
| 4337251 | 4350814 | LOC_Os04g56090.1 | LOC_Os11g37860.1 | 0.404 |
| 4337251 | 4350954 | LOC_Os04g56090.1 | LOC_Os11g40780.1 | 0.404 |
| 4337251 | 4350996 | LOC_Os04g56090.1 | LOC_Os11g42040.1 | 0.404 |
| 4337251 | 4351038 | LOC_Os04g56090.1 | LOC_Os11g43250.1 | 0.404 |
| 4337251 | 4351041 | LOC_Os04g56090.1 | LOC_Os11g43390.1 | 0.404 |
| 4337251 | 4351749 | LOC_Os04g56090.1 | LOC_Os12g10410.1 | 0.404 |
| 4337251 | 4352130 | LOC_Os04g56090.1 | LOC_Os12g25170.1 | 0.404 |
| 4337251 | 4352270 | LOC_Os04g56090.1 | LOC_Os12g31620.1 | 0.404 |
| 4337251 | IRL7 | LOC_Os04g56090.1 | LOC_Os03g11360.2 | 0.404 |
| 4337251 | LOC_Os02g49270.1 | LOC_Os04g56090.1 | LOC_Os02g49270.1 | 0.408 |
| 4337257 | 4326546 | LOC_Os04g56160.1 | LOC_Os01g53920.1 | 0.588 |
| 4337257 | 4338096 | LOC_Os04g56160.1 | LOC_Os05g11990.1 | 0.516 |
| 4337257 | 4341965 | LOC_Os04g56160.1 | LOC_Os06g48720.1 | 0.745 |
| 4337257 | 4342017 | LOC_Os04g56160.1 | LOC_Os06g49430.1 | 0.412 |
| 4337257 | 4345065 | LOC_Os04g56160.1 | LOC_Os08g14990.1 | 0.588 |
| 4337257 | 4345708 | LOC_Os04g56160.1 | LOC_Os08g34650.1 | 0.588 |
| 4337257 | 4347095 | LOC_Os04g56160.1 | LOC_Os09g25540.1 | 0.588 |
| 4337317 | 4326270 | LOC_Os04g56980.1 | LOC_Os01g16414.1 | 0.603 |
| 4337317 | 4335089 | LOC_Os04g56980.1 | LOC_Os04g09860.1 | 0.603 |
| 4337317 | 4343299 | LOC_Os04g56980.1 | LOC_Os07g31450.1 | 0.531 |
| 4337317 | 4348805 | LOC_Os04g56980.1 | LOC_Os10g31970.1 | 0.529 |
| 4337356 | 4337658 | LOC_Os04g57520.1 | LOC_Os05g03100.1 | 0.488 |
| 4337356 | 4347401 | LOC_Os04g57520.1 | LOC_Os09g30411.1 | 0.524 |
| 4337356 | 4348731 | LOC_Os04g57520.1 | LOC_Os10g30580.1 | 0.929 |
| 4337360 | 4324980 | LOC_Os04g57560.1 | LOC_Os01g67970.1 | 0.565 |
| 4337360 | 4344410 | LOC_Os04g57560.1 | LOC_Os08g01054.1 | 0.436 |
| 4337360 | 4344819 | LOC_Os04g57560.1 | LOC_Os08g08210.1 | 0.433 |
| 4337435 | 4327051 | LOC_Os04g58560.1 | LOC_Os01g57720.1 | 0.691 |
| 4337533 | 4339099 | LOC_Os05g01230.1 | LOC_Os05g39850.1 | 0.457 |
| 4337533 | 4340849 | LOC_Os05g01230.1 | LOC_Os06g20370.1 | 0.424 |
| 4337533 | 4343388 | LOC_Os05g01230.1 | LOC_Os07g33370.1 | 0.487 |
| 4337586 | 4327967 | LOC_Os05g01940.1 | LOC_Os01g58780.1 | 0.9 |
| 4337586 | 4327983 | LOC_Os05g01940.1 | LOC_Os02g01170.1 | 0.908 |
| 4337586 | 4344172 | LOC_Os05g01940.1 | LOC_Os07g46560.1 | 0.902 |
| 4337619 | 4326546 | LOC_Os05g02500.1 | LOC_Os01g53920.1 | 0.817 |
| 4337619 | 4328135 | LOC_Os05g02500.1 | LOC_Os02g03060.1 | 0.496 |
| 4337619 | 4330866 | LOC_Os05g02500.1 | LOC_Os02g53120.1 | 0.422 |
| 4337619 | 4342017 | LOC_Os05g02500.1 | LOC_Os06g49430.1 | 0.873 |
| 4337619 | 4344698 | LOC_Os05g02500.1 | LOC_Os08g06060.1 | 0.873 |
| 4337619 | 4345065 | LOC_Os05g02500.1 | LOC_Os08g14990.1 | 0.817 |
| 4337619 | 4345628 | LOC_Os05g02500.1 | LOC_Os08g33200.1 | 0.936 |
| 4337619 | 4345708 | LOC_Os05g02500.1 | LOC_Os08g34650.1 | 0.817 |
| 4337619 | 4347005 | LOC_Os05g02500.1 | LOC_Os09g23740.1 | 0.936 |
| 4337619 | 4347095 | LOC_Os05g02500.1 | LOC_Os09g25540.1 | 0.817 |
| 4337645 | 4338096 | LOC_Os05g02890.1 | LOC_Os05g11990.1 | 0.478 |
| 4337645 | 4341281 | LOC_Os05g02890.1 | LOC_Os06g36090.1 | 0.638 |
| 4337645 | 4343299 | LOC_Os05g02890.1 | LOC_Os07g31450.1 | 0.466 |
| 4337658 | 4326546 | LOC_Os05g03100.1 | LOC_Os01g53920.1 | 0.655 |
| 4337658 | 4327022 | LOC_Os05g03100.1 | LOC_Os01g55610.1 | 0.495 |
| 4337658 | 4327983 | LOC_Os05g03100.1 | LOC_Os02g01170.1 | 0.422 |
| 4337658 | 4328632 | LOC_Os05g03100.1 | LOC_Os02g10510.1 | 0.431 |
| 4337658 | 4336851 | LOC_Os05g03100.1 | LOC_Os04g50930.1 | 0.495 |
| 4337658 | 4337356 | LOC_Os05g03100.1 | LOC_Os04g57520.1 | 0.488 |
| 4337658 | 4345065 | LOC_Os05g03100.1 | LOC_Os08g14990.1 | 0.655 |
| 4337658 | 4345708 | LOC_Os05g03100.1 | LOC_Os08g34650.1 | 0.655 |
| 4337658 | 4346109 | LOC_Os05g03100.1 | LOC_Os08g41630.1 | 0.521 |
| 4337658 | 4347095 | LOC_Os05g03100.1 | LOC_Os09g25540.1 | 0.655 |
| 4337658 | 4347401 | LOC_Os05g03100.1 | LOC_Os09g30411.1 | 0.506 |
| 4337658 | 4348731 | LOC_Os05g03100.1 | LOC_Os10g30580.1 | 0.588 |
| 4337658 | LOC_Os03g64080.1 | LOC_Os05g03100.1 | LOC_Os03g64080.1 | 0.424 |
| 4337686 | 4326546 | LOC_Os05g03610.1 | LOC_Os01g53920.1 | 0.726 |
| 4337686 | 4345065 | LOC_Os05g03610.1 | LOC_Os08g14990.1 | 0.726 |
| 4337686 | 4345628 | LOC_Os05g03610.1 | LOC_Os08g33200.1 | 0.946 |
| 4337686 | 4345708 | LOC_Os05g03610.1 | LOC_Os08g34650.1 | 0.726 |
| 4337686 | 4347005 | LOC_Os05g03610.1 | LOC_Os09g23740.1 | 0.946 |
| 4337686 | 4347095 | LOC_Os05g03610.1 | LOC_Os09g25540.1 | 0.726 |
| 4337886 | 4330866 | LOC_Os05g06840.1 | LOC_Os02g53120.1 | 0.405 |
| 4337886 | 4330902 | LOC_Os05g06840.1 | LOC_Os02g53680.1 | 0.51 |
| 4337886 | 4332190 | LOC_Os05g06840.1 | LOC_Os03g13460.1 | 0.585 |
| 4337886 | 4339099 | LOC_Os05g06840.1 | LOC_Os05g39850.1 | 0.867 |
| 4337886 | 4340849 | LOC_Os05g06840.1 | LOC_Os06g20370.1 | 0.585 |
| 4337886 | 4341510 | LOC_Os05g06840.1 | LOC_Os06g40840.1 | 0.585 |
| 4337886 | 4343388 | LOC_Os05g06840.1 | LOC_Os07g33370.1 | 0.413 |
| 4337886 | 4347221 | LOC_Os05g06840.1 | LOC_Os09g27700.1 | 0.585 |
| 4337886 | 4351770 | LOC_Os05g06840.1 | LOC_Os12g10670.1 | 0.523 |
| 4338043 | 4342472 | LOC_Os05g10770.1 | LOC_Os07g06980.1 | 0.464 |
| 4338043 | 4344819 | LOC_Os05g10770.1 | LOC_Os08g08210.1 | 0.678 |
| 4338080 | 4324511 | LOC_Os05g11750.1 | LOC_Os01g05620.1 | 0.404 |
| 4338080 | 4324660 | LOC_Os05g11750.1 | LOC_Os01g52380.1 | 0.404 |
| 4338080 | 4333771 | LOC_Os05g11750.1 | LOC_Os03g48320.1 | 0.404 |
| 4338080 | 4334935 | LOC_Os05g11750.1 | LOC_Os04g02110.1 | 0.404 |
| 4338080 | 4335058 | LOC_Os05g11750.1 | LOC_Os04g08740.1 | 0.415 |
| 4338080 | 4336120 | LOC_Os05g11750.1 | LOC_Os04g39460.1 | 0.404 |
| 4338080 | 4338096 | LOC_Os05g11750.1 | LOC_Os05g11990.1 | 0.703 |
| 4338080 | 4338531 | LOC_Os05g11750.1 | LOC_Os05g29030.1 | 0.724 |
| 4338080 | 4339763 | LOC_Os05g11750.1 | LOC_Os05g50970.1 | 0.724 |
| 4338080 | 4340110 | LOC_Os05g11750.1 | LOC_Os06g05359.1 | 0.404 |
| 4338080 | 4340753 | LOC_Os05g11750.1 | LOC_Os06g16450.1 | 0.404 |
| 4338080 | 4341646 | LOC_Os05g11750.1 | LOC_Os06g43670.1 | 0.404 |
| 4338080 | 4342017 | LOC_Os05g11750.1 | LOC_Os06g49430.1 | 0.468 |
| 4338080 | 4343409 | LOC_Os05g11750.1 | LOC_Os07g33730.1 | 0.404 |
| 4338080 | 4344698 | LOC_Os05g11750.1 | LOC_Os08g06060.1 | 0.492 |
| 4338080 | 4345202 | LOC_Os05g11750.1 | LOC_Os08g20000.1 | 0.404 |
| 4338080 | 4345297 | LOC_Os05g11750.1 | LOC_Os08g24380.1 | 0.404 |
| 4338080 | 4345628 | LOC_Os05g11750.1 | LOC_Os08g33200.1 | 0.562 |
| 4338080 | 4347005 | LOC_Os05g11750.1 | LOC_Os09g23740.1 | 0.562 |
| 4338080 | 4347787 | LOC_Os05g11750.1 | LOC_Os09g37860.1 | 0.443 |
| 4338080 | 4349742 | LOC_Os05g11750.1 | LOC_Os11g04600.1 | 0.423 |
| 4338080 | 4350109 | LOC_Os05g11750.1 | LOC_Os11g11770.1 | 0.404 |
| 4338080 | 4350130 | LOC_Os05g11750.1 | LOC_Os11g12300.1 | 0.404 |
| 4338080 | 4350814 | LOC_Os05g11750.1 | LOC_Os11g37860.1 | 0.404 |
| 4338080 | 4350954 | LOC_Os05g11750.1 | LOC_Os11g40780.1 | 0.404 |
| 4338080 | 4350996 | LOC_Os05g11750.1 | LOC_Os11g42040.1 | 0.404 |
| 4338080 | 4351038 | LOC_Os05g11750.1 | LOC_Os11g43250.1 | 0.404 |
| 4338080 | 4351041 | LOC_Os05g11750.1 | LOC_Os11g43390.1 | 0.404 |
| 4338080 | 4351457 | LOC_Os05g11750.1 | LOC_Os12g04410.1 | 0.423 |
| 4338080 | 4351749 | LOC_Os05g11750.1 | LOC_Os12g10410.1 | 0.404 |
| 4338080 | 4352130 | LOC_Os05g11750.1 | LOC_Os12g25170.1 | 0.404 |
| 4338080 | 4352270 | LOC_Os05g11750.1 | LOC_Os12g31620.1 | 0.404 |
| 4338080 | IRL7 | LOC_Os05g11750.1 | LOC_Os03g11360.2 | 0.404 |
| 4338080 | LOC_Os02g49270.1 | LOC_Os05g11750.1 | LOC_Os02g49270.1 | 0.46 |
| 4338096 | 4324082 | LOC_Os05g11990.1 | LOC_Os01g06320.1 | 0.42 |
| 4338096 | 4324364 | LOC_Os05g11990.1 | LOC_Os01g69030.2 | 0.671 |
| 4338096 | 4324511 | LOC_Os05g11990.1 | LOC_Os01g05620.1 | 0.628 |
| 4338096 | 4324660 | LOC_Os05g11990.1 | LOC_Os01g52380.1 | 0.628 |
| 4338096 | 4324980 | LOC_Os05g11990.1 | LOC_Os01g67970.1 | 0.666 |
| 4338096 | 4325022 | LOC_Os05g11990.1 | LOC_Os01g67770.1 | 0.478 |
| 4338096 | 4326270 | LOC_Os05g11990.1 | LOC_Os01g16414.1 | 0.582 |
| 4338096 | 4326546 | LOC_Os05g11990.1 | LOC_Os01g53920.1 | 0.968 |
| 4338096 | 4327667 | LOC_Os05g11990.1 | LOC_Os01g40590.1 | 0.703 |
| 4338096 | 4327790 | LOC_Os05g11990.1 | LOC_Os01g13570.1 | 0.509 |
| 4338096 | 4328135 | LOC_Os05g11990.1 | LOC_Os02g03060.1 | 0.598 |
| 4338096 | 4328633 | LOC_Os05g11990.1 | LOC_Os02g10520.1 | 0.447 |
| 4338096 | 4328702 | LOC_Os05g11990.1 | LOC_Os02g11960.1 | 0.477 |
| 4338096 | 4328725 | LOC_Os05g11990.1 | LOC_Os02g12440.1 | 0.703 |
| 4338096 | 4329438 | LOC_Os05g11990.1 | LOC_Os02g30310.1 | 0.466 |
| 4338096 | 4329448 | LOC_Os05g11990.1 | LOC_Os02g30620.1 | 0.972 |
| 4338096 | 4330701 | LOC_Os05g11990.1 | LOC_Os02g50970.1 | 0.703 |
| 4338096 | 4331134 | LOC_Os05g11990.1 | LOC_Os02g57080.1 | 0.703 |
| 4338096 | 4331991 | LOC_Os05g11990.1 | LOC_Os03g10780.1 | 0.451 |
| 4338096 | 4332080 | LOC_Os05g11990.1 | LOC_Os03g11910.1 | 0.928 |
| 4338096 | 4332258 | LOC_Os05g11990.1 | LOC_Os03g14540.1 | 0.491 |
| 4338096 | 4333060 | LOC_Os05g11990.1 | LOC_Os03g28300.1 | 0.703 |
| 4338096 | 4333771 | LOC_Os05g11990.1 | LOC_Os03g48320.1 | 0.628 |
| 4338096 | 4334290 | LOC_Os05g11990.1 | LOC_Os03g56460.1 | 0.432 |
| 4338096 | 4334921 | LOC_Os05g11990.1 | LOC_Os04g01874.1 | 0.703 |
| 4338096 | 4334935 | LOC_Os05g11990.1 | LOC_Os04g02110.1 | 0.628 |
| 4338096 | 4335058 | LOC_Os05g11990.1 | LOC_Os04g08740.1 | 0.528 |
| 4338096 | 4335089 | LOC_Os05g11990.1 | LOC_Os04g09860.1 | 0.48 |
| 4338096 | 4335824 | LOC_Os05g11990.1 | LOC_Os04g34250.1 | 0.703 |
| 4338096 | 4336120 | LOC_Os05g11990.1 | LOC_Os04g39460.1 | 0.628 |
| 4338096 | 4336977 | LOC_Os05g11990.1 | LOC_Os04g52370.1 | 0.567 |
| 4338096 | 4337251 | LOC_Os05g11990.1 | LOC_Os04g56090.1 | 0.703 |
| 4338096 | 4337257 | LOC_Os05g11990.1 | LOC_Os04g56160.1 | 0.516 |
| 4338096 | 4337645 | LOC_Os05g11990.1 | LOC_Os05g02890.1 | 0.478 |
| 4338096 | 4338080 | LOC_Os05g11990.1 | LOC_Os05g11750.1 | 0.703 |
| 4338096 | 4338531 | LOC_Os05g11990.1 | LOC_Os05g29030.1 | 0.654 |
| 4338096 | 4339142 | LOC_Os05g11990.1 | LOC_Os05g40770.1 | 0.703 |
| 4338096 | 4339763 | LOC_Os05g11990.1 | LOC_Os05g50970.1 | 0.654 |
| 4338096 | 4340110 | LOC_Os05g11990.1 | LOC_Os06g05359.1 | 0.628 |
| 4338096 | 4340753 | LOC_Os05g11990.1 | LOC_Os06g16450.1 | 0.628 |
| 4338096 | 4341066 | LOC_Os05g11990.1 | LOC_Os06g29080.1 | 0.703 |
| 4338096 | 4341219 | LOC_Os05g11990.1 | LOC_Os06g34690.1 | 0.661 |
| 4338096 | 4341568 | LOC_Os05g11990.1 | LOC_Os06g41980.1 | 0.703 |
| 4338096 | 4341646 | LOC_Os05g11990.1 | LOC_Os06g43670.1 | 0.628 |
| 4338096 | 4341853 | LOC_Os05g11990.1 | LOC_Os06g46600.1 | 0.928 |
| 4338096 | 4341947 | LOC_Os05g11990.1 | LOC_Os06g48240.1 | 0.401 |
| 4338096 | 4341965 | LOC_Os05g11990.1 | LOC_Os06g48720.1 | 0.483 |
| 4338096 | 4342017 | LOC_Os05g11990.1 | LOC_Os06g49430.1 | 0.439 |
| 4338096 | 4342049 | LOC_Os05g11990.1 | LOC_Os06g49870.1 | 0.478 |
| 4338096 | 4342396 | LOC_Os05g11990.1 | LOC_Os07g05370.1 | 0.703 |
| 4338096 | 4342410 | LOC_Os05g11990.1 | LOC_Os07g05620.1 | 0.708 |
| 4338096 | 4342472 | LOC_Os05g11990.1 | LOC_Os07g06980.1 | 0.476 |
| 4338096 | 4342673 | LOC_Os05g11990.1 | LOC_Os07g10350.1 | 0.457 |
| 4338096 | 4342694 | LOC_Os05g11990.1 | LOC_Os07g10630.1 | 0.628 |
| 4338096 | 4343196 | LOC_Os05g11990.1 | LOC_Os07g28800.1 | 0.972 |
| 4338096 | 4343409 | LOC_Os05g11990.1 | LOC_Os07g33730.1 | 0.628 |
| 4338096 | 4343699 | LOC_Os05g11990.1 | LOC_Os07g38810.1 | 0.703 |
| 4338096 | 4343832 | LOC_Os05g11990.1 | LOC_Os07g41140.1 | 0.703 |
| 4338096 | 4344584 | LOC_Os05g11990.1 | LOC_Os08g03570.1 | 0.432 |
| 4338096 | 4344612 | LOC_Os05g11990.1 | LOC_Os08g04180.1 | 0.532 |
| 4338096 | 4344698 | LOC_Os05g11990.1 | LOC_Os08g06060.1 | 0.439 |
| 4338096 | 4344819 | LOC_Os05g11990.1 | LOC_Os08g08210.1 | 0.569 |
| 4338096 | 4345065 | LOC_Os05g11990.1 | LOC_Os08g14990.1 | 0.968 |
| 4338096 | 4345202 | LOC_Os05g11990.1 | LOC_Os08g20000.1 | 0.628 |
| 4338096 | 4345297 | LOC_Os05g11990.1 | LOC_Os08g24380.1 | 0.628 |
| 4338096 | 4345628 | LOC_Os05g11990.1 | LOC_Os08g33200.1 | 0.677 |
| 4338096 | 4345708 | LOC_Os05g11990.1 | LOC_Os08g34650.1 | 0.968 |
| 4338096 | 4345910 | LOC_Os05g11990.1 | LOC_Os08g38410.1 | 0.726 |
| 4338096 | 4346276 | LOC_Os05g11990.1 | LOC_Os08g44050.1 | 0.474 |
| 4338096 | 4347005 | LOC_Os05g11990.1 | LOC_Os09g23740.1 | 0.677 |
| 4338096 | 4347095 | LOC_Os05g11990.1 | LOC_Os09g25540.1 | 0.968 |
| 4338096 | 4347172 | LOC_Os05g11990.1 | LOC_Os09g26880.1 | 0.501 |
| 4338096 | 4347311 | LOC_Os05g11990.1 | LOC_Os09g29070.1 | 0.432 |
| 4338096 | 4347787 | LOC_Os05g11990.1 | LOC_Os09g37860.1 | 0.4 |
| 4338096 | 4348561 | LOC_Os05g11990.1 | LOC_Os10g26010.1 | 0.485 |
| 4338096 | 4348731 | LOC_Os05g11990.1 | LOC_Os10g30580.1 | 0.686 |
| 4338096 | 4348853 | LOC_Os05g11990.1 | LOC_Os10g32980.1 | 0.558 |
| 4338096 | 4349742 | LOC_Os05g11990.1 | LOC_Os11g04600.1 | 0.79 |
| 4338096 | 4350049 | LOC_Os05g11990.1 | LOC_Os11g10430.1 | 0.56 |
| 4338096 | 4350109 | LOC_Os05g11990.1 | LOC_Os11g11770.1 | 0.628 |
| 4338096 | 4350130 | LOC_Os05g11990.1 | LOC_Os11g12300.1 | 0.628 |
| 4338096 | 4350814 | LOC_Os05g11990.1 | LOC_Os11g37860.1 | 0.628 |
| 4338096 | 4350837 | LOC_Os05g11990.1 | LOC_Os11g38170.1 | 0.571 |
| 4338096 | 4350954 | LOC_Os05g11990.1 | LOC_Os11g40780.1 | 0.628 |
| 4338096 | 4350996 | LOC_Os05g11990.1 | LOC_Os11g42040.1 | 0.628 |
| 4338096 | 4351008 | LOC_Os05g11990.1 | LOC_Os11g42350.1 | 0.459 |
| 4338096 | 4351038 | LOC_Os05g11990.1 | LOC_Os11g43250.1 | 0.628 |
| 4338096 | 4351041 | LOC_Os05g11990.1 | LOC_Os11g43390.1 | 0.628 |
| 4338096 | 4351457 | LOC_Os05g11990.1 | LOC_Os12g04410.1 | 0.79 |
| 4338096 | 4351749 | LOC_Os05g11990.1 | LOC_Os12g10410.1 | 0.628 |
| 4338096 | 4351847 | LOC_Os05g11990.1 | LOC_Os12g13320.1 | 0.663 |
| 4338096 | 4352130 | LOC_Os05g11990.1 | LOC_Os12g25170.1 | 0.628 |
| 4338096 | 4352270 | LOC_Os05g11990.1 | LOC_Os12g31620.1 | 0.628 |
| 4338096 | 4352691 | LOC_Os05g11990.1 | LOC_Os12g40419.1 | 0.703 |
| 4338096 | 4352904 | LOC_Os05g11990.1 | LOC_Os12g44090.1 | 0.703 |
| 4338096 | CRSH3 | LOC_Os05g11990.1 | LOC_Os05g06940.1 | 0.539 |
| 4338096 | IRL7 | LOC_Os05g11990.1 | LOC_Os03g11360.2 | 0.628 |
| 4338096 | LOC_Os02g49270.1 | LOC_Os05g11990.1 | LOC_Os02g49270.1 | 0.4 |
| 4338096 | LOC_Os10g11340.1 | LOC_Os05g11990.1 | LOC_Os10g11340.1 | 0.427 |
| 4338309 | 4339356 | LOC_Os05g22800.1 | LOC_Os05g44600.1 | 0.817 |
| 4338309 | 4344268 | LOC_Os05g22800.1 | LOC_Os07g47830.1 | 0.824 |
| 4338481 | 4326546 | LOC_Os05g27880.1 | LOC_Os01g53920.1 | 0.661 |
| 4338481 | 4345065 | LOC_Os05g27880.1 | LOC_Os08g14990.1 | 0.655 |
| 4338481 | 4345708 | LOC_Os05g27880.1 | LOC_Os08g34650.1 | 0.655 |
| 4338481 | 4347095 | LOC_Os05g27880.1 | LOC_Os09g25540.1 | 0.655 |
| 4338481 | LOC_Os08g43250.1 | LOC_Os05g27880.1 | LOC_Os08g43250.1 | 0.609 |
| 4338531 | 4324584 | LOC_Os05g29030.1 | LOC_Os01g11920.1 | 0.516 |
| 4338531 | 4326546 | LOC_Os05g29030.1 | LOC_Os01g53920.1 | 0.963 |
| 4338531 | 4327667 | LOC_Os05g29030.1 | LOC_Os01g40590.1 | 0.724 |
| 4338531 | 4328725 | LOC_Os05g29030.1 | LOC_Os02g12440.1 | 0.703 |
| 4338531 | 4330701 | LOC_Os05g29030.1 | LOC_Os02g50970.1 | 0.78 |
| 4338531 | 4331134 | LOC_Os05g29030.1 | LOC_Os02g57080.1 | 0.724 |
| 4338531 | 4333060 | LOC_Os05g29030.1 | LOC_Os03g28300.1 | 0.78 |
| 4338531 | 4334214 | LOC_Os05g29030.1 | LOC_Os03g55560.1 | 0.593 |
| 4338531 | 4334921 | LOC_Os05g29030.1 | LOC_Os04g01874.1 | 0.703 |
| 4338531 | 4335824 | LOC_Os05g29030.1 | LOC_Os04g34250.1 | 0.703 |
| 4338531 | 4337251 | LOC_Os05g29030.1 | LOC_Os04g56090.1 | 0.703 |
| 4338531 | 4338080 | LOC_Os05g29030.1 | LOC_Os05g11750.1 | 0.724 |
| 4338531 | 4338096 | LOC_Os05g29030.1 | LOC_Os05g11990.1 | 0.654 |
| 4338531 | 4339142 | LOC_Os05g29030.1 | LOC_Os05g40770.1 | 0.703 |
| 4338531 | 4341066 | LOC_Os05g29030.1 | LOC_Os06g29080.1 | 0.724 |
| 4338531 | 4341568 | LOC_Os05g29030.1 | LOC_Os06g41980.1 | 0.703 |
| 4338531 | 4342017 | LOC_Os05g29030.1 | LOC_Os06g49430.1 | 0.771 |
| 4338531 | 4342396 | LOC_Os05g29030.1 | LOC_Os07g05370.1 | 0.724 |
| 4338531 | 4342410 | LOC_Os05g29030.1 | LOC_Os07g05620.1 | 0.738 |
| 4338531 | 4343699 | LOC_Os05g29030.1 | LOC_Os07g38810.1 | 0.703 |
| 4338531 | 4343832 | LOC_Os05g29030.1 | LOC_Os07g41140.1 | 0.703 |
| 4338531 | 4344698 | LOC_Os05g29030.1 | LOC_Os08g06060.1 | 0.771 |
| 4338531 | 4345065 | LOC_Os05g29030.1 | LOC_Os08g14990.1 | 0.963 |
| 4338531 | 4345708 | LOC_Os05g29030.1 | LOC_Os08g34650.1 | 0.963 |
| 4338531 | 4347095 | LOC_Os05g29030.1 | LOC_Os09g25540.1 | 0.963 |
| 4338531 | 4347787 | LOC_Os05g29030.1 | LOC_Os09g37860.1 | 0.404 |
| 4338531 | 4352691 | LOC_Os05g29030.1 | LOC_Os12g40419.1 | 0.724 |
| 4338531 | 4352904 | LOC_Os05g29030.1 | LOC_Os12g44090.1 | 0.703 |
| 4338531 | LOC_Os02g49270.1 | LOC_Os05g29030.1 | LOC_Os02g49270.1 | 0.404 |
| 4338689 | 4326378 | LOC_Os05g32600.1 | LOC_Os01g40980.1 | 0.899 |
| 4338689 | 4328135 | LOC_Os05g32600.1 | LOC_Os02g03060.1 | 0.934 |
| 4338689 | 4331991 | LOC_Os05g32600.1 | LOC_Os03g10780.1 | 0.617 |
| 4339099 | 4328135 | LOC_Os05g39850.1 | LOC_Os02g03060.1 | 0.964 |
| 4339099 | 4330866 | LOC_Os05g39850.1 | LOC_Os02g53120.1 | 0.72 |
| 4339099 | 4330902 | LOC_Os05g39850.1 | LOC_Os02g53680.1 | 0.748 |
| 4339099 | 4337533 | LOC_Os05g39850.1 | LOC_Os05g01230.1 | 0.457 |
| 4339099 | 4337886 | LOC_Os05g39850.1 | LOC_Os05g06840.1 | 0.867 |
| 4339099 | 4340849 | LOC_Os05g39850.1 | LOC_Os06g20370.1 | 0.504 |
| 4339099 | 4341655 | LOC_Os05g39850.1 | LOC_Os06g43790.1 | 0.443 |
| 4339099 | 4343388 | LOC_Os05g39850.1 | LOC_Os07g33370.1 | 0.525 |
| 4339099 | 4344306 | LOC_Os05g39850.1 | LOC_Os07g48360.1 | 0.472 |
| 4339099 | 4347823 | LOC_Os05g39850.1 | LOC_Os09g38450.1 | 0.484 |
| 4339099 | 4351770 | LOC_Os05g39850.1 | LOC_Os12g10670.1 | 0.939 |
| 4339099 | LOC_Os03g64080.1 | LOC_Os05g39850.1 | LOC_Os03g64080.1 | 0.479 |
| 4339142 | 4324511 | LOC_Os05g40770.1 | LOC_Os01g05620.1 | 0.404 |
| 4339142 | 4324660 | LOC_Os05g40770.1 | LOC_Os01g52380.1 | 0.404 |
| 4339142 | 4333771 | LOC_Os05g40770.1 | LOC_Os03g48320.1 | 0.404 |
| 4339142 | 4334214 | LOC_Os05g40770.1 | LOC_Os03g55560.1 | 0.438 |
| 4339142 | 4334935 | LOC_Os05g40770.1 | LOC_Os04g02110.1 | 0.404 |
| 4339142 | 4335058 | LOC_Os05g40770.1 | LOC_Os04g08740.1 | 0.461 |
| 4339142 | 4336120 | LOC_Os05g40770.1 | LOC_Os04g39460.1 | 0.404 |
| 4339142 | 4338096 | LOC_Os05g40770.1 | LOC_Os05g11990.1 | 0.703 |
| 4339142 | 4338531 | LOC_Os05g40770.1 | LOC_Os05g29030.1 | 0.703 |
| 4339142 | 4339763 | LOC_Os05g40770.1 | LOC_Os05g50970.1 | 0.703 |
| 4339142 | 4340110 | LOC_Os05g40770.1 | LOC_Os06g05359.1 | 0.404 |
| 4339142 | 4340753 | LOC_Os05g40770.1 | LOC_Os06g16450.1 | 0.404 |
| 4339142 | 4341646 | LOC_Os05g40770.1 | LOC_Os06g43670.1 | 0.404 |
| 4339142 | 4342017 | LOC_Os05g40770.1 | LOC_Os06g49430.1 | 0.469 |
| 4339142 | 4343409 | LOC_Os05g40770.1 | LOC_Os07g33730.1 | 0.404 |
| 4339142 | 4344698 | LOC_Os05g40770.1 | LOC_Os08g06060.1 | 0.468 |
| 4339142 | 4345202 | LOC_Os05g40770.1 | LOC_Os08g20000.1 | 0.404 |
| 4339142 | 4345297 | LOC_Os05g40770.1 | LOC_Os08g24380.1 | 0.404 |
| 4339142 | 4345628 | LOC_Os05g40770.1 | LOC_Os08g33200.1 | 0.562 |
| 4339142 | 4347005 | LOC_Os05g40770.1 | LOC_Os09g23740.1 | 0.562 |
| 4339142 | 4347787 | LOC_Os05g40770.1 | LOC_Os09g37860.1 | 0.408 |
| 4339142 | 4350109 | LOC_Os05g40770.1 | LOC_Os11g11770.1 | 0.404 |
| 4339142 | 4350130 | LOC_Os05g40770.1 | LOC_Os11g12300.1 | 0.404 |
| 4339142 | 4350814 | LOC_Os05g40770.1 | LOC_Os11g37860.1 | 0.404 |
| 4339142 | 4350954 | LOC_Os05g40770.1 | LOC_Os11g40780.1 | 0.404 |
| 4339142 | 4350996 | LOC_Os05g40770.1 | LOC_Os11g42040.1 | 0.404 |
| 4339142 | 4351038 | LOC_Os05g40770.1 | LOC_Os11g43250.1 | 0.404 |
| 4339142 | 4351041 | LOC_Os05g40770.1 | LOC_Os11g43390.1 | 0.404 |
| 4339142 | 4351749 | LOC_Os05g40770.1 | LOC_Os12g10410.1 | 0.404 |
| 4339142 | 4352130 | LOC_Os05g40770.1 | LOC_Os12g25170.1 | 0.404 |
| 4339142 | 4352270 | LOC_Os05g40770.1 | LOC_Os12g31620.1 | 0.404 |
| 4339142 | LOC_Os02g49270.1 | LOC_Os05g40770.1 | LOC_Os02g49270.1 | 0.408 |
| 4339356 | 4338309 | LOC_Os05g44600.1 | LOC_Os05g22800.1 | 0.817 |
| 4339379 | 4323903 | LOC_Os05g45020.1 | LOC_Os01g19820.1 | 0.458 |
| 4339523 | 4332492 | LOC_Os05g47660.1 | LOC_Os03g17940.1 | 0.408 |
| 4339523 | 4340373 | LOC_Os05g47660.1 | LOC_Os06g09240.1 | 0.411 |
| 4339523 | 4341683 | LOC_Os05g47660.1 | LOC_Os06g44060.1 | 0.403 |
| 4339523 | 4342770 | LOC_Os05g47660.1 | LOC_Os07g12640.1 | 0.401 |
| 4339523 | 4343352 | LOC_Os05g47660.1 | LOC_Os07g32620.1 | 0.411 |
| 4339763 | 4324584 | LOC_Os05g50970.1 | LOC_Os01g11920.1 | 0.516 |
| 4339763 | 4326546 | LOC_Os05g50970.1 | LOC_Os01g53920.1 | 0.963 |
| 4339763 | 4327667 | LOC_Os05g50970.1 | LOC_Os01g40590.1 | 0.724 |
| 4339763 | 4328725 | LOC_Os05g50970.1 | LOC_Os02g12440.1 | 0.703 |
| 4339763 | 4330701 | LOC_Os05g50970.1 | LOC_Os02g50970.1 | 0.78 |
| 4339763 | 4331134 | LOC_Os05g50970.1 | LOC_Os02g57080.1 | 0.724 |
| 4339763 | 4333060 | LOC_Os05g50970.1 | LOC_Os03g28300.1 | 0.78 |
| 4339763 | 4334214 | LOC_Os05g50970.1 | LOC_Os03g55560.1 | 0.593 |
| 4339763 | 4334921 | LOC_Os05g50970.1 | LOC_Os04g01874.1 | 0.703 |
| 4339763 | 4335824 | LOC_Os05g50970.1 | LOC_Os04g34250.1 | 0.703 |
| 4339763 | 4337251 | LOC_Os05g50970.1 | LOC_Os04g56090.1 | 0.703 |
| 4339763 | 4338080 | LOC_Os05g50970.1 | LOC_Os05g11750.1 | 0.724 |
| 4339763 | 4338096 | LOC_Os05g50970.1 | LOC_Os05g11990.1 | 0.654 |
| 4339763 | 4339142 | LOC_Os05g50970.1 | LOC_Os05g40770.1 | 0.703 |
| 4339763 | 4341066 | LOC_Os05g50970.1 | LOC_Os06g29080.1 | 0.724 |
| 4339763 | 4341568 | LOC_Os05g50970.1 | LOC_Os06g41980.1 | 0.703 |
| 4339763 | 4342017 | LOC_Os05g50970.1 | LOC_Os06g49430.1 | 0.771 |
| 4339763 | 4342396 | LOC_Os05g50970.1 | LOC_Os07g05370.1 | 0.724 |
| 4339763 | 4342410 | LOC_Os05g50970.1 | LOC_Os07g05620.1 | 0.738 |
| 4339763 | 4343699 | LOC_Os05g50970.1 | LOC_Os07g38810.1 | 0.703 |
| 4339763 | 4343832 | LOC_Os05g50970.1 | LOC_Os07g41140.1 | 0.703 |
| 4339763 | 4344698 | LOC_Os05g50970.1 | LOC_Os08g06060.1 | 0.771 |
| 4339763 | 4345065 | LOC_Os05g50970.1 | LOC_Os08g14990.1 | 0.963 |
| 4339763 | 4345708 | LOC_Os05g50970.1 | LOC_Os08g34650.1 | 0.963 |
| 4339763 | 4347095 | LOC_Os05g50970.1 | LOC_Os09g25540.1 | 0.963 |
| 4339763 | 4347787 | LOC_Os05g50970.1 | LOC_Os09g37860.1 | 0.404 |
| 4339763 | 4352691 | LOC_Os05g50970.1 | LOC_Os12g40419.1 | 0.724 |
| 4339763 | 4352904 | LOC_Os05g50970.1 | LOC_Os12g44090.1 | 0.703 |
| 4339763 | LOC_Os02g49270.1 | LOC_Os05g50970.1 | LOC_Os02g49270.1 | 0.404 |
| 4339944 | 4324584 | LOC_Os06g02900.1 | LOC_Os01g11920.1 | 0.531 |
| 4339944 | 4326270 | LOC_Os06g02900.1 | LOC_Os01g16414.1 | 0.4 |
| 4339944 | 4350472 | LOC_Os06g02900.1 | LOC_Os11g27264.1 | 0.701 |
| 4339944 | 4350473 | LOC_Os06g02900.1 | LOC_Os11g27329.1 | 0.705 |
| 4339983 | 4331019 | LOC_Os06g03760.1 | LOC_Os02g55440.1 | 0.496 |
| 4340110 | 4326546 | LOC_Os06g05359.1 | LOC_Os01g53920.1 | 0.404 |
| 4340110 | 4327667 | LOC_Os06g05359.1 | LOC_Os01g40590.1 | 0.404 |
| 4340110 | 4328725 | LOC_Os06g05359.1 | LOC_Os02g12440.1 | 0.404 |
| 4340110 | 4330701 | LOC_Os06g05359.1 | LOC_Os02g50970.1 | 0.404 |
| 4340110 | 4331134 | LOC_Os06g05359.1 | LOC_Os02g57080.1 | 0.404 |
| 4340110 | 4333060 | LOC_Os06g05359.1 | LOC_Os03g28300.1 | 0.404 |
| 4340110 | 4334921 | LOC_Os06g05359.1 | LOC_Os04g01874.1 | 0.404 |
| 4340110 | 4335824 | LOC_Os06g05359.1 | LOC_Os04g34250.1 | 0.404 |
| 4340110 | 4337251 | LOC_Os06g05359.1 | LOC_Os04g56090.1 | 0.404 |
| 4340110 | 4338080 | LOC_Os06g05359.1 | LOC_Os05g11750.1 | 0.404 |
| 4340110 | 4338096 | LOC_Os06g05359.1 | LOC_Os05g11990.1 | 0.628 |
| 4340110 | 4339142 | LOC_Os06g05359.1 | LOC_Os05g40770.1 | 0.404 |
| 4340110 | 4341066 | LOC_Os06g05359.1 | LOC_Os06g29080.1 | 0.404 |
| 4340110 | 4341568 | LOC_Os06g05359.1 | LOC_Os06g41980.1 | 0.404 |
| 4340110 | 4342396 | LOC_Os06g05359.1 | LOC_Os07g05370.1 | 0.404 |
| 4340110 | 4342410 | LOC_Os06g05359.1 | LOC_Os07g05620.1 | 0.404 |
| 4340110 | 4343699 | LOC_Os06g05359.1 | LOC_Os07g38810.1 | 0.404 |
| 4340110 | 4343832 | LOC_Os06g05359.1 | LOC_Os07g41140.1 | 0.404 |
| 4340110 | 4345065 | LOC_Os06g05359.1 | LOC_Os08g14990.1 | 0.404 |
| 4340110 | 4345628 | LOC_Os06g05359.1 | LOC_Os08g33200.1 | 0.806 |
| 4340110 | 4345708 | LOC_Os06g05359.1 | LOC_Os08g34650.1 | 0.404 |
| 4340110 | 4347005 | LOC_Os06g05359.1 | LOC_Os09g23740.1 | 0.806 |
| 4340110 | 4347095 | LOC_Os06g05359.1 | LOC_Os09g25540.1 | 0.404 |
| 4340110 | 4352691 | LOC_Os06g05359.1 | LOC_Os12g40419.1 | 0.404 |
| 4340110 | 4352904 | LOC_Os06g05359.1 | LOC_Os12g44090.1 | 0.404 |
| 4340177 | 4346508 | LOC_Os06g06190.1 | LOC_Os09g07570.1 | 0.537 |
| 4340177 | CRSH3 | LOC_Os06g06190.1 | LOC_Os05g06940.1 | 0.711 |
| 4340373 | 4329422 | LOC_Os06g09240.1 | LOC_Os02g29960.1 | 0.436 |
| 4340373 | 4339523 | LOC_Os06g09240.1 | LOC_Os05g47660.1 | 0.411 |
| 4340373 | 4340915 | LOC_Os06g09240.1 | LOC_Os06g22340.1 | 0.436 |
| 4340373 | 4343395 | LOC_Os06g09240.1 | LOC_Os07g33480.1 | 0.436 |
| 4340373 | 4347809 | LOC_Os06g09240.1 | LOC_Os09g38239.1 | 0.408 |
| 4340373 | BGLU2 | LOC_Os06g09240.1 | LOC_Os01g59819.1 | 0.485 |
| 4340563 | 4332080 | LOC_Os06g12370.1 | LOC_Os03g11910.1 | 0.438 |
| 4340753 | 4326546 | LOC_Os06g16450.1 | LOC_Os01g53920.1 | 0.404 |
| 4340753 | 4327667 | LOC_Os06g16450.1 | LOC_Os01g40590.1 | 0.404 |
| 4340753 | 4328725 | LOC_Os06g16450.1 | LOC_Os02g12440.1 | 0.404 |
| 4340753 | 4330701 | LOC_Os06g16450.1 | LOC_Os02g50970.1 | 0.404 |
| 4340753 | 4331134 | LOC_Os06g16450.1 | LOC_Os02g57080.1 | 0.404 |
| 4340753 | 4333060 | LOC_Os06g16450.1 | LOC_Os03g28300.1 | 0.404 |
| 4340753 | 4334921 | LOC_Os06g16450.1 | LOC_Os04g01874.1 | 0.404 |
| 4340753 | 4335824 | LOC_Os06g16450.1 | LOC_Os04g34250.1 | 0.404 |
| 4340753 | 4337251 | LOC_Os06g16450.1 | LOC_Os04g56090.1 | 0.404 |
| 4340753 | 4338080 | LOC_Os06g16450.1 | LOC_Os05g11750.1 | 0.404 |
| 4340753 | 4338096 | LOC_Os06g16450.1 | LOC_Os05g11990.1 | 0.628 |
| 4340753 | 4339142 | LOC_Os06g16450.1 | LOC_Os05g40770.1 | 0.404 |
| 4340753 | 4341066 | LOC_Os06g16450.1 | LOC_Os06g29080.1 | 0.404 |
| 4340753 | 4341568 | LOC_Os06g16450.1 | LOC_Os06g41980.1 | 0.404 |
| 4340753 | 4342396 | LOC_Os06g16450.1 | LOC_Os07g05370.1 | 0.404 |
| 4340753 | 4342410 | LOC_Os06g16450.1 | LOC_Os07g05620.1 | 0.404 |
| 4340753 | 4343699 | LOC_Os06g16450.1 | LOC_Os07g38810.1 | 0.404 |
| 4340753 | 4343832 | LOC_Os06g16450.1 | LOC_Os07g41140.1 | 0.404 |
| 4340753 | 4345065 | LOC_Os06g16450.1 | LOC_Os08g14990.1 | 0.404 |
| 4340753 | 4345628 | LOC_Os06g16450.1 | LOC_Os08g33200.1 | 0.806 |
| 4340753 | 4345708 | LOC_Os06g16450.1 | LOC_Os08g34650.1 | 0.404 |
| 4340753 | 4347005 | LOC_Os06g16450.1 | LOC_Os09g23740.1 | 0.806 |
| 4340753 | 4347095 | LOC_Os06g16450.1 | LOC_Os09g25540.1 | 0.404 |
| 4340753 | 4352691 | LOC_Os06g16450.1 | LOC_Os12g40419.1 | 0.404 |
| 4340753 | 4352904 | LOC_Os06g16450.1 | LOC_Os12g44090.1 | 0.404 |
| 4340847 | 4326546 | LOC_Os06g20340.1 | LOC_Os01g53920.1 | 0.817 |
| 4340847 | 4328135 | LOC_Os06g20340.1 | LOC_Os02g03060.1 | 0.496 |
| 4340847 | 4330866 | LOC_Os06g20340.1 | LOC_Os02g53120.1 | 0.422 |
| 4340847 | 4342017 | LOC_Os06g20340.1 | LOC_Os06g49430.1 | 0.873 |
| 4340847 | 4344698 | LOC_Os06g20340.1 | LOC_Os08g06060.1 | 0.873 |
| 4340847 | 4345065 | LOC_Os06g20340.1 | LOC_Os08g14990.1 | 0.817 |
| 4340847 | 4345628 | LOC_Os06g20340.1 | LOC_Os08g33200.1 | 0.936 |
| 4340847 | 4345708 | LOC_Os06g20340.1 | LOC_Os08g34650.1 | 0.817 |
| 4340847 | 4347005 | LOC_Os06g20340.1 | LOC_Os09g23740.1 | 0.936 |
| 4340847 | 4347095 | LOC_Os06g20340.1 | LOC_Os09g25540.1 | 0.817 |
| 4340849 | 4327983 | LOC_Os06g20370.1 | LOC_Os02g01170.1 | 0.657 |
| 4340849 | 4328135 | LOC_Os06g20370.1 | LOC_Os02g03060.1 | 0.744 |
| 4340849 | 4330866 | LOC_Os06g20370.1 | LOC_Os02g53120.1 | 0.705 |
| 4340849 | 4337533 | LOC_Os06g20370.1 | LOC_Os05g01230.1 | 0.424 |
| 4340849 | 4337886 | LOC_Os06g20370.1 | LOC_Os05g06840.1 | 0.585 |
| 4340849 | 4339099 | LOC_Os06g20370.1 | LOC_Os05g39850.1 | 0.504 |
| 4340849 | 4343388 | LOC_Os06g20370.1 | LOC_Os07g33370.1 | 0.754 |
| 4340892 | 4333882 | LOC_Os06g21590.1 | LOC_Os03g50310.1 | 0.635 |
| 4340915 | 4326546 | LOC_Os06g22340.1 | LOC_Os01g53920.1 | 0.495 |
| 4340915 | 4340373 | LOC_Os06g22340.1 | LOC_Os06g09240.1 | 0.436 |
| 4340915 | 4343183 | LOC_Os06g22340.1 | LOC_Os07g28480.1 | 0.412 |
| 4340915 | 4343352 | LOC_Os06g22340.1 | LOC_Os07g32620.1 | 0.436 |
| 4340915 | 4343395 | LOC_Os06g22340.1 | LOC_Os07g33480.1 | 0.51 |
| 4340915 | 4345065 | LOC_Os06g22340.1 | LOC_Os08g14990.1 | 0.495 |
| 4340915 | 4345708 | LOC_Os06g22340.1 | LOC_Os08g34650.1 | 0.495 |
| 4340915 | 4347095 | LOC_Os06g22340.1 | LOC_Os09g25540.1 | 0.495 |
| 4340915 | 4347809 | LOC_Os06g22340.1 | LOC_Os09g38239.1 | 0.403 |
| 4340964 | 4326546 | LOC_Os06g23530.1 | LOC_Os01g53920.1 | 0.446 |
| 4340964 | 4330796 | LOC_Os06g23530.1 | LOC_Os02g52250.1 | 0.73 |
| 4340964 | 4331005 | LOC_Os06g23530.1 | LOC_Os02g55260.1 | 0.506 |
| 4340964 | 4333814 | LOC_Os06g23530.1 | LOC_Os03g49210.1 | 0.413 |
| 4340964 | 4336833 | LOC_Os06g23530.1 | LOC_Os04g50660.1 | 0.565 |
| 4340964 | 4342673 | LOC_Os06g23530.1 | LOC_Os07g10350.1 | 0.636 |
| 4340964 | 4345065 | LOC_Os06g23530.1 | LOC_Os08g14990.1 | 0.446 |
| 4340964 | 4345708 | LOC_Os06g23530.1 | LOC_Os08g34650.1 | 0.446 |
| 4340964 | 4345910 | LOC_Os06g23530.1 | LOC_Os08g38410.1 | 0.534 |
| 4340964 | 4347095 | LOC_Os06g23530.1 | LOC_Os09g25540.1 | 0.446 |
| 4341066 | 4324511 | LOC_Os06g29080.1 | LOC_Os01g05620.1 | 0.404 |
| 4341066 | 4324660 | LOC_Os06g29080.1 | LOC_Os01g52380.1 | 0.404 |
| 4341066 | 4333771 | LOC_Os06g29080.1 | LOC_Os03g48320.1 | 0.404 |
| 4341066 | 4334935 | LOC_Os06g29080.1 | LOC_Os04g02110.1 | 0.404 |
| 4341066 | 4335058 | LOC_Os06g29080.1 | LOC_Os04g08740.1 | 0.415 |
| 4341066 | 4336120 | LOC_Os06g29080.1 | LOC_Os04g39460.1 | 0.404 |
| 4341066 | 4338096 | LOC_Os06g29080.1 | LOC_Os05g11990.1 | 0.703 |
| 4341066 | 4338531 | LOC_Os06g29080.1 | LOC_Os05g29030.1 | 0.724 |
| 4341066 | 4339763 | LOC_Os06g29080.1 | LOC_Os05g50970.1 | 0.724 |
| 4341066 | 4340110 | LOC_Os06g29080.1 | LOC_Os06g05359.1 | 0.404 |
| 4341066 | 4340753 | LOC_Os06g29080.1 | LOC_Os06g16450.1 | 0.404 |
| 4341066 | 4341646 | LOC_Os06g29080.1 | LOC_Os06g43670.1 | 0.404 |
| 4341066 | 4342017 | LOC_Os06g29080.1 | LOC_Os06g49430.1 | 0.469 |
| 4341066 | 4343409 | LOC_Os06g29080.1 | LOC_Os07g33730.1 | 0.404 |
| 4341066 | 4344698 | LOC_Os06g29080.1 | LOC_Os08g06060.1 | 0.468 |
| 4341066 | 4345202 | LOC_Os06g29080.1 | LOC_Os08g20000.1 | 0.404 |
| 4341066 | 4345297 | LOC_Os06g29080.1 | LOC_Os08g24380.1 | 0.404 |
| 4341066 | 4345628 | LOC_Os06g29080.1 | LOC_Os08g33200.1 | 0.562 |
| 4341066 | 4347005 | LOC_Os06g29080.1 | LOC_Os09g23740.1 | 0.562 |
| 4341066 | 4347787 | LOC_Os06g29080.1 | LOC_Os09g37860.1 | 0.408 |
| 4341066 | 4349742 | LOC_Os06g29080.1 | LOC_Os11g04600.1 | 0.423 |
| 4341066 | 4350109 | LOC_Os06g29080.1 | LOC_Os11g11770.1 | 0.404 |
| 4341066 | 4350130 | LOC_Os06g29080.1 | LOC_Os11g12300.1 | 0.404 |
| 4341066 | 4350814 | LOC_Os06g29080.1 | LOC_Os11g37860.1 | 0.404 |
| 4341066 | 4350954 | LOC_Os06g29080.1 | LOC_Os11g40780.1 | 0.404 |
| 4341066 | 4350996 | LOC_Os06g29080.1 | LOC_Os11g42040.1 | 0.404 |
| 4341066 | 4351038 | LOC_Os06g29080.1 | LOC_Os11g43250.1 | 0.404 |
| 4341066 | 4351041 | LOC_Os06g29080.1 | LOC_Os11g43390.1 | 0.404 |
| 4341066 | 4351457 | LOC_Os06g29080.1 | LOC_Os12g04410.1 | 0.423 |
| 4341066 | 4351749 | LOC_Os06g29080.1 | LOC_Os12g10410.1 | 0.404 |
| 4341066 | 4352130 | LOC_Os06g29080.1 | LOC_Os12g25170.1 | 0.404 |
| 4341066 | 4352270 | LOC_Os06g29080.1 | LOC_Os12g31620.1 | 0.404 |
| 4341066 | IRL7 | LOC_Os06g29080.1 | LOC_Os03g11360.2 | 0.404 |
| 4341066 | LOC_Os02g49270.1 | LOC_Os06g29080.1 | LOC_Os02g49270.1 | 0.408 |
| 4341119 | 4342017 | LOC_Os06g30710.1 | LOC_Os06g49430.1 | 0.587 |
| 4341119 | 4344698 | LOC_Os06g30710.1 | LOC_Os08g06060.1 | 0.587 |
| 4341219 | 4326546 | LOC_Os06g34690.1 | LOC_Os01g53920.1 | 0.641 |
| 4341219 | 4329448 | LOC_Os06g34690.1 | LOC_Os02g30620.1 | 0.575 |
| 4341219 | 4330971 | LOC_Os06g34690.1 | LOC_Os02g54820.1 | 0.423 |
| 4341219 | 4332080 | LOC_Os06g34690.1 | LOC_Os03g11910.1 | 0.843 |
| 4341219 | 4334290 | LOC_Os06g34690.1 | LOC_Os03g56460.1 | 0.427 |
| 4341219 | 4338096 | LOC_Os06g34690.1 | LOC_Os05g11990.1 | 0.661 |
| 4341219 | 4341853 | LOC_Os06g34690.1 | LOC_Os06g46600.1 | 0.843 |
| 4341219 | 4342673 | LOC_Os06g34690.1 | LOC_Os07g10350.1 | 0.492 |
| 4341219 | 4343196 | LOC_Os06g34690.1 | LOC_Os07g28800.1 | 0.575 |
| 4341219 | 4344935 | LOC_Os06g34690.1 | LOC_Os08g10608.1 | 0.51 |
| 4341219 | 4345065 | LOC_Os06g34690.1 | LOC_Os08g14990.1 | 0.641 |
| 4341219 | 4345708 | LOC_Os06g34690.1 | LOC_Os08g34650.1 | 0.641 |
| 4341219 | 4347095 | LOC_Os06g34690.1 | LOC_Os09g25540.1 | 0.641 |
| 4341219 | 4347311 | LOC_Os06g34690.1 | LOC_Os09g29070.1 | 0.427 |
| 4341219 | 4347787 | LOC_Os06g34690.1 | LOC_Os09g37860.1 | 0.468 |
| 4341219 | LOC_Os02g49270.1 | LOC_Os06g34690.1 | LOC_Os02g49270.1 | 0.635 |
| 4341252 | 4334290 | LOC_Os06g35540.1 | LOC_Os03g56460.1 | 0.566 |
| 4341252 | 4335673 | LOC_Os06g35540.1 | LOC_Os04g32020.1 | 0.924 |
| 4341252 | 4346699 | LOC_Os06g35540.1 | LOC_Os09g14670.1 | 0.441 |
| 4341252 | 4347172 | LOC_Os06g35540.1 | LOC_Os09g26880.1 | 0.438 |
| 4341252 | 4347311 | LOC_Os06g35540.1 | LOC_Os09g29070.1 | 0.566 |
| 4341252 | 4348561 | LOC_Os06g35540.1 | LOC_Os10g26010.1 | 0.428 |
| 4341252 | 4351017 | LOC_Os06g35540.1 | LOC_Os11g42510.1 | 0.689 |
| 4341252 | 4351847 | LOC_Os06g35540.1 | LOC_Os12g13320.1 | 0.928 |
| 4341281 | 4328702 | LOC_Os06g36090.1 | LOC_Os02g11960.1 | 0.643 |
| 4341281 | 4337645 | LOC_Os06g36090.1 | LOC_Os05g02890.1 | 0.638 |
| 4341288 | 4342673 | LOC_Os06g36220.1 | LOC_Os07g10350.1 | 0.425 |
| 4341510 | 4328135 | LOC_Os06g40840.1 | LOC_Os02g03060.1 | 0.717 |
| 4341510 | 4330866 | LOC_Os06g40840.1 | LOC_Os02g53120.1 | 0.687 |
| 4341510 | 4333586 | LOC_Os06g40840.1 | LOC_Os03g44760.1 | 0.776 |
| 4341510 | 4337886 | LOC_Os06g40840.1 | LOC_Os05g06840.1 | 0.585 |
| 4341510 | 4343388 | LOC_Os06g40840.1 | LOC_Os07g33370.1 | 0.732 |
| 4341568 | 4324511 | LOC_Os06g41980.1 | LOC_Os01g05620.1 | 0.404 |
| 4341568 | 4324660 | LOC_Os06g41980.1 | LOC_Os01g52380.1 | 0.404 |
| 4341568 | 4333771 | LOC_Os06g41980.1 | LOC_Os03g48320.1 | 0.404 |
| 4341568 | 4334935 | LOC_Os06g41980.1 | LOC_Os04g02110.1 | 0.404 |
| 4341568 | 4335058 | LOC_Os06g41980.1 | LOC_Os04g08740.1 | 0.415 |
| 4341568 | 4336120 | LOC_Os06g41980.1 | LOC_Os04g39460.1 | 0.404 |
| 4341568 | 4338096 | LOC_Os06g41980.1 | LOC_Os05g11990.1 | 0.703 |
| 4341568 | 4338531 | LOC_Os06g41980.1 | LOC_Os05g29030.1 | 0.703 |
| 4341568 | 4339763 | LOC_Os06g41980.1 | LOC_Os05g50970.1 | 0.703 |
| 4341568 | 4340110 | LOC_Os06g41980.1 | LOC_Os06g05359.1 | 0.404 |
| 4341568 | 4340753 | LOC_Os06g41980.1 | LOC_Os06g16450.1 | 0.404 |
| 4341568 | 4341646 | LOC_Os06g41980.1 | LOC_Os06g43670.1 | 0.404 |
| 4341568 | 4342017 | LOC_Os06g41980.1 | LOC_Os06g49430.1 | 0.469 |
| 4341568 | 4343409 | LOC_Os06g41980.1 | LOC_Os07g33730.1 | 0.404 |
| 4341568 | 4344698 | LOC_Os06g41980.1 | LOC_Os08g06060.1 | 0.492 |
| 4341568 | 4345202 | LOC_Os06g41980.1 | LOC_Os08g20000.1 | 0.404 |
| 4341568 | 4345297 | LOC_Os06g41980.1 | LOC_Os08g24380.1 | 0.404 |
| 4341568 | 4345628 | LOC_Os06g41980.1 | LOC_Os08g33200.1 | 0.562 |
| 4341568 | 4347005 | LOC_Os06g41980.1 | LOC_Os09g23740.1 | 0.562 |
| 4341568 | 4347787 | LOC_Os06g41980.1 | LOC_Os09g37860.1 | 0.408 |
| 4341568 | 4350109 | LOC_Os06g41980.1 | LOC_Os11g11770.1 | 0.404 |
| 4341568 | 4350130 | LOC_Os06g41980.1 | LOC_Os11g12300.1 | 0.404 |
| 4341568 | 4350814 | LOC_Os06g41980.1 | LOC_Os11g37860.1 | 0.404 |
| 4341568 | 4350954 | LOC_Os06g41980.1 | LOC_Os11g40780.1 | 0.404 |
| 4341568 | 4350996 | LOC_Os06g41980.1 | LOC_Os11g42040.1 | 0.404 |
| 4341568 | 4351038 | LOC_Os06g41980.1 | LOC_Os11g43250.1 | 0.404 |
| 4341568 | 4351041 | LOC_Os06g41980.1 | LOC_Os11g43390.1 | 0.404 |
| 4341568 | 4351749 | LOC_Os06g41980.1 | LOC_Os12g10410.1 | 0.404 |
| 4341568 | 4352130 | LOC_Os06g41980.1 | LOC_Os12g25170.1 | 0.404 |
| 4341568 | 4352270 | LOC_Os06g41980.1 | LOC_Os12g31620.1 | 0.404 |
| 4341568 | IRL7 | LOC_Os06g41980.1 | LOC_Os03g11360.2 | 0.404 |
| 4341568 | LOC_Os02g49270.1 | LOC_Os06g41980.1 | LOC_Os02g49270.1 | 0.408 |
| 4341646 | 4326546 | LOC_Os06g43670.1 | LOC_Os01g53920.1 | 0.404 |
| 4341646 | 4327667 | LOC_Os06g43670.1 | LOC_Os01g40590.1 | 0.404 |
| 4341646 | 4328725 | LOC_Os06g43670.1 | LOC_Os02g12440.1 | 0.404 |
| 4341646 | 4330701 | LOC_Os06g43670.1 | LOC_Os02g50970.1 | 0.404 |
| 4341646 | 4331134 | LOC_Os06g43670.1 | LOC_Os02g57080.1 | 0.404 |
| 4341646 | 4333060 | LOC_Os06g43670.1 | LOC_Os03g28300.1 | 0.404 |
| 4341646 | 4334921 | LOC_Os06g43670.1 | LOC_Os04g01874.1 | 0.404 |
| 4341646 | 4335824 | LOC_Os06g43670.1 | LOC_Os04g34250.1 | 0.404 |
| 4341646 | 4337251 | LOC_Os06g43670.1 | LOC_Os04g56090.1 | 0.404 |
| 4341646 | 4338080 | LOC_Os06g43670.1 | LOC_Os05g11750.1 | 0.404 |
| 4341646 | 4338096 | LOC_Os06g43670.1 | LOC_Os05g11990.1 | 0.628 |
| 4341646 | 4339142 | LOC_Os06g43670.1 | LOC_Os05g40770.1 | 0.404 |
| 4341646 | 4341066 | LOC_Os06g43670.1 | LOC_Os06g29080.1 | 0.404 |
| 4341646 | 4341568 | LOC_Os06g43670.1 | LOC_Os06g41980.1 | 0.404 |
| 4341646 | 4342396 | LOC_Os06g43670.1 | LOC_Os07g05370.1 | 0.404 |
| 4341646 | 4342410 | LOC_Os06g43670.1 | LOC_Os07g05620.1 | 0.404 |
| 4341646 | 4343699 | LOC_Os06g43670.1 | LOC_Os07g38810.1 | 0.404 |
| 4341646 | 4343832 | LOC_Os06g43670.1 | LOC_Os07g41140.1 | 0.404 |
| 4341646 | 4345628 | LOC_Os06g43670.1 | LOC_Os08g33200.1 | 0.806 |
| 4341646 | 4345708 | LOC_Os06g43670.1 | LOC_Os08g34650.1 | 0.404 |
| 4341646 | 4347005 | LOC_Os06g43670.1 | LOC_Os09g23740.1 | 0.806 |
| 4341646 | 4352691 | LOC_Os06g43670.1 | LOC_Os12g40419.1 | 0.404 |
| 4341646 | 4352904 | LOC_Os06g43670.1 | LOC_Os12g44090.1 | 0.404 |
| 4341655 | 4324495 | LOC_Os06g43790.1 | LOC_Os01g05900.1 | 0.683 |
| 4341655 | 4324500 | LOC_Os06g43790.1 | LOC_Os01g05630.1 | 0.683 |
| 4341655 | 4324980 | LOC_Os06g43790.1 | LOC_Os01g67970.1 | 0.414 |
| 4341655 | 4327384 | LOC_Os06g43790.1 | LOC_Os01g62230.1 | 0.683 |
| 4341655 | 4339099 | LOC_Os06g43790.1 | LOC_Os05g39850.1 | 0.443 |
| 4341655 | H2B.9 | LOC_Os06g43790.1 | LOC_Os05g49860.1 | 0.683 |
| 4341683 | 4324495 | LOC_Os06g44060.1 | LOC_Os01g05900.1 | 0.769 |
| 4341683 | 4327790 | LOC_Os06g44060.1 | LOC_Os01g13570.1 | 0.654 |
| 4341683 | 4339523 | LOC_Os06g44060.1 | LOC_Os05g47660.1 | 0.403 |
| 4341853 | 4324082 | LOC_Os06g46600.1 | LOC_Os01g06320.1 | 0.572 |
| 4341853 | 4324584 | LOC_Os06g46600.1 | LOC_Os01g11920.1 | 0.656 |
| 4341853 | 4326546 | LOC_Os06g46600.1 | LOC_Os01g53920.1 | 0.776 |
| 4341853 | 4329448 | LOC_Os06g46600.1 | LOC_Os02g30620.1 | 0.879 |
| 4341853 | 4330971 | LOC_Os06g46600.1 | LOC_Os02g54820.1 | 0.544 |
| 4341853 | 4331168 | LOC_Os06g46600.1 | LOC_Os02g57470.1 | 0.404 |
| 4341853 | 4332080 | LOC_Os06g46600.1 | LOC_Os03g11910.1 | 0.653 |
| 4341853 | 4338096 | LOC_Os06g46600.1 | LOC_Os05g11990.1 | 0.928 |
| 4341853 | 4341219 | LOC_Os06g46600.1 | LOC_Os06g34690.1 | 0.843 |
| 4341853 | 4342673 | LOC_Os06g46600.1 | LOC_Os07g10350.1 | 0.444 |
| 4341853 | 4343196 | LOC_Os06g46600.1 | LOC_Os07g28800.1 | 0.906 |
| 4341853 | 4345065 | LOC_Os06g46600.1 | LOC_Os08g14990.1 | 0.776 |
| 4341853 | 4345628 | LOC_Os06g46600.1 | LOC_Os08g33200.1 | 0.88 |
| 4341853 | 4345708 | LOC_Os06g46600.1 | LOC_Os08g34650.1 | 0.776 |
| 4341853 | 4347005 | LOC_Os06g46600.1 | LOC_Os09g23740.1 | 0.88 |
| 4341853 | 4347095 | LOC_Os06g46600.1 | LOC_Os09g25540.1 | 0.776 |
| 4341853 | 4348731 | LOC_Os06g46600.1 | LOC_Os10g30580.1 | 0.456 |
| 4341853 | 4349490 | LOC_Os06g46600.1 | LOC_Os10g42510.1 | 0.487 |
| 4341947 | 4338096 | LOC_Os06g48240.1 | LOC_Os05g11990.1 | 0.401 |
| 4341947 | 4342049 | LOC_Os06g48240.1 | LOC_Os06g49870.1 | 0.403 |
| 4341965 | 4325022 | LOC_Os06g48720.1 | LOC_Os01g67770.1 | 0.61 |
| 4341965 | 4326027 | LOC_Os06g48720.1 | LOC_Os01g25820.1 | 0.461 |
| 4341965 | 4326546 | LOC_Os06g48720.1 | LOC_Os01g53920.1 | 0.551 |
| 4341965 | 4337257 | LOC_Os06g48720.1 | LOC_Os04g56160.1 | 0.745 |
| 4341965 | 4338096 | LOC_Os06g48720.1 | LOC_Os05g11990.1 | 0.483 |
| 4341965 | 4345065 | LOC_Os06g48720.1 | LOC_Os08g14990.1 | 0.551 |
| 4341965 | 4345708 | LOC_Os06g48720.1 | LOC_Os08g34650.1 | 0.551 |
| 4341965 | 4347095 | LOC_Os06g48720.1 | LOC_Os09g25540.1 | 0.551 |
| 4341965 | 4351005 | LOC_Os06g48720.1 | LOC_Os11g42200.1 | 0.612 |
| 4342017 | 4324584 | LOC_Os06g49430.1 | LOC_Os01g11920.1 | 0.516 |
| 4342017 | 4324980 | LOC_Os06g49430.1 | LOC_Os01g67970.1 | 0.718 |
| 4342017 | 4326027 | LOC_Os06g49430.1 | LOC_Os01g25820.1 | 0.431 |
| 4342017 | 4326270 | LOC_Os06g49430.1 | LOC_Os01g16414.1 | 0.519 |
| 4342017 | 4326546 | LOC_Os06g49430.1 | LOC_Os01g53920.1 | 0.765 |
| 4342017 | 4327667 | LOC_Os06g49430.1 | LOC_Os01g40590.1 | 0.468 |
| 4342017 | 4328135 | LOC_Os06g49430.1 | LOC_Os02g03060.1 | 0.594 |
| 4342017 | 4328300 | LOC_Os06g49430.1 | LOC_Os02g05510.1 | 0.414 |
| 4342017 | 4328725 | LOC_Os06g49430.1 | LOC_Os02g12440.1 | 0.469 |
| 4342017 | 4329677 | LOC_Os06g49430.1 | LOC_Os02g35180.1 | 0.414 |
| 4342017 | 4330701 | LOC_Os06g49430.1 | LOC_Os02g50970.1 | 0.538 |
| 4342017 | 4331134 | LOC_Os06g49430.1 | LOC_Os02g57080.1 | 0.468 |
| 4342017 | 4333060 | LOC_Os06g49430.1 | LOC_Os03g28300.1 | 0.516 |
| 4342017 | 4333882 | LOC_Os06g49430.1 | LOC_Os03g50310.1 | 0.414 |
| 4342017 | 4334214 | LOC_Os06g49430.1 | LOC_Os03g55560.1 | 0.714 |
| 4342017 | 4334921 | LOC_Os06g49430.1 | LOC_Os04g01874.1 | 0.492 |
| 4342017 | 4335058 | LOC_Os06g49430.1 | LOC_Os04g08740.1 | 0.48 |
| 4342017 | 4335824 | LOC_Os06g49430.1 | LOC_Os04g34250.1 | 0.469 |
| 4342017 | 4337251 | LOC_Os06g49430.1 | LOC_Os04g56090.1 | 0.469 |
| 4342017 | 4337257 | LOC_Os06g49430.1 | LOC_Os04g56160.1 | 0.412 |
| 4342017 | 4337619 | LOC_Os06g49430.1 | LOC_Os05g02500.1 | 0.873 |
| 4342017 | 4338080 | LOC_Os06g49430.1 | LOC_Os05g11750.1 | 0.468 |
| 4342017 | 4338096 | LOC_Os06g49430.1 | LOC_Os05g11990.1 | 0.439 |
| 4342017 | 4338531 | LOC_Os06g49430.1 | LOC_Os05g29030.1 | 0.771 |
| 4342017 | 4339142 | LOC_Os06g49430.1 | LOC_Os05g40770.1 | 0.469 |
| 4342017 | 4339763 | LOC_Os06g49430.1 | LOC_Os05g50970.1 | 0.771 |
| 4342017 | 4340847 | LOC_Os06g49430.1 | LOC_Os06g20340.1 | 0.873 |
| 4342017 | 4341066 | LOC_Os06g49430.1 | LOC_Os06g29080.1 | 0.469 |
| 4342017 | 4341119 | LOC_Os06g49430.1 | LOC_Os06g30710.1 | 0.587 |
| 4342017 | 4341568 | LOC_Os06g49430.1 | LOC_Os06g41980.1 | 0.469 |
| 4342017 | 4342396 | LOC_Os06g49430.1 | LOC_Os07g05370.1 | 0.469 |
| 4342017 | 4342410 | LOC_Os06g49430.1 | LOC_Os07g05620.1 | 0.517 |
| 4342017 | 4343699 | LOC_Os06g49430.1 | LOC_Os07g38810.1 | 0.469 |
| 4342017 | 4343794 | LOC_Os06g49430.1 | LOC_Os07g40550.1 | 0.45 |
| 4342017 | 4343832 | LOC_Os06g49430.1 | LOC_Os07g41140.1 | 0.468 |
| 4342017 | 4345065 | LOC_Os06g49430.1 | LOC_Os08g14990.1 | 0.764 |
| 4342017 | 4345628 | LOC_Os06g49430.1 | LOC_Os08g33200.1 | 0.519 |
| 4342017 | 4345708 | LOC_Os06g49430.1 | LOC_Os08g34650.1 | 0.762 |
| 4342017 | 4346276 | LOC_Os06g49430.1 | LOC_Os08g44050.1 | 0.693 |
| 4342017 | 4347005 | LOC_Os06g49430.1 | LOC_Os09g23740.1 | 0.519 |
| 4342017 | 4347095 | LOC_Os06g49430.1 | LOC_Os09g25540.1 | 0.764 |
| 4342017 | 4349742 | LOC_Os06g49430.1 | LOC_Os11g04600.1 | 0.476 |
| 4342017 | 4351431 | LOC_Os06g49430.1 | LOC_Os12g03990.1 | 0.873 |
| 4342017 | 4351457 | LOC_Os06g49430.1 | LOC_Os12g04410.1 | 0.476 |
| 4342017 | 4352691 | LOC_Os06g49430.1 | LOC_Os12g40419.1 | 0.469 |
| 4342017 | 4352904 | LOC_Os06g49430.1 | LOC_Os12g44090.1 | 0.468 |
| 4342017 | IRL7 | LOC_Os06g49430.1 | LOC_Os03g11360.2 | 0.523 |
| 4342017 | LOC_Os03g64080.1 | LOC_Os06g49430.1 | LOC_Os03g64080.1 | 0.656 |
| 4342049 | 4338096 | LOC_Os06g49870.1 | LOC_Os05g11990.1 | 0.478 |
| 4342049 | 4341947 | LOC_Os06g49870.1 | LOC_Os06g48240.1 | 0.403 |
| 4342396 | 4324511 | LOC_Os07g05370.1 | LOC_Os01g05620.1 | 0.404 |
| 4342396 | 4324660 | LOC_Os07g05370.1 | LOC_Os01g52380.1 | 0.404 |
| 4342396 | 4333771 | LOC_Os07g05370.1 | LOC_Os03g48320.1 | 0.404 |
| 4342396 | 4334935 | LOC_Os07g05370.1 | LOC_Os04g02110.1 | 0.404 |
| 4342396 | 4335058 | LOC_Os07g05370.1 | LOC_Os04g08740.1 | 0.415 |
| 4342396 | 4336120 | LOC_Os07g05370.1 | LOC_Os04g39460.1 | 0.404 |
| 4342396 | 4338096 | LOC_Os07g05370.1 | LOC_Os05g11990.1 | 0.703 |
| 4342396 | 4338531 | LOC_Os07g05370.1 | LOC_Os05g29030.1 | 0.724 |
| 4342396 | 4339763 | LOC_Os07g05370.1 | LOC_Os05g50970.1 | 0.724 |
| 4342396 | 4340110 | LOC_Os07g05370.1 | LOC_Os06g05359.1 | 0.404 |
| 4342396 | 4340753 | LOC_Os07g05370.1 | LOC_Os06g16450.1 | 0.404 |
| 4342396 | 4341646 | LOC_Os07g05370.1 | LOC_Os06g43670.1 | 0.404 |
| 4342396 | 4342017 | LOC_Os07g05370.1 | LOC_Os06g49430.1 | 0.469 |
| 4342396 | 4343409 | LOC_Os07g05370.1 | LOC_Os07g33730.1 | 0.404 |
| 4342396 | 4344698 | LOC_Os07g05370.1 | LOC_Os08g06060.1 | 0.492 |
| 4342396 | 4345202 | LOC_Os07g05370.1 | LOC_Os08g20000.1 | 0.404 |
| 4342396 | 4345297 | LOC_Os07g05370.1 | LOC_Os08g24380.1 | 0.404 |
| 4342396 | 4345628 | LOC_Os07g05370.1 | LOC_Os08g33200.1 | 0.562 |
| 4342396 | 4347005 | LOC_Os07g05370.1 | LOC_Os09g23740.1 | 0.562 |
| 4342396 | 4347787 | LOC_Os07g05370.1 | LOC_Os09g37860.1 | 0.408 |
| 4342396 | 4349742 | LOC_Os07g05370.1 | LOC_Os11g04600.1 | 0.423 |
| 4342396 | 4350109 | LOC_Os07g05370.1 | LOC_Os11g11770.1 | 0.404 |
| 4342396 | 4350130 | LOC_Os07g05370.1 | LOC_Os11g12300.1 | 0.404 |
| 4342396 | 4350814 | LOC_Os07g05370.1 | LOC_Os11g37860.1 | 0.404 |
| 4342396 | 4350954 | LOC_Os07g05370.1 | LOC_Os11g40780.1 | 0.404 |
| 4342396 | 4350996 | LOC_Os07g05370.1 | LOC_Os11g42040.1 | 0.404 |
| 4342396 | 4351038 | LOC_Os07g05370.1 | LOC_Os11g43250.1 | 0.404 |
| 4342396 | 4351041 | LOC_Os07g05370.1 | LOC_Os11g43390.1 | 0.404 |
| 4342396 | 4351457 | LOC_Os07g05370.1 | LOC_Os12g04410.1 | 0.423 |
| 4342396 | 4351749 | LOC_Os07g05370.1 | LOC_Os12g10410.1 | 0.404 |
| 4342396 | 4352130 | LOC_Os07g05370.1 | LOC_Os12g25170.1 | 0.404 |
| 4342396 | 4352270 | LOC_Os07g05370.1 | LOC_Os12g31620.1 | 0.404 |
| 4342396 | IRL7 | LOC_Os07g05370.1 | LOC_Os03g11360.2 | 0.404 |
| 4342396 | LOC_Os02g49270.1 | LOC_Os07g05370.1 | LOC_Os02g49270.1 | 0.408 |
| 4342410 | 4324364 | LOC_Os07g05620.1 | LOC_Os01g69030.2 | 0.421 |
| 4342410 | 4324511 | LOC_Os07g05620.1 | LOC_Os01g05620.1 | 0.404 |
| 4342410 | 4324584 | LOC_Os07g05620.1 | LOC_Os01g11920.1 | 0.403 |
| 4342410 | 4324660 | LOC_Os07g05620.1 | LOC_Os01g52380.1 | 0.404 |
| 4342410 | 4324980 | LOC_Os07g05620.1 | LOC_Os01g67970.1 | 0.41 |
| 4342410 | 4328135 | LOC_Os07g05620.1 | LOC_Os02g03060.1 | 0.442 |
| 4342410 | 4333771 | LOC_Os07g05620.1 | LOC_Os03g48320.1 | 0.404 |
| 4342410 | 4334214 | LOC_Os07g05620.1 | LOC_Os03g55560.1 | 0.45 |
| 4342410 | 4334935 | LOC_Os07g05620.1 | LOC_Os04g02110.1 | 0.404 |
| 4342410 | 4335058 | LOC_Os07g05620.1 | LOC_Os04g08740.1 | 0.415 |
| 4342410 | 4336120 | LOC_Os07g05620.1 | LOC_Os04g39460.1 | 0.404 |
| 4342410 | 4338096 | LOC_Os07g05620.1 | LOC_Os05g11990.1 | 0.708 |
| 4342410 | 4338531 | LOC_Os07g05620.1 | LOC_Os05g29030.1 | 0.738 |
| 4342410 | 4339763 | LOC_Os07g05620.1 | LOC_Os05g50970.1 | 0.738 |
| 4342410 | 4340110 | LOC_Os07g05620.1 | LOC_Os06g05359.1 | 0.404 |
| 4342410 | 4340753 | LOC_Os07g05620.1 | LOC_Os06g16450.1 | 0.404 |
| 4342410 | 4341646 | LOC_Os07g05620.1 | LOC_Os06g43670.1 | 0.404 |
| 4342410 | 4342017 | LOC_Os07g05620.1 | LOC_Os06g49430.1 | 0.517 |
| 4342410 | 4342694 | LOC_Os07g05620.1 | LOC_Os07g10630.1 | 0.404 |
| 4342410 | 4343409 | LOC_Os07g05620.1 | LOC_Os07g33730.1 | 0.404 |
| 4342410 | 4344698 | LOC_Os07g05620.1 | LOC_Os08g06060.1 | 0.576 |
| 4342410 | 4345065 | LOC_Os07g05620.1 | LOC_Os08g14990.1 | 0.404 |
| 4342410 | 4345202 | LOC_Os07g05620.1 | LOC_Os08g20000.1 | 0.404 |
| 4342410 | 4345297 | LOC_Os07g05620.1 | LOC_Os08g24380.1 | 0.404 |
| 4342410 | 4345628 | LOC_Os07g05620.1 | LOC_Os08g33200.1 | 0.578 |
| 4342410 | 4346276 | LOC_Os07g05620.1 | LOC_Os08g44050.1 | 0.41 |
| 4342410 | 4347005 | LOC_Os07g05620.1 | LOC_Os09g23740.1 | 0.578 |
| 4342410 | 4347787 | LOC_Os07g05620.1 | LOC_Os09g37860.1 | 0.408 |
| 4342410 | 4349742 | LOC_Os07g05620.1 | LOC_Os11g04600.1 | 0.421 |
| 4342410 | 4350109 | LOC_Os07g05620.1 | LOC_Os11g11770.1 | 0.404 |
| 4342410 | 4350130 | LOC_Os07g05620.1 | LOC_Os11g12300.1 | 0.404 |
| 4342410 | 4350814 | LOC_Os07g05620.1 | LOC_Os11g37860.1 | 0.404 |
| 4342410 | 4350954 | LOC_Os07g05620.1 | LOC_Os11g40780.1 | 0.404 |
| 4342410 | 4350996 | LOC_Os07g05620.1 | LOC_Os11g42040.1 | 0.404 |
| 4342410 | 4351038 | LOC_Os07g05620.1 | LOC_Os11g43250.1 | 0.404 |
| 4342410 | 4351041 | LOC_Os07g05620.1 | LOC_Os11g43390.1 | 0.404 |
| 4342410 | 4351457 | LOC_Os07g05620.1 | LOC_Os12g04410.1 | 0.421 |
| 4342410 | 4351749 | LOC_Os07g05620.1 | LOC_Os12g10410.1 | 0.404 |
| 4342410 | 4352130 | LOC_Os07g05620.1 | LOC_Os12g25170.1 | 0.404 |
| 4342410 | 4352270 | LOC_Os07g05620.1 | LOC_Os12g31620.1 | 0.404 |
| 4342410 | IRL7 | LOC_Os07g05620.1 | LOC_Os03g11360.2 | 0.404 |
| 4342410 | LOC_Os02g49270.1 | LOC_Os07g05620.1 | LOC_Os02g49270.1 | 0.408 |
| 4342472 | 4324481 | LOC_Os07g06980.1 | LOC_Os03g17100.1 | 0.657 |
| 4342472 | 4324495 | LOC_Os07g06980.1 | LOC_Os01g05900.1 | 0.774 |
| 4342472 | 4324500 | LOC_Os07g06980.1 | LOC_Os01g05630.1 | 0.774 |
| 4342472 | 4324584 | LOC_Os07g06980.1 | LOC_Os01g11920.1 | 0.475 |
| 4342472 | 4324980 | LOC_Os07g06980.1 | LOC_Os01g67970.1 | 0.81 |
| 4342472 | 4326270 | LOC_Os07g06980.1 | LOC_Os01g16414.1 | 0.433 |
| 4342472 | 4327384 | LOC_Os07g06980.1 | LOC_Os01g62230.1 | 0.774 |
| 4342472 | 4330796 | LOC_Os07g06980.1 | LOC_Os02g52250.1 | 0.604 |
| 4342472 | 4331253 | LOC_Os07g06980.1 | LOC_Os02g58490.1 | 0.449 |
| 4342472 | 4333814 | LOC_Os07g06980.1 | LOC_Os03g49210.1 | 0.628 |
| 4342472 | 4338043 | LOC_Os07g06980.1 | LOC_Os05g10770.1 | 0.464 |
| 4342472 | 4338096 | LOC_Os07g06980.1 | LOC_Os05g11990.1 | 0.476 |
| 4342472 | 4343299 | LOC_Os07g06980.1 | LOC_Os07g31450.1 | 0.554 |
| 4342472 | 4344819 | LOC_Os07g06980.1 | LOC_Os08g08210.1 | 0.729 |
| 4342472 | 4346276 | LOC_Os07g06980.1 | LOC_Os08g44050.1 | 0.496 |
| 4342472 | H2B.9 | LOC_Os07g06980.1 | LOC_Os05g49860.1 | 0.774 |
| 4342544 | 4326378 | LOC_Os07g08050.1 | LOC_Os01g40980.1 | 0.722 |
| 4342645 | 4324709 | LOC_Os07g09690.1 | LOC_Os01g56570.1 | 0.439 |
| 4342645 | 4332380 | LOC_Os07g09690.1 | LOC_Os03g16290.1 | 0.543 |
| 4342645 | STLP1 | LOC_Os07g09690.1 | LOC_Os01g63970.1 | 0.434 |
| 4342673 | 4331005 | LOC_Os07g10350.1 | LOC_Os02g55260.1 | 0.769 |
| 4342673 | 4331991 | LOC_Os07g10350.1 | LOC_Os03g10780.1 | 0.439 |
| 4342673 | 4332080 | LOC_Os07g10350.1 | LOC_Os03g11910.1 | 0.437 |
| 4342673 | 4332567 | LOC_Os07g10350.1 | LOC_Os03g18840.1 | 0.983 |
| 4342673 | 4333574 | LOC_Os07g10350.1 | LOC_Os03g44530.1 | 0.719 |
| 4342673 | 4333814 | LOC_Os07g10350.1 | LOC_Os03g49210.1 | 0.978 |
| 4342673 | 4336833 | LOC_Os07g10350.1 | LOC_Os04g50660.1 | 0.996 |
| 4342673 | 4338096 | LOC_Os07g10350.1 | LOC_Os05g11990.1 | 0.457 |
| 4342673 | 4340964 | LOC_Os07g10350.1 | LOC_Os06g23530.1 | 0.636 |
| 4342673 | 4341219 | LOC_Os07g10350.1 | LOC_Os06g34690.1 | 0.492 |
| 4342673 | 4341288 | LOC_Os07g10350.1 | LOC_Os06g36220.1 | 0.425 |
| 4342673 | 4341853 | LOC_Os07g10350.1 | LOC_Os06g46600.1 | 0.444 |
| 4342673 | 4342770 | LOC_Os07g10350.1 | LOC_Os07g12640.1 | 0.424 |
| 4342673 | 4343339 | LOC_Os07g10350.1 | LOC_Os07g32430.1 | 0.617 |
| 4342673 | 4344612 | LOC_Os07g10350.1 | LOC_Os08g04180.1 | 0.404 |
| 4342673 | 4344935 | LOC_Os07g10350.1 | LOC_Os08g10608.1 | 0.807 |
| 4342673 | 4345304 | LOC_Os07g10350.1 | LOC_Os08g24760.1 | 0.617 |
| 4342673 | 4345910 | LOC_Os07g10350.1 | LOC_Os08g38410.1 | 0.408 |
| 4342673 | 4347787 | LOC_Os07g10350.1 | LOC_Os09g37860.1 | 0.997 |
| 4342673 | 4350837 | LOC_Os07g10350.1 | LOC_Os11g38170.1 | 0.619 |
| 4342673 | LOC_Os02g49270.1 | LOC_Os07g10350.1 | LOC_Os02g49270.1 | 0.988 |
| 4342694 | 4338096 | LOC_Os07g10630.1 | LOC_Os05g11990.1 | 0.628 |
| 4342694 | 4342410 | LOC_Os07g10630.1 | LOC_Os07g05620.1 | 0.404 |
| 4342694 | 4345628 | LOC_Os07g10630.1 | LOC_Os08g33200.1 | 0.803 |
| 4342694 | 4347005 | LOC_Os07g10630.1 | LOC_Os09g23740.1 | 0.803 |
| 4342770 | 4330971 | LOC_Os07g12640.1 | LOC_Os02g54820.1 | 0.478 |
| 4342770 | 4334290 | LOC_Os07g12640.1 | LOC_Os03g56460.1 | 0.497 |
| 4342770 | 4334412 | LOC_Os07g12640.1 | LOC_Os03g58100.1 | 0.431 |
| 4342770 | 4339523 | LOC_Os07g12640.1 | LOC_Os05g47660.1 | 0.401 |
| 4342770 | 4342673 | LOC_Os07g12640.1 | LOC_Os07g10350.1 | 0.424 |
| 4342770 | 4347311 | LOC_Os07g12640.1 | LOC_Os09g29070.1 | 0.497 |
| 4342995 | 4347823 | LOC_Os07g22580.1 | LOC_Os09g38450.1 | 0.67 |
| 4343183 | 4329422 | LOC_Os07g28480.1 | LOC_Os02g29960.1 | 0.412 |
| 4343183 | 4340915 | LOC_Os07g28480.1 | LOC_Os06g22340.1 | 0.412 |
| 4343183 | 4351008 | LOC_Os07g28480.1 | LOC_Os11g42350.1 | 0.908 |
| 4343196 | 4324082 | LOC_Os07g28800.1 | LOC_Os01g06320.1 | 0.476 |
| 4343196 | 4324584 | LOC_Os07g28800.1 | LOC_Os01g11920.1 | 0.669 |
| 4343196 | 4331168 | LOC_Os07g28800.1 | LOC_Os02g57470.1 | 0.513 |
| 4343196 | 4332080 | LOC_Os07g28800.1 | LOC_Os03g11910.1 | 0.91 |
| 4343196 | 4338096 | LOC_Os07g28800.1 | LOC_Os05g11990.1 | 0.972 |
| 4343196 | 4341219 | LOC_Os07g28800.1 | LOC_Os06g34690.1 | 0.575 |
| 4343196 | 4341853 | LOC_Os07g28800.1 | LOC_Os06g46600.1 | 0.906 |
| 4343196 | 4345628 | LOC_Os07g28800.1 | LOC_Os08g33200.1 | 0.636 |
| 4343196 | 4347005 | LOC_Os07g28800.1 | LOC_Os09g23740.1 | 0.636 |
| 4343243 | 4343388 | LOC_Os07g30110.1 | LOC_Os07g33370.1 | 0.441 |
| 4343276 | 4330902 | LOC_Os07g30980.1 | LOC_Os02g53680.1 | 0.473 |
| 4343276 | 4331991 | LOC_Os07g30980.1 | LOC_Os03g10780.1 | 0.691 |
| 4343276 | 4343299 | LOC_Os07g30980.1 | LOC_Os07g31450.1 | 0.459 |
| 4343276 | 4344306 | LOC_Os07g30980.1 | LOC_Os07g48360.1 | 0.944 |
| 4343276 | 4348805 | LOC_Os07g30980.1 | LOC_Os10g31970.1 | 0.459 |
| 4343299 | 4324495 | LOC_Os07g31450.1 | LOC_Os01g05900.1 | 0.463 |
| 4343299 | 4324500 | LOC_Os07g31450.1 | LOC_Os01g05630.1 | 0.463 |
| 4343299 | 4324980 | LOC_Os07g31450.1 | LOC_Os01g67970.1 | 0.44 |
| 4343299 | 4326270 | LOC_Os07g31450.1 | LOC_Os01g16414.1 | 0.435 |
| 4343299 | 4326546 | LOC_Os07g31450.1 | LOC_Os01g53920.1 | 0.762 |
| 4343299 | 4327384 | LOC_Os07g31450.1 | LOC_Os01g62230.1 | 0.463 |
| 4343299 | 4335089 | LOC_Os07g31450.1 | LOC_Os04g09860.1 | 0.444 |
| 4343299 | 4337317 | LOC_Os07g31450.1 | LOC_Os04g56980.1 | 0.531 |
| 4343299 | 4337645 | LOC_Os07g31450.1 | LOC_Os05g02890.1 | 0.466 |
| 4343299 | 4342472 | LOC_Os07g31450.1 | LOC_Os07g06980.1 | 0.554 |
| 4343299 | 4343276 | LOC_Os07g31450.1 | LOC_Os07g30980.1 | 0.459 |
| 4343299 | 4344306 | LOC_Os07g31450.1 | LOC_Os07g48360.1 | 0.587 |
| 4343299 | 4344819 | LOC_Os07g31450.1 | LOC_Os08g08210.1 | 0.539 |
| 4343299 | 4345065 | LOC_Os07g31450.1 | LOC_Os08g14990.1 | 0.762 |
| 4343299 | 4345708 | LOC_Os07g31450.1 | LOC_Os08g34650.1 | 0.762 |
| 4343299 | 4347095 | LOC_Os07g31450.1 | LOC_Os09g25540.1 | 0.762 |
| 4343299 | H2B.9 | LOC_Os07g31450.1 | LOC_Os05g49860.1 | 0.463 |
| 4343339 | 4326546 | LOC_Os07g32430.1 | LOC_Os01g53920.1 | 0.446 |
| 4343339 | 4330796 | LOC_Os07g32430.1 | LOC_Os02g52250.1 | 0.97 |
| 4343339 | 4333814 | LOC_Os07g32430.1 | LOC_Os03g49210.1 | 0.451 |
| 4343339 | 4336833 | LOC_Os07g32430.1 | LOC_Os04g50660.1 | 0.562 |
| 4343339 | 4342673 | LOC_Os07g32430.1 | LOC_Os07g10350.1 | 0.617 |
| 4343339 | 4345065 | LOC_Os07g32430.1 | LOC_Os08g14990.1 | 0.446 |
| 4343339 | 4345502 | LOC_Os07g32430.1 | LOC_Os08g30820.1 | 0.9 |
| 4343339 | 4345708 | LOC_Os07g32430.1 | LOC_Os08g34650.1 | 0.446 |
| 4343339 | 4347095 | LOC_Os07g32430.1 | LOC_Os09g25540.1 | 0.446 |
| 4343352 | 4329422 | LOC_Os07g32620.1 | LOC_Os02g29960.1 | 0.436 |
| 4343352 | 4336977 | LOC_Os07g32620.1 | LOC_Os04g52370.1 | 0.426 |
| 4343352 | 4339523 | LOC_Os07g32620.1 | LOC_Os05g47660.1 | 0.411 |
| 4343352 | 4340915 | LOC_Os07g32620.1 | LOC_Os06g22340.1 | 0.436 |
| 4343352 | 4343395 | LOC_Os07g32620.1 | LOC_Os07g33480.1 | 0.436 |
| 4343352 | 4347809 | LOC_Os07g32620.1 | LOC_Os09g38239.1 | 0.408 |
| 4343352 | 4348853 | LOC_Os07g32620.1 | LOC_Os10g32980.1 | 0.445 |
| 4343352 | BGLU2 | LOC_Os07g32620.1 | LOC_Os01g59819.1 | 0.485 |
| 4343388 | 4330866 | LOC_Os07g33370.1 | LOC_Os02g53120.1 | 0.444 |
| 4343388 | 4332190 | LOC_Os07g33370.1 | LOC_Os03g13460.1 | 0.732 |
| 4343388 | 4337533 | LOC_Os07g33370.1 | LOC_Os05g01230.1 | 0.487 |
| 4343388 | 4337886 | LOC_Os07g33370.1 | LOC_Os05g06840.1 | 0.413 |
| 4343388 | 4339099 | LOC_Os07g33370.1 | LOC_Os05g39850.1 | 0.525 |
| 4343388 | 4340849 | LOC_Os07g33370.1 | LOC_Os06g20370.1 | 0.754 |
| 4343388 | 4341510 | LOC_Os07g33370.1 | LOC_Os06g40840.1 | 0.732 |
| 4343388 | 4343243 | LOC_Os07g33370.1 | LOC_Os07g30110.1 | 0.441 |
| 4343388 | 4347221 | LOC_Os07g33370.1 | LOC_Os09g27700.1 | 0.732 |
| 4343388 | 4347823 | LOC_Os07g33370.1 | LOC_Os09g38450.1 | 0.589 |
| 4343395 | 4326546 | LOC_Os07g33480.1 | LOC_Os01g53920.1 | 0.495 |
| 4343395 | 4329422 | LOC_Os07g33480.1 | LOC_Os02g29960.1 | 0.494 |
| 4343395 | 4340373 | LOC_Os07g33480.1 | LOC_Os06g09240.1 | 0.436 |
| 4343395 | 4340915 | LOC_Os07g33480.1 | LOC_Os06g22340.1 | 0.51 |
| 4343395 | 4343352 | LOC_Os07g33480.1 | LOC_Os07g32620.1 | 0.436 |
| 4343395 | 4345065 | LOC_Os07g33480.1 | LOC_Os08g14990.1 | 0.495 |
| 4343395 | 4345708 | LOC_Os07g33480.1 | LOC_Os08g34650.1 | 0.495 |
| 4343395 | 4347095 | LOC_Os07g33480.1 | LOC_Os09g25540.1 | 0.495 |
| 4343395 | 4347172 | LOC_Os07g33480.1 | LOC_Os09g26880.1 | 0.456 |
| 4343395 | 4347809 | LOC_Os07g33480.1 | LOC_Os09g38239.1 | 0.402 |
| 4343395 | YUCCA1 | LOC_Os07g33480.1 | LOC_Os01g45760.1 | 0.536 |
| 4343409 | 4326546 | LOC_Os07g33730.1 | LOC_Os01g53920.1 | 0.404 |
| 4343409 | 4327667 | LOC_Os07g33730.1 | LOC_Os01g40590.1 | 0.404 |
| 4343409 | 4328725 | LOC_Os07g33730.1 | LOC_Os02g12440.1 | 0.404 |
| 4343409 | 4330701 | LOC_Os07g33730.1 | LOC_Os02g50970.1 | 0.404 |
| 4343409 | 4331134 | LOC_Os07g33730.1 | LOC_Os02g57080.1 | 0.404 |
| 4343409 | 4333060 | LOC_Os07g33730.1 | LOC_Os03g28300.1 | 0.404 |
| 4343409 | 4334921 | LOC_Os07g33730.1 | LOC_Os04g01874.1 | 0.404 |
| 4343409 | 4335824 | LOC_Os07g33730.1 | LOC_Os04g34250.1 | 0.404 |
| 4343409 | 4337251 | LOC_Os07g33730.1 | LOC_Os04g56090.1 | 0.404 |
| 4343409 | 4338080 | LOC_Os07g33730.1 | LOC_Os05g11750.1 | 0.404 |
| 4343409 | 4338096 | LOC_Os07g33730.1 | LOC_Os05g11990.1 | 0.628 |
| 4343409 | 4339142 | LOC_Os07g33730.1 | LOC_Os05g40770.1 | 0.404 |
| 4343409 | 4341066 | LOC_Os07g33730.1 | LOC_Os06g29080.1 | 0.404 |
| 4343409 | 4341568 | LOC_Os07g33730.1 | LOC_Os06g41980.1 | 0.404 |
| 4343409 | 4342396 | LOC_Os07g33730.1 | LOC_Os07g05370.1 | 0.404 |
| 4343409 | 4342410 | LOC_Os07g33730.1 | LOC_Os07g05620.1 | 0.404 |
| 4343409 | 4343699 | LOC_Os07g33730.1 | LOC_Os07g38810.1 | 0.404 |
| 4343409 | 4343832 | LOC_Os07g33730.1 | LOC_Os07g41140.1 | 0.404 |
| 4343409 | 4345065 | LOC_Os07g33730.1 | LOC_Os08g14990.1 | 0.404 |
| 4343409 | 4345628 | LOC_Os07g33730.1 | LOC_Os08g33200.1 | 0.806 |
| 4343409 | 4345708 | LOC_Os07g33730.1 | LOC_Os08g34650.1 | 0.404 |
| 4343409 | 4347005 | LOC_Os07g33730.1 | LOC_Os09g23740.1 | 0.806 |
| 4343409 | 4347095 | LOC_Os07g33730.1 | LOC_Os09g25540.1 | 0.404 |
| 4343409 | 4352691 | LOC_Os07g33730.1 | LOC_Os12g40419.1 | 0.404 |
| 4343409 | 4352904 | LOC_Os07g33730.1 | LOC_Os12g44090.1 | 0.404 |
| 4343665 | 4347935 | LOC_Os07g38360.2 | LOC_Os10g01044.1 | 0.493 |
| 4343699 | 4324511 | LOC_Os07g38810.1 | LOC_Os01g05620.1 | 0.404 |
| 4343699 | 4324660 | LOC_Os07g38810.1 | LOC_Os01g52380.1 | 0.404 |
| 4343699 | 4333771 | LOC_Os07g38810.1 | LOC_Os03g48320.1 | 0.404 |
| 4343699 | 4334935 | LOC_Os07g38810.1 | LOC_Os04g02110.1 | 0.404 |
| 4343699 | 4335058 | LOC_Os07g38810.1 | LOC_Os04g08740.1 | 0.415 |
| 4343699 | 4336120 | LOC_Os07g38810.1 | LOC_Os04g39460.1 | 0.404 |
| 4343699 | 4338096 | LOC_Os07g38810.1 | LOC_Os05g11990.1 | 0.703 |
| 4343699 | 4338531 | LOC_Os07g38810.1 | LOC_Os05g29030.1 | 0.703 |
| 4343699 | 4339763 | LOC_Os07g38810.1 | LOC_Os05g50970.1 | 0.703 |
| 4343699 | 4340110 | LOC_Os07g38810.1 | LOC_Os06g05359.1 | 0.404 |
| 4343699 | 4340753 | LOC_Os07g38810.1 | LOC_Os06g16450.1 | 0.404 |
| 4343699 | 4341646 | LOC_Os07g38810.1 | LOC_Os06g43670.1 | 0.404 |
| 4343699 | 4342017 | LOC_Os07g38810.1 | LOC_Os06g49430.1 | 0.469 |
| 4343699 | 4343409 | LOC_Os07g38810.1 | LOC_Os07g33730.1 | 0.404 |
| 4343699 | 4344698 | LOC_Os07g38810.1 | LOC_Os08g06060.1 | 0.468 |
| 4343699 | 4345202 | LOC_Os07g38810.1 | LOC_Os08g20000.1 | 0.485 |
| 4343699 | 4345297 | LOC_Os07g38810.1 | LOC_Os08g24380.1 | 0.404 |
| 4343699 | 4345628 | LOC_Os07g38810.1 | LOC_Os08g33200.1 | 0.562 |
| 4343699 | 4347005 | LOC_Os07g38810.1 | LOC_Os09g23740.1 | 0.562 |
| 4343699 | 4347787 | LOC_Os07g38810.1 | LOC_Os09g37860.1 | 0.408 |
| 4343699 | 4350109 | LOC_Os07g38810.1 | LOC_Os11g11770.1 | 0.404 |
| 4343699 | 4350130 | LOC_Os07g38810.1 | LOC_Os11g12300.1 | 0.404 |
| 4343699 | 4350814 | LOC_Os07g38810.1 | LOC_Os11g37860.1 | 0.404 |
| 4343699 | 4350954 | LOC_Os07g38810.1 | LOC_Os11g40780.1 | 0.404 |
| 4343699 | 4350996 | LOC_Os07g38810.1 | LOC_Os11g42040.1 | 0.404 |
| 4343699 | 4351038 | LOC_Os07g38810.1 | LOC_Os11g43250.1 | 0.404 |
| 4343699 | 4351041 | LOC_Os07g38810.1 | LOC_Os11g43390.1 | 0.404 |
| 4343699 | 4351749 | LOC_Os07g38810.1 | LOC_Os12g10410.1 | 0.404 |
| 4343699 | 4352130 | LOC_Os07g38810.1 | LOC_Os12g25170.1 | 0.44 |
| 4343699 | 4352270 | LOC_Os07g38810.1 | LOC_Os12g31620.1 | 0.404 |
| 4343699 | IRL7 | LOC_Os07g38810.1 | LOC_Os03g11360.2 | 0.404 |
| 4343699 | LOC_Os02g49270.1 | LOC_Os07g38810.1 | LOC_Os02g49270.1 | 0.408 |
| 4343794 | 4342017 | LOC_Os07g40550.1 | LOC_Os06g49430.1 | 0.45 |
| 4343794 | 4344698 | LOC_Os07g40550.1 | LOC_Os08g06060.1 | 0.44 |
| 4343794 | 4344819 | LOC_Os07g40550.1 | LOC_Os08g08210.1 | 0.549 |
| 4343794 | 4350352 | LOC_Os07g40550.1 | LOC_Os11g20384.1 | 0.438 |
| 4343832 | 4324511 | LOC_Os07g41140.1 | LOC_Os01g05620.1 | 0.404 |
| 4343832 | 4324660 | LOC_Os07g41140.1 | LOC_Os01g52380.1 | 0.404 |
| 4343832 | 4333771 | LOC_Os07g41140.1 | LOC_Os03g48320.1 | 0.404 |
| 4343832 | 4334935 | LOC_Os07g41140.1 | LOC_Os04g02110.1 | 0.404 |
| 4343832 | 4335058 | LOC_Os07g41140.1 | LOC_Os04g08740.1 | 0.415 |
| 4343832 | 4336120 | LOC_Os07g41140.1 | LOC_Os04g39460.1 | 0.404 |
| 4343832 | 4338096 | LOC_Os07g41140.1 | LOC_Os05g11990.1 | 0.703 |
| 4343832 | 4338531 | LOC_Os07g41140.1 | LOC_Os05g29030.1 | 0.703 |
| 4343832 | 4339763 | LOC_Os07g41140.1 | LOC_Os05g50970.1 | 0.703 |
| 4343832 | 4340110 | LOC_Os07g41140.1 | LOC_Os06g05359.1 | 0.404 |
| 4343832 | 4340753 | LOC_Os07g41140.1 | LOC_Os06g16450.1 | 0.404 |
| 4343832 | 4341646 | LOC_Os07g41140.1 | LOC_Os06g43670.1 | 0.404 |
| 4343832 | 4342017 | LOC_Os07g41140.1 | LOC_Os06g49430.1 | 0.468 |
| 4343832 | 4343409 | LOC_Os07g41140.1 | LOC_Os07g33730.1 | 0.404 |
| 4343832 | 4344698 | LOC_Os07g41140.1 | LOC_Os08g06060.1 | 0.468 |
| 4343832 | 4345202 | LOC_Os07g41140.1 | LOC_Os08g20000.1 | 0.404 |
| 4343832 | 4345297 | LOC_Os07g41140.1 | LOC_Os08g24380.1 | 0.404 |
| 4343832 | 4345628 | LOC_Os07g41140.1 | LOC_Os08g33200.1 | 0.562 |
| 4343832 | 4347005 | LOC_Os07g41140.1 | LOC_Os09g23740.1 | 0.562 |
| 4343832 | 4347787 | LOC_Os07g41140.1 | LOC_Os09g37860.1 | 0.408 |
| 4343832 | 4350109 | LOC_Os07g41140.1 | LOC_Os11g11770.1 | 0.404 |
| 4343832 | 4350130 | LOC_Os07g41140.1 | LOC_Os11g12300.1 | 0.404 |
| 4343832 | 4350814 | LOC_Os07g41140.1 | LOC_Os11g37860.1 | 0.404 |
| 4343832 | 4350954 | LOC_Os07g41140.1 | LOC_Os11g40780.1 | 0.404 |
| 4343832 | 4350996 | LOC_Os07g41140.1 | LOC_Os11g42040.1 | 0.404 |
| 4343832 | 4351038 | LOC_Os07g41140.1 | LOC_Os11g43250.1 | 0.404 |
| 4343832 | 4351041 | LOC_Os07g41140.1 | LOC_Os11g43390.1 | 0.404 |
| 4343832 | 4351749 | LOC_Os07g41140.1 | LOC_Os12g10410.1 | 0.404 |
| 4343832 | 4352130 | LOC_Os07g41140.1 | LOC_Os12g25170.1 | 0.404 |
| 4343832 | 4352270 | LOC_Os07g41140.1 | LOC_Os12g31620.1 | 0.404 |
| 4343832 | LOC_Os02g49270.1 | LOC_Os07g41140.1 | LOC_Os02g49270.1 | 0.408 |
| 4344172 | 4327967 | LOC_Os07g46560.1 | LOC_Os01g58780.1 | 0.902 |
| 4344172 | 4327983 | LOC_Os07g46560.1 | LOC_Os02g01170.1 | 0.9 |
| 4344172 | 4337586 | LOC_Os07g46560.1 | LOC_Os05g01940.1 | 0.902 |
| 4344267 | 4327790 | LOC_Os07g47820.1 | LOC_Os01g13570.1 | 0.42 |
| 4344267 | 4332628 | LOC_Os07g47820.1 | LOC_Os03g19680.1 | 0.471 |
| 4344267 | 4347172 | LOC_Os07g47820.1 | LOC_Os09g26880.1 | 0.929 |
| 4344268 | 4329416 | LOC_Os07g47830.1 | LOC_Os02g29530.1 | 0.922 |
| 4344268 | 4338309 | LOC_Os07g47830.1 | LOC_Os05g22800.1 | 0.824 |
| 4344306 | 4326151 | LOC_Os07g48360.1 | LOC_Os01g01689.1 | 0.663 |
| 4344306 | 4326378 | LOC_Os07g48360.1 | LOC_Os01g40980.1 | 0.476 |
| 4344306 | 4330902 | LOC_Os07g48360.1 | LOC_Os02g53680.1 | 0.866 |
| 4344306 | 4331991 | LOC_Os07g48360.1 | LOC_Os03g10780.1 | 0.792 |
| 4344306 | 4339099 | LOC_Os07g48360.1 | LOC_Os05g39850.1 | 0.472 |
| 4344306 | 4343276 | LOC_Os07g48360.1 | LOC_Os07g30980.1 | 0.944 |
| 4344306 | 4343299 | LOC_Os07g48360.1 | LOC_Os07g31450.1 | 0.587 |
| 4344306 | 4348805 | LOC_Os07g48360.1 | LOC_Os10g31970.1 | 0.582 |
| 4344329 | 4337178 | LOC_Os07g48610.1 | LOC_Os04g55060.1 | 0.438 |
| 4344329 | 4347677 | LOC_Os07g48610.1 | LOC_Os09g36240.1 | 0.581 |
| 4344410 | 4337360 | LOC_Os08g01054.1 | LOC_Os04g57560.1 | 0.436 |
| 4344584 | 4324364 | LOC_Os08g03570.1 | LOC_Os01g69030.2 | 0.573 |
| 4344584 | 4334290 | LOC_Os08g03570.1 | LOC_Os03g56460.1 | 0.46 |
| 4344584 | 4336977 | LOC_Os08g03570.1 | LOC_Os04g52370.1 | 0.484 |
| 4344584 | 4338096 | LOC_Os08g03570.1 | LOC_Os05g11990.1 | 0.432 |
| 4344584 | 4347311 | LOC_Os08g03570.1 | LOC_Os09g29070.1 | 0.46 |
| 4344584 | 4348853 | LOC_Os08g03570.1 | LOC_Os10g32980.1 | 0.485 |
| 4344612 | 4338096 | LOC_Os08g04180.1 | LOC_Os05g11990.1 | 0.532 |
| 4344612 | 4342673 | LOC_Os08g04180.1 | LOC_Os07g10350.1 | 0.404 |
| 4344612 | 4348561 | LOC_Os08g04180.1 | LOC_Os10g26010.1 | 0.453 |
| 4344698 | 4324584 | LOC_Os08g06060.1 | LOC_Os01g11920.1 | 0.516 |
| 4344698 | 4324980 | LOC_Os08g06060.1 | LOC_Os01g67970.1 | 0.718 |
| 4344698 | 4326027 | LOC_Os08g06060.1 | LOC_Os01g25820.1 | 0.431 |
| 4344698 | 4326270 | LOC_Os08g06060.1 | LOC_Os01g16414.1 | 0.519 |
| 4344698 | 4326546 | LOC_Os08g06060.1 | LOC_Os01g53920.1 | 0.846 |
| 4344698 | 4327667 | LOC_Os08g06060.1 | LOC_Os01g40590.1 | 0.468 |
| 4344698 | 4328135 | LOC_Os08g06060.1 | LOC_Os02g03060.1 | 0.619 |
| 4344698 | 4328300 | LOC_Os08g06060.1 | LOC_Os02g05510.1 | 0.414 |
| 4344698 | 4328725 | LOC_Os08g06060.1 | LOC_Os02g12440.1 | 0.492 |
| 4344698 | 4329677 | LOC_Os08g06060.1 | LOC_Os02g35180.1 | 0.414 |
| 4344698 | 4330701 | LOC_Os08g06060.1 | LOC_Os02g50970.1 | 0.514 |
| 4344698 | 4331134 | LOC_Os08g06060.1 | LOC_Os02g57080.1 | 0.468 |
| 4344698 | 4333060 | LOC_Os08g06060.1 | LOC_Os03g28300.1 | 0.514 |
| 4344698 | 4333882 | LOC_Os08g06060.1 | LOC_Os03g50310.1 | 0.414 |
| 4344698 | 4334214 | LOC_Os08g06060.1 | LOC_Os03g55560.1 | 0.687 |
| 4344698 | 4334921 | LOC_Os08g06060.1 | LOC_Os04g01874.1 | 0.492 |
| 4344698 | 4335058 | LOC_Os08g06060.1 | LOC_Os04g08740.1 | 0.48 |
| 4344698 | 4335824 | LOC_Os08g06060.1 | LOC_Os04g34250.1 | 0.468 |
| 4344698 | 4337251 | LOC_Os08g06060.1 | LOC_Os04g56090.1 | 0.492 |
| 4344698 | 4337619 | LOC_Os08g06060.1 | LOC_Os05g02500.1 | 0.873 |
| 4344698 | 4338080 | LOC_Os08g06060.1 | LOC_Os05g11750.1 | 0.492 |
| 4344698 | 4338096 | LOC_Os08g06060.1 | LOC_Os05g11990.1 | 0.439 |
| 4344698 | 4338531 | LOC_Os08g06060.1 | LOC_Os05g29030.1 | 0.771 |
| 4344698 | 4339142 | LOC_Os08g06060.1 | LOC_Os05g40770.1 | 0.468 |
| 4344698 | 4339763 | LOC_Os08g06060.1 | LOC_Os05g50970.1 | 0.771 |
| 4344698 | 4340847 | LOC_Os08g06060.1 | LOC_Os06g20340.1 | 0.873 |
| 4344698 | 4341066 | LOC_Os08g06060.1 | LOC_Os06g29080.1 | 0.468 |
| 4344698 | 4341119 | LOC_Os08g06060.1 | LOC_Os06g30710.1 | 0.587 |
| 4344698 | 4341568 | LOC_Os08g06060.1 | LOC_Os06g41980.1 | 0.492 |
| 4344698 | 4342396 | LOC_Os08g06060.1 | LOC_Os07g05370.1 | 0.492 |
| 4344698 | 4342410 | LOC_Os08g06060.1 | LOC_Os07g05620.1 | 0.576 |
| 4344698 | 4343699 | LOC_Os08g06060.1 | LOC_Os07g38810.1 | 0.468 |
| 4344698 | 4343794 | LOC_Os08g06060.1 | LOC_Os07g40550.1 | 0.44 |
| 4344698 | 4343832 | LOC_Os08g06060.1 | LOC_Os07g41140.1 | 0.468 |
| 4344698 | 4345065 | LOC_Os08g06060.1 | LOC_Os08g14990.1 | 0.846 |
| 4344698 | 4345628 | LOC_Os08g06060.1 | LOC_Os08g33200.1 | 0.519 |
| 4344698 | 4345708 | LOC_Os08g06060.1 | LOC_Os08g34650.1 | 0.762 |
| 4344698 | 4346276 | LOC_Os08g06060.1 | LOC_Os08g44050.1 | 0.693 |
| 4344698 | 4347005 | LOC_Os08g06060.1 | LOC_Os09g23740.1 | 0.519 |
| 4344698 | 4347095 | LOC_Os08g06060.1 | LOC_Os09g25540.1 | 0.846 |
| 4344698 | 4349742 | LOC_Os08g06060.1 | LOC_Os11g04600.1 | 0.476 |
| 4344698 | 4351431 | LOC_Os08g06060.1 | LOC_Os12g03990.1 | 0.873 |
| 4344698 | 4351457 | LOC_Os08g06060.1 | LOC_Os12g04410.1 | 0.476 |
| 4344698 | 4352691 | LOC_Os08g06060.1 | LOC_Os12g40419.1 | 0.492 |
| 4344698 | 4352904 | LOC_Os08g06060.1 | LOC_Os12g44090.1 | 0.468 |
| 4344698 | IRL7 | LOC_Os08g06060.1 | LOC_Os03g11360.2 | 0.523 |
| 4344698 | LOC_Os03g64080.1 | LOC_Os08g06060.1 | LOC_Os03g64080.1 | 0.656 |
| 4344819 | 4324481 | LOC_Os08g08210.1 | LOC_Os03g17100.1 | 0.483 |
| 4344819 | 4324495 | LOC_Os08g08210.1 | LOC_Os01g05900.1 | 0.492 |
| 4344819 | 4324500 | LOC_Os08g08210.1 | LOC_Os01g05630.1 | 0.492 |
| 4344819 | 4324980 | LOC_Os08g08210.1 | LOC_Os01g67970.1 | 0.815 |
| 4344819 | 4326151 | LOC_Os08g08210.1 | LOC_Os01g01689.1 | 0.584 |
| 4344819 | 4327384 | LOC_Os08g08210.1 | LOC_Os01g62230.1 | 0.492 |
| 4344819 | 4337360 | LOC_Os08g08210.1 | LOC_Os04g57560.1 | 0.433 |
| 4344819 | 4338043 | LOC_Os08g08210.1 | LOC_Os05g10770.1 | 0.678 |
| 4344819 | 4338096 | LOC_Os08g08210.1 | LOC_Os05g11990.1 | 0.569 |
| 4344819 | 4342472 | LOC_Os08g08210.1 | LOC_Os07g06980.1 | 0.729 |
| 4344819 | 4343299 | LOC_Os08g08210.1 | LOC_Os07g31450.1 | 0.539 |
| 4344819 | 4343794 | LOC_Os08g08210.1 | LOC_Os07g40550.1 | 0.549 |
| 4344819 | 4349776 | LOC_Os08g08210.1 | LOC_Os11g05130.1 | 0.497 |
| 4344819 | 4350837 | LOC_Os08g08210.1 | LOC_Os11g38170.1 | 0.615 |
| 4344819 | H2B.9 | LOC_Os08g08210.1 | LOC_Os05g49860.1 | 0.492 |
| 4344935 | 4332080 | LOC_Os08g10608.1 | LOC_Os03g11910.1 | 0.406 |
| 4344935 | 4333079 | LOC_Os08g10608.1 | LOC_Os03g29260.1 | 0.763 |
| 4344935 | 4341219 | LOC_Os08g10608.1 | LOC_Os06g34690.1 | 0.51 |
| 4344935 | 4342673 | LOC_Os08g10608.1 | LOC_Os07g10350.1 | 0.807 |
| 4344935 | 4347787 | LOC_Os08g10608.1 | LOC_Os09g37860.1 | 0.554 |
| 4344935 | 4350745 | LOC_Os08g10608.1 | LOC_Os11g36050.1 | 0.485 |
| 4345065 | 4324364 | LOC_Os08g14990.1 | LOC_Os01g69030.2 | 0.634 |
| 4345065 | 4324511 | LOC_Os08g14990.1 | LOC_Os01g05620.1 | 0.404 |
| 4345065 | 4324584 | LOC_Os08g14990.1 | LOC_Os01g11920.1 | 0.691 |
| 4345065 | 4324660 | LOC_Os08g14990.1 | LOC_Os01g52380.1 | 0.404 |
| 4345065 | 4324980 | LOC_Os08g14990.1 | LOC_Os01g67970.1 | 0.703 |
| 4345065 | 4325022 | LOC_Os08g14990.1 | LOC_Os01g67770.1 | 0.463 |
| 4345065 | 4326270 | LOC_Os08g14990.1 | LOC_Os01g16414.1 | 0.606 |
| 4345065 | 4326546 | LOC_Os08g14990.1 | LOC_Os01g53920.1 | 0.956 |
| 4345065 | 4327983 | LOC_Os08g14990.1 | LOC_Os02g01170.1 | 0.655 |
| 4345065 | 4328135 | LOC_Os08g14990.1 | LOC_Os02g03060.1 | 0.675 |
| 4345065 | 4328633 | LOC_Os08g14990.1 | LOC_Os02g10520.1 | 0.605 |
| 4345065 | 4329422 | LOC_Os08g14990.1 | LOC_Os02g29960.1 | 0.495 |
| 4345065 | 4330003 | LOC_Os08g14990.1 | LOC_Os02g40664.1 | 0.421 |
| 4345065 | 4331144 | LOC_Os08g14990.1 | LOC_Os02g57190.1 | 0.457 |
| 4345065 | 4331168 | LOC_Os08g14990.1 | LOC_Os02g57470.1 | 0.691 |
| 4345065 | 4332080 | LOC_Os08g14990.1 | LOC_Os03g11910.1 | 0.776 |
| 4345065 | 4333771 | LOC_Os08g14990.1 | LOC_Os03g48320.1 | 0.404 |
| 4345065 | 4334214 | LOC_Os08g14990.1 | LOC_Os03g55560.1 | 0.927 |
| 4345065 | 4334935 | LOC_Os08g14990.1 | LOC_Os04g02110.1 | 0.404 |
| 4345065 | 4335058 | LOC_Os08g14990.1 | LOC_Os04g08740.1 | 0.659 |
| 4345065 | 4335089 | LOC_Os08g14990.1 | LOC_Os04g09860.1 | 0.606 |
| 4345065 | 4336120 | LOC_Os08g14990.1 | LOC_Os04g39460.1 | 0.404 |
| 4345065 | 4337257 | LOC_Os08g14990.1 | LOC_Os04g56160.1 | 0.588 |
| 4345065 | 4337619 | LOC_Os08g14990.1 | LOC_Os05g02500.1 | 0.817 |
| 4345065 | 4337658 | LOC_Os08g14990.1 | LOC_Os05g03100.1 | 0.655 |
| 4345065 | 4337686 | LOC_Os08g14990.1 | LOC_Os05g03610.1 | 0.726 |
| 4345065 | 4338096 | LOC_Os08g14990.1 | LOC_Os05g11990.1 | 0.968 |
| 4345065 | 4338481 | LOC_Os08g14990.1 | LOC_Os05g27880.1 | 0.655 |
| 4345065 | 4338531 | LOC_Os08g14990.1 | LOC_Os05g29030.1 | 0.963 |
| 4345065 | 4339763 | LOC_Os08g14990.1 | LOC_Os05g50970.1 | 0.963 |
| 4345065 | 4340110 | LOC_Os08g14990.1 | LOC_Os06g05359.1 | 0.404 |
| 4345065 | 4340753 | LOC_Os08g14990.1 | LOC_Os06g16450.1 | 0.404 |
| 4345065 | 4340847 | LOC_Os08g14990.1 | LOC_Os06g20340.1 | 0.817 |
| 4345065 | 4340915 | LOC_Os08g14990.1 | LOC_Os06g22340.1 | 0.495 |
| 4345065 | 4340964 | LOC_Os08g14990.1 | LOC_Os06g23530.1 | 0.446 |
| 4345065 | 4341219 | LOC_Os08g14990.1 | LOC_Os06g34690.1 | 0.641 |
| 4345065 | 4341853 | LOC_Os08g14990.1 | LOC_Os06g46600.1 | 0.776 |
| 4345065 | 4341965 | LOC_Os08g14990.1 | LOC_Os06g48720.1 | 0.551 |
| 4345065 | 4342017 | LOC_Os08g14990.1 | LOC_Os06g49430.1 | 0.764 |
| 4345065 | 4342410 | LOC_Os08g14990.1 | LOC_Os07g05620.1 | 0.404 |
| 4345065 | 4343299 | LOC_Os08g14990.1 | LOC_Os07g31450.1 | 0.762 |
| 4345065 | 4343339 | LOC_Os08g14990.1 | LOC_Os07g32430.1 | 0.446 |
| 4345065 | 4343395 | LOC_Os08g14990.1 | LOC_Os07g33480.1 | 0.495 |
| 4345065 | 4343409 | LOC_Os08g14990.1 | LOC_Os07g33730.1 | 0.404 |
| 4345065 | 4344698 | LOC_Os08g14990.1 | LOC_Os08g06060.1 | 0.846 |
| 4345065 | 4345202 | LOC_Os08g14990.1 | LOC_Os08g20000.1 | 0.404 |
| 4345065 | 4345297 | LOC_Os08g14990.1 | LOC_Os08g24380.1 | 0.404 |
| 4345065 | 4345304 | LOC_Os08g14990.1 | LOC_Os08g24760.1 | 0.446 |
| 4345065 | 4345628 | LOC_Os08g14990.1 | LOC_Os08g33200.1 | 0.985 |
| 4345065 | 4345708 | LOC_Os08g14990.1 | LOC_Os08g34650.1 | 0.952 |
| 4345065 | 4345910 | LOC_Os08g14990.1 | LOC_Os08g38410.1 | 0.628 |
| 4345065 | 4346276 | LOC_Os08g14990.1 | LOC_Os08g44050.1 | 0.703 |
| 4345065 | 4346315 | LOC_Os08g14990.1 | LOC_Os08g44510.1 | 0.709 |
| 4345065 | 4347005 | LOC_Os08g14990.1 | LOC_Os09g23740.1 | 0.985 |
| 4345065 | 4347095 | LOC_Os08g14990.1 | LOC_Os09g25540.1 | 0.951 |
| 4345065 | 4347787 | LOC_Os08g14990.1 | LOC_Os09g37860.1 | 0.537 |
| 4345065 | 4348731 | LOC_Os08g14990.1 | LOC_Os10g30580.1 | 0.584 |
| 4345065 | 4348805 | LOC_Os08g14990.1 | LOC_Os10g31970.1 | 0.762 |
| 4345065 | 4348853 | LOC_Os08g14990.1 | LOC_Os10g32980.1 | 0.434 |
| 4345065 | 4349742 | LOC_Os08g14990.1 | LOC_Os11g04600.1 | 0.837 |
| 4345065 | 4350109 | LOC_Os08g14990.1 | LOC_Os11g11770.1 | 0.404 |
| 4345065 | 4350130 | LOC_Os08g14990.1 | LOC_Os11g12300.1 | 0.404 |
| 4345065 | 4350472 | LOC_Os08g14990.1 | LOC_Os11g27264.1 | 0.614 |
| 4345065 | 4350473 | LOC_Os08g14990.1 | LOC_Os11g27329.1 | 0.614 |
| 4345065 | 4350814 | LOC_Os08g14990.1 | LOC_Os11g37860.1 | 0.404 |
| 4345065 | 4350954 | LOC_Os08g14990.1 | LOC_Os11g40780.1 | 0.404 |
| 4345065 | 4350996 | LOC_Os08g14990.1 | LOC_Os11g42040.1 | 0.404 |
| 4345065 | 4351038 | LOC_Os08g14990.1 | LOC_Os11g43250.1 | 0.404 |
| 4345065 | 4351041 | LOC_Os08g14990.1 | LOC_Os11g43390.1 | 0.404 |
| 4345065 | 4351431 | LOC_Os08g14990.1 | LOC_Os12g03990.1 | 0.817 |
| 4345065 | 4351457 | LOC_Os08g14990.1 | LOC_Os12g04410.1 | 0.837 |
| 4345065 | 4351749 | LOC_Os08g14990.1 | LOC_Os12g10410.1 | 0.404 |
| 4345065 | 4352130 | LOC_Os08g14990.1 | LOC_Os12g25170.1 | 0.404 |
| 4345065 | 4352270 | LOC_Os08g14990.1 | LOC_Os12g31620.1 | 0.404 |
| 4345065 | LOC_Os02g49270.1 | LOC_Os08g14990.1 | LOC_Os02g49270.1 | 0.537 |
| 4345202 | 4326546 | LOC_Os08g20000.1 | LOC_Os01g53920.1 | 0.404 |
| 4345202 | 4327667 | LOC_Os08g20000.1 | LOC_Os01g40590.1 | 0.404 |
| 4345202 | 4328725 | LOC_Os08g20000.1 | LOC_Os02g12440.1 | 0.404 |
| 4345202 | 4330701 | LOC_Os08g20000.1 | LOC_Os02g50970.1 | 0.404 |
| 4345202 | 4331134 | LOC_Os08g20000.1 | LOC_Os02g57080.1 | 0.404 |
| 4345202 | 4333060 | LOC_Os08g20000.1 | LOC_Os03g28300.1 | 0.404 |
| 4345202 | 4334921 | LOC_Os08g20000.1 | LOC_Os04g01874.1 | 0.404 |
| 4345202 | 4335824 | LOC_Os08g20000.1 | LOC_Os04g34250.1 | 0.404 |
| 4345202 | 4337251 | LOC_Os08g20000.1 | LOC_Os04g56090.1 | 0.404 |
| 4345202 | 4338080 | LOC_Os08g20000.1 | LOC_Os05g11750.1 | 0.404 |
| 4345202 | 4338096 | LOC_Os08g20000.1 | LOC_Os05g11990.1 | 0.628 |
| 4345202 | 4339142 | LOC_Os08g20000.1 | LOC_Os05g40770.1 | 0.404 |
| 4345202 | 4341066 | LOC_Os08g20000.1 | LOC_Os06g29080.1 | 0.404 |
| 4345202 | 4341568 | LOC_Os08g20000.1 | LOC_Os06g41980.1 | 0.404 |
| 4345202 | 4342396 | LOC_Os08g20000.1 | LOC_Os07g05370.1 | 0.404 |
| 4345202 | 4342410 | LOC_Os08g20000.1 | LOC_Os07g05620.1 | 0.404 |
| 4345202 | 4343699 | LOC_Os08g20000.1 | LOC_Os07g38810.1 | 0.485 |
| 4345202 | 4343832 | LOC_Os08g20000.1 | LOC_Os07g41140.1 | 0.404 |
| 4345202 | 4345065 | LOC_Os08g20000.1 | LOC_Os08g14990.1 | 0.404 |
| 4345202 | 4345628 | LOC_Os08g20000.1 | LOC_Os08g33200.1 | 0.806 |
| 4345202 | 4345708 | LOC_Os08g20000.1 | LOC_Os08g34650.1 | 0.404 |
| 4345202 | 4347005 | LOC_Os08g20000.1 | LOC_Os09g23740.1 | 0.806 |
| 4345202 | 4347095 | LOC_Os08g20000.1 | LOC_Os09g25540.1 | 0.404 |
| 4345202 | 4352691 | LOC_Os08g20000.1 | LOC_Os12g40419.1 | 0.404 |
| 4345202 | 4352904 | LOC_Os08g20000.1 | LOC_Os12g44090.1 | 0.404 |
| 4345297 | 4326546 | LOC_Os08g24380.1 | LOC_Os01g53920.1 | 0.404 |
| 4345297 | 4327667 | LOC_Os08g24380.1 | LOC_Os01g40590.1 | 0.404 |
| 4345297 | 4328725 | LOC_Os08g24380.1 | LOC_Os02g12440.1 | 0.404 |
| 4345297 | 4330701 | LOC_Os08g24380.1 | LOC_Os02g50970.1 | 0.404 |
| 4345297 | 4331134 | LOC_Os08g24380.1 | LOC_Os02g57080.1 | 0.404 |
| 4345297 | 4333060 | LOC_Os08g24380.1 | LOC_Os03g28300.1 | 0.404 |
| 4345297 | 4334921 | LOC_Os08g24380.1 | LOC_Os04g01874.1 | 0.404 |
| 4345297 | 4335824 | LOC_Os08g24380.1 | LOC_Os04g34250.1 | 0.404 |
| 4345297 | 4337251 | LOC_Os08g24380.1 | LOC_Os04g56090.1 | 0.404 |
| 4345297 | 4338080 | LOC_Os08g24380.1 | LOC_Os05g11750.1 | 0.404 |
| 4345297 | 4338096 | LOC_Os08g24380.1 | LOC_Os05g11990.1 | 0.628 |
| 4345297 | 4339142 | LOC_Os08g24380.1 | LOC_Os05g40770.1 | 0.404 |
| 4345297 | 4341066 | LOC_Os08g24380.1 | LOC_Os06g29080.1 | 0.404 |
| 4345297 | 4341568 | LOC_Os08g24380.1 | LOC_Os06g41980.1 | 0.404 |
| 4345297 | 4342396 | LOC_Os08g24380.1 | LOC_Os07g05370.1 | 0.404 |
| 4345297 | 4342410 | LOC_Os08g24380.1 | LOC_Os07g05620.1 | 0.404 |
| 4345297 | 4343699 | LOC_Os08g24380.1 | LOC_Os07g38810.1 | 0.404 |
| 4345297 | 4343832 | LOC_Os08g24380.1 | LOC_Os07g41140.1 | 0.404 |
| 4345297 | 4345065 | LOC_Os08g24380.1 | LOC_Os08g14990.1 | 0.404 |
| 4345297 | 4345628 | LOC_Os08g24380.1 | LOC_Os08g33200.1 | 0.806 |
| 4345297 | 4345708 | LOC_Os08g24380.1 | LOC_Os08g34650.1 | 0.404 |
| 4345297 | 4347005 | LOC_Os08g24380.1 | LOC_Os09g23740.1 | 0.806 |
| 4345297 | 4347095 | LOC_Os08g24380.1 | LOC_Os09g25540.1 | 0.404 |
| 4345297 | 4352691 | LOC_Os08g24380.1 | LOC_Os12g40419.1 | 0.404 |
| 4345297 | 4352904 | LOC_Os08g24380.1 | LOC_Os12g44090.1 | 0.404 |
| 4345304 | 4326546 | LOC_Os08g24760.1 | LOC_Os01g53920.1 | 0.446 |
| 4345304 | 4330796 | LOC_Os08g24760.1 | LOC_Os02g52250.1 | 0.895 |
| 4345304 | 4333814 | LOC_Os08g24760.1 | LOC_Os03g49210.1 | 0.413 |
| 4345304 | 4336833 | LOC_Os08g24760.1 | LOC_Os04g50660.1 | 0.562 |
| 4345304 | 4342673 | LOC_Os08g24760.1 | LOC_Os07g10350.1 | 0.617 |
| 4345304 | 4345065 | LOC_Os08g24760.1 | LOC_Os08g14990.1 | 0.446 |
| 4345304 | 4345708 | LOC_Os08g24760.1 | LOC_Os08g34650.1 | 0.446 |
| 4345304 | 4345910 | LOC_Os08g24760.1 | LOC_Os08g38410.1 | 0.616 |
| 4345304 | 4347095 | LOC_Os08g24760.1 | LOC_Os09g25540.1 | 0.446 |
| 4345365 | 4324587 | LOC_Os08g27010.1 | LOC_Os01g11946.1 | 0.403 |
| 4345365 | 4326439 | LOC_Os08g27010.1 | LOC_Os01g01302.1 | 0.847 |
| 4345365 | 4329037 | LOC_Os08g27010.1 | LOC_Os02g18450.1 | 0.723 |
| 4345365 | 4334795 | LOC_Os08g27010.1 | LOC_Os03g63360.1 | 0.6 |
| 4345365 | 4346508 | LOC_Os08g27010.1 | LOC_Os09g07570.1 | 0.858 |
| 4345365 | 4350398 | LOC_Os08g27010.1 | LOC_Os11g24450.1 | 0.645 |
| 4345502 | 4328135 | LOC_Os08g30820.1 | LOC_Os02g03060.1 | 0.449 |
| 4345502 | 4330796 | LOC_Os08g30820.1 | LOC_Os02g52250.1 | 0.9 |
| 4345502 | 4343339 | LOC_Os08g30820.1 | LOC_Os07g32430.1 | 0.9 |
| 4345628 | 4324511 | LOC_Os08g33200.1 | LOC_Os01g05620.1 | 0.806 |
| 4345628 | 4324660 | LOC_Os08g33200.1 | LOC_Os01g52380.1 | 0.806 |
| 4345628 | 4326270 | LOC_Os08g33200.1 | LOC_Os01g16414.1 | 0.514 |
| 4345628 | 4326546 | LOC_Os08g33200.1 | LOC_Os01g53920.1 | 0.985 |
| 4345628 | 4327667 | LOC_Os08g33200.1 | LOC_Os01g40590.1 | 0.562 |
| 4345628 | 4328725 | LOC_Os08g33200.1 | LOC_Os02g12440.1 | 0.562 |
| 4345628 | 4329448 | LOC_Os08g33200.1 | LOC_Os02g30620.1 | 0.636 |
| 4345628 | 4330159 | LOC_Os08g33200.1 | LOC_Os02g43350.1 | 0.408 |
| 4345628 | 4330701 | LOC_Os08g33200.1 | LOC_Os02g50970.1 | 0.562 |
| 4345628 | 4330971 | LOC_Os08g33200.1 | LOC_Os02g54820.1 | 0.511 |
| 4345628 | 4331134 | LOC_Os08g33200.1 | LOC_Os02g57080.1 | 0.562 |
| 4345628 | 4331991 | LOC_Os08g33200.1 | LOC_Os03g10780.1 | 0.4 |
| 4345628 | 4332080 | LOC_Os08g33200.1 | LOC_Os03g11910.1 | 0.88 |
| 4345628 | 4333060 | LOC_Os08g33200.1 | LOC_Os03g28300.1 | 0.562 |
| 4345628 | 4333771 | LOC_Os08g33200.1 | LOC_Os03g48320.1 | 0.806 |
| 4345628 | 4334214 | LOC_Os08g33200.1 | LOC_Os03g55560.1 | 0.408 |
| 4345628 | 4334921 | LOC_Os08g33200.1 | LOC_Os04g01874.1 | 0.562 |
| 4345628 | 4334935 | LOC_Os08g33200.1 | LOC_Os04g02110.1 | 0.806 |
| 4345628 | 4335089 | LOC_Os08g33200.1 | LOC_Os04g09860.1 | 0.514 |
| 4345628 | 4335824 | LOC_Os08g33200.1 | LOC_Os04g34250.1 | 0.562 |
| 4345628 | 4336120 | LOC_Os08g33200.1 | LOC_Os04g39460.1 | 0.806 |
| 4345628 | 4337251 | LOC_Os08g33200.1 | LOC_Os04g56090.1 | 0.562 |
| 4345628 | 4337619 | LOC_Os08g33200.1 | LOC_Os05g02500.1 | 0.936 |
| 4345628 | 4337686 | LOC_Os08g33200.1 | LOC_Os05g03610.1 | 0.946 |
| 4345628 | 4338080 | LOC_Os08g33200.1 | LOC_Os05g11750.1 | 0.562 |
| 4345628 | 4338096 | LOC_Os08g33200.1 | LOC_Os05g11990.1 | 0.677 |
| 4345628 | 4339142 | LOC_Os08g33200.1 | LOC_Os05g40770.1 | 0.562 |
| 4345628 | 4340110 | LOC_Os08g33200.1 | LOC_Os06g05359.1 | 0.806 |
| 4345628 | 4340753 | LOC_Os08g33200.1 | LOC_Os06g16450.1 | 0.806 |
| 4345628 | 4340847 | LOC_Os08g33200.1 | LOC_Os06g20340.1 | 0.936 |
| 4345628 | 4341066 | LOC_Os08g33200.1 | LOC_Os06g29080.1 | 0.562 |
| 4345628 | 4341568 | LOC_Os08g33200.1 | LOC_Os06g41980.1 | 0.562 |
| 4345628 | 4341646 | LOC_Os08g33200.1 | LOC_Os06g43670.1 | 0.806 |
| 4345628 | 4341853 | LOC_Os08g33200.1 | LOC_Os06g46600.1 | 0.88 |
| 4345628 | 4342017 | LOC_Os08g33200.1 | LOC_Os06g49430.1 | 0.519 |
| 4345628 | 4342396 | LOC_Os08g33200.1 | LOC_Os07g05370.1 | 0.562 |
| 4345628 | 4342410 | LOC_Os08g33200.1 | LOC_Os07g05620.1 | 0.578 |
| 4345628 | 4342694 | LOC_Os08g33200.1 | LOC_Os07g10630.1 | 0.803 |
| 4345628 | 4343196 | LOC_Os08g33200.1 | LOC_Os07g28800.1 | 0.636 |
| 4345628 | 4343409 | LOC_Os08g33200.1 | LOC_Os07g33730.1 | 0.806 |
| 4345628 | 4343699 | LOC_Os08g33200.1 | LOC_Os07g38810.1 | 0.562 |
| 4345628 | 4343832 | LOC_Os08g33200.1 | LOC_Os07g41140.1 | 0.562 |
| 4345628 | 4344698 | LOC_Os08g33200.1 | LOC_Os08g06060.1 | 0.519 |
| 4345628 | 4345065 | LOC_Os08g33200.1 | LOC_Os08g14990.1 | 0.985 |
| 4345628 | 4345202 | LOC_Os08g33200.1 | LOC_Os08g20000.1 | 0.806 |
| 4345628 | 4345297 | LOC_Os08g33200.1 | LOC_Os08g24380.1 | 0.806 |
| 4345628 | 4345708 | LOC_Os08g33200.1 | LOC_Os08g34650.1 | 0.985 |
| 4345628 | 4347005 | LOC_Os08g33200.1 | LOC_Os09g23740.1 | 0.895 |
| 4345628 | 4347095 | LOC_Os08g33200.1 | LOC_Os09g25540.1 | 0.985 |
| 4345628 | 4347814 | LOC_Os08g33200.1 | LOC_Os09g38330.1 | 0.401 |
| 4345628 | 4349742 | LOC_Os08g33200.1 | LOC_Os11g04600.1 | 0.542 |
| 4345628 | 4350109 | LOC_Os08g33200.1 | LOC_Os11g11770.1 | 0.806 |
| 4345628 | 4350130 | LOC_Os08g33200.1 | LOC_Os11g12300.1 | 0.806 |
| 4345628 | 4350352 | LOC_Os08g33200.1 | LOC_Os11g20384.1 | 0.964 |
| 4345628 | 4350814 | LOC_Os08g33200.1 | LOC_Os11g37860.1 | 0.806 |
| 4345628 | 4350954 | LOC_Os08g33200.1 | LOC_Os11g40780.1 | 0.806 |
| 4345628 | 4350996 | LOC_Os08g33200.1 | LOC_Os11g42040.1 | 0.806 |
| 4345628 | 4351038 | LOC_Os08g33200.1 | LOC_Os11g43250.1 | 0.806 |
| 4345628 | 4351041 | LOC_Os08g33200.1 | LOC_Os11g43390.1 | 0.806 |
| 4345628 | 4351431 | LOC_Os08g33200.1 | LOC_Os12g03990.1 | 0.936 |
| 4345628 | 4351457 | LOC_Os08g33200.1 | LOC_Os12g04410.1 | 0.542 |
| 4345628 | 4351749 | LOC_Os08g33200.1 | LOC_Os12g10410.1 | 0.806 |
| 4345628 | 4352130 | LOC_Os08g33200.1 | LOC_Os12g25170.1 | 0.806 |
| 4345628 | 4352270 | LOC_Os08g33200.1 | LOC_Os12g31620.1 | 0.806 |
| 4345628 | 4352691 | LOC_Os08g33200.1 | LOC_Os12g40419.1 | 0.562 |
| 4345628 | 4352904 | LOC_Os08g33200.1 | LOC_Os12g44090.1 | 0.562 |
| 4345628 | IRL7 | LOC_Os08g33200.1 | LOC_Os03g11360.2 | 0.814 |
| 4345708 | 4324364 | LOC_Os08g34650.1 | LOC_Os01g69030.2 | 0.634 |
| 4345708 | 4324511 | LOC_Os08g34650.1 | LOC_Os01g05620.1 | 0.404 |
| 4345708 | 4324584 | LOC_Os08g34650.1 | LOC_Os01g11920.1 | 0.691 |
| 4345708 | 4324660 | LOC_Os08g34650.1 | LOC_Os01g52380.1 | 0.404 |
| 4345708 | 4324980 | LOC_Os08g34650.1 | LOC_Os01g67970.1 | 0.703 |
| 4345708 | 4325022 | LOC_Os08g34650.1 | LOC_Os01g67770.1 | 0.463 |
| 4345708 | 4326270 | LOC_Os08g34650.1 | LOC_Os01g16414.1 | 0.606 |
| 4345708 | 4326546 | LOC_Os08g34650.1 | LOC_Os01g53920.1 | 0.953 |
| 4345708 | 4327983 | LOC_Os08g34650.1 | LOC_Os02g01170.1 | 0.655 |
| 4345708 | 4328135 | LOC_Os08g34650.1 | LOC_Os02g03060.1 | 0.671 |
| 4345708 | 4328633 | LOC_Os08g34650.1 | LOC_Os02g10520.1 | 0.605 |
| 4345708 | 4329422 | LOC_Os08g34650.1 | LOC_Os02g29960.1 | 0.495 |
| 4345708 | 4330003 | LOC_Os08g34650.1 | LOC_Os02g40664.1 | 0.421 |
| 4345708 | 4331144 | LOC_Os08g34650.1 | LOC_Os02g57190.1 | 0.457 |
| 4345708 | 4331168 | LOC_Os08g34650.1 | LOC_Os02g57470.1 | 0.691 |
| 4345708 | 4332080 | LOC_Os08g34650.1 | LOC_Os03g11910.1 | 0.776 |
| 4345708 | 4333771 | LOC_Os08g34650.1 | LOC_Os03g48320.1 | 0.404 |
| 4345708 | 4334214 | LOC_Os08g34650.1 | LOC_Os03g55560.1 | 0.927 |
| 4345708 | 4334935 | LOC_Os08g34650.1 | LOC_Os04g02110.1 | 0.404 |
| 4345708 | 4335058 | LOC_Os08g34650.1 | LOC_Os04g08740.1 | 0.659 |
| 4345708 | 4335089 | LOC_Os08g34650.1 | LOC_Os04g09860.1 | 0.606 |
| 4345708 | 4336120 | LOC_Os08g34650.1 | LOC_Os04g39460.1 | 0.404 |
| 4345708 | 4337257 | LOC_Os08g34650.1 | LOC_Os04g56160.1 | 0.588 |
| 4345708 | 4337619 | LOC_Os08g34650.1 | LOC_Os05g02500.1 | 0.817 |
| 4345708 | 4337658 | LOC_Os08g34650.1 | LOC_Os05g03100.1 | 0.655 |
| 4345708 | 4337686 | LOC_Os08g34650.1 | LOC_Os05g03610.1 | 0.726 |
| 4345708 | 4338096 | LOC_Os08g34650.1 | LOC_Os05g11990.1 | 0.968 |
| 4345708 | 4338481 | LOC_Os08g34650.1 | LOC_Os05g27880.1 | 0.655 |
| 4345708 | 4338531 | LOC_Os08g34650.1 | LOC_Os05g29030.1 | 0.963 |
| 4345708 | 4339763 | LOC_Os08g34650.1 | LOC_Os05g50970.1 | 0.963 |
| 4345708 | 4340110 | LOC_Os08g34650.1 | LOC_Os06g05359.1 | 0.404 |
| 4345708 | 4340753 | LOC_Os08g34650.1 | LOC_Os06g16450.1 | 0.404 |
| 4345708 | 4340847 | LOC_Os08g34650.1 | LOC_Os06g20340.1 | 0.817 |
| 4345708 | 4340915 | LOC_Os08g34650.1 | LOC_Os06g22340.1 | 0.495 |
| 4345708 | 4340964 | LOC_Os08g34650.1 | LOC_Os06g23530.1 | 0.446 |
| 4345708 | 4341219 | LOC_Os08g34650.1 | LOC_Os06g34690.1 | 0.641 |
| 4345708 | 4341646 | LOC_Os08g34650.1 | LOC_Os06g43670.1 | 0.404 |
| 4345708 | 4341853 | LOC_Os08g34650.1 | LOC_Os06g46600.1 | 0.776 |
| 4345708 | 4341965 | LOC_Os08g34650.1 | LOC_Os06g48720.1 | 0.551 |
| 4345708 | 4342017 | LOC_Os08g34650.1 | LOC_Os06g49430.1 | 0.762 |
| 4345708 | 4343299 | LOC_Os08g34650.1 | LOC_Os07g31450.1 | 0.762 |
| 4345708 | 4343339 | LOC_Os08g34650.1 | LOC_Os07g32430.1 | 0.446 |
| 4345708 | 4343395 | LOC_Os08g34650.1 | LOC_Os07g33480.1 | 0.495 |
| 4345708 | 4343409 | LOC_Os08g34650.1 | LOC_Os07g33730.1 | 0.404 |
| 4345708 | 4344698 | LOC_Os08g34650.1 | LOC_Os08g06060.1 | 0.762 |
| 4345708 | 4345065 | LOC_Os08g34650.1 | LOC_Os08g14990.1 | 0.952 |
| 4345708 | 4345202 | LOC_Os08g34650.1 | LOC_Os08g20000.1 | 0.404 |
| 4345708 | 4345297 | LOC_Os08g34650.1 | LOC_Os08g24380.1 | 0.404 |
| 4345708 | 4345304 | LOC_Os08g34650.1 | LOC_Os08g24760.1 | 0.446 |
| 4345708 | 4345628 | LOC_Os08g34650.1 | LOC_Os08g33200.1 | 0.985 |
| 4345708 | 4345910 | LOC_Os08g34650.1 | LOC_Os08g38410.1 | 0.628 |
| 4345708 | 4346276 | LOC_Os08g34650.1 | LOC_Os08g44050.1 | 0.703 |
| 4345708 | 4346315 | LOC_Os08g34650.1 | LOC_Os08g44510.1 | 0.709 |
| 4345708 | 4347005 | LOC_Os08g34650.1 | LOC_Os09g23740.1 | 0.985 |
| 4345708 | 4347095 | LOC_Os08g34650.1 | LOC_Os09g25540.1 | 0.953 |
| 4345708 | 4347787 | LOC_Os08g34650.1 | LOC_Os09g37860.1 | 0.537 |
| 4345708 | 4348731 | LOC_Os08g34650.1 | LOC_Os10g30580.1 | 0.584 |
| 4345708 | 4348805 | LOC_Os08g34650.1 | LOC_Os10g31970.1 | 0.762 |
| 4345708 | 4348853 | LOC_Os08g34650.1 | LOC_Os10g32980.1 | 0.434 |
| 4345708 | 4349742 | LOC_Os08g34650.1 | LOC_Os11g04600.1 | 0.837 |
| 4345708 | 4350109 | LOC_Os08g34650.1 | LOC_Os11g11770.1 | 0.404 |
| 4345708 | 4350130 | LOC_Os08g34650.1 | LOC_Os11g12300.1 | 0.404 |
| 4345708 | 4350472 | LOC_Os08g34650.1 | LOC_Os11g27264.1 | 0.614 |
| 4345708 | 4350473 | LOC_Os08g34650.1 | LOC_Os11g27329.1 | 0.614 |
| 4345708 | 4350814 | LOC_Os08g34650.1 | LOC_Os11g37860.1 | 0.404 |
| 4345708 | 4350954 | LOC_Os08g34650.1 | LOC_Os11g40780.1 | 0.404 |
| 4345708 | 4350996 | LOC_Os08g34650.1 | LOC_Os11g42040.1 | 0.404 |
| 4345708 | 4351038 | LOC_Os08g34650.1 | LOC_Os11g43250.1 | 0.404 |
| 4345708 | 4351041 | LOC_Os08g34650.1 | LOC_Os11g43390.1 | 0.404 |
| 4345708 | 4351431 | LOC_Os08g34650.1 | LOC_Os12g03990.1 | 0.817 |
| 4345708 | 4351457 | LOC_Os08g34650.1 | LOC_Os12g04410.1 | 0.837 |
| 4345708 | 4351749 | LOC_Os08g34650.1 | LOC_Os12g10410.1 | 0.404 |
| 4345708 | 4352130 | LOC_Os08g34650.1 | LOC_Os12g25170.1 | 0.404 |
| 4345708 | 4352270 | LOC_Os08g34650.1 | LOC_Os12g31620.1 | 0.404 |
| 4345708 | LOC_Os02g49270.1 | LOC_Os08g34650.1 | LOC_Os02g49270.1 | 0.537 |
| 4345819 | 4332129 | LOC_Os08g36994.1 | LOC_Os03g12590.1 | 0.844 |
| 4345820 | 4351017 | LOC_Os08g37010.1 | LOC_Os11g42510.1 | 0.415 |
| 4345910 | 4326546 | LOC_Os08g38410.1 | LOC_Os01g53920.1 | 0.628 |
| 4345910 | 4330796 | LOC_Os08g38410.1 | LOC_Os02g52250.1 | 0.664 |
| 4345910 | 4338096 | LOC_Os08g38410.1 | LOC_Os05g11990.1 | 0.726 |
| 4345910 | 4340964 | LOC_Os08g38410.1 | LOC_Os06g23530.1 | 0.534 |
| 4345910 | 4342673 | LOC_Os08g38410.1 | LOC_Os07g10350.1 | 0.408 |
| 4345910 | 4345065 | LOC_Os08g38410.1 | LOC_Os08g14990.1 | 0.628 |
| 4345910 | 4345304 | LOC_Os08g38410.1 | LOC_Os08g24760.1 | 0.616 |
| 4345910 | 4345708 | LOC_Os08g38410.1 | LOC_Os08g34650.1 | 0.628 |
| 4345910 | 4347095 | LOC_Os08g38410.1 | LOC_Os09g25540.1 | 0.628 |
| 4345910 | 4348996 | LOC_Os08g38410.1 | LOC_Os10g35220.1 | 0.619 |
| 4346109 | 4324495 | LOC_Os08g41630.1 | LOC_Os01g05900.1 | 0.53 |
| 4346109 | 4324500 | LOC_Os08g41630.1 | LOC_Os01g05630.1 | 0.53 |
| 4346109 | 4327384 | LOC_Os08g41630.1 | LOC_Os01g62230.1 | 0.53 |
| 4346109 | 4337658 | LOC_Os08g41630.1 | LOC_Os05g03100.1 | 0.521 |
| 4346109 | H2B.9 | LOC_Os08g41630.1 | LOC_Os05g49860.1 | 0.53 |
| 4346208 | 4328135 | LOC_Os08g43090.1 | LOC_Os02g03060.1 | 0.434 |
| 4346276 | 4324584 | LOC_Os08g44050.1 | LOC_Os01g11920.1 | 0.478 |
| 4346276 | 4326239 | LOC_Os08g44050.1 | LOC_Os01g15480.1 | 0.458 |
| 4346276 | 4326270 | LOC_Os08g44050.1 | LOC_Os01g16414.1 | 0.549 |
| 4346276 | 4326546 | LOC_Os08g44050.1 | LOC_Os01g53920.1 | 0.703 |
| 4346276 | 4328135 | LOC_Os08g44050.1 | LOC_Os02g03060.1 | 0.612 |
| 4346276 | 4330484 | LOC_Os08g44050.1 | LOC_Os02g47970.1 | 0.459 |
| 4346276 | 4337063 | LOC_Os08g44050.1 | LOC_Os04g53410.1 | 0.454 |
| 4346276 | 4338096 | LOC_Os08g44050.1 | LOC_Os05g11990.1 | 0.474 |
| 4346276 | 4342017 | LOC_Os08g44050.1 | LOC_Os06g49430.1 | 0.693 |
| 4346276 | 4342410 | LOC_Os08g44050.1 | LOC_Os07g05620.1 | 0.41 |
| 4346276 | 4342472 | LOC_Os08g44050.1 | LOC_Os07g06980.1 | 0.496 |
| 4346276 | 4344698 | LOC_Os08g44050.1 | LOC_Os08g06060.1 | 0.693 |
| 4346276 | 4345065 | LOC_Os08g44050.1 | LOC_Os08g14990.1 | 0.703 |
| 4346276 | 4345708 | LOC_Os08g44050.1 | LOC_Os08g34650.1 | 0.703 |
| 4346276 | 4347095 | LOC_Os08g44050.1 | LOC_Os09g25540.1 | 0.703 |
| 4346276 | LOC_Os03g64080.1 | LOC_Os08g44050.1 | LOC_Os03g64080.1 | 0.76 |
| 4346315 | 4325329 | LOC_Os08g44510.1 | LOC_Os01g08700.1 | 0.644 |
| 4346315 | 4326546 | LOC_Os08g44510.1 | LOC_Os01g53920.1 | 0.709 |
| 4346315 | 4330701 | LOC_Os08g44510.1 | LOC_Os02g50970.1 | 0.406 |
| 4346315 | 4345065 | LOC_Os08g44510.1 | LOC_Os08g14990.1 | 0.709 |
| 4346315 | 4345708 | LOC_Os08g44510.1 | LOC_Os08g34650.1 | 0.709 |
| 4346315 | 4347095 | LOC_Os08g44510.1 | LOC_Os09g25540.1 | 0.709 |
| 4346508 | 4326439 | LOC_Os09g07570.1 | LOC_Os01g01302.1 | 0.477 |
| 4346508 | 4329037 | LOC_Os09g07570.1 | LOC_Os02g18450.1 | 0.592 |
| 4346508 | 4334795 | LOC_Os09g07570.1 | LOC_Os03g63360.1 | 0.426 |
| 4346508 | 4340177 | LOC_Os09g07570.1 | LOC_Os06g06190.1 | 0.537 |
| 4346508 | 4345365 | LOC_Os09g07570.1 | LOC_Os08g27010.1 | 0.858 |
| 4346699 | 4327790 | LOC_Os09g14670.1 | LOC_Os01g13570.1 | 0.445 |
| 4346699 | 4328828 | LOC_Os09g14670.1 | LOC_Os02g14110.1 | 0.441 |
| 4346699 | 4341252 | LOC_Os09g14670.1 | LOC_Os06g35540.1 | 0.441 |
| 4346808 | 4333493 | LOC_Os09g17810.1 | LOC_Os03g42750.1 | 0.855 |
| 4347005 | 4324511 | LOC_Os09g23740.1 | LOC_Os01g05620.1 | 0.806 |
| 4347005 | 4324660 | LOC_Os09g23740.1 | LOC_Os01g52380.1 | 0.806 |
| 4347005 | 4326270 | LOC_Os09g23740.1 | LOC_Os01g16414.1 | 0.514 |
| 4347005 | 4326546 | LOC_Os09g23740.1 | LOC_Os01g53920.1 | 0.985 |
| 4347005 | 4327667 | LOC_Os09g23740.1 | LOC_Os01g40590.1 | 0.562 |
| 4347005 | 4328725 | LOC_Os09g23740.1 | LOC_Os02g12440.1 | 0.562 |
| 4347005 | 4329448 | LOC_Os09g23740.1 | LOC_Os02g30620.1 | 0.636 |
| 4347005 | 4330701 | LOC_Os09g23740.1 | LOC_Os02g50970.1 | 0.562 |
| 4347005 | 4330971 | LOC_Os09g23740.1 | LOC_Os02g54820.1 | 0.511 |
| 4347005 | 4331134 | LOC_Os09g23740.1 | LOC_Os02g57080.1 | 0.562 |
| 4347005 | 4331991 | LOC_Os09g23740.1 | LOC_Os03g10780.1 | 0.4 |
| 4347005 | 4332080 | LOC_Os09g23740.1 | LOC_Os03g11910.1 | 0.88 |
| 4347005 | 4333060 | LOC_Os09g23740.1 | LOC_Os03g28300.1 | 0.562 |
| 4347005 | 4333771 | LOC_Os09g23740.1 | LOC_Os03g48320.1 | 0.806 |
| 4347005 | 4334214 | LOC_Os09g23740.1 | LOC_Os03g55560.1 | 0.408 |
| 4347005 | 4334921 | LOC_Os09g23740.1 | LOC_Os04g01874.1 | 0.562 |
| 4347005 | 4334935 | LOC_Os09g23740.1 | LOC_Os04g02110.1 | 0.806 |
| 4347005 | 4335089 | LOC_Os09g23740.1 | LOC_Os04g09860.1 | 0.514 |
| 4347005 | 4335824 | LOC_Os09g23740.1 | LOC_Os04g34250.1 | 0.562 |
| 4347005 | 4336120 | LOC_Os09g23740.1 | LOC_Os04g39460.1 | 0.806 |
| 4347005 | 4337251 | LOC_Os09g23740.1 | LOC_Os04g56090.1 | 0.562 |
| 4347005 | 4337619 | LOC_Os09g23740.1 | LOC_Os05g02500.1 | 0.936 |
| 4347005 | 4337686 | LOC_Os09g23740.1 | LOC_Os05g03610.1 | 0.946 |
| 4347005 | 4338080 | LOC_Os09g23740.1 | LOC_Os05g11750.1 | 0.562 |
| 4347005 | 4338096 | LOC_Os09g23740.1 | LOC_Os05g11990.1 | 0.677 |
| 4347005 | 4339142 | LOC_Os09g23740.1 | LOC_Os05g40770.1 | 0.562 |
| 4347005 | 4340110 | LOC_Os09g23740.1 | LOC_Os06g05359.1 | 0.806 |
| 4347005 | 4340753 | LOC_Os09g23740.1 | LOC_Os06g16450.1 | 0.806 |
| 4347005 | 4340847 | LOC_Os09g23740.1 | LOC_Os06g20340.1 | 0.936 |
| 4347005 | 4341066 | LOC_Os09g23740.1 | LOC_Os06g29080.1 | 0.562 |
| 4347005 | 4341568 | LOC_Os09g23740.1 | LOC_Os06g41980.1 | 0.562 |
| 4347005 | 4341646 | LOC_Os09g23740.1 | LOC_Os06g43670.1 | 0.806 |
| 4347005 | 4341853 | LOC_Os09g23740.1 | LOC_Os06g46600.1 | 0.88 |
| 4347005 | 4342017 | LOC_Os09g23740.1 | LOC_Os06g49430.1 | 0.519 |
| 4347005 | 4342396 | LOC_Os09g23740.1 | LOC_Os07g05370.1 | 0.562 |
| 4347005 | 4342410 | LOC_Os09g23740.1 | LOC_Os07g05620.1 | 0.578 |
| 4347005 | 4342694 | LOC_Os09g23740.1 | LOC_Os07g10630.1 | 0.803 |
| 4347005 | 4343196 | LOC_Os09g23740.1 | LOC_Os07g28800.1 | 0.636 |
| 4347005 | 4343409 | LOC_Os09g23740.1 | LOC_Os07g33730.1 | 0.806 |
| 4347005 | 4343699 | LOC_Os09g23740.1 | LOC_Os07g38810.1 | 0.562 |
| 4347005 | 4343832 | LOC_Os09g23740.1 | LOC_Os07g41140.1 | 0.562 |
| 4347005 | 4344698 | LOC_Os09g23740.1 | LOC_Os08g06060.1 | 0.519 |
| 4347005 | 4345065 | LOC_Os09g23740.1 | LOC_Os08g14990.1 | 0.985 |
| 4347005 | 4345202 | LOC_Os09g23740.1 | LOC_Os08g20000.1 | 0.806 |
| 4347005 | 4345297 | LOC_Os09g23740.1 | LOC_Os08g24380.1 | 0.806 |
| 4347005 | 4345628 | LOC_Os09g23740.1 | LOC_Os08g33200.1 | 0.895 |
| 4347005 | 4345708 | LOC_Os09g23740.1 | LOC_Os08g34650.1 | 0.985 |
| 4347005 | 4347095 | LOC_Os09g23740.1 | LOC_Os09g25540.1 | 0.985 |
| 4347005 | 4347814 | LOC_Os09g23740.1 | LOC_Os09g38330.1 | 0.401 |
| 4347005 | 4349742 | LOC_Os09g23740.1 | LOC_Os11g04600.1 | 0.542 |
| 4347005 | 4350109 | LOC_Os09g23740.1 | LOC_Os11g11770.1 | 0.806 |
| 4347005 | 4350130 | LOC_Os09g23740.1 | LOC_Os11g12300.1 | 0.806 |
| 4347005 | 4350352 | LOC_Os09g23740.1 | LOC_Os11g20384.1 | 0.964 |
| 4347005 | 4350814 | LOC_Os09g23740.1 | LOC_Os11g37860.1 | 0.806 |
| 4347005 | 4350954 | LOC_Os09g23740.1 | LOC_Os11g40780.1 | 0.806 |
| 4347005 | 4350996 | LOC_Os09g23740.1 | LOC_Os11g42040.1 | 0.806 |
| 4347005 | 4351038 | LOC_Os09g23740.1 | LOC_Os11g43250.1 | 0.806 |
| 4347005 | 4351041 | LOC_Os09g23740.1 | LOC_Os11g43390.1 | 0.806 |
| 4347005 | 4351431 | LOC_Os09g23740.1 | LOC_Os12g03990.1 | 0.936 |
| 4347005 | 4351457 | LOC_Os09g23740.1 | LOC_Os12g04410.1 | 0.542 |
| 4347005 | 4351749 | LOC_Os09g23740.1 | LOC_Os12g10410.1 | 0.806 |
| 4347005 | 4352130 | LOC_Os09g23740.1 | LOC_Os12g25170.1 | 0.806 |
| 4347005 | 4352270 | LOC_Os09g23740.1 | LOC_Os12g31620.1 | 0.806 |
| 4347005 | 4352691 | LOC_Os09g23740.1 | LOC_Os12g40419.1 | 0.562 |
| 4347005 | 4352904 | LOC_Os09g23740.1 | LOC_Os12g44090.1 | 0.562 |
| 4347005 | IRL7 | LOC_Os09g23740.1 | LOC_Os03g11360.2 | 0.814 |
| 4347095 | 4324364 | LOC_Os09g25540.1 | LOC_Os01g69030.2 | 0.634 |
| 4347095 | 4324511 | LOC_Os09g25540.1 | LOC_Os01g05620.1 | 0.404 |
| 4347095 | 4324584 | LOC_Os09g25540.1 | LOC_Os01g11920.1 | 0.691 |
| 4347095 | 4324660 | LOC_Os09g25540.1 | LOC_Os01g52380.1 | 0.404 |
| 4347095 | 4324980 | LOC_Os09g25540.1 | LOC_Os01g67970.1 | 0.703 |
| 4347095 | 4325022 | LOC_Os09g25540.1 | LOC_Os01g67770.1 | 0.463 |
| 4347095 | 4326270 | LOC_Os09g25540.1 | LOC_Os01g16414.1 | 0.606 |
| 4347095 | 4326546 | LOC_Os09g25540.1 | LOC_Os01g53920.1 | 0.956 |
| 4347095 | 4327983 | LOC_Os09g25540.1 | LOC_Os02g01170.1 | 0.655 |
| 4347095 | 4328135 | LOC_Os09g25540.1 | LOC_Os02g03060.1 | 0.673 |
| 4347095 | 4328633 | LOC_Os09g25540.1 | LOC_Os02g10520.1 | 0.605 |
| 4347095 | 4329422 | LOC_Os09g25540.1 | LOC_Os02g29960.1 | 0.495 |
| 4347095 | 4330003 | LOC_Os09g25540.1 | LOC_Os02g40664.1 | 0.421 |
| 4347095 | 4331144 | LOC_Os09g25540.1 | LOC_Os02g57190.1 | 0.457 |
| 4347095 | 4331168 | LOC_Os09g25540.1 | LOC_Os02g57470.1 | 0.691 |
| 4347095 | 4332080 | LOC_Os09g25540.1 | LOC_Os03g11910.1 | 0.776 |
| 4347095 | 4334214 | LOC_Os09g25540.1 | LOC_Os03g55560.1 | 0.927 |
| 4347095 | 4334935 | LOC_Os09g25540.1 | LOC_Os04g02110.1 | 0.404 |
| 4347095 | 4335058 | LOC_Os09g25540.1 | LOC_Os04g08740.1 | 0.659 |
| 4347095 | 4335089 | LOC_Os09g25540.1 | LOC_Os04g09860.1 | 0.606 |
| 4347095 | 4336120 | LOC_Os09g25540.1 | LOC_Os04g39460.1 | 0.404 |
| 4347095 | 4337257 | LOC_Os09g25540.1 | LOC_Os04g56160.1 | 0.588 |
| 4347095 | 4337619 | LOC_Os09g25540.1 | LOC_Os05g02500.1 | 0.817 |
| 4347095 | 4337658 | LOC_Os09g25540.1 | LOC_Os05g03100.1 | 0.655 |
| 4347095 | 4337686 | LOC_Os09g25540.1 | LOC_Os05g03610.1 | 0.726 |
| 4347095 | 4338096 | LOC_Os09g25540.1 | LOC_Os05g11990.1 | 0.968 |
| 4347095 | 4338481 | LOC_Os09g25540.1 | LOC_Os05g27880.1 | 0.655 |
| 4347095 | 4338531 | LOC_Os09g25540.1 | LOC_Os05g29030.1 | 0.963 |
| 4347095 | 4339763 | LOC_Os09g25540.1 | LOC_Os05g50970.1 | 0.963 |
| 4347095 | 4340110 | LOC_Os09g25540.1 | LOC_Os06g05359.1 | 0.404 |
| 4347095 | 4340753 | LOC_Os09g25540.1 | LOC_Os06g16450.1 | 0.404 |
| 4347095 | 4340847 | LOC_Os09g25540.1 | LOC_Os06g20340.1 | 0.817 |
| 4347095 | 4340915 | LOC_Os09g25540.1 | LOC_Os06g22340.1 | 0.495 |
| 4347095 | 4340964 | LOC_Os09g25540.1 | LOC_Os06g23530.1 | 0.446 |
| 4347095 | 4341219 | LOC_Os09g25540.1 | LOC_Os06g34690.1 | 0.641 |
| 4347095 | 4341853 | LOC_Os09g25540.1 | LOC_Os06g46600.1 | 0.776 |
| 4347095 | 4341965 | LOC_Os09g25540.1 | LOC_Os06g48720.1 | 0.551 |
| 4347095 | 4342017 | LOC_Os09g25540.1 | LOC_Os06g49430.1 | 0.764 |
| 4347095 | 4343299 | LOC_Os09g25540.1 | LOC_Os07g31450.1 | 0.762 |
| 4347095 | 4343339 | LOC_Os09g25540.1 | LOC_Os07g32430.1 | 0.446 |
| 4347095 | 4343395 | LOC_Os09g25540.1 | LOC_Os07g33480.1 | 0.495 |
| 4347095 | 4343409 | LOC_Os09g25540.1 | LOC_Os07g33730.1 | 0.404 |
| 4347095 | 4344698 | LOC_Os09g25540.1 | LOC_Os08g06060.1 | 0.846 |
| 4347095 | 4345065 | LOC_Os09g25540.1 | LOC_Os08g14990.1 | 0.951 |
| 4347095 | 4345202 | LOC_Os09g25540.1 | LOC_Os08g20000.1 | 0.404 |
| 4347095 | 4345297 | LOC_Os09g25540.1 | LOC_Os08g24380.1 | 0.404 |
| 4347095 | 4345304 | LOC_Os09g25540.1 | LOC_Os08g24760.1 | 0.446 |
| 4347095 | 4345628 | LOC_Os09g25540.1 | LOC_Os08g33200.1 | 0.985 |
| 4347095 | 4345708 | LOC_Os09g25540.1 | LOC_Os08g34650.1 | 0.953 |
| 4347095 | 4345910 | LOC_Os09g25540.1 | LOC_Os08g38410.1 | 0.628 |
| 4347095 | 4346276 | LOC_Os09g25540.1 | LOC_Os08g44050.1 | 0.703 |
| 4347095 | 4346315 | LOC_Os09g25540.1 | LOC_Os08g44510.1 | 0.709 |
| 4347095 | 4347005 | LOC_Os09g25540.1 | LOC_Os09g23740.1 | 0.985 |
| 4347095 | 4347787 | LOC_Os09g25540.1 | LOC_Os09g37860.1 | 0.537 |
| 4347095 | 4348731 | LOC_Os09g25540.1 | LOC_Os10g30580.1 | 0.584 |
| 4347095 | 4348805 | LOC_Os09g25540.1 | LOC_Os10g31970.1 | 0.762 |
| 4347095 | 4348853 | LOC_Os09g25540.1 | LOC_Os10g32980.1 | 0.434 |
| 4347095 | 4349742 | LOC_Os09g25540.1 | LOC_Os11g04600.1 | 0.837 |
| 4347095 | 4350109 | LOC_Os09g25540.1 | LOC_Os11g11770.1 | 0.404 |
| 4347095 | 4350130 | LOC_Os09g25540.1 | LOC_Os11g12300.1 | 0.404 |
| 4347095 | 4350472 | LOC_Os09g25540.1 | LOC_Os11g27264.1 | 0.614 |
| 4347095 | 4350473 | LOC_Os09g25540.1 | LOC_Os11g27329.1 | 0.614 |
| 4347095 | 4350814 | LOC_Os09g25540.1 | LOC_Os11g37860.1 | 0.404 |
| 4347095 | 4350954 | LOC_Os09g25540.1 | LOC_Os11g40780.1 | 0.404 |
| 4347095 | 4350996 | LOC_Os09g25540.1 | LOC_Os11g42040.1 | 0.404 |
| 4347095 | 4351038 | LOC_Os09g25540.1 | LOC_Os11g43250.1 | 0.404 |
| 4347095 | 4351041 | LOC_Os09g25540.1 | LOC_Os11g43390.1 | 0.404 |
| 4347095 | 4351431 | LOC_Os09g25540.1 | LOC_Os12g03990.1 | 0.817 |
| 4347095 | 4351457 | LOC_Os09g25540.1 | LOC_Os12g04410.1 | 0.837 |
| 4347095 | 4351749 | LOC_Os09g25540.1 | LOC_Os12g10410.1 | 0.404 |
| 4347095 | 4352130 | LOC_Os09g25540.1 | LOC_Os12g25170.1 | 0.404 |
| 4347095 | 4352270 | LOC_Os09g25540.1 | LOC_Os12g31620.1 | 0.404 |
| 4347095 | LOC_Os02g49270.1 | LOC_Os09g25540.1 | LOC_Os02g49270.1 | 0.537 |
| 4347172 | 4328828 | LOC_Os09g26880.1 | LOC_Os02g14110.1 | 0.438 |
| 4347172 | 4330971 | LOC_Os09g26880.1 | LOC_Os02g54820.1 | 0.457 |
| 4347172 | 4332628 | LOC_Os09g26880.1 | LOC_Os03g19680.1 | 0.557 |
| 4347172 | 4334290 | LOC_Os09g26880.1 | LOC_Os03g56460.1 | 0.566 |
| 4347172 | 4338096 | LOC_Os09g26880.1 | LOC_Os05g11990.1 | 0.501 |
| 4347172 | 4341252 | LOC_Os09g26880.1 | LOC_Os06g35540.1 | 0.438 |
| 4347172 | 4343395 | LOC_Os09g26880.1 | LOC_Os07g33480.1 | 0.456 |
| 4347172 | 4344267 | LOC_Os09g26880.1 | LOC_Os07g47820.1 | 0.929 |
| 4347172 | 4347311 | LOC_Os09g26880.1 | LOC_Os09g29070.1 | 0.566 |
| 4347172 | YUCCA1 | LOC_Os09g26880.1 | LOC_Os01g45760.1 | 0.863 |
| 4347221 | 4328135 | LOC_Os09g27700.1 | LOC_Os02g03060.1 | 0.717 |
| 4347221 | 4330866 | LOC_Os09g27700.1 | LOC_Os02g53120.1 | 0.687 |
| 4347221 | 4337886 | LOC_Os09g27700.1 | LOC_Os05g06840.1 | 0.585 |
| 4347221 | 4343388 | LOC_Os09g27700.1 | LOC_Os07g33370.1 | 0.732 |
| 4347311 | 4327790 | LOC_Os09g29070.1 | LOC_Os01g13570.1 | 0.616 |
| 4347311 | 4328828 | LOC_Os09g29070.1 | LOC_Os02g14110.1 | 0.566 |
| 4347311 | 4330709 | LOC_Os09g29070.1 | LOC_Os02g51070.1 | 0.549 |
| 4347311 | 4330971 | LOC_Os09g29070.1 | LOC_Os02g54820.1 | 0.53 |
| 4347311 | 4332258 | LOC_Os09g29070.1 | LOC_Os03g14540.1 | 0.46 |
| 4347311 | 4334290 | LOC_Os09g29070.1 | LOC_Os03g56460.1 | 0.938 |
| 4347311 | 4336977 | LOC_Os09g29070.1 | LOC_Os04g52370.1 | 0.529 |
| 4347311 | 4338096 | LOC_Os09g29070.1 | LOC_Os05g11990.1 | 0.432 |
| 4347311 | 4341219 | LOC_Os09g29070.1 | LOC_Os06g34690.1 | 0.427 |
| 4347311 | 4341252 | LOC_Os09g29070.1 | LOC_Os06g35540.1 | 0.566 |
| 4347311 | 4342770 | LOC_Os09g29070.1 | LOC_Os07g12640.1 | 0.497 |
| 4347311 | 4344584 | LOC_Os09g29070.1 | LOC_Os08g03570.1 | 0.46 |
| 4347311 | 4347172 | LOC_Os09g29070.1 | LOC_Os09g26880.1 | 0.566 |
| 4347311 | 4352803 | LOC_Os09g29070.1 | LOC_Os12g42230.1 | 0.948 |
| 4347401 | 4337356 | LOC_Os09g30411.1 | LOC_Os04g57520.1 | 0.524 |
| 4347401 | 4337658 | LOC_Os09g30411.1 | LOC_Os05g03100.1 | 0.506 |
| 4347401 | 4348731 | LOC_Os09g30411.1 | LOC_Os10g30580.1 | 0.96 |
| 4347677 | 4330868 | LOC_Os09g36240.1 | LOC_Os02g53140.1 | 0.582 |
| 4347677 | 4337178 | LOC_Os09g36240.1 | LOC_Os04g55060.1 | 0.438 |
| 4347677 | 4344329 | LOC_Os09g36240.1 | LOC_Os07g48610.1 | 0.581 |
| 4347787 | 4326546 | LOC_Os09g37860.1 | LOC_Os01g53920.1 | 0.537 |
| 4347787 | 4327667 | LOC_Os09g37860.1 | LOC_Os01g40590.1 | 0.408 |
| 4347787 | 4328725 | LOC_Os09g37860.1 | LOC_Os02g12440.1 | 0.408 |
| 4347787 | 4330701 | LOC_Os09g37860.1 | LOC_Os02g50970.1 | 0.408 |
| 4347787 | 4331005 | LOC_Os09g37860.1 | LOC_Os02g55260.1 | 0.716 |
| 4347787 | 4331134 | LOC_Os09g37860.1 | LOC_Os02g57080.1 | 0.408 |
| 4347787 | 4331168 | LOC_Os09g37860.1 | LOC_Os02g57470.1 | 0.402 |
| 4347787 | 4331367 | LOC_Os09g37860.1 | LOC_Os03g02110.1 | 0.491 |
| 4347787 | 4332049 | LOC_Os09g37860.1 | LOC_Os03g11510.1 | 0.483 |
| 4347787 | 4332567 | LOC_Os09g37860.1 | LOC_Os03g18840.1 | 0.909 |
| 4347787 | 4333060 | LOC_Os09g37860.1 | LOC_Os03g28300.1 | 0.408 |
| 4347787 | 4333574 | LOC_Os09g37860.1 | LOC_Os03g44530.1 | 0.866 |
| 4347787 | 4333814 | LOC_Os09g37860.1 | LOC_Os03g49210.1 | 0.998 |
| 4347787 | 4334921 | LOC_Os09g37860.1 | LOC_Os04g01874.1 | 0.408 |
| 4347787 | 4335824 | LOC_Os09g37860.1 | LOC_Os04g34250.1 | 0.408 |
| 4347787 | 4336833 | LOC_Os09g37860.1 | LOC_Os04g50660.1 | 0.966 |
| 4347787 | 4337251 | LOC_Os09g37860.1 | LOC_Os04g56090.1 | 0.408 |
| 4347787 | 4338080 | LOC_Os09g37860.1 | LOC_Os05g11750.1 | 0.443 |
| 4347787 | 4338096 | LOC_Os09g37860.1 | LOC_Os05g11990.1 | 0.4 |
| 4347787 | 4338531 | LOC_Os09g37860.1 | LOC_Os05g29030.1 | 0.404 |
| 4347787 | 4339142 | LOC_Os09g37860.1 | LOC_Os05g40770.1 | 0.408 |
| 4347787 | 4339763 | LOC_Os09g37860.1 | LOC_Os05g50970.1 | 0.404 |
| 4347787 | 4341066 | LOC_Os09g37860.1 | LOC_Os06g29080.1 | 0.408 |
| 4347787 | 4341219 | LOC_Os09g37860.1 | LOC_Os06g34690.1 | 0.468 |
| 4347787 | 4341568 | LOC_Os09g37860.1 | LOC_Os06g41980.1 | 0.408 |
| 4347787 | 4342396 | LOC_Os09g37860.1 | LOC_Os07g05370.1 | 0.408 |
| 4347787 | 4342410 | LOC_Os09g37860.1 | LOC_Os07g05620.1 | 0.408 |
| 4347787 | 4342673 | LOC_Os09g37860.1 | LOC_Os07g10350.1 | 0.997 |
| 4347787 | 4343699 | LOC_Os09g37860.1 | LOC_Os07g38810.1 | 0.408 |
| 4347787 | 4343832 | LOC_Os09g37860.1 | LOC_Os07g41140.1 | 0.408 |
| 4347787 | 4344935 | LOC_Os09g37860.1 | LOC_Os08g10608.1 | 0.554 |
| 4347787 | 4345065 | LOC_Os09g37860.1 | LOC_Os08g14990.1 | 0.537 |
| 4347787 | 4345708 | LOC_Os09g37860.1 | LOC_Os08g34650.1 | 0.537 |
| 4347787 | 4347095 | LOC_Os09g37860.1 | LOC_Os09g25540.1 | 0.537 |
| 4347787 | 4350837 | LOC_Os09g37860.1 | LOC_Os11g38170.1 | 0.743 |
| 4347787 | 4352691 | LOC_Os09g37860.1 | LOC_Os12g40419.1 | 0.408 |
| 4347787 | 4352904 | LOC_Os09g37860.1 | LOC_Os12g44090.1 | 0.408 |
| 4347787 | LOC_Os02g49270.1 | LOC_Os09g37860.1 | LOC_Os02g49270.1 | 0.837 |
| 4347809 | 4329422 | LOC_Os09g38239.1 | LOC_Os02g29960.1 | 0.403 |
| 4347809 | 4340373 | LOC_Os09g38239.1 | LOC_Os06g09240.1 | 0.408 |
| 4347809 | 4340915 | LOC_Os09g38239.1 | LOC_Os06g22340.1 | 0.403 |
| 4347809 | 4343352 | LOC_Os09g38239.1 | LOC_Os07g32620.1 | 0.408 |
| 4347809 | 4343395 | LOC_Os09g38239.1 | LOC_Os07g33480.1 | 0.402 |
| 4347814 | 4345628 | LOC_Os09g38330.1 | LOC_Os08g33200.1 | 0.401 |
| 4347814 | 4347005 | LOC_Os09g38330.1 | LOC_Os09g23740.1 | 0.401 |
| 4347823 | 4326239 | LOC_Os09g38450.1 | LOC_Os01g15480.1 | 0.425 |
| 4347823 | 4332784 | LOC_Os09g38450.1 | LOC_Os03g22040.1 | 0.428 |
| 4347823 | 4339099 | LOC_Os09g38450.1 | LOC_Os05g39850.1 | 0.484 |
| 4347823 | 4342995 | LOC_Os09g38450.1 | LOC_Os07g22580.1 | 0.67 |
| 4347823 | 4343388 | LOC_Os09g38450.1 | LOC_Os07g33370.1 | 0.589 |
| 4347935 | 4332040 | LOC_Os10g01044.1 | LOC_Os03g11410.1 | 0.565 |
| 4347935 | 4343665 | LOC_Os10g01044.1 | LOC_Os07g38360.2 | 0.493 |
| 4348486 | 4337178 | LOC_Os10g22960.1 | LOC_Os04g55060.1 | 0.438 |
| 4348561 | 4324584 | LOC_Os10g26010.1 | LOC_Os01g11920.1 | 0.433 |
| 4348561 | 4328828 | LOC_Os10g26010.1 | LOC_Os02g14110.1 | 0.428 |
| 4348561 | 4338096 | LOC_Os10g26010.1 | LOC_Os05g11990.1 | 0.485 |
| 4348561 | 4341252 | LOC_Os10g26010.1 | LOC_Os06g35540.1 | 0.428 |
| 4348561 | 4344612 | LOC_Os10g26010.1 | LOC_Os08g04180.1 | 0.453 |
| 4348561 | 4351017 | LOC_Os10g26010.1 | LOC_Os11g42510.1 | 0.493 |
| 4348731 | 4326546 | LOC_Os10g30580.1 | LOC_Os01g53920.1 | 0.584 |
| 4348731 | 4328287 | LOC_Os10g30580.1 | LOC_Os02g05340.1 | 0.748 |
| 4348731 | 4330866 | LOC_Os10g30580.1 | LOC_Os02g53120.1 | 0.629 |
| 4348731 | 4332080 | LOC_Os10g30580.1 | LOC_Os03g11910.1 | 0.551 |
| 4348731 | 4337356 | LOC_Os10g30580.1 | LOC_Os04g57520.1 | 0.929 |
| 4348731 | 4337658 | LOC_Os10g30580.1 | LOC_Os05g03100.1 | 0.588 |
| 4348731 | 4338096 | LOC_Os10g30580.1 | LOC_Os05g11990.1 | 0.686 |
| 4348731 | 4341853 | LOC_Os10g30580.1 | LOC_Os06g46600.1 | 0.456 |
| 4348731 | 4345065 | LOC_Os10g30580.1 | LOC_Os08g14990.1 | 0.584 |
| 4348731 | 4345708 | LOC_Os10g30580.1 | LOC_Os08g34650.1 | 0.584 |
| 4348731 | 4347095 | LOC_Os10g30580.1 | LOC_Os09g25540.1 | 0.584 |
| 4348731 | 4347401 | LOC_Os10g30580.1 | LOC_Os09g30411.1 | 0.96 |
| 4348805 | 4326270 | LOC_Os10g31970.1 | LOC_Os01g16414.1 | 0.435 |
| 4348805 | 4326546 | LOC_Os10g31970.1 | LOC_Os01g53920.1 | 0.762 |
| 4348805 | 4335089 | LOC_Os10g31970.1 | LOC_Os04g09860.1 | 0.435 |
| 4348805 | 4337317 | LOC_Os10g31970.1 | LOC_Os04g56980.1 | 0.529 |
| 4348805 | 4343276 | LOC_Os10g31970.1 | LOC_Os07g30980.1 | 0.459 |
| 4348805 | 4344306 | LOC_Os10g31970.1 | LOC_Os07g48360.1 | 0.582 |
| 4348805 | 4345065 | LOC_Os10g31970.1 | LOC_Os08g14990.1 | 0.762 |
| 4348805 | 4345708 | LOC_Os10g31970.1 | LOC_Os08g34650.1 | 0.762 |
| 4348805 | 4347095 | LOC_Os10g31970.1 | LOC_Os09g25540.1 | 0.762 |
| 4348805 | 4352699 | LOC_Os10g31970.1 | LOC_Os12g40490.1 | 0.584 |
| 4348853 | 4324364 | LOC_Os10g32980.1 | LOC_Os01g69030.2 | 0.519 |
| 4348853 | 4326546 | LOC_Os10g32980.1 | LOC_Os01g53920.1 | 0.434 |
| 4348853 | 4332258 | LOC_Os10g32980.1 | LOC_Os03g14540.1 | 0.485 |
| 4348853 | 4334029 | LOC_Os10g32980.1 | LOC_Os03g52630.1 | 0.593 |
| 4348853 | 4336977 | LOC_Os10g32980.1 | LOC_Os04g52370.1 | 0.584 |
| 4348853 | 4338096 | LOC_Os10g32980.1 | LOC_Os05g11990.1 | 0.558 |
| 4348853 | 4343352 | LOC_Os10g32980.1 | LOC_Os07g32620.1 | 0.445 |
| 4348853 | 4344584 | LOC_Os10g32980.1 | LOC_Os08g03570.1 | 0.485 |
| 4348853 | 4345065 | LOC_Os10g32980.1 | LOC_Os08g14990.1 | 0.434 |
| 4348853 | 4345708 | LOC_Os10g32980.1 | LOC_Os08g34650.1 | 0.434 |
| 4348853 | 4347095 | LOC_Os10g32980.1 | LOC_Os09g25540.1 | 0.434 |
| 4348919 | 4331253 | LOC_Os10g33960.1 | LOC_Os02g58490.1 | 0.401 |
| 4348996 | 4345910 | LOC_Os10g35220.1 | LOC_Os08g38410.1 | 0.619 |
| 4349306 | 4326830 | LOC_Os10g40140.1 | LOC_Os01g70790.1 | 0.445 |
| 4349490 | 4341853 | LOC_Os10g42510.1 | LOC_Os06g46600.1 | 0.487 |
| 4349531 | 4330868 | LOC_Os11g01140.1 | LOC_Os02g53140.1 | 0.514 |
| 4349531 | 4335058 | LOC_Os11g01140.1 | LOC_Os04g08740.1 | 0.522 |
| 4349719 | 4328287 | LOC_Os11g04220.1 | LOC_Os02g05340.1 | 0.792 |
| 4349719 | 4332129 | LOC_Os11g04220.1 | LOC_Os03g12590.1 | 0.595 |
| 4349742 | 4324584 | LOC_Os11g04600.1 | LOC_Os01g11920.1 | 0.595 |
| 4349742 | 4326546 | LOC_Os11g04600.1 | LOC_Os01g53920.1 | 0.838 |
| 4349742 | 4327667 | LOC_Os11g04600.1 | LOC_Os01g40590.1 | 0.423 |
| 4349742 | 4328135 | LOC_Os11g04600.1 | LOC_Os02g03060.1 | 0.678 |
| 4349742 | 4331134 | LOC_Os11g04600.1 | LOC_Os02g57080.1 | 0.423 |
| 4349742 | 4338080 | LOC_Os11g04600.1 | LOC_Os05g11750.1 | 0.423 |
| 4349742 | 4338096 | LOC_Os11g04600.1 | LOC_Os05g11990.1 | 0.79 |
| 4349742 | 4341066 | LOC_Os11g04600.1 | LOC_Os06g29080.1 | 0.423 |
| 4349742 | 4342017 | LOC_Os11g04600.1 | LOC_Os06g49430.1 | 0.476 |
| 4349742 | 4342396 | LOC_Os11g04600.1 | LOC_Os07g05370.1 | 0.423 |
| 4349742 | 4342410 | LOC_Os11g04600.1 | LOC_Os07g05620.1 | 0.421 |
| 4349742 | 4344698 | LOC_Os11g04600.1 | LOC_Os08g06060.1 | 0.476 |
| 4349742 | 4345065 | LOC_Os11g04600.1 | LOC_Os08g14990.1 | 0.837 |
| 4349742 | 4345628 | LOC_Os11g04600.1 | LOC_Os08g33200.1 | 0.542 |
| 4349742 | 4345708 | LOC_Os11g04600.1 | LOC_Os08g34650.1 | 0.837 |
| 4349742 | 4347005 | LOC_Os11g04600.1 | LOC_Os09g23740.1 | 0.542 |
| 4349742 | 4347095 | LOC_Os11g04600.1 | LOC_Os09g25540.1 | 0.837 |
| 4349742 | 4352691 | LOC_Os11g04600.1 | LOC_Os12g40419.1 | 0.423 |
| 4349776 | 4344819 | LOC_Os11g05130.1 | LOC_Os08g08210.1 | 0.497 |
| 4349875 | 4333493 | LOC_Os11g06700.1 | LOC_Os03g42750.1 | 0.984 |
| 4349939 | 4330868 | LOC_Os11g07910.1 | LOC_Os02g53140.1 | 0.595 |
| 4349939 | 4332129 | LOC_Os11g07910.1 | LOC_Os03g12590.1 | 0.492 |
| 4350049 | 4338096 | LOC_Os11g10430.1 | LOC_Os05g11990.1 | 0.56 |
| 4350109 | 4326546 | LOC_Os11g11770.1 | LOC_Os01g53920.1 | 0.404 |
| 4350109 | 4327667 | LOC_Os11g11770.1 | LOC_Os01g40590.1 | 0.404 |
| 4350109 | 4328725 | LOC_Os11g11770.1 | LOC_Os02g12440.1 | 0.404 |
| 4350109 | 4330701 | LOC_Os11g11770.1 | LOC_Os02g50970.1 | 0.404 |
| 4350109 | 4331134 | LOC_Os11g11770.1 | LOC_Os02g57080.1 | 0.404 |
| 4350109 | 4333060 | LOC_Os11g11770.1 | LOC_Os03g28300.1 | 0.404 |
| 4350109 | 4334921 | LOC_Os11g11770.1 | LOC_Os04g01874.1 | 0.404 |
| 4350109 | 4335824 | LOC_Os11g11770.1 | LOC_Os04g34250.1 | 0.404 |
| 4350109 | 4337251 | LOC_Os11g11770.1 | LOC_Os04g56090.1 | 0.404 |
| 4350109 | 4338080 | LOC_Os11g11770.1 | LOC_Os05g11750.1 | 0.404 |
| 4350109 | 4338096 | LOC_Os11g11770.1 | LOC_Os05g11990.1 | 0.628 |
| 4350109 | 4339142 | LOC_Os11g11770.1 | LOC_Os05g40770.1 | 0.404 |
| 4350109 | 4341066 | LOC_Os11g11770.1 | LOC_Os06g29080.1 | 0.404 |
| 4350109 | 4341568 | LOC_Os11g11770.1 | LOC_Os06g41980.1 | 0.404 |
| 4350109 | 4342396 | LOC_Os11g11770.1 | LOC_Os07g05370.1 | 0.404 |
| 4350109 | 4342410 | LOC_Os11g11770.1 | LOC_Os07g05620.1 | 0.404 |
| 4350109 | 4343699 | LOC_Os11g11770.1 | LOC_Os07g38810.1 | 0.404 |
| 4350109 | 4343832 | LOC_Os11g11770.1 | LOC_Os07g41140.1 | 0.404 |
| 4350109 | 4345065 | LOC_Os11g11770.1 | LOC_Os08g14990.1 | 0.404 |
| 4350109 | 4345628 | LOC_Os11g11770.1 | LOC_Os08g33200.1 | 0.806 |
| 4350109 | 4345708 | LOC_Os11g11770.1 | LOC_Os08g34650.1 | 0.404 |
| 4350109 | 4347005 | LOC_Os11g11770.1 | LOC_Os09g23740.1 | 0.806 |
| 4350109 | 4347095 | LOC_Os11g11770.1 | LOC_Os09g25540.1 | 0.404 |
| 4350109 | 4352691 | LOC_Os11g11770.1 | LOC_Os12g40419.1 | 0.404 |
| 4350109 | 4352904 | LOC_Os11g11770.1 | LOC_Os12g44090.1 | 0.404 |
| 4350130 | 4326546 | LOC_Os11g12300.1 | LOC_Os01g53920.1 | 0.404 |
| 4350130 | 4327667 | LOC_Os11g12300.1 | LOC_Os01g40590.1 | 0.404 |
| 4350130 | 4328725 | LOC_Os11g12300.1 | LOC_Os02g12440.1 | 0.404 |
| 4350130 | 4330701 | LOC_Os11g12300.1 | LOC_Os02g50970.1 | 0.404 |
| 4350130 | 4331134 | LOC_Os11g12300.1 | LOC_Os02g57080.1 | 0.404 |
| 4350130 | 4333060 | LOC_Os11g12300.1 | LOC_Os03g28300.1 | 0.404 |
| 4350130 | 4334921 | LOC_Os11g12300.1 | LOC_Os04g01874.1 | 0.404 |
| 4350130 | 4335824 | LOC_Os11g12300.1 | LOC_Os04g34250.1 | 0.404 |
| 4350130 | 4337251 | LOC_Os11g12300.1 | LOC_Os04g56090.1 | 0.404 |
| 4350130 | 4338080 | LOC_Os11g12300.1 | LOC_Os05g11750.1 | 0.404 |
| 4350130 | 4338096 | LOC_Os11g12300.1 | LOC_Os05g11990.1 | 0.628 |
| 4350130 | 4339142 | LOC_Os11g12300.1 | LOC_Os05g40770.1 | 0.404 |
| 4350130 | 4341066 | LOC_Os11g12300.1 | LOC_Os06g29080.1 | 0.404 |
| 4350130 | 4341568 | LOC_Os11g12300.1 | LOC_Os06g41980.1 | 0.404 |
| 4350130 | 4342396 | LOC_Os11g12300.1 | LOC_Os07g05370.1 | 0.404 |
| 4350130 | 4342410 | LOC_Os11g12300.1 | LOC_Os07g05620.1 | 0.404 |
| 4350130 | 4343699 | LOC_Os11g12300.1 | LOC_Os07g38810.1 | 0.404 |
| 4350130 | 4343832 | LOC_Os11g12300.1 | LOC_Os07g41140.1 | 0.404 |
| 4350130 | 4345065 | LOC_Os11g12300.1 | LOC_Os08g14990.1 | 0.404 |
| 4350130 | 4345628 | LOC_Os11g12300.1 | LOC_Os08g33200.1 | 0.806 |
| 4350130 | 4345708 | LOC_Os11g12300.1 | LOC_Os08g34650.1 | 0.404 |
| 4350130 | 4347005 | LOC_Os11g12300.1 | LOC_Os09g23740.1 | 0.806 |
| 4350130 | 4347095 | LOC_Os11g12300.1 | LOC_Os09g25540.1 | 0.404 |
| 4350130 | 4352691 | LOC_Os11g12300.1 | LOC_Os12g40419.1 | 0.404 |
| 4350130 | 4352904 | LOC_Os11g12300.1 | LOC_Os12g44090.1 | 0.404 |
| 4350352 | 4343794 | LOC_Os11g20384.1 | LOC_Os07g40550.1 | 0.438 |
| 4350352 | 4345628 | LOC_Os11g20384.1 | LOC_Os08g33200.1 | 0.964 |
| 4350352 | 4347005 | LOC_Os11g20384.1 | LOC_Os09g23740.1 | 0.964 |
| 4350398 | 4329037 | LOC_Os11g24450.1 | LOC_Os02g18450.1 | 0.741 |
| 4350398 | 4345365 | LOC_Os11g24450.1 | LOC_Os08g27010.1 | 0.645 |
| 4350472 | 4326546 | LOC_Os11g27264.1 | LOC_Os01g53920.1 | 0.614 |
| 4350472 | 4330744 | LOC_Os11g27264.1 | LOC_Os02g51540.1 | 0.68 |
| 4350472 | 4339944 | LOC_Os11g27264.1 | LOC_Os06g02900.1 | 0.701 |
| 4350472 | 4345065 | LOC_Os11g27264.1 | LOC_Os08g14990.1 | 0.614 |
| 4350472 | 4345708 | LOC_Os11g27264.1 | LOC_Os08g34650.1 | 0.614 |
| 4350472 | 4347095 | LOC_Os11g27264.1 | LOC_Os09g25540.1 | 0.614 |
| 4350473 | 4326546 | LOC_Os11g27329.1 | LOC_Os01g53920.1 | 0.614 |
| 4350473 | 4330744 | LOC_Os11g27329.1 | LOC_Os02g51540.1 | 0.68 |
| 4350473 | 4339944 | LOC_Os11g27329.1 | LOC_Os06g02900.1 | 0.705 |
| 4350473 | 4345065 | LOC_Os11g27329.1 | LOC_Os08g14990.1 | 0.614 |
| 4350473 | 4345708 | LOC_Os11g27329.1 | LOC_Os08g34650.1 | 0.614 |
| 4350473 | 4347095 | LOC_Os11g27329.1 | LOC_Os09g25540.1 | 0.614 |
| 4350745 | 4344935 | LOC_Os11g36050.1 | LOC_Os08g10608.1 | 0.485 |
| 4350814 | 4326546 | LOC_Os11g37860.1 | LOC_Os01g53920.1 | 0.404 |
| 4350814 | 4327667 | LOC_Os11g37860.1 | LOC_Os01g40590.1 | 0.404 |
| 4350814 | 4328725 | LOC_Os11g37860.1 | LOC_Os02g12440.1 | 0.404 |
| 4350814 | 4330701 | LOC_Os11g37860.1 | LOC_Os02g50970.1 | 0.404 |
| 4350814 | 4331134 | LOC_Os11g37860.1 | LOC_Os02g57080.1 | 0.404 |
| 4350814 | 4333060 | LOC_Os11g37860.1 | LOC_Os03g28300.1 | 0.404 |
| 4350814 | 4334921 | LOC_Os11g37860.1 | LOC_Os04g01874.1 | 0.404 |
| 4350814 | 4335824 | LOC_Os11g37860.1 | LOC_Os04g34250.1 | 0.404 |
| 4350814 | 4337251 | LOC_Os11g37860.1 | LOC_Os04g56090.1 | 0.404 |
| 4350814 | 4338080 | LOC_Os11g37860.1 | LOC_Os05g11750.1 | 0.404 |
| 4350814 | 4338096 | LOC_Os11g37860.1 | LOC_Os05g11990.1 | 0.628 |
| 4350814 | 4339142 | LOC_Os11g37860.1 | LOC_Os05g40770.1 | 0.404 |
| 4350814 | 4341066 | LOC_Os11g37860.1 | LOC_Os06g29080.1 | 0.404 |
| 4350814 | 4341568 | LOC_Os11g37860.1 | LOC_Os06g41980.1 | 0.404 |
| 4350814 | 4342396 | LOC_Os11g37860.1 | LOC_Os07g05370.1 | 0.404 |
| 4350814 | 4342410 | LOC_Os11g37860.1 | LOC_Os07g05620.1 | 0.404 |
| 4350814 | 4343699 | LOC_Os11g37860.1 | LOC_Os07g38810.1 | 0.404 |
| 4350814 | 4343832 | LOC_Os11g37860.1 | LOC_Os07g41140.1 | 0.404 |
| 4350814 | 4345065 | LOC_Os11g37860.1 | LOC_Os08g14990.1 | 0.404 |
| 4350814 | 4345628 | LOC_Os11g37860.1 | LOC_Os08g33200.1 | 0.806 |
| 4350814 | 4345708 | LOC_Os11g37860.1 | LOC_Os08g34650.1 | 0.404 |
| 4350814 | 4347005 | LOC_Os11g37860.1 | LOC_Os09g23740.1 | 0.806 |
| 4350814 | 4347095 | LOC_Os11g37860.1 | LOC_Os09g25540.1 | 0.404 |
| 4350814 | 4352691 | LOC_Os11g37860.1 | LOC_Os12g40419.1 | 0.404 |
| 4350814 | 4352904 | LOC_Os11g37860.1 | LOC_Os12g44090.1 | 0.404 |
| 4350837 | 4324584 | LOC_Os11g38170.1 | LOC_Os01g11920.1 | 0.418 |
| 4350837 | 4331005 | LOC_Os11g38170.1 | LOC_Os02g55260.1 | 0.492 |
| 4350837 | 4331168 | LOC_Os11g38170.1 | LOC_Os02g57470.1 | 0.467 |
| 4350837 | 4332567 | LOC_Os11g38170.1 | LOC_Os03g18840.1 | 0.821 |
| 4350837 | 4333574 | LOC_Os11g38170.1 | LOC_Os03g44530.1 | 0.532 |
| 4350837 | 4333814 | LOC_Os11g38170.1 | LOC_Os03g49210.1 | 0.924 |
| 4350837 | 4336833 | LOC_Os11g38170.1 | LOC_Os04g50660.1 | 0.966 |
| 4350837 | 4338096 | LOC_Os11g38170.1 | LOC_Os05g11990.1 | 0.571 |
| 4350837 | 4342673 | LOC_Os11g38170.1 | LOC_Os07g10350.1 | 0.619 |
| 4350837 | 4344819 | LOC_Os11g38170.1 | LOC_Os08g08210.1 | 0.615 |
| 4350837 | 4347787 | LOC_Os11g38170.1 | LOC_Os09g37860.1 | 0.743 |
| 4350837 | LOC_Os02g49270.1 | LOC_Os11g38170.1 | LOC_Os02g49270.1 | 0.721 |
| 4350954 | 4326546 | LOC_Os11g40780.1 | LOC_Os01g53920.1 | 0.404 |
| 4350954 | 4327667 | LOC_Os11g40780.1 | LOC_Os01g40590.1 | 0.404 |
| 4350954 | 4328725 | LOC_Os11g40780.1 | LOC_Os02g12440.1 | 0.404 |
| 4350954 | 4330701 | LOC_Os11g40780.1 | LOC_Os02g50970.1 | 0.404 |
| 4350954 | 4331134 | LOC_Os11g40780.1 | LOC_Os02g57080.1 | 0.404 |
| 4350954 | 4333060 | LOC_Os11g40780.1 | LOC_Os03g28300.1 | 0.404 |
| 4350954 | 4334921 | LOC_Os11g40780.1 | LOC_Os04g01874.1 | 0.404 |
| 4350954 | 4335824 | LOC_Os11g40780.1 | LOC_Os04g34250.1 | 0.404 |
| 4350954 | 4337251 | LOC_Os11g40780.1 | LOC_Os04g56090.1 | 0.404 |
| 4350954 | 4338080 | LOC_Os11g40780.1 | LOC_Os05g11750.1 | 0.404 |
| 4350954 | 4338096 | LOC_Os11g40780.1 | LOC_Os05g11990.1 | 0.628 |
| 4350954 | 4339142 | LOC_Os11g40780.1 | LOC_Os05g40770.1 | 0.404 |
| 4350954 | 4341066 | LOC_Os11g40780.1 | LOC_Os06g29080.1 | 0.404 |
| 4350954 | 4341568 | LOC_Os11g40780.1 | LOC_Os06g41980.1 | 0.404 |
| 4350954 | 4342396 | LOC_Os11g40780.1 | LOC_Os07g05370.1 | 0.404 |
| 4350954 | 4342410 | LOC_Os11g40780.1 | LOC_Os07g05620.1 | 0.404 |
| 4350954 | 4343699 | LOC_Os11g40780.1 | LOC_Os07g38810.1 | 0.404 |
| 4350954 | 4343832 | LOC_Os11g40780.1 | LOC_Os07g41140.1 | 0.404 |
| 4350954 | 4345065 | LOC_Os11g40780.1 | LOC_Os08g14990.1 | 0.404 |
| 4350954 | 4345628 | LOC_Os11g40780.1 | LOC_Os08g33200.1 | 0.806 |
| 4350954 | 4345708 | LOC_Os11g40780.1 | LOC_Os08g34650.1 | 0.404 |
| 4350954 | 4347005 | LOC_Os11g40780.1 | LOC_Os09g23740.1 | 0.806 |
| 4350954 | 4347095 | LOC_Os11g40780.1 | LOC_Os09g25540.1 | 0.404 |
| 4350954 | 4352691 | LOC_Os11g40780.1 | LOC_Os12g40419.1 | 0.404 |
| 4350954 | 4352904 | LOC_Os11g40780.1 | LOC_Os12g44090.1 | 0.404 |
| 4350996 | 4326546 | LOC_Os11g42040.1 | LOC_Os01g53920.1 | 0.404 |
| 4350996 | 4327667 | LOC_Os11g42040.1 | LOC_Os01g40590.1 | 0.404 |
| 4350996 | 4328725 | LOC_Os11g42040.1 | LOC_Os02g12440.1 | 0.404 |
| 4350996 | 4330701 | LOC_Os11g42040.1 | LOC_Os02g50970.1 | 0.404 |
| 4350996 | 4331134 | LOC_Os11g42040.1 | LOC_Os02g57080.1 | 0.404 |
| 4350996 | 4332696 | LOC_Os11g42040.1 | LOC_Os03g20780.1 | 0.845 |
| 4350996 | 4333060 | LOC_Os11g42040.1 | LOC_Os03g28300.1 | 0.404 |
| 4350996 | 4334921 | LOC_Os11g42040.1 | LOC_Os04g01874.1 | 0.404 |
| 4350996 | 4335824 | LOC_Os11g42040.1 | LOC_Os04g34250.1 | 0.404 |
| 4350996 | 4337251 | LOC_Os11g42040.1 | LOC_Os04g56090.1 | 0.404 |
| 4350996 | 4338080 | LOC_Os11g42040.1 | LOC_Os05g11750.1 | 0.404 |
| 4350996 | 4338096 | LOC_Os11g42040.1 | LOC_Os05g11990.1 | 0.628 |
| 4350996 | 4339142 | LOC_Os11g42040.1 | LOC_Os05g40770.1 | 0.404 |
| 4350996 | 4341066 | LOC_Os11g42040.1 | LOC_Os06g29080.1 | 0.404 |
| 4350996 | 4341568 | LOC_Os11g42040.1 | LOC_Os06g41980.1 | 0.404 |
| 4350996 | 4342396 | LOC_Os11g42040.1 | LOC_Os07g05370.1 | 0.404 |
| 4350996 | 4342410 | LOC_Os11g42040.1 | LOC_Os07g05620.1 | 0.404 |
| 4350996 | 4343699 | LOC_Os11g42040.1 | LOC_Os07g38810.1 | 0.404 |
| 4350996 | 4343832 | LOC_Os11g42040.1 | LOC_Os07g41140.1 | 0.404 |
| 4350996 | 4345065 | LOC_Os11g42040.1 | LOC_Os08g14990.1 | 0.404 |
| 4350996 | 4345628 | LOC_Os11g42040.1 | LOC_Os08g33200.1 | 0.806 |
| 4350996 | 4345708 | LOC_Os11g42040.1 | LOC_Os08g34650.1 | 0.404 |
| 4350996 | 4347005 | LOC_Os11g42040.1 | LOC_Os09g23740.1 | 0.806 |
| 4350996 | 4347095 | LOC_Os11g42040.1 | LOC_Os09g25540.1 | 0.404 |
| 4350996 | 4350997 | LOC_Os11g42040.1 | LOC_Os11g42060.1 | 0.54 |
| 4350996 | 4352691 | LOC_Os11g42040.1 | LOC_Os12g40419.1 | 0.404 |
| 4350996 | 4352904 | LOC_Os11g42040.1 | LOC_Os12g44090.1 | 0.404 |
| 4350997 | 4350996 | LOC_Os11g42060.1 | LOC_Os11g42040.1 | 0.54 |
| 4351005 | 4326027 | LOC_Os11g42200.1 | LOC_Os01g25820.1 | 0.603 |
| 4351005 | 4341965 | LOC_Os11g42200.1 | LOC_Os06g48720.1 | 0.612 |
| 4351008 | 4324584 | LOC_Os11g42350.1 | LOC_Os01g11920.1 | 0.455 |
| 4351008 | 4338096 | LOC_Os11g42350.1 | LOC_Os05g11990.1 | 0.459 |
| 4351008 | 4343183 | LOC_Os11g42350.1 | LOC_Os07g28480.1 | 0.908 |
| 4351017 | 4328828 | LOC_Os11g42510.1 | LOC_Os02g14110.1 | 0.689 |
| 4351017 | 4341252 | LOC_Os11g42510.1 | LOC_Os06g35540.1 | 0.689 |
| 4351017 | 4345820 | LOC_Os11g42510.1 | LOC_Os08g37010.1 | 0.415 |
| 4351017 | 4348561 | LOC_Os11g42510.1 | LOC_Os10g26010.1 | 0.493 |
| 4351038 | 4326546 | LOC_Os11g43250.1 | LOC_Os01g53920.1 | 0.404 |
| 4351038 | 4327667 | LOC_Os11g43250.1 | LOC_Os01g40590.1 | 0.404 |
| 4351038 | 4328725 | LOC_Os11g43250.1 | LOC_Os02g12440.1 | 0.404 |
| 4351038 | 4330701 | LOC_Os11g43250.1 | LOC_Os02g50970.1 | 0.404 |
| 4351038 | 4331134 | LOC_Os11g43250.1 | LOC_Os02g57080.1 | 0.404 |
| 4351038 | 4333060 | LOC_Os11g43250.1 | LOC_Os03g28300.1 | 0.404 |
| 4351038 | 4334921 | LOC_Os11g43250.1 | LOC_Os04g01874.1 | 0.404 |
| 4351038 | 4335824 | LOC_Os11g43250.1 | LOC_Os04g34250.1 | 0.404 |
| 4351038 | 4337251 | LOC_Os11g43250.1 | LOC_Os04g56090.1 | 0.404 |
| 4351038 | 4338080 | LOC_Os11g43250.1 | LOC_Os05g11750.1 | 0.404 |
| 4351038 | 4338096 | LOC_Os11g43250.1 | LOC_Os05g11990.1 | 0.628 |
| 4351038 | 4339142 | LOC_Os11g43250.1 | LOC_Os05g40770.1 | 0.404 |
| 4351038 | 4341066 | LOC_Os11g43250.1 | LOC_Os06g29080.1 | 0.404 |
| 4351038 | 4341568 | LOC_Os11g43250.1 | LOC_Os06g41980.1 | 0.404 |
| 4351038 | 4342396 | LOC_Os11g43250.1 | LOC_Os07g05370.1 | 0.404 |
| 4351038 | 4342410 | LOC_Os11g43250.1 | LOC_Os07g05620.1 | 0.404 |
| 4351038 | 4343699 | LOC_Os11g43250.1 | LOC_Os07g38810.1 | 0.404 |
| 4351038 | 4343832 | LOC_Os11g43250.1 | LOC_Os07g41140.1 | 0.404 |
| 4351038 | 4345065 | LOC_Os11g43250.1 | LOC_Os08g14990.1 | 0.404 |
| 4351038 | 4345628 | LOC_Os11g43250.1 | LOC_Os08g33200.1 | 0.806 |
| 4351038 | 4345708 | LOC_Os11g43250.1 | LOC_Os08g34650.1 | 0.404 |
| 4351038 | 4347005 | LOC_Os11g43250.1 | LOC_Os09g23740.1 | 0.806 |
| 4351038 | 4347095 | LOC_Os11g43250.1 | LOC_Os09g25540.1 | 0.404 |
| 4351038 | 4352691 | LOC_Os11g43250.1 | LOC_Os12g40419.1 | 0.404 |
| 4351038 | 4352904 | LOC_Os11g43250.1 | LOC_Os12g44090.1 | 0.404 |
| 4351041 | 4326546 | LOC_Os11g43390.1 | LOC_Os01g53920.1 | 0.404 |
| 4351041 | 4327667 | LOC_Os11g43390.1 | LOC_Os01g40590.1 | 0.404 |
| 4351041 | 4328725 | LOC_Os11g43390.1 | LOC_Os02g12440.1 | 0.404 |
| 4351041 | 4330701 | LOC_Os11g43390.1 | LOC_Os02g50970.1 | 0.404 |
| 4351041 | 4331134 | LOC_Os11g43390.1 | LOC_Os02g57080.1 | 0.404 |
| 4351041 | 4333060 | LOC_Os11g43390.1 | LOC_Os03g28300.1 | 0.404 |
| 4351041 | 4334921 | LOC_Os11g43390.1 | LOC_Os04g01874.1 | 0.404 |
| 4351041 | 4335824 | LOC_Os11g43390.1 | LOC_Os04g34250.1 | 0.404 |
| 4351041 | 4337251 | LOC_Os11g43390.1 | LOC_Os04g56090.1 | 0.404 |
| 4351041 | 4338080 | LOC_Os11g43390.1 | LOC_Os05g11750.1 | 0.404 |
| 4351041 | 4338096 | LOC_Os11g43390.1 | LOC_Os05g11990.1 | 0.628 |
| 4351041 | 4339142 | LOC_Os11g43390.1 | LOC_Os05g40770.1 | 0.404 |
| 4351041 | 4341066 | LOC_Os11g43390.1 | LOC_Os06g29080.1 | 0.404 |
| 4351041 | 4341568 | LOC_Os11g43390.1 | LOC_Os06g41980.1 | 0.404 |
| 4351041 | 4342396 | LOC_Os11g43390.1 | LOC_Os07g05370.1 | 0.404 |
| 4351041 | 4342410 | LOC_Os11g43390.1 | LOC_Os07g05620.1 | 0.404 |
| 4351041 | 4343699 | LOC_Os11g43390.1 | LOC_Os07g38810.1 | 0.404 |
| 4351041 | 4343832 | LOC_Os11g43390.1 | LOC_Os07g41140.1 | 0.404 |
| 4351041 | 4345065 | LOC_Os11g43390.1 | LOC_Os08g14990.1 | 0.404 |
| 4351041 | 4345628 | LOC_Os11g43390.1 | LOC_Os08g33200.1 | 0.806 |
| 4351041 | 4345708 | LOC_Os11g43390.1 | LOC_Os08g34650.1 | 0.404 |
| 4351041 | 4347005 | LOC_Os11g43390.1 | LOC_Os09g23740.1 | 0.806 |
| 4351041 | 4347095 | LOC_Os11g43390.1 | LOC_Os09g25540.1 | 0.404 |
| 4351041 | 4352691 | LOC_Os11g43390.1 | LOC_Os12g40419.1 | 0.404 |
| 4351041 | 4352904 | LOC_Os11g43390.1 | LOC_Os12g44090.1 | 0.404 |
| 4351431 | 4324980 | LOC_Os12g03990.1 | LOC_Os01g67970.1 | 0.459 |
| 4351431 | 4326546 | LOC_Os12g03990.1 | LOC_Os01g53920.1 | 0.817 |
| 4351431 | 4328135 | LOC_Os12g03990.1 | LOC_Os02g03060.1 | 0.496 |
| 4351431 | 4330866 | LOC_Os12g03990.1 | LOC_Os02g53120.1 | 0.422 |
| 4351431 | 4342017 | LOC_Os12g03990.1 | LOC_Os06g49430.1 | 0.873 |
| 4351431 | 4344698 | LOC_Os12g03990.1 | LOC_Os08g06060.1 | 0.873 |
| 4351431 | 4345065 | LOC_Os12g03990.1 | LOC_Os08g14990.1 | 0.817 |
| 4351431 | 4345628 | LOC_Os12g03990.1 | LOC_Os08g33200.1 | 0.936 |
| 4351431 | 4345708 | LOC_Os12g03990.1 | LOC_Os08g34650.1 | 0.817 |
| 4351431 | 4347005 | LOC_Os12g03990.1 | LOC_Os09g23740.1 | 0.936 |
| 4351431 | 4347095 | LOC_Os12g03990.1 | LOC_Os09g25540.1 | 0.817 |
| 4351436 | 4327983 | LOC_Os12g04030.1 | LOC_Os02g01170.1 | 0.403 |
| 4351436 | 4328287 | LOC_Os12g04030.1 | LOC_Os02g05340.1 | 0.792 |
| 4351436 | 4332129 | LOC_Os12g04030.1 | LOC_Os03g12590.1 | 0.595 |
| 4351457 | 4324584 | LOC_Os12g04410.1 | LOC_Os01g11920.1 | 0.595 |
| 4351457 | 4326546 | LOC_Os12g04410.1 | LOC_Os01g53920.1 | 0.838 |
| 4351457 | 4327667 | LOC_Os12g04410.1 | LOC_Os01g40590.1 | 0.423 |
| 4351457 | 4328135 | LOC_Os12g04410.1 | LOC_Os02g03060.1 | 0.678 |
| 4351457 | 4331134 | LOC_Os12g04410.1 | LOC_Os02g57080.1 | 0.423 |
| 4351457 | 4338080 | LOC_Os12g04410.1 | LOC_Os05g11750.1 | 0.423 |
| 4351457 | 4338096 | LOC_Os12g04410.1 | LOC_Os05g11990.1 | 0.79 |
| 4351457 | 4341066 | LOC_Os12g04410.1 | LOC_Os06g29080.1 | 0.423 |
| 4351457 | 4342017 | LOC_Os12g04410.1 | LOC_Os06g49430.1 | 0.476 |
| 4351457 | 4342396 | LOC_Os12g04410.1 | LOC_Os07g05370.1 | 0.423 |
| 4351457 | 4342410 | LOC_Os12g04410.1 | LOC_Os07g05620.1 | 0.421 |
| 4351457 | 4344698 | LOC_Os12g04410.1 | LOC_Os08g06060.1 | 0.476 |
| 4351457 | 4345065 | LOC_Os12g04410.1 | LOC_Os08g14990.1 | 0.837 |
| 4351457 | 4345628 | LOC_Os12g04410.1 | LOC_Os08g33200.1 | 0.542 |
| 4351457 | 4345708 | LOC_Os12g04410.1 | LOC_Os08g34650.1 | 0.837 |
| 4351457 | 4347005 | LOC_Os12g04410.1 | LOC_Os09g23740.1 | 0.542 |
| 4351457 | 4347095 | LOC_Os12g04410.1 | LOC_Os09g25540.1 | 0.837 |
| 4351457 | 4352691 | LOC_Os12g04410.1 | LOC_Os12g40419.1 | 0.423 |
| 4351749 | 4326546 | LOC_Os12g10410.1 | LOC_Os01g53920.1 | 0.404 |
| 4351749 | 4327667 | LOC_Os12g10410.1 | LOC_Os01g40590.1 | 0.404 |
| 4351749 | 4328725 | LOC_Os12g10410.1 | LOC_Os02g12440.1 | 0.404 |
| 4351749 | 4330701 | LOC_Os12g10410.1 | LOC_Os02g50970.1 | 0.404 |
| 4351749 | 4331134 | LOC_Os12g10410.1 | LOC_Os02g57080.1 | 0.404 |
| 4351749 | 4333060 | LOC_Os12g10410.1 | LOC_Os03g28300.1 | 0.404 |
| 4351749 | 4334921 | LOC_Os12g10410.1 | LOC_Os04g01874.1 | 0.404 |
| 4351749 | 4335824 | LOC_Os12g10410.1 | LOC_Os04g34250.1 | 0.404 |
| 4351749 | 4337251 | LOC_Os12g10410.1 | LOC_Os04g56090.1 | 0.404 |
| 4351749 | 4338080 | LOC_Os12g10410.1 | LOC_Os05g11750.1 | 0.404 |
| 4351749 | 4338096 | LOC_Os12g10410.1 | LOC_Os05g11990.1 | 0.628 |
| 4351749 | 4339142 | LOC_Os12g10410.1 | LOC_Os05g40770.1 | 0.404 |
| 4351749 | 4341066 | LOC_Os12g10410.1 | LOC_Os06g29080.1 | 0.404 |
| 4351749 | 4341568 | LOC_Os12g10410.1 | LOC_Os06g41980.1 | 0.404 |
| 4351749 | 4342396 | LOC_Os12g10410.1 | LOC_Os07g05370.1 | 0.404 |
| 4351749 | 4342410 | LOC_Os12g10410.1 | LOC_Os07g05620.1 | 0.404 |
| 4351749 | 4343699 | LOC_Os12g10410.1 | LOC_Os07g38810.1 | 0.404 |
| 4351749 | 4343832 | LOC_Os12g10410.1 | LOC_Os07g41140.1 | 0.404 |
| 4351749 | 4345065 | LOC_Os12g10410.1 | LOC_Os08g14990.1 | 0.404 |
| 4351749 | 4345628 | LOC_Os12g10410.1 | LOC_Os08g33200.1 | 0.806 |
| 4351749 | 4345708 | LOC_Os12g10410.1 | LOC_Os08g34650.1 | 0.404 |
| 4351749 | 4347005 | LOC_Os12g10410.1 | LOC_Os09g23740.1 | 0.806 |
| 4351749 | 4347095 | LOC_Os12g10410.1 | LOC_Os09g25540.1 | 0.404 |
| 4351749 | 4352691 | LOC_Os12g10410.1 | LOC_Os12g40419.1 | 0.404 |
| 4351749 | 4352904 | LOC_Os12g10410.1 | LOC_Os12g44090.1 | 0.404 |
| 4351770 | 4330902 | LOC_Os12g10670.1 | LOC_Os02g53680.1 | 0.793 |
| 4351770 | 4331991 | LOC_Os12g10670.1 | LOC_Os03g10780.1 | 0.551 |
| 4351770 | 4337228 | LOC_Os12g10670.1 | LOC_Os04g55700.1 | 0.786 |
| 4351770 | 4337886 | LOC_Os12g10670.1 | LOC_Os05g06840.1 | 0.523 |
| 4351770 | 4339099 | LOC_Os12g10670.1 | LOC_Os05g39850.1 | 0.939 |
| 4351847 | 4328828 | LOC_Os12g13320.1 | LOC_Os02g14110.1 | 0.936 |
| 4351847 | 4338096 | LOC_Os12g13320.1 | LOC_Os05g11990.1 | 0.663 |
| 4351847 | 4341252 | LOC_Os12g13320.1 | LOC_Os06g35540.1 | 0.928 |
| 4352130 | 4326546 | LOC_Os12g25170.1 | LOC_Os01g53920.1 | 0.404 |
| 4352130 | 4327667 | LOC_Os12g25170.1 | LOC_Os01g40590.1 | 0.404 |
| 4352130 | 4328725 | LOC_Os12g25170.1 | LOC_Os02g12440.1 | 0.404 |
| 4352130 | 4330701 | LOC_Os12g25170.1 | LOC_Os02g50970.1 | 0.404 |
| 4352130 | 4331134 | LOC_Os12g25170.1 | LOC_Os02g57080.1 | 0.404 |
| 4352130 | 4333060 | LOC_Os12g25170.1 | LOC_Os03g28300.1 | 0.404 |
| 4352130 | 4334921 | LOC_Os12g25170.1 | LOC_Os04g01874.1 | 0.404 |
| 4352130 | 4335824 | LOC_Os12g25170.1 | LOC_Os04g34250.1 | 0.404 |
| 4352130 | 4337251 | LOC_Os12g25170.1 | LOC_Os04g56090.1 | 0.404 |
| 4352130 | 4338080 | LOC_Os12g25170.1 | LOC_Os05g11750.1 | 0.404 |
| 4352130 | 4338096 | LOC_Os12g25170.1 | LOC_Os05g11990.1 | 0.628 |
| 4352130 | 4339142 | LOC_Os12g25170.1 | LOC_Os05g40770.1 | 0.404 |
| 4352130 | 4341066 | LOC_Os12g25170.1 | LOC_Os06g29080.1 | 0.404 |
| 4352130 | 4341568 | LOC_Os12g25170.1 | LOC_Os06g41980.1 | 0.404 |
| 4352130 | 4342396 | LOC_Os12g25170.1 | LOC_Os07g05370.1 | 0.404 |
| 4352130 | 4342410 | LOC_Os12g25170.1 | LOC_Os07g05620.1 | 0.404 |
| 4352130 | 4343699 | LOC_Os12g25170.1 | LOC_Os07g38810.1 | 0.44 |
| 4352130 | 4343832 | LOC_Os12g25170.1 | LOC_Os07g41140.1 | 0.404 |
| 4352130 | 4345065 | LOC_Os12g25170.1 | LOC_Os08g14990.1 | 0.404 |
| 4352130 | 4345628 | LOC_Os12g25170.1 | LOC_Os08g33200.1 | 0.806 |
| 4352130 | 4345708 | LOC_Os12g25170.1 | LOC_Os08g34650.1 | 0.404 |
| 4352130 | 4347005 | LOC_Os12g25170.1 | LOC_Os09g23740.1 | 0.806 |
| 4352130 | 4347095 | LOC_Os12g25170.1 | LOC_Os09g25540.1 | 0.404 |
| 4352130 | 4352691 | LOC_Os12g25170.1 | LOC_Os12g40419.1 | 0.404 |
| 4352130 | 4352904 | LOC_Os12g25170.1 | LOC_Os12g44090.1 | 0.404 |
| 4352270 | 4326546 | LOC_Os12g31620.1 | LOC_Os01g53920.1 | 0.404 |
| 4352270 | 4327667 | LOC_Os12g31620.1 | LOC_Os01g40590.1 | 0.404 |
| 4352270 | 4328725 | LOC_Os12g31620.1 | LOC_Os02g12440.1 | 0.404 |
| 4352270 | 4330701 | LOC_Os12g31620.1 | LOC_Os02g50970.1 | 0.404 |
| 4352270 | 4331134 | LOC_Os12g31620.1 | LOC_Os02g57080.1 | 0.404 |
| 4352270 | 4333060 | LOC_Os12g31620.1 | LOC_Os03g28300.1 | 0.404 |
| 4352270 | 4334921 | LOC_Os12g31620.1 | LOC_Os04g01874.1 | 0.404 |
| 4352270 | 4335824 | LOC_Os12g31620.1 | LOC_Os04g34250.1 | 0.404 |
| 4352270 | 4337251 | LOC_Os12g31620.1 | LOC_Os04g56090.1 | 0.404 |
| 4352270 | 4338080 | LOC_Os12g31620.1 | LOC_Os05g11750.1 | 0.404 |
| 4352270 | 4338096 | LOC_Os12g31620.1 | LOC_Os05g11990.1 | 0.628 |
| 4352270 | 4339142 | LOC_Os12g31620.1 | LOC_Os05g40770.1 | 0.404 |
| 4352270 | 4341066 | LOC_Os12g31620.1 | LOC_Os06g29080.1 | 0.404 |
| 4352270 | 4341568 | LOC_Os12g31620.1 | LOC_Os06g41980.1 | 0.404 |
| 4352270 | 4342396 | LOC_Os12g31620.1 | LOC_Os07g05370.1 | 0.404 |
| 4352270 | 4342410 | LOC_Os12g31620.1 | LOC_Os07g05620.1 | 0.404 |
| 4352270 | 4343699 | LOC_Os12g31620.1 | LOC_Os07g38810.1 | 0.404 |
| 4352270 | 4343832 | LOC_Os12g31620.1 | LOC_Os07g41140.1 | 0.404 |
| 4352270 | 4345065 | LOC_Os12g31620.1 | LOC_Os08g14990.1 | 0.404 |
| 4352270 | 4345628 | LOC_Os12g31620.1 | LOC_Os08g33200.1 | 0.806 |
| 4352270 | 4345708 | LOC_Os12g31620.1 | LOC_Os08g34650.1 | 0.404 |
| 4352270 | 4347005 | LOC_Os12g31620.1 | LOC_Os09g23740.1 | 0.806 |
| 4352270 | 4347095 | LOC_Os12g31620.1 | LOC_Os09g25540.1 | 0.404 |
| 4352270 | 4352691 | LOC_Os12g31620.1 | LOC_Os12g40419.1 | 0.404 |
| 4352270 | 4352904 | LOC_Os12g31620.1 | LOC_Os12g44090.1 | 0.404 |
| 4352691 | 4324511 | LOC_Os12g40419.1 | LOC_Os01g05620.1 | 0.404 |
| 4352691 | 4324660 | LOC_Os12g40419.1 | LOC_Os01g52380.1 | 0.404 |
| 4352691 | 4333771 | LOC_Os12g40419.1 | LOC_Os03g48320.1 | 0.404 |
| 4352691 | 4334935 | LOC_Os12g40419.1 | LOC_Os04g02110.1 | 0.404 |
| 4352691 | 4335058 | LOC_Os12g40419.1 | LOC_Os04g08740.1 | 0.415 |
| 4352691 | 4336120 | LOC_Os12g40419.1 | LOC_Os04g39460.1 | 0.404 |
| 4352691 | 4338096 | LOC_Os12g40419.1 | LOC_Os05g11990.1 | 0.703 |
| 4352691 | 4338531 | LOC_Os12g40419.1 | LOC_Os05g29030.1 | 0.724 |
| 4352691 | 4339763 | LOC_Os12g40419.1 | LOC_Os05g50970.1 | 0.724 |
| 4352691 | 4340110 | LOC_Os12g40419.1 | LOC_Os06g05359.1 | 0.404 |
| 4352691 | 4340753 | LOC_Os12g40419.1 | LOC_Os06g16450.1 | 0.404 |
| 4352691 | 4341646 | LOC_Os12g40419.1 | LOC_Os06g43670.1 | 0.404 |
| 4352691 | 4342017 | LOC_Os12g40419.1 | LOC_Os06g49430.1 | 0.469 |
| 4352691 | 4343409 | LOC_Os12g40419.1 | LOC_Os07g33730.1 | 0.404 |
| 4352691 | 4344698 | LOC_Os12g40419.1 | LOC_Os08g06060.1 | 0.492 |
| 4352691 | 4345202 | LOC_Os12g40419.1 | LOC_Os08g20000.1 | 0.404 |
| 4352691 | 4345297 | LOC_Os12g40419.1 | LOC_Os08g24380.1 | 0.404 |
| 4352691 | 4345628 | LOC_Os12g40419.1 | LOC_Os08g33200.1 | 0.562 |
| 4352691 | 4347005 | LOC_Os12g40419.1 | LOC_Os09g23740.1 | 0.562 |
| 4352691 | 4347787 | LOC_Os12g40419.1 | LOC_Os09g37860.1 | 0.408 |
| 4352691 | 4349742 | LOC_Os12g40419.1 | LOC_Os11g04600.1 | 0.423 |
| 4352691 | 4350109 | LOC_Os12g40419.1 | LOC_Os11g11770.1 | 0.404 |
| 4352691 | 4350130 | LOC_Os12g40419.1 | LOC_Os11g12300.1 | 0.404 |
| 4352691 | 4350814 | LOC_Os12g40419.1 | LOC_Os11g37860.1 | 0.404 |
| 4352691 | 4350954 | LOC_Os12g40419.1 | LOC_Os11g40780.1 | 0.404 |
| 4352691 | 4350996 | LOC_Os12g40419.1 | LOC_Os11g42040.1 | 0.404 |
| 4352691 | 4351038 | LOC_Os12g40419.1 | LOC_Os11g43250.1 | 0.404 |
| 4352691 | 4351041 | LOC_Os12g40419.1 | LOC_Os11g43390.1 | 0.404 |
| 4352691 | 4351457 | LOC_Os12g40419.1 | LOC_Os12g04410.1 | 0.423 |
| 4352691 | 4351749 | LOC_Os12g40419.1 | LOC_Os12g10410.1 | 0.404 |
| 4352691 | 4352130 | LOC_Os12g40419.1 | LOC_Os12g25170.1 | 0.404 |
| 4352691 | 4352270 | LOC_Os12g40419.1 | LOC_Os12g31620.1 | 0.404 |
| 4352691 | IRL7 | LOC_Os12g40419.1 | LOC_Os03g11360.2 | 0.404 |
| 4352691 | LOC_Os02g49270.1 | LOC_Os12g40419.1 | LOC_Os02g49270.1 | 0.408 |
| 4352699 | 4326270 | LOC_Os12g40490.1 | LOC_Os01g16414.1 | 0.428 |
| 4352699 | 4337228 | LOC_Os12g40490.1 | LOC_Os04g55700.1 | 0.422 |
| 4352699 | 4348805 | LOC_Os12g40490.1 | LOC_Os10g31970.1 | 0.584 |
| 4352803 | 4332040 | LOC_Os12g42230.1 | LOC_Os03g11410.1 | 0.718 |
| 4352803 | 4334290 | LOC_Os12g42230.1 | LOC_Os03g56460.1 | 0.948 |
| 4352803 | 4335058 | LOC_Os12g42230.1 | LOC_Os04g08740.1 | 0.42 |
| 4352803 | 4335673 | LOC_Os12g42230.1 | LOC_Os04g32020.1 | 0.569 |
| 4352803 | 4337178 | LOC_Os12g42230.1 | LOC_Os04g55060.1 | 0.408 |
| 4352803 | 4347311 | LOC_Os12g42230.1 | LOC_Os09g29070.1 | 0.948 |
| 4352904 | 4324511 | LOC_Os12g44090.1 | LOC_Os01g05620.1 | 0.404 |
| 4352904 | 4324660 | LOC_Os12g44090.1 | LOC_Os01g52380.1 | 0.404 |
| 4352904 | 4333771 | LOC_Os12g44090.1 | LOC_Os03g48320.1 | 0.404 |
| 4352904 | 4334935 | LOC_Os12g44090.1 | LOC_Os04g02110.1 | 0.404 |
| 4352904 | 4335058 | LOC_Os12g44090.1 | LOC_Os04g08740.1 | 0.415 |
| 4352904 | 4336120 | LOC_Os12g44090.1 | LOC_Os04g39460.1 | 0.404 |
| 4352904 | 4338096 | LOC_Os12g44090.1 | LOC_Os05g11990.1 | 0.703 |
| 4352904 | 4338531 | LOC_Os12g44090.1 | LOC_Os05g29030.1 | 0.703 |
| 4352904 | 4339763 | LOC_Os12g44090.1 | LOC_Os05g50970.1 | 0.703 |
| 4352904 | 4340110 | LOC_Os12g44090.1 | LOC_Os06g05359.1 | 0.404 |
| 4352904 | 4340753 | LOC_Os12g44090.1 | LOC_Os06g16450.1 | 0.404 |
| 4352904 | 4341646 | LOC_Os12g44090.1 | LOC_Os06g43670.1 | 0.404 |
| 4352904 | 4342017 | LOC_Os12g44090.1 | LOC_Os06g49430.1 | 0.468 |
| 4352904 | 4343409 | LOC_Os12g44090.1 | LOC_Os07g33730.1 | 0.404 |
| 4352904 | 4344698 | LOC_Os12g44090.1 | LOC_Os08g06060.1 | 0.468 |
| 4352904 | 4345202 | LOC_Os12g44090.1 | LOC_Os08g20000.1 | 0.404 |
| 4352904 | 4345297 | LOC_Os12g44090.1 | LOC_Os08g24380.1 | 0.404 |
| 4352904 | 4345628 | LOC_Os12g44090.1 | LOC_Os08g33200.1 | 0.562 |
| 4352904 | 4347005 | LOC_Os12g44090.1 | LOC_Os09g23740.1 | 0.562 |
| 4352904 | 4347787 | LOC_Os12g44090.1 | LOC_Os09g37860.1 | 0.408 |
| 4352904 | 4350109 | LOC_Os12g44090.1 | LOC_Os11g11770.1 | 0.404 |
| 4352904 | 4350130 | LOC_Os12g44090.1 | LOC_Os11g12300.1 | 0.404 |
| 4352904 | 4350814 | LOC_Os12g44090.1 | LOC_Os11g37860.1 | 0.404 |
| 4352904 | 4350954 | LOC_Os12g44090.1 | LOC_Os11g40780.1 | 0.404 |
| 4352904 | 4350996 | LOC_Os12g44090.1 | LOC_Os11g42040.1 | 0.404 |
| 4352904 | 4351038 | LOC_Os12g44090.1 | LOC_Os11g43250.1 | 0.404 |
| 4352904 | 4351041 | LOC_Os12g44090.1 | LOC_Os11g43390.1 | 0.404 |
| 4352904 | 4351749 | LOC_Os12g44090.1 | LOC_Os12g10410.1 | 0.404 |
| 4352904 | 4352130 | LOC_Os12g44090.1 | LOC_Os12g25170.1 | 0.404 |
| 4352904 | 4352270 | LOC_Os12g44090.1 | LOC_Os12g31620.1 | 0.404 |
| 4352904 | IRL7 | LOC_Os12g44090.1 | LOC_Os03g11360.2 | 0.404 |
| 4352904 | LOC_Os02g49270.1 | LOC_Os12g44090.1 | LOC_Os02g49270.1 | 0.408 |
| ARF15 | 4327389 | LOC_Os05g48870.1 | LOC_Os01g62300.1 | 0.445 |
| ARF15 | 4331253 | LOC_Os05g48870.1 | LOC_Os02g58490.1 | 0.423 |
| ARF15 | C3H33 | LOC_Os05g48870.1 | LOC_Os05g03760.1 | 0.507 |
| BGLU2 | 4328745 | LOC_Os01g59819.1 | LOC_Os02g12730.1 | 0.468 |
| BGLU2 | 4334114 | LOC_Os01g59819.1 | LOC_Os03g53800.1 | 0.412 |
| BGLU2 | 4340373 | LOC_Os01g59819.1 | LOC_Os06g09240.1 | 0.485 |
| BGLU2 | 4343352 | LOC_Os01g59819.1 | LOC_Os07g32620.1 | 0.485 |
| C3H33 | ARF15 | LOC_Os05g03760.1 | LOC_Os05g48870.1 | 0.507 |
| CRSH3 | 4338096 | LOC_Os05g06940.1 | LOC_Os05g11990.1 | 0.539 |
| CRSH3 | 4340177 | LOC_Os05g06940.1 | LOC_Os06g06190.1 | 0.711 |
| H2B.9 | 4324481 | LOC_Os05g49860.1 | LOC_Os03g17100.1 | 0.986 |
| H2B.9 | 4324495 | LOC_Os05g49860.1 | LOC_Os01g05900.1 | 0.54 |
| H2B.9 | 4324500 | LOC_Os05g49860.1 | LOC_Os01g05630.1 | 0.894 |
| H2B.9 | 4324980 | LOC_Os05g49860.1 | LOC_Os01g67970.1 | 0.426 |
| H2B.9 | 4326270 | LOC_Os05g49860.1 | LOC_Os01g16414.1 | 0.473 |
| H2B.9 | 4327384 | LOC_Os05g49860.1 | LOC_Os01g62230.1 | 0.54 |
| H2B.9 | 4341655 | LOC_Os05g49860.1 | LOC_Os06g43790.1 | 0.683 |
| H2B.9 | 4342472 | LOC_Os05g49860.1 | LOC_Os07g06980.1 | 0.774 |
| H2B.9 | 4343299 | LOC_Os05g49860.1 | LOC_Os07g31450.1 | 0.463 |
| H2B.9 | 4344819 | LOC_Os05g49860.1 | LOC_Os08g08210.1 | 0.492 |
| H2B.9 | 4346109 | LOC_Os05g49860.1 | LOC_Os08g41630.1 | 0.53 |
| IRL7 | 4326270 | LOC_Os03g11360.2 | LOC_Os01g16414.1 | 0.45 |
| IRL7 | 4327667 | LOC_Os03g11360.2 | LOC_Os01g40590.1 | 0.404 |
| IRL7 | 4330701 | LOC_Os03g11360.2 | LOC_Os02g50970.1 | 0.404 |
| IRL7 | 4331134 | LOC_Os03g11360.2 | LOC_Os02g57080.1 | 0.404 |
| IRL7 | 4333060 | LOC_Os03g11360.2 | LOC_Os03g28300.1 | 0.404 |
| IRL7 | 4334214 | LOC_Os03g11360.2 | LOC_Os03g55560.1 | 0.604 |
| IRL7 | 4334921 | LOC_Os03g11360.2 | LOC_Os04g01874.1 | 0.404 |
| IRL7 | 4335824 | LOC_Os03g11360.2 | LOC_Os04g34250.1 | 0.404 |
| IRL7 | 4337251 | LOC_Os03g11360.2 | LOC_Os04g56090.1 | 0.404 |
| IRL7 | 4338080 | LOC_Os03g11360.2 | LOC_Os05g11750.1 | 0.404 |
| IRL7 | 4338096 | LOC_Os03g11360.2 | LOC_Os05g11990.1 | 0.628 |
| IRL7 | 4341066 | LOC_Os03g11360.2 | LOC_Os06g29080.1 | 0.404 |
| IRL7 | 4341568 | LOC_Os03g11360.2 | LOC_Os06g41980.1 | 0.404 |
| IRL7 | 4342017 | LOC_Os03g11360.2 | LOC_Os06g49430.1 | 0.523 |
| IRL7 | 4342396 | LOC_Os03g11360.2 | LOC_Os07g05370.1 | 0.404 |
| IRL7 | 4342410 | LOC_Os03g11360.2 | LOC_Os07g05620.1 | 0.404 |
| IRL7 | 4343699 | LOC_Os03g11360.2 | LOC_Os07g38810.1 | 0.404 |
| IRL7 | 4344698 | LOC_Os03g11360.2 | LOC_Os08g06060.1 | 0.523 |
| IRL7 | 4345628 | LOC_Os03g11360.2 | LOC_Os08g33200.1 | 0.814 |
| IRL7 | 4347005 | LOC_Os03g11360.2 | LOC_Os09g23740.1 | 0.814 |
| IRL7 | 4352691 | LOC_Os03g11360.2 | LOC_Os12g40419.1 | 0.404 |
| IRL7 | 4352904 | LOC_Os03g11360.2 | LOC_Os12g44090.1 | 0.404 |
| LOC_Os02g49270.1 | 4326546 | LOC_Os02g49270.1 | LOC_Os01g53920.1 | 0.537 |
| LOC_Os02g49270.1 | 4327667 | LOC_Os02g49270.1 | LOC_Os01g40590.1 | 0.408 |
| LOC_Os02g49270.1 | 4328725 | LOC_Os02g49270.1 | LOC_Os02g12440.1 | 0.408 |
| LOC_Os02g49270.1 | 4330701 | LOC_Os02g49270.1 | LOC_Os02g50970.1 | 0.408 |
| LOC_Os02g49270.1 | 4331005 | LOC_Os02g49270.1 | LOC_Os02g55260.1 | 0.679 |
| LOC_Os02g49270.1 | 4331134 | LOC_Os02g49270.1 | LOC_Os02g57080.1 | 0.408 |
| LOC_Os02g49270.1 | 4332567 | LOC_Os02g49270.1 | LOC_Os03g18840.1 | 0.784 |
| LOC_Os02g49270.1 | 4333060 | LOC_Os02g49270.1 | LOC_Os03g28300.1 | 0.408 |
| LOC_Os02g49270.1 | 4333574 | LOC_Os02g49270.1 | LOC_Os03g44530.1 | 0.819 |
| LOC_Os02g49270.1 | 4333814 | LOC_Os02g49270.1 | LOC_Os03g49210.1 | 0.997 |
| LOC_Os02g49270.1 | 4334921 | LOC_Os02g49270.1 | LOC_Os04g01874.1 | 0.408 |
| LOC_Os02g49270.1 | 4335824 | LOC_Os02g49270.1 | LOC_Os04g34250.1 | 0.408 |
| LOC_Os02g49270.1 | 4336833 | LOC_Os02g49270.1 | LOC_Os04g50660.1 | 0.968 |
| LOC_Os02g49270.1 | 4337251 | LOC_Os02g49270.1 | LOC_Os04g56090.1 | 0.408 |
| LOC_Os02g49270.1 | 4338080 | LOC_Os02g49270.1 | LOC_Os05g11750.1 | 0.46 |
| LOC_Os02g49270.1 | 4338096 | LOC_Os02g49270.1 | LOC_Os05g11990.1 | 0.4 |
| LOC_Os02g49270.1 | 4338531 | LOC_Os02g49270.1 | LOC_Os05g29030.1 | 0.404 |
| LOC_Os02g49270.1 | 4339142 | LOC_Os02g49270.1 | LOC_Os05g40770.1 | 0.408 |
| LOC_Os02g49270.1 | 4339763 | LOC_Os02g49270.1 | LOC_Os05g50970.1 | 0.404 |
| LOC_Os02g49270.1 | 4341066 | LOC_Os02g49270.1 | LOC_Os06g29080.1 | 0.408 |
| LOC_Os02g49270.1 | 4341219 | LOC_Os02g49270.1 | LOC_Os06g34690.1 | 0.635 |
| LOC_Os02g49270.1 | 4341568 | LOC_Os02g49270.1 | LOC_Os06g41980.1 | 0.408 |
| LOC_Os02g49270.1 | 4342396 | LOC_Os02g49270.1 | LOC_Os07g05370.1 | 0.408 |
| LOC_Os02g49270.1 | 4342410 | LOC_Os02g49270.1 | LOC_Os07g05620.1 | 0.408 |
| LOC_Os02g49270.1 | 4342673 | LOC_Os02g49270.1 | LOC_Os07g10350.1 | 0.988 |
| LOC_Os02g49270.1 | 4343699 | LOC_Os02g49270.1 | LOC_Os07g38810.1 | 0.408 |
| LOC_Os02g49270.1 | 4343832 | LOC_Os02g49270.1 | LOC_Os07g41140.1 | 0.408 |
| LOC_Os02g49270.1 | 4345065 | LOC_Os02g49270.1 | LOC_Os08g14990.1 | 0.537 |
| LOC_Os02g49270.1 | 4345708 | LOC_Os02g49270.1 | LOC_Os08g34650.1 | 0.537 |
| LOC_Os02g49270.1 | 4347095 | LOC_Os02g49270.1 | LOC_Os09g25540.1 | 0.537 |
| LOC_Os02g49270.1 | 4347787 | LOC_Os02g49270.1 | LOC_Os09g37860.1 | 0.837 |
| LOC_Os02g49270.1 | 4350837 | LOC_Os02g49270.1 | LOC_Os11g38170.1 | 0.721 |
| LOC_Os02g49270.1 | 4352691 | LOC_Os02g49270.1 | LOC_Os12g40419.1 | 0.408 |
| LOC_Os02g49270.1 | 4352904 | LOC_Os02g49270.1 | LOC_Os12g44090.1 | 0.408 |
| LOC_Os03g20790.1 | 4335058 | LOC_Os03g20790.1 | LOC_Os04g08740.1 | 0.461 |
| LOC_Os03g64080.1 | 4324980 | LOC_Os03g64080.1 | LOC_Os01g67970.1 | 0.788 |
| LOC_Os03g64080.1 | 4332129 | LOC_Os03g64080.1 | LOC_Os03g12590.1 | 0.41 |
| LOC_Os03g64080.1 | 4337658 | LOC_Os03g64080.1 | LOC_Os05g03100.1 | 0.424 |
| LOC_Os03g64080.1 | 4339099 | LOC_Os03g64080.1 | LOC_Os05g39850.1 | 0.479 |
| LOC_Os03g64080.1 | 4342017 | LOC_Os03g64080.1 | LOC_Os06g49430.1 | 0.656 |
| LOC_Os03g64080.1 | 4344698 | LOC_Os03g64080.1 | LOC_Os08g06060.1 | 0.656 |
| LOC_Os03g64080.1 | 4346276 | LOC_Os03g64080.1 | LOC_Os08g44050.1 | 0.76 |
| LOC_Os06g04340.1 | 4332049 | LOC_Os06g04340.1 | LOC_Os03g11510.1 | 0.425 |
| LOC_Os06g04340.1 | 4332567 | LOC_Os06g04340.1 | LOC_Os03g18840.1 | 0.405 |
| LOC_Os08g43250.1 | 4338481 | LOC_Os08g43250.1 | LOC_Os05g27880.1 | 0.609 |
| LOC_Os10g11340.1 | 4338096 | LOC_Os10g11340.1 | LOC_Os05g11990.1 | 0.427 |
| STLP1 | 4324709 | LOC_Os01g63970.1 | LOC_Os01g56570.1 | 0.436 |
| STLP1 | 4332380 | LOC_Os01g63970.1 | LOC_Os03g16290.1 | 0.408 |
| STLP1 | 4342645 | LOC_Os01g63970.1 | LOC_Os07g09690.1 | 0.434 |
| YUCCA1 | 4343395 | LOC_Os01g45760.1 | LOC_Os07g33480.1 | 0.536 |
| YUCCA1 | 4347172 | LOC_Os01g45760.1 | LOC_Os09g26880.1 | 0.863 |

Table S2. Interaction Network Table of 50 Core Nodes

| node1 | node2 | node1 accession | node2 accession | score |
| --- | --- | --- | --- | --- |
| 4324364 | 4326546 | LOC_Os01g69030.2 | LOC_Os01g53920.1 | 0.634 |
| 4324364 | 4338096 | LOC_Os01g69030.2 | LOC_Os05g11990.1 | 0.671 |
| 4324364 | 4345065 | LOC_Os01g69030.2 | LOC_Os08g14990.1 | 0.634 |
| 4324364 | 4345708 | LOC_Os01g69030.2 | LOC_Os08g34650.1 | 0.634 |
| 4324364 | 4347095 | LOC_Os01g69030.2 | LOC_Os09g25540.1 | 0.634 |
| 4324481 | 4324495 | LOC_Os03g17100.1 | LOC_Os01g05900.1 | 0.986 |
| 4324481 | 4324500 | LOC_Os03g17100.1 | LOC_Os01g05630.1 | 0.986 |
| 4324481 | 4324980 | LOC_Os03g17100.1 | LOC_Os01g67970.1 | 0.645 |
| 4324481 | 4342472 | LOC_Os03g17100.1 | LOC_Os07g06980.1 | 0.657 |
| 4324495 | 4324481 | LOC_Os01g05900.1 | LOC_Os03g17100.1 | 0.986 |
| 4324495 | 4324500 | LOC_Os01g05900.1 | LOC_Os01g05630.1 | 0.645 |
| 4324495 | 4326270 | LOC_Os01g05900.1 | LOC_Os01g16414.1 | 0.473 |
| 4324495 | 4342472 | LOC_Os01g05900.1 | LOC_Os07g06980.1 | 0.774 |
| 4324500 | 4324481 | LOC_Os01g05630.1 | LOC_Os03g17100.1 | 0.986 |
| 4324500 | 4324495 | LOC_Os01g05630.1 | LOC_Os01g05900.1 | 0.645 |
| 4324500 | 4326270 | LOC_Os01g05630.1 | LOC_Os01g16414.1 | 0.473 |
| 4324500 | 4342472 | LOC_Os01g05630.1 | LOC_Os07g06980.1 | 0.774 |
| 4324584 | 4324980 | LOC_Os01g11920.1 | LOC_Os01g67970.1 | 0.652 |
| 4324584 | 4326270 | LOC_Os01g11920.1 | LOC_Os01g16414.1 | 0.646 |
| 4324584 | 4326546 | LOC_Os01g11920.1 | LOC_Os01g53920.1 | 0.691 |
| 4324584 | 4328135 | LOC_Os01g11920.1 | LOC_Os02g03060.1 | 0.553 |
| 4324584 | 4332080 | LOC_Os01g11920.1 | LOC_Os03g11910.1 | 0.807 |
| 4324584 | 4338531 | LOC_Os01g11920.1 | LOC_Os05g29030.1 | 0.516 |
| 4324584 | 4339763 | LOC_Os01g11920.1 | LOC_Os05g50970.1 | 0.516 |
| 4324584 | 4341853 | LOC_Os01g11920.1 | LOC_Os06g46600.1 | 0.656 |
| 4324584 | 4342017 | LOC_Os01g11920.1 | LOC_Os06g49430.1 | 0.516 |
| 4324584 | 4342472 | LOC_Os01g11920.1 | LOC_Os07g06980.1 | 0.475 |
| 4324584 | 4343196 | LOC_Os01g11920.1 | LOC_Os07g28800.1 | 0.669 |
| 4324584 | 4344698 | LOC_Os01g11920.1 | LOC_Os08g06060.1 | 0.516 |
| 4324584 | 4345065 | LOC_Os01g11920.1 | LOC_Os08g14990.1 | 0.691 |
| 4324584 | 4345708 | LOC_Os01g11920.1 | LOC_Os08g34650.1 | 0.691 |
| 4324584 | 4346276 | LOC_Os01g11920.1 | LOC_Os08g44050.1 | 0.478 |
| 4324584 | 4347095 | LOC_Os01g11920.1 | LOC_Os09g25540.1 | 0.691 |
| 4324584 | 4349742 | LOC_Os01g11920.1 | LOC_Os11g04600.1 | 0.595 |
| 4324584 | 4350837 | LOC_Os01g11920.1 | LOC_Os11g38170.1 | 0.418 |
| 4324584 | 4351457 | LOC_Os01g11920.1 | LOC_Os12g04410.1 | 0.595 |
| 4324980 | 4324481 | LOC_Os01g67970.1 | LOC_Os03g17100.1 | 0.645 |
| 4324980 | 4324584 | LOC_Os01g67970.1 | LOC_Os01g11920.1 | 0.652 |
| 4324980 | 4326270 | LOC_Os01g67970.1 | LOC_Os01g16414.1 | 0.611 |
| 4324980 | 4326546 | LOC_Os01g67970.1 | LOC_Os01g53920.1 | 0.703 |
| 4324980 | 4328135 | LOC_Os01g67970.1 | LOC_Os02g03060.1 | 0.659 |
| 4324980 | 4338096 | LOC_Os01g67970.1 | LOC_Os05g11990.1 | 0.666 |
| 4324980 | 4342017 | LOC_Os01g67970.1 | LOC_Os06g49430.1 | 0.718 |
| 4324980 | 4342472 | LOC_Os01g67970.1 | LOC_Os07g06980.1 | 0.81 |
| 4324980 | 4344698 | LOC_Os01g67970.1 | LOC_Os08g06060.1 | 0.718 |
| 4324980 | 4345065 | LOC_Os01g67970.1 | LOC_Os08g14990.1 | 0.703 |
| 4324980 | 4345708 | LOC_Os01g67970.1 | LOC_Os08g34650.1 | 0.703 |
| 4324980 | 4347095 | LOC_Os01g67970.1 | LOC_Os09g25540.1 | 0.703 |
| 4324980 | 4351431 | LOC_Os01g67970.1 | LOC_Os12g03990.1 | 0.459 |
| 4326270 | 4324495 | LOC_Os01g16414.1 | LOC_Os01g05900.1 | 0.473 |
| 4326270 | 4324500 | LOC_Os01g16414.1 | LOC_Os01g05630.1 | 0.473 |
| 4326270 | 4324584 | LOC_Os01g16414.1 | LOC_Os01g11920.1 | 0.646 |
| 4326270 | 4324980 | LOC_Os01g16414.1 | LOC_Os01g67970.1 | 0.611 |
| 4326270 | 4326546 | LOC_Os01g16414.1 | LOC_Os01g53920.1 | 0.606 |
| 4326270 | 4328135 | LOC_Os01g16414.1 | LOC_Os02g03060.1 | 0.466 |
| 4326270 | 4335089 | LOC_Os01g16414.1 | LOC_Os04g09860.1 | 0.697 |
| 4326270 | 4338096 | LOC_Os01g16414.1 | LOC_Os05g11990.1 | 0.582 |
| 4326270 | 4342017 | LOC_Os01g16414.1 | LOC_Os06g49430.1 | 0.519 |
| 4326270 | 4342472 | LOC_Os01g16414.1 | LOC_Os07g06980.1 | 0.433 |
| 4326270 | 4344698 | LOC_Os01g16414.1 | LOC_Os08g06060.1 | 0.519 |
| 4326270 | 4345065 | LOC_Os01g16414.1 | LOC_Os08g14990.1 | 0.606 |
| 4326270 | 4345628 | LOC_Os01g16414.1 | LOC_Os08g33200.1 | 0.514 |
| 4326270 | 4345708 | LOC_Os01g16414.1 | LOC_Os08g34650.1 | 0.606 |
| 4326270 | 4346276 | LOC_Os01g16414.1 | LOC_Os08g44050.1 | 0.549 |
| 4326270 | 4347005 | LOC_Os01g16414.1 | LOC_Os09g23740.1 | 0.514 |
| 4326270 | 4347095 | LOC_Os01g16414.1 | LOC_Os09g25540.1 | 0.606 |
| 4326546 | 4324364 | LOC_Os01g53920.1 | LOC_Os01g69030.2 | 0.634 |
| 4326546 | 4324584 | LOC_Os01g53920.1 | LOC_Os01g11920.1 | 0.691 |
| 4326546 | 4324980 | LOC_Os01g53920.1 | LOC_Os01g67970.1 | 0.703 |
| 4326546 | 4326270 | LOC_Os01g53920.1 | LOC_Os01g16414.1 | 0.606 |
| 4326546 | 4327983 | LOC_Os01g53920.1 | LOC_Os02g01170.1 | 0.655 |
| 4326546 | 4328135 | LOC_Os01g53920.1 | LOC_Os02g03060.1 | 0.677 |
| 4326546 | 4332080 | LOC_Os01g53920.1 | LOC_Os03g11910.1 | 0.776 |
| 4326546 | 4334214 | LOC_Os01g53920.1 | LOC_Os03g55560.1 | 0.927 |
| 4326546 | 4335058 | LOC_Os01g53920.1 | LOC_Os04g08740.1 | 0.659 |
| 4326546 | 4335089 | LOC_Os01g53920.1 | LOC_Os04g09860.1 | 0.606 |
| 4326546 | 4337619 | LOC_Os01g53920.1 | LOC_Os05g02500.1 | 0.817 |
| 4326546 | 4337686 | LOC_Os01g53920.1 | LOC_Os05g03610.1 | 0.726 |
| 4326546 | 4338096 | LOC_Os01g53920.1 | LOC_Os05g11990.1 | 0.968 |
| 4326546 | 4338531 | LOC_Os01g53920.1 | LOC_Os05g29030.1 | 0.963 |
| 4326546 | 4339763 | LOC_Os01g53920.1 | LOC_Os05g50970.1 | 0.963 |
| 4326546 | 4340847 | LOC_Os01g53920.1 | LOC_Os06g20340.1 | 0.817 |
| 4326546 | 4341219 | LOC_Os01g53920.1 | LOC_Os06g34690.1 | 0.641 |
| 4326546 | 4341853 | LOC_Os01g53920.1 | LOC_Os06g46600.1 | 0.776 |
| 4326546 | 4342017 | LOC_Os01g53920.1 | LOC_Os06g49430.1 | 0.765 |
| 4326546 | 4344698 | LOC_Os01g53920.1 | LOC_Os08g06060.1 | 0.846 |
| 4326546 | 4345065 | LOC_Os01g53920.1 | LOC_Os08g14990.1 | 0.956 |
| 4326546 | 4345628 | LOC_Os01g53920.1 | LOC_Os08g33200.1 | 0.985 |
| 4326546 | 4345708 | LOC_Os01g53920.1 | LOC_Os08g34650.1 | 0.953 |
| 4326546 | 4345910 | LOC_Os01g53920.1 | LOC_Os08g38410.1 | 0.628 |
| 4326546 | 4346276 | LOC_Os01g53920.1 | LOC_Os08g44050.1 | 0.703 |
| 4326546 | 4347005 | LOC_Os01g53920.1 | LOC_Os09g23740.1 | 0.985 |
| 4326546 | 4347095 | LOC_Os01g53920.1 | LOC_Os09g25540.1 | 0.956 |
| 4326546 | 4347787 | LOC_Os01g53920.1 | LOC_Os09g37860.1 | 0.537 |
| 4326546 | 4349742 | LOC_Os01g53920.1 | LOC_Os11g04600.1 | 0.838 |
| 4326546 | 4350472 | LOC_Os01g53920.1 | LOC_Os11g27264.1 | 0.614 |
| 4326546 | 4350473 | LOC_Os01g53920.1 | LOC_Os11g27329.1 | 0.614 |
| 4326546 | 4351431 | LOC_Os01g53920.1 | LOC_Os12g03990.1 | 0.817 |
| 4326546 | 4351457 | LOC_Os01g53920.1 | LOC_Os12g04410.1 | 0.838 |
| 4326546 | LOC_Os02g49270.1 | LOC_Os01g53920.1 | LOC_Os02g49270.1 | 0.537 |
| 4327983 | 4326546 | LOC_Os02g01170.1 | LOC_Os01g53920.1 | 0.655 |
| 4327983 | 4345065 | LOC_Os02g01170.1 | LOC_Os08g14990.1 | 0.655 |
| 4327983 | 4345708 | LOC_Os02g01170.1 | LOC_Os08g34650.1 | 0.655 |
| 4327983 | 4347095 | LOC_Os02g01170.1 | LOC_Os09g25540.1 | 0.655 |
| 4328135 | 4324584 | LOC_Os02g03060.1 | LOC_Os01g11920.1 | 0.553 |
| 4328135 | 4324980 | LOC_Os02g03060.1 | LOC_Os01g67970.1 | 0.659 |
| 4328135 | 4326270 | LOC_Os02g03060.1 | LOC_Os01g16414.1 | 0.466 |
| 4328135 | 4326546 | LOC_Os02g03060.1 | LOC_Os01g53920.1 | 0.677 |
| 4328135 | 4328287 | LOC_Os02g03060.1 | LOC_Os02g05340.1 | 0.991 |
| 4328135 | 4330866 | LOC_Os02g03060.1 | LOC_Os02g53120.1 | 0.528 |
| 4328135 | 4337619 | LOC_Os02g03060.1 | LOC_Os05g02500.1 | 0.496 |
| 4328135 | 4338096 | LOC_Os02g03060.1 | LOC_Os05g11990.1 | 0.598 |
| 4328135 | 4340847 | LOC_Os02g03060.1 | LOC_Os06g20340.1 | 0.496 |
| 4328135 | 4342017 | LOC_Os02g03060.1 | LOC_Os06g49430.1 | 0.594 |
| 4328135 | 4344698 | LOC_Os02g03060.1 | LOC_Os08g06060.1 | 0.619 |
| 4328135 | 4345065 | LOC_Os02g03060.1 | LOC_Os08g14990.1 | 0.675 |
| 4328135 | 4345708 | LOC_Os02g03060.1 | LOC_Os08g34650.1 | 0.671 |
| 4328135 | 4346276 | LOC_Os02g03060.1 | LOC_Os08g44050.1 | 0.612 |
| 4328135 | 4347095 | LOC_Os02g03060.1 | LOC_Os09g25540.1 | 0.673 |
| 4328135 | 4349742 | LOC_Os02g03060.1 | LOC_Os11g04600.1 | 0.678 |
| 4328135 | 4351431 | LOC_Os02g03060.1 | LOC_Os12g03990.1 | 0.496 |
| 4328135 | 4351457 | LOC_Os02g03060.1 | LOC_Os12g04410.1 | 0.678 |
| 4328287 | 4328135 | LOC_Os02g05340.1 | LOC_Os02g03060.1 | 0.991 |
| 4330796 | 4333814 | LOC_Os02g52250.1 | LOC_Os03g49210.1 | 0.635 |
| 4330796 | 4342472 | LOC_Os02g52250.1 | LOC_Os07g06980.1 | 0.604 |
| 4330796 | 4345910 | LOC_Os02g52250.1 | LOC_Os08g38410.1 | 0.664 |
| 4330866 | 4328135 | LOC_Os02g53120.1 | LOC_Os02g03060.1 | 0.528 |
| 4330866 | 4334214 | LOC_Os02g53120.1 | LOC_Os03g55560.1 | 0.43 |
| 4330866 | 4337619 | LOC_Os02g53120.1 | LOC_Os05g02500.1 | 0.422 |
| 4330866 | 4340847 | LOC_Os02g53120.1 | LOC_Os06g20340.1 | 0.422 |
| 4330866 | 4351431 | LOC_Os02g53120.1 | LOC_Os12g03990.1 | 0.422 |
| 4331005 | 4332567 | LOC_Os02g55260.1 | LOC_Os03g18840.1 | 0.901 |
| 4331005 | 4333574 | LOC_Os02g55260.1 | LOC_Os03g44530.1 | 0.499 |
| 4331005 | 4333814 | LOC_Os02g55260.1 | LOC_Os03g49210.1 | 0.835 |
| 4331005 | 4336833 | LOC_Os02g55260.1 | LOC_Os04g50660.1 | 0.903 |
| 4331005 | 4342673 | LOC_Os02g55260.1 | LOC_Os07g10350.1 | 0.769 |
| 4331005 | 4347787 | LOC_Os02g55260.1 | LOC_Os09g37860.1 | 0.716 |
| 4331005 | 4350837 | LOC_Os02g55260.1 | LOC_Os11g38170.1 | 0.492 |
| 4331005 | LOC_Os02g49270.1 | LOC_Os02g55260.1 | LOC_Os02g49270.1 | 0.679 |
| 4332080 | 4324584 | LOC_Os03g11910.1 | LOC_Os01g11920.1 | 0.807 |
| 4332080 | 4326546 | LOC_Os03g11910.1 | LOC_Os01g53920.1 | 0.776 |
| 4332080 | 4338096 | LOC_Os03g11910.1 | LOC_Os05g11990.1 | 0.928 |
| 4332080 | 4341219 | LOC_Os03g11910.1 | LOC_Os06g34690.1 | 0.843 |
| 4332080 | 4341853 | LOC_Os03g11910.1 | LOC_Os06g46600.1 | 0.653 |
| 4332080 | 4342673 | LOC_Os03g11910.1 | LOC_Os07g10350.1 | 0.437 |
| 4332080 | 4343196 | LOC_Os03g11910.1 | LOC_Os07g28800.1 | 0.91 |
| 4332080 | 4345065 | LOC_Os03g11910.1 | LOC_Os08g14990.1 | 0.776 |
| 4332080 | 4345628 | LOC_Os03g11910.1 | LOC_Os08g33200.1 | 0.88 |
| 4332080 | 4345708 | LOC_Os03g11910.1 | LOC_Os08g34650.1 | 0.776 |
| 4332080 | 4347005 | LOC_Os03g11910.1 | LOC_Os09g23740.1 | 0.88 |
| 4332080 | 4347095 | LOC_Os03g11910.1 | LOC_Os09g25540.1 | 0.776 |
| 4332567 | 4331005 | LOC_Os03g18840.1 | LOC_Os02g55260.1 | 0.901 |
| 4332567 | 4333574 | LOC_Os03g18840.1 | LOC_Os03g44530.1 | 0.912 |
| 4332567 | 4333814 | LOC_Os03g18840.1 | LOC_Os03g49210.1 | 0.973 |
| 4332567 | 4336833 | LOC_Os03g18840.1 | LOC_Os04g50660.1 | 0.997 |
| 4332567 | 4342673 | LOC_Os03g18840.1 | LOC_Os07g10350.1 | 0.983 |
| 4332567 | 4347787 | LOC_Os03g18840.1 | LOC_Os09g37860.1 | 0.909 |
| 4332567 | 4350837 | LOC_Os03g18840.1 | LOC_Os11g38170.1 | 0.821 |
| 4332567 | LOC_Os02g49270.1 | LOC_Os03g18840.1 | LOC_Os02g49270.1 | 0.784 |
| 4333574 | 4331005 | LOC_Os03g44530.1 | LOC_Os02g55260.1 | 0.499 |
| 4333574 | 4332567 | LOC_Os03g44530.1 | LOC_Os03g18840.1 | 0.912 |
| 4333574 | 4333814 | LOC_Os03g44530.1 | LOC_Os03g49210.1 | 0.985 |
| 4333574 | 4336833 | LOC_Os03g44530.1 | LOC_Os04g50660.1 | 0.871 |
| 4333574 | 4342673 | LOC_Os03g44530.1 | LOC_Os07g10350.1 | 0.719 |
| 4333574 | 4347787 | LOC_Os03g44530.1 | LOC_Os09g37860.1 | 0.866 |
| 4333574 | 4350837 | LOC_Os03g44530.1 | LOC_Os11g38170.1 | 0.532 |
| 4333574 | LOC_Os02g49270.1 | LOC_Os03g44530.1 | LOC_Os02g49270.1 | 0.819 |
| 4333814 | 4330796 | LOC_Os03g49210.1 | LOC_Os02g52250.1 | 0.635 |
| 4333814 | 4331005 | LOC_Os03g49210.1 | LOC_Os02g55260.1 | 0.835 |
| 4333814 | 4332567 | LOC_Os03g49210.1 | LOC_Os03g18840.1 | 0.973 |
| 4333814 | 4333574 | LOC_Os03g49210.1 | LOC_Os03g44530.1 | 0.985 |
| 4333814 | 4336833 | LOC_Os03g49210.1 | LOC_Os04g50660.1 | 0.966 |
| 4333814 | 4342472 | LOC_Os03g49210.1 | LOC_Os07g06980.1 | 0.628 |
| 4333814 | 4342673 | LOC_Os03g49210.1 | LOC_Os07g10350.1 | 0.978 |
| 4333814 | 4347787 | LOC_Os03g49210.1 | LOC_Os09g37860.1 | 0.998 |
| 4333814 | 4350837 | LOC_Os03g49210.1 | LOC_Os11g38170.1 | 0.924 |
| 4333814 | LOC_Os02g49270.1 | LOC_Os03g49210.1 | LOC_Os02g49270.1 | 0.997 |
| 4334214 | 4326546 | LOC_Os03g55560.1 | LOC_Os01g53920.1 | 0.927 |
| 4334214 | 4330866 | LOC_Os03g55560.1 | LOC_Os02g53120.1 | 0.43 |
| 4334214 | 4338531 | LOC_Os03g55560.1 | LOC_Os05g29030.1 | 0.593 |
| 4334214 | 4339763 | LOC_Os03g55560.1 | LOC_Os05g50970.1 | 0.593 |
| 4334214 | 4342017 | LOC_Os03g55560.1 | LOC_Os06g49430.1 | 0.714 |
| 4334214 | 4344698 | LOC_Os03g55560.1 | LOC_Os08g06060.1 | 0.687 |
| 4334214 | 4345065 | LOC_Os03g55560.1 | LOC_Os08g14990.1 | 0.927 |
| 4334214 | 4345628 | LOC_Os03g55560.1 | LOC_Os08g33200.1 | 0.408 |
| 4334214 | 4345708 | LOC_Os03g55560.1 | LOC_Os08g34650.1 | 0.927 |
| 4334214 | 4347005 | LOC_Os03g55560.1 | LOC_Os09g23740.1 | 0.408 |
| 4334214 | 4347095 | LOC_Os03g55560.1 | LOC_Os09g25540.1 | 0.927 |
| 4335058 | 4326546 | LOC_Os04g08740.1 | LOC_Os01g53920.1 | 0.659 |
| 4335058 | 4338096 | LOC_Os04g08740.1 | LOC_Os05g11990.1 | 0.528 |
| 4335058 | 4342017 | LOC_Os04g08740.1 | LOC_Os06g49430.1 | 0.48 |
| 4335058 | 4344698 | LOC_Os04g08740.1 | LOC_Os08g06060.1 | 0.48 |
| 4335058 | 4345065 | LOC_Os04g08740.1 | LOC_Os08g14990.1 | 0.659 |
| 4335058 | 4345708 | LOC_Os04g08740.1 | LOC_Os08g34650.1 | 0.659 |
| 4335058 | 4347095 | LOC_Os04g08740.1 | LOC_Os09g25540.1 | 0.659 |
| 4335089 | 4326270 | LOC_Os04g09860.1 | LOC_Os01g16414.1 | 0.697 |
| 4335089 | 4326546 | LOC_Os04g09860.1 | LOC_Os01g53920.1 | 0.606 |
| 4335089 | 4338096 | LOC_Os04g09860.1 | LOC_Os05g11990.1 | 0.48 |
| 4335089 | 4345065 | LOC_Os04g09860.1 | LOC_Os08g14990.1 | 0.606 |
| 4335089 | 4345628 | LOC_Os04g09860.1 | LOC_Os08g33200.1 | 0.514 |
| 4335089 | 4345708 | LOC_Os04g09860.1 | LOC_Os08g34650.1 | 0.606 |
| 4335089 | 4347005 | LOC_Os04g09860.1 | LOC_Os09g23740.1 | 0.514 |
| 4335089 | 4347095 | LOC_Os04g09860.1 | LOC_Os09g25540.1 | 0.606 |
| 4336833 | 4331005 | LOC_Os04g50660.1 | LOC_Os02g55260.1 | 0.903 |
| 4336833 | 4332567 | LOC_Os04g50660.1 | LOC_Os03g18840.1 | 0.997 |
| 4336833 | 4333574 | LOC_Os04g50660.1 | LOC_Os03g44530.1 | 0.871 |
| 4336833 | 4333814 | LOC_Os04g50660.1 | LOC_Os03g49210.1 | 0.966 |
| 4336833 | 4342673 | LOC_Os04g50660.1 | LOC_Os07g10350.1 | 0.996 |
| 4336833 | 4347787 | LOC_Os04g50660.1 | LOC_Os09g37860.1 | 0.966 |
| 4336833 | 4350837 | LOC_Os04g50660.1 | LOC_Os11g38170.1 | 0.966 |
| 4336833 | LOC_Os02g49270.1 | LOC_Os04g50660.1 | LOC_Os02g49270.1 | 0.968 |
| 4337619 | 4326546 | LOC_Os05g02500.1 | LOC_Os01g53920.1 | 0.817 |
| 4337619 | 4328135 | LOC_Os05g02500.1 | LOC_Os02g03060.1 | 0.496 |
| 4337619 | 4330866 | LOC_Os05g02500.1 | LOC_Os02g53120.1 | 0.422 |
| 4337619 | 4342017 | LOC_Os05g02500.1 | LOC_Os06g49430.1 | 0.873 |
| 4337619 | 4344698 | LOC_Os05g02500.1 | LOC_Os08g06060.1 | 0.873 |
| 4337619 | 4345065 | LOC_Os05g02500.1 | LOC_Os08g14990.1 | 0.817 |
| 4337619 | 4345628 | LOC_Os05g02500.1 | LOC_Os08g33200.1 | 0.936 |
| 4337619 | 4345708 | LOC_Os05g02500.1 | LOC_Os08g34650.1 | 0.817 |
| 4337619 | 4347005 | LOC_Os05g02500.1 | LOC_Os09g23740.1 | 0.936 |
| 4337619 | 4347095 | LOC_Os05g02500.1 | LOC_Os09g25540.1 | 0.817 |
| 4337686 | 4326546 | LOC_Os05g03610.1 | LOC_Os01g53920.1 | 0.726 |
| 4337686 | 4345065 | LOC_Os05g03610.1 | LOC_Os08g14990.1 | 0.726 |
| 4337686 | 4345628 | LOC_Os05g03610.1 | LOC_Os08g33200.1 | 0.946 |
| 4337686 | 4345708 | LOC_Os05g03610.1 | LOC_Os08g34650.1 | 0.726 |
| 4337686 | 4347005 | LOC_Os05g03610.1 | LOC_Os09g23740.1 | 0.946 |
| 4337686 | 4347095 | LOC_Os05g03610.1 | LOC_Os09g25540.1 | 0.726 |
| 4338096 | 4324364 | LOC_Os05g11990.1 | LOC_Os01g69030.2 | 0.671 |
| 4338096 | 4324980 | LOC_Os05g11990.1 | LOC_Os01g67970.1 | 0.666 |
| 4338096 | 4326270 | LOC_Os05g11990.1 | LOC_Os01g16414.1 | 0.582 |
| 4338096 | 4326546 | LOC_Os05g11990.1 | LOC_Os01g53920.1 | 0.968 |
| 4338096 | 4328135 | LOC_Os05g11990.1 | LOC_Os02g03060.1 | 0.598 |
| 4338096 | 4332080 | LOC_Os05g11990.1 | LOC_Os03g11910.1 | 0.928 |
| 4338096 | 4335058 | LOC_Os05g11990.1 | LOC_Os04g08740.1 | 0.528 |
| 4338096 | 4335089 | LOC_Os05g11990.1 | LOC_Os04g09860.1 | 0.48 |
| 4338096 | 4338531 | LOC_Os05g11990.1 | LOC_Os05g29030.1 | 0.654 |
| 4338096 | 4339763 | LOC_Os05g11990.1 | LOC_Os05g50970.1 | 0.654 |
| 4338096 | 4341219 | LOC_Os05g11990.1 | LOC_Os06g34690.1 | 0.661 |
| 4338096 | 4341853 | LOC_Os05g11990.1 | LOC_Os06g46600.1 | 0.928 |
| 4338096 | 4342017 | LOC_Os05g11990.1 | LOC_Os06g49430.1 | 0.439 |
| 4338096 | 4342472 | LOC_Os05g11990.1 | LOC_Os07g06980.1 | 0.476 |
| 4338096 | 4342673 | LOC_Os05g11990.1 | LOC_Os07g10350.1 | 0.457 |
| 4338096 | 4343196 | LOC_Os05g11990.1 | LOC_Os07g28800.1 | 0.972 |
| 4338096 | 4344698 | LOC_Os05g11990.1 | LOC_Os08g06060.1 | 0.439 |
| 4338096 | 4345065 | LOC_Os05g11990.1 | LOC_Os08g14990.1 | 0.968 |
| 4338096 | 4345628 | LOC_Os05g11990.1 | LOC_Os08g33200.1 | 0.677 |
| 4338096 | 4345708 | LOC_Os05g11990.1 | LOC_Os08g34650.1 | 0.968 |
| 4338096 | 4345910 | LOC_Os05g11990.1 | LOC_Os08g38410.1 | 0.726 |
| 4338096 | 4346276 | LOC_Os05g11990.1 | LOC_Os08g44050.1 | 0.474 |
| 4338096 | 4347005 | LOC_Os05g11990.1 | LOC_Os09g23740.1 | 0.677 |
| 4338096 | 4347095 | LOC_Os05g11990.1 | LOC_Os09g25540.1 | 0.968 |
| 4338096 | 4347787 | LOC_Os05g11990.1 | LOC_Os09g37860.1 | 0.4 |
| 4338096 | 4349742 | LOC_Os05g11990.1 | LOC_Os11g04600.1 | 0.79 |
| 4338096 | 4350837 | LOC_Os05g11990.1 | LOC_Os11g38170.1 | 0.571 |
| 4338096 | 4351457 | LOC_Os05g11990.1 | LOC_Os12g04410.1 | 0.79 |
| 4338096 | LOC_Os02g49270.1 | LOC_Os05g11990.1 | LOC_Os02g49270.1 | 0.4 |
| 4338531 | 4324584 | LOC_Os05g29030.1 | LOC_Os01g11920.1 | 0.516 |
| 4338531 | 4326546 | LOC_Os05g29030.1 | LOC_Os01g53920.1 | 0.963 |
| 4338531 | 4334214 | LOC_Os05g29030.1 | LOC_Os03g55560.1 | 0.593 |
| 4338531 | 4338096 | LOC_Os05g29030.1 | LOC_Os05g11990.1 | 0.654 |
| 4338531 | 4342017 | LOC_Os05g29030.1 | LOC_Os06g49430.1 | 0.771 |
| 4338531 | 4344698 | LOC_Os05g29030.1 | LOC_Os08g06060.1 | 0.771 |
| 4338531 | 4345065 | LOC_Os05g29030.1 | LOC_Os08g14990.1 | 0.963 |
| 4338531 | 4345708 | LOC_Os05g29030.1 | LOC_Os08g34650.1 | 0.963 |
| 4338531 | 4347095 | LOC_Os05g29030.1 | LOC_Os09g25540.1 | 0.963 |
| 4338531 | 4347787 | LOC_Os05g29030.1 | LOC_Os09g37860.1 | 0.404 |
| 4338531 | LOC_Os02g49270.1 | LOC_Os05g29030.1 | LOC_Os02g49270.1 | 0.404 |
| 4339763 | 4324584 | LOC_Os05g50970.1 | LOC_Os01g11920.1 | 0.516 |
| 4339763 | 4326546 | LOC_Os05g50970.1 | LOC_Os01g53920.1 | 0.963 |
| 4339763 | 4334214 | LOC_Os05g50970.1 | LOC_Os03g55560.1 | 0.593 |
| 4339763 | 4338096 | LOC_Os05g50970.1 | LOC_Os05g11990.1 | 0.654 |
| 4339763 | 4342017 | LOC_Os05g50970.1 | LOC_Os06g49430.1 | 0.771 |
| 4339763 | 4344698 | LOC_Os05g50970.1 | LOC_Os08g06060.1 | 0.771 |
| 4339763 | 4345065 | LOC_Os05g50970.1 | LOC_Os08g14990.1 | 0.963 |
| 4339763 | 4345708 | LOC_Os05g50970.1 | LOC_Os08g34650.1 | 0.963 |
| 4339763 | 4347095 | LOC_Os05g50970.1 | LOC_Os09g25540.1 | 0.963 |
| 4339763 | 4347787 | LOC_Os05g50970.1 | LOC_Os09g37860.1 | 0.404 |
| 4339763 | LOC_Os02g49270.1 | LOC_Os05g50970.1 | LOC_Os02g49270.1 | 0.404 |
| 4340847 | 4326546 | LOC_Os06g20340.1 | LOC_Os01g53920.1 | 0.817 |
| 4340847 | 4328135 | LOC_Os06g20340.1 | LOC_Os02g03060.1 | 0.496 |
| 4340847 | 4330866 | LOC_Os06g20340.1 | LOC_Os02g53120.1 | 0.422 |
| 4340847 | 4342017 | LOC_Os06g20340.1 | LOC_Os06g49430.1 | 0.873 |
| 4340847 | 4344698 | LOC_Os06g20340.1 | LOC_Os08g06060.1 | 0.873 |
| 4340847 | 4345065 | LOC_Os06g20340.1 | LOC_Os08g14990.1 | 0.817 |
| 4340847 | 4345628 | LOC_Os06g20340.1 | LOC_Os08g33200.1 | 0.936 |
| 4340847 | 4345708 | LOC_Os06g20340.1 | LOC_Os08g34650.1 | 0.817 |
| 4340847 | 4347005 | LOC_Os06g20340.1 | LOC_Os09g23740.1 | 0.936 |
| 4340847 | 4347095 | LOC_Os06g20340.1 | LOC_Os09g25540.1 | 0.817 |
| 4341219 | 4326546 | LOC_Os06g34690.1 | LOC_Os01g53920.1 | 0.641 |
| 4341219 | 4332080 | LOC_Os06g34690.1 | LOC_Os03g11910.1 | 0.843 |
| 4341219 | 4338096 | LOC_Os06g34690.1 | LOC_Os05g11990.1 | 0.661 |
| 4341219 | 4341853 | LOC_Os06g34690.1 | LOC_Os06g46600.1 | 0.843 |
| 4341219 | 4342673 | LOC_Os06g34690.1 | LOC_Os07g10350.1 | 0.492 |
| 4341219 | 4343196 | LOC_Os06g34690.1 | LOC_Os07g28800.1 | 0.575 |
| 4341219 | 4345065 | LOC_Os06g34690.1 | LOC_Os08g14990.1 | 0.641 |
| 4341219 | 4345708 | LOC_Os06g34690.1 | LOC_Os08g34650.1 | 0.641 |
| 4341219 | 4347095 | LOC_Os06g34690.1 | LOC_Os09g25540.1 | 0.641 |
| 4341219 | 4347787 | LOC_Os06g34690.1 | LOC_Os09g37860.1 | 0.468 |
| 4341219 | LOC_Os02g49270.1 | LOC_Os06g34690.1 | LOC_Os02g49270.1 | 0.635 |
| 4341853 | 4324584 | LOC_Os06g46600.1 | LOC_Os01g11920.1 | 0.656 |
| 4341853 | 4326546 | LOC_Os06g46600.1 | LOC_Os01g53920.1 | 0.776 |
| 4341853 | 4332080 | LOC_Os06g46600.1 | LOC_Os03g11910.1 | 0.653 |
| 4341853 | 4338096 | LOC_Os06g46600.1 | LOC_Os05g11990.1 | 0.928 |
| 4341853 | 4341219 | LOC_Os06g46600.1 | LOC_Os06g34690.1 | 0.843 |
| 4341853 | 4342673 | LOC_Os06g46600.1 | LOC_Os07g10350.1 | 0.444 |
| 4341853 | 4343196 | LOC_Os06g46600.1 | LOC_Os07g28800.1 | 0.906 |
| 4341853 | 4345065 | LOC_Os06g46600.1 | LOC_Os08g14990.1 | 0.776 |
| 4341853 | 4345628 | LOC_Os06g46600.1 | LOC_Os08g33200.1 | 0.88 |
| 4341853 | 4345708 | LOC_Os06g46600.1 | LOC_Os08g34650.1 | 0.776 |
| 4341853 | 4347005 | LOC_Os06g46600.1 | LOC_Os09g23740.1 | 0.88 |
| 4341853 | 4347095 | LOC_Os06g46600.1 | LOC_Os09g25540.1 | 0.776 |
| 4342017 | 4324584 | LOC_Os06g49430.1 | LOC_Os01g11920.1 | 0.516 |
| 4342017 | 4324980 | LOC_Os06g49430.1 | LOC_Os01g67970.1 | 0.718 |
| 4342017 | 4326270 | LOC_Os06g49430.1 | LOC_Os01g16414.1 | 0.519 |
| 4342017 | 4326546 | LOC_Os06g49430.1 | LOC_Os01g53920.1 | 0.765 |
| 4342017 | 4328135 | LOC_Os06g49430.1 | LOC_Os02g03060.1 | 0.594 |
| 4342017 | 4334214 | LOC_Os06g49430.1 | LOC_Os03g55560.1 | 0.714 |
| 4342017 | 4335058 | LOC_Os06g49430.1 | LOC_Os04g08740.1 | 0.48 |
| 4342017 | 4337619 | LOC_Os06g49430.1 | LOC_Os05g02500.1 | 0.873 |
| 4342017 | 4338096 | LOC_Os06g49430.1 | LOC_Os05g11990.1 | 0.439 |
| 4342017 | 4338531 | LOC_Os06g49430.1 | LOC_Os05g29030.1 | 0.771 |
| 4342017 | 4339763 | LOC_Os06g49430.1 | LOC_Os05g50970.1 | 0.771 |
| 4342017 | 4340847 | LOC_Os06g49430.1 | LOC_Os06g20340.1 | 0.873 |
| 4342017 | 4345065 | LOC_Os06g49430.1 | LOC_Os08g14990.1 | 0.764 |
| 4342017 | 4345628 | LOC_Os06g49430.1 | LOC_Os08g33200.1 | 0.519 |
| 4342017 | 4345708 | LOC_Os06g49430.1 | LOC_Os08g34650.1 | 0.762 |
| 4342017 | 4346276 | LOC_Os06g49430.1 | LOC_Os08g44050.1 | 0.693 |
| 4342017 | 4347005 | LOC_Os06g49430.1 | LOC_Os09g23740.1 | 0.519 |
| 4342017 | 4347095 | LOC_Os06g49430.1 | LOC_Os09g25540.1 | 0.764 |
| 4342017 | 4349742 | LOC_Os06g49430.1 | LOC_Os11g04600.1 | 0.476 |
| 4342017 | 4351431 | LOC_Os06g49430.1 | LOC_Os12g03990.1 | 0.873 |
| 4342017 | 4351457 | LOC_Os06g49430.1 | LOC_Os12g04410.1 | 0.476 |
| 4342472 | 4324481 | LOC_Os07g06980.1 | LOC_Os03g17100.1 | 0.657 |
| 4342472 | 4324495 | LOC_Os07g06980.1 | LOC_Os01g05900.1 | 0.774 |
| 4342472 | 4324500 | LOC_Os07g06980.1 | LOC_Os01g05630.1 | 0.774 |
| 4342472 | 4324584 | LOC_Os07g06980.1 | LOC_Os01g11920.1 | 0.475 |
| 4342472 | 4324980 | LOC_Os07g06980.1 | LOC_Os01g67970.1 | 0.81 |
| 4342472 | 4326270 | LOC_Os07g06980.1 | LOC_Os01g16414.1 | 0.433 |
| 4342472 | 4330796 | LOC_Os07g06980.1 | LOC_Os02g52250.1 | 0.604 |
| 4342472 | 4333814 | LOC_Os07g06980.1 | LOC_Os03g49210.1 | 0.628 |
| 4342472 | 4338096 | LOC_Os07g06980.1 | LOC_Os05g11990.1 | 0.476 |
| 4342472 | 4346276 | LOC_Os07g06980.1 | LOC_Os08g44050.1 | 0.496 |
| 4342673 | 4331005 | LOC_Os07g10350.1 | LOC_Os02g55260.1 | 0.769 |
| 4342673 | 4332080 | LOC_Os07g10350.1 | LOC_Os03g11910.1 | 0.437 |
| 4342673 | 4332567 | LOC_Os07g10350.1 | LOC_Os03g18840.1 | 0.983 |
| 4342673 | 4333574 | LOC_Os07g10350.1 | LOC_Os03g44530.1 | 0.719 |
| 4342673 | 4333814 | LOC_Os07g10350.1 | LOC_Os03g49210.1 | 0.978 |
| 4342673 | 4336833 | LOC_Os07g10350.1 | LOC_Os04g50660.1 | 0.996 |
| 4342673 | 4338096 | LOC_Os07g10350.1 | LOC_Os05g11990.1 | 0.457 |
| 4342673 | 4341219 | LOC_Os07g10350.1 | LOC_Os06g34690.1 | 0.492 |
| 4342673 | 4341853 | LOC_Os07g10350.1 | LOC_Os06g46600.1 | 0.444 |
| 4342673 | 4345910 | LOC_Os07g10350.1 | LOC_Os08g38410.1 | 0.408 |
| 4342673 | 4347787 | LOC_Os07g10350.1 | LOC_Os09g37860.1 | 0.997 |
| 4342673 | 4350837 | LOC_Os07g10350.1 | LOC_Os11g38170.1 | 0.619 |
| 4342673 | LOC_Os02g49270.1 | LOC_Os07g10350.1 | LOC_Os02g49270.1 | 0.988 |
| 4343196 | 4324584 | LOC_Os07g28800.1 | LOC_Os01g11920.1 | 0.669 |
| 4343196 | 4332080 | LOC_Os07g28800.1 | LOC_Os03g11910.1 | 0.91 |
| 4343196 | 4338096 | LOC_Os07g28800.1 | LOC_Os05g11990.1 | 0.972 |
| 4343196 | 4341219 | LOC_Os07g28800.1 | LOC_Os06g34690.1 | 0.575 |
| 4343196 | 4341853 | LOC_Os07g28800.1 | LOC_Os06g46600.1 | 0.906 |
| 4343196 | 4345628 | LOC_Os07g28800.1 | LOC_Os08g33200.1 | 0.636 |
| 4343196 | 4347005 | LOC_Os07g28800.1 | LOC_Os09g23740.1 | 0.636 |
| 4344698 | 4324584 | LOC_Os08g06060.1 | LOC_Os01g11920.1 | 0.516 |
| 4344698 | 4324980 | LOC_Os08g06060.1 | LOC_Os01g67970.1 | 0.718 |
| 4344698 | 4326270 | LOC_Os08g06060.1 | LOC_Os01g16414.1 | 0.519 |
| 4344698 | 4326546 | LOC_Os08g06060.1 | LOC_Os01g53920.1 | 0.846 |
| 4344698 | 4328135 | LOC_Os08g06060.1 | LOC_Os02g03060.1 | 0.619 |
| 4344698 | 4334214 | LOC_Os08g06060.1 | LOC_Os03g55560.1 | 0.687 |
| 4344698 | 4335058 | LOC_Os08g06060.1 | LOC_Os04g08740.1 | 0.48 |
| 4344698 | 4337619 | LOC_Os08g06060.1 | LOC_Os05g02500.1 | 0.873 |
| 4344698 | 4338096 | LOC_Os08g06060.1 | LOC_Os05g11990.1 | 0.439 |
| 4344698 | 4338531 | LOC_Os08g06060.1 | LOC_Os05g29030.1 | 0.771 |
| 4344698 | 4339763 | LOC_Os08g06060.1 | LOC_Os05g50970.1 | 0.771 |
| 4344698 | 4340847 | LOC_Os08g06060.1 | LOC_Os06g20340.1 | 0.873 |
| 4344698 | 4345065 | LOC_Os08g06060.1 | LOC_Os08g14990.1 | 0.846 |
| 4344698 | 4345628 | LOC_Os08g06060.1 | LOC_Os08g33200.1 | 0.519 |
| 4344698 | 4345708 | LOC_Os08g06060.1 | LOC_Os08g34650.1 | 0.762 |
| 4344698 | 4346276 | LOC_Os08g06060.1 | LOC_Os08g44050.1 | 0.693 |
| 4344698 | 4347005 | LOC_Os08g06060.1 | LOC_Os09g23740.1 | 0.519 |
| 4344698 | 4347095 | LOC_Os08g06060.1 | LOC_Os09g25540.1 | 0.846 |
| 4344698 | 4349742 | LOC_Os08g06060.1 | LOC_Os11g04600.1 | 0.476 |
| 4344698 | 4351431 | LOC_Os08g06060.1 | LOC_Os12g03990.1 | 0.873 |
| 4344698 | 4351457 | LOC_Os08g06060.1 | LOC_Os12g04410.1 | 0.476 |
| 4345065 | 4324364 | LOC_Os08g14990.1 | LOC_Os01g69030.2 | 0.634 |
| 4345065 | 4324584 | LOC_Os08g14990.1 | LOC_Os01g11920.1 | 0.691 |
| 4345065 | 4324980 | LOC_Os08g14990.1 | LOC_Os01g67970.1 | 0.703 |
| 4345065 | 4326270 | LOC_Os08g14990.1 | LOC_Os01g16414.1 | 0.606 |
| 4345065 | 4326546 | LOC_Os08g14990.1 | LOC_Os01g53920.1 | 0.956 |
| 4345065 | 4327983 | LOC_Os08g14990.1 | LOC_Os02g01170.1 | 0.655 |
| 4345065 | 4328135 | LOC_Os08g14990.1 | LOC_Os02g03060.1 | 0.675 |
| 4345065 | 4332080 | LOC_Os08g14990.1 | LOC_Os03g11910.1 | 0.776 |
| 4345065 | 4334214 | LOC_Os08g14990.1 | LOC_Os03g55560.1 | 0.927 |
| 4345065 | 4335058 | LOC_Os08g14990.1 | LOC_Os04g08740.1 | 0.659 |
| 4345065 | 4335089 | LOC_Os08g14990.1 | LOC_Os04g09860.1 | 0.606 |
| 4345065 | 4337619 | LOC_Os08g14990.1 | LOC_Os05g02500.1 | 0.817 |
| 4345065 | 4337686 | LOC_Os08g14990.1 | LOC_Os05g03610.1 | 0.726 |
| 4345065 | 4338096 | LOC_Os08g14990.1 | LOC_Os05g11990.1 | 0.968 |
| 4345065 | 4338531 | LOC_Os08g14990.1 | LOC_Os05g29030.1 | 0.963 |
| 4345065 | 4339763 | LOC_Os08g14990.1 | LOC_Os05g50970.1 | 0.963 |
| 4345065 | 4340847 | LOC_Os08g14990.1 | LOC_Os06g20340.1 | 0.817 |
| 4345065 | 4341219 | LOC_Os08g14990.1 | LOC_Os06g34690.1 | 0.641 |
| 4345065 | 4341853 | LOC_Os08g14990.1 | LOC_Os06g46600.1 | 0.776 |
| 4345065 | 4342017 | LOC_Os08g14990.1 | LOC_Os06g49430.1 | 0.764 |
| 4345065 | 4344698 | LOC_Os08g14990.1 | LOC_Os08g06060.1 | 0.846 |
| 4345065 | 4345628 | LOC_Os08g14990.1 | LOC_Os08g33200.1 | 0.985 |
| 4345065 | 4345708 | LOC_Os08g14990.1 | LOC_Os08g34650.1 | 0.952 |
| 4345065 | 4345910 | LOC_Os08g14990.1 | LOC_Os08g38410.1 | 0.628 |
| 4345065 | 4346276 | LOC_Os08g14990.1 | LOC_Os08g44050.1 | 0.703 |
| 4345065 | 4347005 | LOC_Os08g14990.1 | LOC_Os09g23740.1 | 0.985 |
| 4345065 | 4347095 | LOC_Os08g14990.1 | LOC_Os09g25540.1 | 0.951 |
| 4345065 | 4347787 | LOC_Os08g14990.1 | LOC_Os09g37860.1 | 0.537 |
| 4345065 | 4349742 | LOC_Os08g14990.1 | LOC_Os11g04600.1 | 0.837 |
| 4345065 | 4350472 | LOC_Os08g14990.1 | LOC_Os11g27264.1 | 0.614 |
| 4345065 | 4350473 | LOC_Os08g14990.1 | LOC_Os11g27329.1 | 0.614 |
| 4345065 | 4351431 | LOC_Os08g14990.1 | LOC_Os12g03990.1 | 0.817 |
| 4345065 | 4351457 | LOC_Os08g14990.1 | LOC_Os12g04410.1 | 0.837 |
| 4345065 | LOC_Os02g49270.1 | LOC_Os08g14990.1 | LOC_Os02g49270.1 | 0.537 |
| 4345628 | 4326270 | LOC_Os08g33200.1 | LOC_Os01g16414.1 | 0.514 |
| 4345628 | 4326546 | LOC_Os08g33200.1 | LOC_Os01g53920.1 | 0.985 |
| 4345628 | 4332080 | LOC_Os08g33200.1 | LOC_Os03g11910.1 | 0.88 |
| 4345628 | 4334214 | LOC_Os08g33200.1 | LOC_Os03g55560.1 | 0.408 |
| 4345628 | 4335089 | LOC_Os08g33200.1 | LOC_Os04g09860.1 | 0.514 |
| 4345628 | 4337619 | LOC_Os08g33200.1 | LOC_Os05g02500.1 | 0.936 |
| 4345628 | 4337686 | LOC_Os08g33200.1 | LOC_Os05g03610.1 | 0.946 |
| 4345628 | 4338096 | LOC_Os08g33200.1 | LOC_Os05g11990.1 | 0.677 |
| 4345628 | 4340847 | LOC_Os08g33200.1 | LOC_Os06g20340.1 | 0.936 |
| 4345628 | 4341853 | LOC_Os08g33200.1 | LOC_Os06g46600.1 | 0.88 |
| 4345628 | 4342017 | LOC_Os08g33200.1 | LOC_Os06g49430.1 | 0.519 |
| 4345628 | 4343196 | LOC_Os08g33200.1 | LOC_Os07g28800.1 | 0.636 |
| 4345628 | 4344698 | LOC_Os08g33200.1 | LOC_Os08g06060.1 | 0.519 |
| 4345628 | 4345065 | LOC_Os08g33200.1 | LOC_Os08g14990.1 | 0.985 |
| 4345628 | 4345708 | LOC_Os08g33200.1 | LOC_Os08g34650.1 | 0.985 |
| 4345628 | 4347005 | LOC_Os08g33200.1 | LOC_Os09g23740.1 | 0.895 |
| 4345628 | 4347095 | LOC_Os08g33200.1 | LOC_Os09g25540.1 | 0.985 |
| 4345628 | 4349742 | LOC_Os08g33200.1 | LOC_Os11g04600.1 | 0.542 |
| 4345628 | 4351431 | LOC_Os08g33200.1 | LOC_Os12g03990.1 | 0.936 |
| 4345628 | 4351457 | LOC_Os08g33200.1 | LOC_Os12g04410.1 | 0.542 |
| 4345708 | 4324364 | LOC_Os08g34650.1 | LOC_Os01g69030.2 | 0.634 |
| 4345708 | 4324584 | LOC_Os08g34650.1 | LOC_Os01g11920.1 | 0.691 |
| 4345708 | 4324980 | LOC_Os08g34650.1 | LOC_Os01g67970.1 | 0.703 |
| 4345708 | 4326270 | LOC_Os08g34650.1 | LOC_Os01g16414.1 | 0.606 |
| 4345708 | 4326546 | LOC_Os08g34650.1 | LOC_Os01g53920.1 | 0.953 |
| 4345708 | 4327983 | LOC_Os08g34650.1 | LOC_Os02g01170.1 | 0.655 |
| 4345708 | 4328135 | LOC_Os08g34650.1 | LOC_Os02g03060.1 | 0.671 |
| 4345708 | 4332080 | LOC_Os08g34650.1 | LOC_Os03g11910.1 | 0.776 |
| 4345708 | 4334214 | LOC_Os08g34650.1 | LOC_Os03g55560.1 | 0.927 |
| 4345708 | 4335058 | LOC_Os08g34650.1 | LOC_Os04g08740.1 | 0.659 |
| 4345708 | 4335089 | LOC_Os08g34650.1 | LOC_Os04g09860.1 | 0.606 |
| 4345708 | 4337619 | LOC_Os08g34650.1 | LOC_Os05g02500.1 | 0.817 |
| 4345708 | 4337686 | LOC_Os08g34650.1 | LOC_Os05g03610.1 | 0.726 |
| 4345708 | 4338096 | LOC_Os08g34650.1 | LOC_Os05g11990.1 | 0.968 |
| 4345708 | 4338531 | LOC_Os08g34650.1 | LOC_Os05g29030.1 | 0.963 |
| 4345708 | 4339763 | LOC_Os08g34650.1 | LOC_Os05g50970.1 | 0.963 |
| 4345708 | 4340847 | LOC_Os08g34650.1 | LOC_Os06g20340.1 | 0.817 |
| 4345708 | 4341219 | LOC_Os08g34650.1 | LOC_Os06g34690.1 | 0.641 |
| 4345708 | 4341853 | LOC_Os08g34650.1 | LOC_Os06g46600.1 | 0.776 |
| 4345708 | 4342017 | LOC_Os08g34650.1 | LOC_Os06g49430.1 | 0.762 |
| 4345708 | 4344698 | LOC_Os08g34650.1 | LOC_Os08g06060.1 | 0.762 |
| 4345708 | 4345065 | LOC_Os08g34650.1 | LOC_Os08g14990.1 | 0.952 |
| 4345708 | 4345628 | LOC_Os08g34650.1 | LOC_Os08g33200.1 | 0.985 |
| 4345708 | 4345910 | LOC_Os08g34650.1 | LOC_Os08g38410.1 | 0.628 |
| 4345708 | 4346276 | LOC_Os08g34650.1 | LOC_Os08g44050.1 | 0.703 |
| 4345708 | 4347005 | LOC_Os08g34650.1 | LOC_Os09g23740.1 | 0.985 |
| 4345708 | 4347095 | LOC_Os08g34650.1 | LOC_Os09g25540.1 | 0.953 |
| 4345708 | 4347787 | LOC_Os08g34650.1 | LOC_Os09g37860.1 | 0.537 |
| 4345708 | 4349742 | LOC_Os08g34650.1 | LOC_Os11g04600.1 | 0.837 |
| 4345708 | 4350472 | LOC_Os08g34650.1 | LOC_Os11g27264.1 | 0.614 |
| 4345708 | 4350473 | LOC_Os08g34650.1 | LOC_Os11g27329.1 | 0.614 |
| 4345708 | 4351431 | LOC_Os08g34650.1 | LOC_Os12g03990.1 | 0.817 |
| 4345708 | 4351457 | LOC_Os08g34650.1 | LOC_Os12g04410.1 | 0.837 |
| 4345708 | LOC_Os02g49270.1 | LOC_Os08g34650.1 | LOC_Os02g49270.1 | 0.537 |
| 4345910 | 4326546 | LOC_Os08g38410.1 | LOC_Os01g53920.1 | 0.628 |
| 4345910 | 4330796 | LOC_Os08g38410.1 | LOC_Os02g52250.1 | 0.664 |
| 4345910 | 4338096 | LOC_Os08g38410.1 | LOC_Os05g11990.1 | 0.726 |
| 4345910 | 4342673 | LOC_Os08g38410.1 | LOC_Os07g10350.1 | 0.408 |
| 4345910 | 4345065 | LOC_Os08g38410.1 | LOC_Os08g14990.1 | 0.628 |
| 4345910 | 4345708 | LOC_Os08g38410.1 | LOC_Os08g34650.1 | 0.628 |
| 4345910 | 4347095 | LOC_Os08g38410.1 | LOC_Os09g25540.1 | 0.628 |
| 4346276 | 4324584 | LOC_Os08g44050.1 | LOC_Os01g11920.1 | 0.478 |
| 4346276 | 4326270 | LOC_Os08g44050.1 | LOC_Os01g16414.1 | 0.549 |
| 4346276 | 4326546 | LOC_Os08g44050.1 | LOC_Os01g53920.1 | 0.703 |
| 4346276 | 4328135 | LOC_Os08g44050.1 | LOC_Os02g03060.1 | 0.612 |
| 4346276 | 4338096 | LOC_Os08g44050.1 | LOC_Os05g11990.1 | 0.474 |
| 4346276 | 4342017 | LOC_Os08g44050.1 | LOC_Os06g49430.1 | 0.693 |
| 4346276 | 4342472 | LOC_Os08g44050.1 | LOC_Os07g06980.1 | 0.496 |
| 4346276 | 4344698 | LOC_Os08g44050.1 | LOC_Os08g06060.1 | 0.693 |
| 4346276 | 4345065 | LOC_Os08g44050.1 | LOC_Os08g14990.1 | 0.703 |
| 4346276 | 4345708 | LOC_Os08g44050.1 | LOC_Os08g34650.1 | 0.703 |
| 4346276 | 4347095 | LOC_Os08g44050.1 | LOC_Os09g25540.1 | 0.703 |
| 4347005 | 4326270 | LOC_Os09g23740.1 | LOC_Os01g16414.1 | 0.514 |
| 4347005 | 4326546 | LOC_Os09g23740.1 | LOC_Os01g53920.1 | 0.985 |
| 4347005 | 4332080 | LOC_Os09g23740.1 | LOC_Os03g11910.1 | 0.88 |
| 4347005 | 4334214 | LOC_Os09g23740.1 | LOC_Os03g55560.1 | 0.408 |
| 4347005 | 4335089 | LOC_Os09g23740.1 | LOC_Os04g09860.1 | 0.514 |
| 4347005 | 4337619 | LOC_Os09g23740.1 | LOC_Os05g02500.1 | 0.936 |
| 4347005 | 4337686 | LOC_Os09g23740.1 | LOC_Os05g03610.1 | 0.946 |
| 4347005 | 4338096 | LOC_Os09g23740.1 | LOC_Os05g11990.1 | 0.677 |
| 4347005 | 4340847 | LOC_Os09g23740.1 | LOC_Os06g20340.1 | 0.936 |
| 4347005 | 4341853 | LOC_Os09g23740.1 | LOC_Os06g46600.1 | 0.88 |
| 4347005 | 4342017 | LOC_Os09g23740.1 | LOC_Os06g49430.1 | 0.519 |
| 4347005 | 4343196 | LOC_Os09g23740.1 | LOC_Os07g28800.1 | 0.636 |
| 4347005 | 4344698 | LOC_Os09g23740.1 | LOC_Os08g06060.1 | 0.519 |
| 4347005 | 4345065 | LOC_Os09g23740.1 | LOC_Os08g14990.1 | 0.985 |
| 4347005 | 4345628 | LOC_Os09g23740.1 | LOC_Os08g33200.1 | 0.895 |
| 4347005 | 4345708 | LOC_Os09g23740.1 | LOC_Os08g34650.1 | 0.985 |
| 4347005 | 4347095 | LOC_Os09g23740.1 | LOC_Os09g25540.1 | 0.985 |
| 4347005 | 4349742 | LOC_Os09g23740.1 | LOC_Os11g04600.1 | 0.542 |
| 4347005 | 4351431 | LOC_Os09g23740.1 | LOC_Os12g03990.1 | 0.936 |
| 4347005 | 4351457 | LOC_Os09g23740.1 | LOC_Os12g04410.1 | 0.542 |
| 4347095 | 4324364 | LOC_Os09g25540.1 | LOC_Os01g69030.2 | 0.634 |
| 4347095 | 4324584 | LOC_Os09g25540.1 | LOC_Os01g11920.1 | 0.691 |
| 4347095 | 4324980 | LOC_Os09g25540.1 | LOC_Os01g67970.1 | 0.703 |
| 4347095 | 4326270 | LOC_Os09g25540.1 | LOC_Os01g16414.1 | 0.606 |
| 4347095 | 4326546 | LOC_Os09g25540.1 | LOC_Os01g53920.1 | 0.956 |
| 4347095 | 4327983 | LOC_Os09g25540.1 | LOC_Os02g01170.1 | 0.655 |
| 4347095 | 4328135 | LOC_Os09g25540.1 | LOC_Os02g03060.1 | 0.673 |
| 4347095 | 4332080 | LOC_Os09g25540.1 | LOC_Os03g11910.1 | 0.776 |
| 4347095 | 4334214 | LOC_Os09g25540.1 | LOC_Os03g55560.1 | 0.927 |
| 4347095 | 4335058 | LOC_Os09g25540.1 | LOC_Os04g08740.1 | 0.659 |
| 4347095 | 4335089 | LOC_Os09g25540.1 | LOC_Os04g09860.1 | 0.606 |
| 4347095 | 4337619 | LOC_Os09g25540.1 | LOC_Os05g02500.1 | 0.817 |
| 4347095 | 4337686 | LOC_Os09g25540.1 | LOC_Os05g03610.1 | 0.726 |
| 4347095 | 4338096 | LOC_Os09g25540.1 | LOC_Os05g11990.1 | 0.968 |
| 4347095 | 4338531 | LOC_Os09g25540.1 | LOC_Os05g29030.1 | 0.963 |
| 4347095 | 4339763 | LOC_Os09g25540.1 | LOC_Os05g50970.1 | 0.963 |
| 4347095 | 4340847 | LOC_Os09g25540.1 | LOC_Os06g20340.1 | 0.817 |
| 4347095 | 4341219 | LOC_Os09g25540.1 | LOC_Os06g34690.1 | 0.641 |
| 4347095 | 4341853 | LOC_Os09g25540.1 | LOC_Os06g46600.1 | 0.776 |
| 4347095 | 4342017 | LOC_Os09g25540.1 | LOC_Os06g49430.1 | 0.764 |
| 4347095 | 4344698 | LOC_Os09g25540.1 | LOC_Os08g06060.1 | 0.846 |
| 4347095 | 4345065 | LOC_Os09g25540.1 | LOC_Os08g14990.1 | 0.951 |
| 4347095 | 4345628 | LOC_Os09g25540.1 | LOC_Os08g33200.1 | 0.985 |
| 4347095 | 4345708 | LOC_Os09g25540.1 | LOC_Os08g34650.1 | 0.953 |
| 4347095 | 4345910 | LOC_Os09g25540.1 | LOC_Os08g38410.1 | 0.628 |
| 4347095 | 4346276 | LOC_Os09g25540.1 | LOC_Os08g44050.1 | 0.703 |
| 4347095 | 4347005 | LOC_Os09g25540.1 | LOC_Os09g23740.1 | 0.985 |
| 4347095 | 4347787 | LOC_Os09g25540.1 | LOC_Os09g37860.1 | 0.537 |
| 4347095 | 4349742 | LOC_Os09g25540.1 | LOC_Os11g04600.1 | 0.837 |
| 4347095 | 4350472 | LOC_Os09g25540.1 | LOC_Os11g27264.1 | 0.614 |
| 4347095 | 4350473 | LOC_Os09g25540.1 | LOC_Os11g27329.1 | 0.614 |
| 4347095 | 4351431 | LOC_Os09g25540.1 | LOC_Os12g03990.1 | 0.817 |
| 4347095 | 4351457 | LOC_Os09g25540.1 | LOC_Os12g04410.1 | 0.837 |
| 4347095 | LOC_Os02g49270.1 | LOC_Os09g25540.1 | LOC_Os02g49270.1 | 0.537 |
| 4347787 | 4326546 | LOC_Os09g37860.1 | LOC_Os01g53920.1 | 0.537 |
| 4347787 | 4331005 | LOC_Os09g37860.1 | LOC_Os02g55260.1 | 0.716 |
| 4347787 | 4332567 | LOC_Os09g37860.1 | LOC_Os03g18840.1 | 0.909 |
| 4347787 | 4333574 | LOC_Os09g37860.1 | LOC_Os03g44530.1 | 0.866 |
| 4347787 | 4333814 | LOC_Os09g37860.1 | LOC_Os03g49210.1 | 0.998 |
| 4347787 | 4336833 | LOC_Os09g37860.1 | LOC_Os04g50660.1 | 0.966 |
| 4347787 | 4338096 | LOC_Os09g37860.1 | LOC_Os05g11990.1 | 0.4 |
| 4347787 | 4338531 | LOC_Os09g37860.1 | LOC_Os05g29030.1 | 0.404 |
| 4347787 | 4339763 | LOC_Os09g37860.1 | LOC_Os05g50970.1 | 0.404 |
| 4347787 | 4341219 | LOC_Os09g37860.1 | LOC_Os06g34690.1 | 0.468 |
| 4347787 | 4342673 | LOC_Os09g37860.1 | LOC_Os07g10350.1 | 0.997 |
| 4347787 | 4345065 | LOC_Os09g37860.1 | LOC_Os08g14990.1 | 0.537 |
| 4347787 | 4345708 | LOC_Os09g37860.1 | LOC_Os08g34650.1 | 0.537 |
| 4347787 | 4347095 | LOC_Os09g37860.1 | LOC_Os09g25540.1 | 0.537 |
| 4347787 | 4350837 | LOC_Os09g37860.1 | LOC_Os11g38170.1 | 0.743 |
| 4347787 | LOC_Os02g49270.1 | LOC_Os09g37860.1 | LOC_Os02g49270.1 | 0.837 |
| 4349742 | 4324584 | LOC_Os11g04600.1 | LOC_Os01g11920.1 | 0.595 |
| 4349742 | 4326546 | LOC_Os11g04600.1 | LOC_Os01g53920.1 | 0.838 |
| 4349742 | 4328135 | LOC_Os11g04600.1 | LOC_Os02g03060.1 | 0.678 |
| 4349742 | 4338096 | LOC_Os11g04600.1 | LOC_Os05g11990.1 | 0.79 |
| 4349742 | 4342017 | LOC_Os11g04600.1 | LOC_Os06g49430.1 | 0.476 |
| 4349742 | 4344698 | LOC_Os11g04600.1 | LOC_Os08g06060.1 | 0.476 |
| 4349742 | 4345065 | LOC_Os11g04600.1 | LOC_Os08g14990.1 | 0.837 |
| 4349742 | 4345628 | LOC_Os11g04600.1 | LOC_Os08g33200.1 | 0.542 |
| 4349742 | 4345708 | LOC_Os11g04600.1 | LOC_Os08g34650.1 | 0.837 |
| 4349742 | 4347005 | LOC_Os11g04600.1 | LOC_Os09g23740.1 | 0.542 |
| 4349742 | 4347095 | LOC_Os11g04600.1 | LOC_Os09g25540.1 | 0.837 |
| 4350472 | 4326546 | LOC_Os11g27264.1 | LOC_Os01g53920.1 | 0.614 |
| 4350472 | 4345065 | LOC_Os11g27264.1 | LOC_Os08g14990.1 | 0.614 |
| 4350472 | 4345708 | LOC_Os11g27264.1 | LOC_Os08g34650.1 | 0.614 |
| 4350472 | 4347095 | LOC_Os11g27264.1 | LOC_Os09g25540.1 | 0.614 |
| 4350473 | 4326546 | LOC_Os11g27329.1 | LOC_Os01g53920.1 | 0.614 |
| 4350473 | 4345065 | LOC_Os11g27329.1 | LOC_Os08g14990.1 | 0.614 |
| 4350473 | 4345708 | LOC_Os11g27329.1 | LOC_Os08g34650.1 | 0.614 |
| 4350473 | 4347095 | LOC_Os11g27329.1 | LOC_Os09g25540.1 | 0.614 |
| 4350837 | 4324584 | LOC_Os11g38170.1 | LOC_Os01g11920.1 | 0.418 |
| 4350837 | 4331005 | LOC_Os11g38170.1 | LOC_Os02g55260.1 | 0.492 |
| 4350837 | 4332567 | LOC_Os11g38170.1 | LOC_Os03g18840.1 | 0.821 |
| 4350837 | 4333574 | LOC_Os11g38170.1 | LOC_Os03g44530.1 | 0.532 |
| 4350837 | 4333814 | LOC_Os11g38170.1 | LOC_Os03g49210.1 | 0.924 |
| 4350837 | 4336833 | LOC_Os11g38170.1 | LOC_Os04g50660.1 | 0.966 |
| 4350837 | 4338096 | LOC_Os11g38170.1 | LOC_Os05g11990.1 | 0.571 |
| 4350837 | 4342673 | LOC_Os11g38170.1 | LOC_Os07g10350.1 | 0.619 |
| 4350837 | 4347787 | LOC_Os11g38170.1 | LOC_Os09g37860.1 | 0.743 |
| 4350837 | LOC_Os02g49270.1 | LOC_Os11g38170.1 | LOC_Os02g49270.1 | 0.721 |
| 4351431 | 4324980 | LOC_Os12g03990.1 | LOC_Os01g67970.1 | 0.459 |
| 4351431 | 4326546 | LOC_Os12g03990.1 | LOC_Os01g53920.1 | 0.817 |
| 4351431 | 4328135 | LOC_Os12g03990.1 | LOC_Os02g03060.1 | 0.496 |
| 4351431 | 4330866 | LOC_Os12g03990.1 | LOC_Os02g53120.1 | 0.422 |
| 4351431 | 4342017 | LOC_Os12g03990.1 | LOC_Os06g49430.1 | 0.873 |
| 4351431 | 4344698 | LOC_Os12g03990.1 | LOC_Os08g06060.1 | 0.873 |
| 4351431 | 4345065 | LOC_Os12g03990.1 | LOC_Os08g14990.1 | 0.817 |
| 4351431 | 4345628 | LOC_Os12g03990.1 | LOC_Os08g33200.1 | 0.936 |
| 4351431 | 4345708 | LOC_Os12g03990.1 | LOC_Os08g34650.1 | 0.817 |
| 4351431 | 4347005 | LOC_Os12g03990.1 | LOC_Os09g23740.1 | 0.936 |
| 4351431 | 4347095 | LOC_Os12g03990.1 | LOC_Os09g25540.1 | 0.817 |
| 4351457 | 4324584 | LOC_Os12g04410.1 | LOC_Os01g11920.1 | 0.595 |
| 4351457 | 4326546 | LOC_Os12g04410.1 | LOC_Os01g53920.1 | 0.838 |
| 4351457 | 4328135 | LOC_Os12g04410.1 | LOC_Os02g03060.1 | 0.678 |
| 4351457 | 4338096 | LOC_Os12g04410.1 | LOC_Os05g11990.1 | 0.79 |
| 4351457 | 4342017 | LOC_Os12g04410.1 | LOC_Os06g49430.1 | 0.476 |
| 4351457 | 4344698 | LOC_Os12g04410.1 | LOC_Os08g06060.1 | 0.476 |
| 4351457 | 4345065 | LOC_Os12g04410.1 | LOC_Os08g14990.1 | 0.837 |
| 4351457 | 4345628 | LOC_Os12g04410.1 | LOC_Os08g33200.1 | 0.542 |
| 4351457 | 4345708 | LOC_Os12g04410.1 | LOC_Os08g34650.1 | 0.837 |
| 4351457 | 4347005 | LOC_Os12g04410.1 | LOC_Os09g23740.1 | 0.542 |
| 4351457 | 4347095 | LOC_Os12g04410.1 | LOC_Os09g25540.1 | 0.837 |
| LOC_Os02g49270.1 | 4326546 | LOC_Os02g49270.1 | LOC_Os01g53920.1 | 0.537 |
| LOC_Os02g49270.1 | 4331005 | LOC_Os02g49270.1 | LOC_Os02g55260.1 | 0.679 |
| LOC_Os02g49270.1 | 4332567 | LOC_Os02g49270.1 | LOC_Os03g18840.1 | 0.784 |
| LOC_Os02g49270.1 | 4333574 | LOC_Os02g49270.1 | LOC_Os03g44530.1 | 0.819 |
| LOC_Os02g49270.1 | 4333814 | LOC_Os02g49270.1 | LOC_Os03g49210.1 | 0.997 |
| LOC_Os02g49270.1 | 4336833 | LOC_Os02g49270.1 | LOC_Os04g50660.1 | 0.968 |
| LOC_Os02g49270.1 | 4338096 | LOC_Os02g49270.1 | LOC_Os05g11990.1 | 0.4 |
| LOC_Os02g49270.1 | 4338531 | LOC_Os02g49270.1 | LOC_Os05g29030.1 | 0.404 |
| LOC_Os02g49270.1 | 4339763 | LOC_Os02g49270.1 | LOC_Os05g50970.1 | 0.404 |
| LOC_Os02g49270.1 | 4341219 | LOC_Os02g49270.1 | LOC_Os06g34690.1 | 0.635 |
| LOC_Os02g49270.1 | 4342673 | LOC_Os02g49270.1 | LOC_Os07g10350.1 | 0.988 |
| LOC_Os02g49270.1 | 4345065 | LOC_Os02g49270.1 | LOC_Os08g14990.1 | 0.537 |
| LOC_Os02g49270.1 | 4345708 | LOC_Os02g49270.1 | LOC_Os08g34650.1 | 0.537 |
| LOC_Os02g49270.1 | 4347095 | LOC_Os02g49270.1 | LOC_Os09g25540.1 | 0.537 |
| LOC_Os02g49270.1 | 4347787 | LOC_Os02g49270.1 | LOC_Os09g37860.1 | 0.837 |
| LOC_Os02g49270.1 | 4350837 | LOC_Os02g49270.1 | LOC_Os11g38170.1 | 0.721 |

Table S3. Biological Process (GO)

| #pathway ID | pathway description | observed gene count | false discovery rate | matching proteins in your network (IDs) |
| --- | --- | --- | --- | --- |
| GO.0006464 | cellular protein modification process | 7 | 2.93E-05 | LOC_Os01g67970.1,LOC_Os02g03060.1,LOC_Os02g55260.1,LOC_Os05g29030.1,LOC_Os05g50970.1,LOC_Os06g49430.1,LOC_Os08g06060.1 |
| GO.0006325 | chromatin organization | 4 | 0.000278 | LOC_Os01g05630.1,LOC_Os01g16414.1,LOC_Os01g67970.1,LOC_Os04g09860.1 |
| GO.0006996 | organelle organization | 5 | 0.000376 | LOC_Os01g05630.1,LOC_Os01g16414.1,LOC_Os01g67970.1,LOC_Os02g55260.1,LOC_Os04g09860.1 |
| GO.0051276 | chromosome organization | 4 | 0.000582 | LOC_Os01g05630.1,LOC_Os01g16414.1,LOC_Os01g67970.1,LOC_Os04g09860.1 |
| GO.0009987 | cellular process | 11 | 0.00114 | LOC_Os01g05630.1,LOC_Os01g16414.1,LOC_Os01g67970.1,LOC_Os01g69030.2,LOC_Os02g03060.1,LOC_Os02g55260.1,LOC_Os04g09860.1,LOC_Os05g29030.1,LOC_Os05g50970.1,LOC_Os06g49430.1,LOC_Os08g06060.1 |
| GO.0006338 | chromatin remodeling | 2 | 0.00372 | LOC_Os01g16414.1,LOC_Os04g09860.1 |
| GO.0016568 | chromatin modification | 3 | 0.005 | LOC_Os01g16414.1,LOC_Os01g67970.1,LOC_Os04g09860.1 |
| GO.0006796 | phosphate-containing compound metabolic process | 5 | 0.00928 | LOC_Os02g03060.1,LOC_Os05g29030.1,LOC_Os05g50970.1,LOC_Os06g49430.1,LOC_Os08g06060.1 |
| GO.0044238 | primary metabolic process | 8 | 0.013 | LOC_Os01g67970.1,LOC_Os01g69030.2,LOC_Os02g03060.1,LOC_Os02g55260.1,LOC_Os05g29030.1,LOC_Os05g50970.1,LOC_Os06g49430.1,LOC_Os08g06060.1 |
| GO.0044237 | cellular metabolic process | 8 | 0.0166 | LOC_Os01g67970.1,LOC_Os01g69030.2,LOC_Os02g03060.1,LOC_Os02g55260.1,LOC_Os05g29030.1,LOC_Os05g50970.1,LOC_Os06g49430.1,LOC_Os08g06060.1 |
| GO.0006952 | defense response | 3 | 0.0179 | LOC_Os01g16414.1,LOC_Os01g67970.1,LOC_Os06g49430.1 |
| GO.0071704 | organic substance metabolic process | 8 | 0.0204 | LOC_Os01g67970.1,LOC_Os01g69030.2,LOC_Os02g03060.1,LOC_Os02g55260.1,LOC_Os05g29030.1,LOC_Os05g50970.1,LOC_Os06g49430.1,LOC_Os08g06060.1 |
| GO.0050896 | response to stimulus | 5 | 0.0205 | LOC_Os01g16414.1,LOC_Os01g67970.1,LOC_Os02g55260.1,LOC_Os06g49430.1,LOC_Os08g06060.1 |
| GO.0000165 | MAPK cascade | 2 | 0.023 | LOC_Os06g49430.1,LOC_Os08g06060.1 |
| GO.0006468 | protein phosphorylation | 3 | 0.023 | LOC_Os02g03060.1,LOC_Os06g49430.1,LOC_Os08g06060.1 |

Table S4. Results of 14 sRNA sequences in Magnaporthe Next-Gen Sequence sRNA database

| Sequence | Len | Sum | Average | Max | Min (>0) | MgCM01 | MgCS03 | MgMM04 | SRNS02 | SRPQ05 | LMg0 | LMg72 | LMg96 | Wt7015 | Wt7015_I | dcl1 | dcl2 | dcl1_2 | dcl2_1 | rdrp13453 | rdrp02748 | rdrp06205 | ago01294_II | ago14873 | ago13617 |
| --- | --- | --- | --- | --- | --- | --- | --- | --- | --- | --- | --- | --- | --- | --- | --- | --- | --- | --- | --- | --- | --- | --- | --- | --- | --- |
| TAGACTTTGATCTGAGCAA | 19 | 1029 | 257 | 754 | 2 | 0 | 2 | 23 | 0 | 0 | 0 | 754 | 250 | 0 | 0 | 0 | 0 | 0 | 0 | 0 | 0 | 0 | 0 | 0 | 0 |
| TGGCAAGTATAGGCCTGTA | 19 | 1145 | 95 | 467 | 7 | 0 | 0 | 0 | 51 | 221 | 0 | 467 | 250 | 12 | 7 | 23 | 0 | 0 | 0 | 0 | 25 | 36 | 7 | 11 | 35 |
| AGCCTGACGATGTCGTTGATGCT | 23 | 627 | 314 | 610 | 17 | 0 | 0 | 17 | 0 | 0 | 0 | 610 | 0 | 0 | 0 | 0 | 0 | 0 | 0 | 0 | 0 | 0 | 0 | 0 | 0 |
| TGGAAGCGTTAGGGGCTTTG | 20 | 811 | 116 | 395 | 2 | 2 | 67 | 0 | 18 | 212 | 0 | 395 | 112 | 0 | 0 | 5 | 0 | 0 | 0 | 0 | 0 | 0 | 0 | 0 | 0 |
| ACGATCTGCAGCGCTTTTCGT | 21 | 3446 | 246 | 1293 | 2 | 2 | 5 | 0 | 145 | 1057 | 0 | 1293 | 350 | 161 | 49 | 60 | 0 | 0 | 0 | 0 | 58 | 71 | 24 | 67 | 104 |
| ACGATCTGCAGCGCTTTTCG | 20 | 2254 | 225 | 1109 | 2 | 2 | 4 | 19 | 218 | 1109 | 0 | 682 | 200 | 0 | 3 | 5 | 0 | 0 | 0 | 0 | 0 | 0 | 0 | 0 | 12 |
| CAGGCGAGGGCGCTCTGCT | 19 | 2108 | 192 | 627 | 2 | 13 | 107 | 228 | 627 | 208 | 214 | 574 | 100 | 0 | 0 | 0 | 15 | 0 | 0 | 20 | 0 | 0 | 2 | 0 | 0 |
| GCACTTGGAAGCATGGGGCT | 20 | 845 | 282 | 682 | 26 | 0 | 0 | 26 | 0 | 0 | 0 | 682 | 137 | 0 | 0 | 0 | 0 | 0 | 0 | 0 | 0 | 0 | 0 | 0 | 0 |
| TAGCGGGGAACTGTGCATG | 19 | 700 | 140 | 467 | 30 | 0 | 56 | 30 | 73 | 74 | 0 | 467 | 0 | 0 | 0 | 0 | 0 | 0 | 0 | 0 | 0 | 0 | 0 | 0 | 0 |
| GGACATGGTTTTGGACGAA | 19 | 1236 | 88 | 467 | 9 | 9 | 12 | 0 | 47 | 165 | 0 | 467 | 125 | 37 | 15 | 32 | 0 | 0 | 0 | 0 | 42 | 57 | 137 | 45 | 46 |
| TACAAGGGACGAAGTGTCT | 19 | 1349 | 104 | 646 | 2 | 2 | 28 | 62 | 0 | 0 | 0 | 646 | 0 | 111 | 109 | 97 | 0 | 0 | 0 | 20 | 79 | 71 | 10 | 45 | 69 |
| AACCCGGAGGTCTCTGGA | 18 | 2291 | 153 | 722 | 6 | 0 | 0 | 0 | 51 | 230 | 0 | 431 | 0 | 62 | 85 | 9 | 92 | 722 | 174 | 164 | 37 | 57 | 6 | 90 | 81 |
| AGTGGTCGTAGACCGCCTGA | 20 | 1933 | 161 | 1126 | 5 | 13 | 21 | 0 | 44 | 1126 | 0 | 359 | 325 | 6 | 10 | 5 | 0 | 0 | 0 | 0 | 8 | 7 | 9 | 0 | 0 |
| CAGGCAGTTGGACTTGACCT | 20 | 889 | 296 | 539 | 28 | 0 | 0 | 28 | 0 | 0 | 322 | 539 | 0 | 0 | 0 | 0 | 0 | 0 | 0 | 0 | 0 | 0 | 0 | 0 | 0 |
